# Efficacy and evaluation of therapeutic exercises on adults with Parkinson’s disease: a systematic review and network meta-analysis

**DOI:** 10.1186/s12877-022-03510-9

**Published:** 2022-10-21

**Authors:** Yong Yang, Guotuan Wang, Shikun Zhang, Huan Wang, Wensheng Zhou, Feifei Ren, Huimin Liang, Dongdong Wu, Xinying Ji, Makoto Hashimoto, Jianshe Wei

**Affiliations:** 1 Institute for Brain Sciences Research, School of Life Sciences, Shunhe District, Henan University, 85 Minglun Rd, Kaifeng City, 475001 China; 2 Laboratory of Kinesiology and Rehabilitation, School of Physical Education and Sport, Henan University, Kaifeng, 475001 China; 3 Department of Police Physical Education, Jiangsu Police Institute, Nanjing, China; 4 Department of Orthopedics, the Second Affiliated Hospital of Air Force Medical University, Xi ’an, China; 5 College of Physical Education, Nanjing Xiao-Zhuang University, Nanjing, China; 6 Department of Physical Education, Beijing Language and Culture University, Beijing, China; 7 Henan Medical School, Parkinson’s Disease Research Center, Henan University, Kaifeng, China; 8 Henan International Joint Laboratory for Nuclear Protein Regulation, Henan Medical School, Henan University, Kaifeng, China; 9 Tokyo Metropolitan Institute of Medical Science, Setagaya Ku, 2-1-6 Kamikitazawa, Tokyo, 1560057 Japan

**Keywords:** Physical activity, Exercise, Parkinson' s disease, Quality of life, Evidence-based medicine

## Abstract

**Background:**

Exercises are an effective treatment in Parkinson’s disease (PD), but there is still controversy over which types should be used. We aimed to compare and rank the types of exercise that improve PD symptoms by quantifying information from randomised controlled trials.

**Methods:**

We performed a systematic review and network meta-analysis and searched PubMed, MEDLINE, Embase, PsycINFO, Cochrane Central Register of Controlled Trials (CENTRAL), Web of Science, and China National Knowledge Infrastructure (CNKI) from their inception date to June 30, 2022. We included randomized controlled trials of 24 types of exercise for the interventional treatment of adults (≥ 50 years old) with PD. Effect size measures were standardized mean differences (SMDs) with 95% credible intervals (CrIs). The confidence of evidence was examined using Confidence in Network Meta-Analysis (CINeMA).

**Results:**

We identified 10 474 citations and included 250 studies involving 13 011 participants. Results of NMA showed that power training (PT) had the best benefits for motor symptoms compared with the control group (CON), with SMDs (95% CrI) (-1.46, [-2.18 to -0.74]). Body weight support treadmill training (BWS_TT) showed the best improvement in balance (1.55, [0.72 to 2.37]), gait velocity (1.15 [0.57 to 1.31]) and walking distance (1.96, [1.18 to 2.73]), and robotic assisted gait training (RA_GT) had the most benefits for freezing of gait (-1.09, [-1.80 to -0.38]). For non-motor symptoms, Dance showed the best benefits for depression (-1.71, [-2.79 to -0.73]). Only Yoga significantly reduced anxiety symptom compared with CON (-0.53, [0.96 to -0.11]). Only resistance training (RT) significantly enhanced sleep quality and cognition (-1.42, [-2.60 to -0.23]; 0.51, [0.09 to 0.94]). For muscle strength, PT showed the best advance (1.04, [0.64 to 1.44]). For concern of falling, five types of exercise were more effective than CON.

**Conclusions:**

There is low quality evidence that PT, Yoga, BWS_TT, Dance, and RT are the most effective treatments, pending outcome of interest, for adults with PD.

**Trial registration:**

PROSPERO (CRD42021220052).

**Supplementary Information:**

The online version contains supplementary material available at 10.1186/s12877-022-03510-9.

## Introduction

Neurological disorders are the leading causes of disability worldwide, and the prevalence rate of Parkinson’s disease (PD) is faster than that of other neurological diseases [1]. The number of PD cases worldwide is expected to double from 6.2 million in 2015 to 12.9 million by 2040 [2]. PD is characterized by the motor symptoms such as bradykinesia, rigidity, tremor, gait dysfunction, and postural instability, and non-motor symptoms caused by mood disorders (depression and anxiety), cognitive, and sleep disorders [3]. The loss of dopamine caused by the prominent early death of dopaminergic neurons in the substantia nigra pars compacta (SNpc) is the intrinsic pathology [4]. Drug treatments are the primary method of traditional PD management [5, 6]. However, even with optimal drug treatments, PD patients can experience balance and gait disorders, which often leads to falls and serious potential complications [7–9].

Epidemiological studies have supported that moderate to vigorous intensity exercise can reduce the risk of PD [10, 11]. Additionally, many studies and published reviews on the effects of exercise on normal ageing and PD support the benefits of exercises, and environmental enrichment [12–14]. In the past decade, exercises have been developed to optimize PD treatments: balance and gait training based on virtual reality environment (VR), body weight support treadmill training (BWS_TT), robotic-assisted gait training (RA_GT), and balance and gait training with the external cue or attention (BGT_ECA) (Additional file 1: Appendix 5, pp 31–32).

Previous meta-analysis examined the efficacy of exercise compared with usual care [15] or between different types of exercise [16, 17]. However, these approaches provide limited insight into the benefits hierarchy of exercises because treatment effects are only estimated and presented from a subset of relevant treatment comparisons. Furthermore, the absence of head-to-head clinical trials of the comparisons of some exercises creates uncertainty for decision-makers. The network meta-analysis is regarded as the highest level of evidence in the treatment guidelines [18]. This is because it includes the comparisons of direct and indirect treatment effects in a single analysis [19], which can provide a complete insight into the clinical efficacy of exercises on PD patients. One network meta-analysis of the effects of exercises on PD patients was published, but it only included limited interventions (only six exercise types, such as Dance, Qigong, Tango, resistance training (RT), Tai Chi (TC) and yoga), and lacked evidence of the effects of exercises on non-motor symptoms of PD patients [20].

The systematic review and network meta-analysis (NMA) in this study combined 11 effectiveness outcomes from 254 randomized controlled trials (RCTs) on 24 exercise types and control groups, with motor symptoms (UPDRS III) as the primary outcome. This study aims to compare the effects of different exercises on motor symptoms, non-motor symptoms, muscle strength, and fall prevention in the treatment of PD patients. This study also provides a better evidence-based basis for decision-makers.

## Methods

We performed a systematic review and network meta-analysis according to the Preferred Reporting Items for Systematic Reviews and Meta-analyses (PRISMA) statement (Additional file 1: Appendix 1 pp 1–4) [21]. In addition, this study has been registered with PROSPERO, under the number CRD42021220052 (Additional file 1: Appendix 2 pp 5–17).

### Data sources and searches

We searched the data in PubMed, MEDLINE, Embase, PsycINFO, Cochrane Central Register of Controlled Trials (CENTRAL), Web of Science, and China National Knowledge Infrastructure (CNKI) from their inception date to June 30, 2022, with no language restrictions. The keywords of Parkinson’s disease (PD) and various exercise names were combined in data searches (Additional file 1: Appendix 3 pp 18–28). The reference list of the included studies and previous reviews were screened for additional studies.

### Study selection

The inclusion criteria were based on the PICOS (participants, interventions, comparators, outcomes, and study design) approach [21]. The participants were diagnosed as PD, the mean age ≥ 50 years, Hoehn and Yahr stages < 4. The exercises were divided into 24 types according to their content. The specific type of exercise training was determined by the group names chosen by authors and the definitions presented in Additional file 1: Appendix 5. The non-exercise training treatment group included health education and usual care. Besides, for head-to-head studies, the comparator may be any of the 24 exercise types. Studies should include at least one of the outcome measures of interest: the primary outcome was the changes in motor symptoms of PD as measured by rating scales, such as the unified Parkinson’s disease rating scale III (UPDRS III). The secondary outcomes were the disorders of balance, gait velocity and walking distance, freezing of gait in motor symptoms, and depression, anxiety, sleep disorder, and cognitive impairment in non-motor symptoms, measured by published rating scales. In addition, muscle strength, and concern of falling were also outcomes of our evaluation (Additional file 1: Appendix 4). In the study design, we included published RCTs (individual design, cluster design, or the first half of crossover) and compared an exercise training intervention with a non-exercise training intervention or another exercise training intervention for network meta-analysis. We excluded studies on the acute effects of a single exercise session on PD patients. In addition, studies that did not clearly describe the types of exercises and the duration of training were also excluded.

### Data abstraction and quality assessment

After all relevant articles were searched in the mentioned databases; they were stored in an EndNote X9 reference manager. Three pairs of investigators (YY, GTW, HW, WSZ, SKZ, and FFR) independently screened the search results, retrieved full-text articles, and extracted the relevant information from the included trials. For non-English literatures, we will invite corresponding language professionals to help translate. The risk of bias for primary outcomes in RCTs was assessed using the TESTEX scale [22]. We classified the quality evaluation results of each included study into low-risk (TESTEX scale ≥ 11 points), moderate-risk (TESTEX scale 6–10 points), and high-risk (TESTEX scale ≤ 5 points). Any discrepancies were resolved by a panel of investigators within the review team (HML, DDW, XYJ, MH, and JSW) through consensus and arbitration. In addition, we examined the confidence of evidence using the Confidence of Network Meta-Analysis (CINeMA) web application, which grades the confidence of the results as high, moderate, low, and very low [23].

### Data synthesis and analysis

Based on the R statistical environment (V.3.6.3, www.r-project.org), we used the gemtc and rjags packages (Additional file 1: Appendix 6 pp 33–39) to perform network meta-analysis combining direct and indirect comparisons in the Bayesian hierarchical model [24]. Due to the use of different rating scales, the efficacy-related continuous outcomes presented the standardized mean differences (SMDs). A random-effects model was used to combine data, and the surface under the curved cumulative ranking probabilities was used to rank the treatments. The transitivity assumption was evaluated by comparing the distribution of potential effect modifiers (the publication year, mean age, years of diagnosis, Hoehn and Yahr stages, sample size, and percentage of males) among studies grouped by comparison (Additional file 1: Appendix 7 pp 41–6). We used the tau square (τ^2^) test and I^2^ to analyze the statistical heterogeneity between the studies. We statistically assessed inconsistency through the design-by-treatment test for global consistency [25] and separated indirect from direct evidence (SIDE test) [26] using the R netmeta package [27]. Besides, we evaluated the robustness of the treatment effects for the primary outcome in the network meta-regression through the publication year, mean age, years of diagnosis, Hoehn and Yahr stages, percentage of males, sample size, exercise duration, exercise frequency, time of the single session, and ON/OFF during testing. We assessed the sensitivity of our findings by repeating each network meta-analysis after excluding studies with TESTEX scale < 11 points, sample sizes less than 20, exercise duration less than 4 weeks and more than 24 weeks, exercise frequency less than 2 and more than 4, OFF state during testing, Chinese studies, and extracted data using GetData with estimated standard deviation value. We used comparison adjusted funnel plot for the primary outcome to investigate the presence of small study effect [28].

## Results

### Study characteristics and quality assessment

**Fig. 1:**
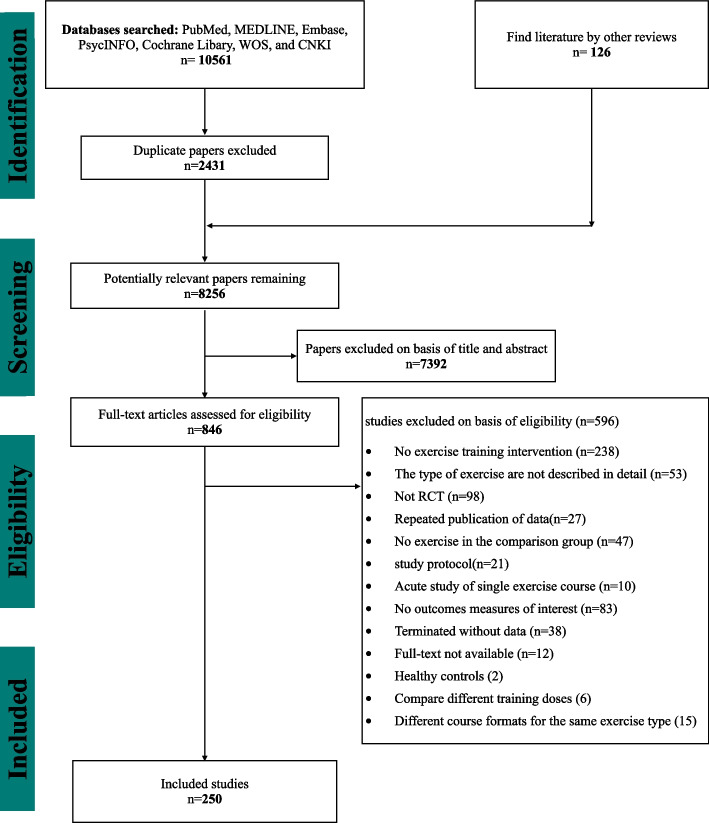
PRISMA Flow diagram of the search process for studies. *RCT* randomized controlled trials

The flow diagram of the search process for the systematic review study is presented in Fig. 1. After excluding 7,392 reports based on their titles and abstracts, 846 full-text articles were retrieved. Three pairs of investigators confirmed the outcomes of interest by viewing the full texts and finally included 250 studies with 13,011 participants, 7,340 (56.41%) of whom were male, and 5,671 (43.59%) were female. The sample size ranged from 4 to 381 with a mean disease duration of 6.84 years (SD 2.30) and a mean of 2.35 (SD 0.42) for Hoehn and Yahr stages (Table 1 and Additional file 1: Appendix 15). The 250 studies were divided into 24 exercise types based on their exercise content (Additional file 1: Appendix 5). The exercise period ranged from 1 to 96 weeks (mean period 12.34 weeks, SD 15.69), and the frequency of exercise training per week ranged from 1–7 (mean frequency 3.09, SD 1.76), and the total time of the single session ranged from 15 to 180 min (mean time 54.13 min, SD 19.44) (Additional file 1: Appendix 15). Overall, 119 studies (47.6%) were moderate risk of bias, and 131 (52.4%) studies were low risk of bias (Additional file 1: Appendix 9 p 117–123).

**Table 1:** Characteristics of the included studies

Characteristics	Number (%) of studies (*n* = 250)
Mean age of participants	
50–60	16 (6.4)
60–64.9	54 (21.6)
65–69.9	112 (44.8)
70–74.9	56 (22.4)
75–79.9	10 (4)
Not reported	2 (0.8)
Year of publication	
1996–99	2 (0.8)
2000–09	23 (9.2)
2010–22	225 (90)
Sample size (No of participants)	
< 10	22 (8.8)
10–19	121 (48.4)
20–29	60 (24)
30–50	27 (10.8)
50–99	16 (6.4)
≥ 100	4 (2)
Exercise period (weeks)	
< 12	163 (65.2)
12–23	57 (22.8)
25–48	20 (8)
> 48	6 (2.4)
Average disease duration (years)	
< 5	43 (17.2)
5–10	154 (61.6)
> 10	16 (6.4)
Not reported	41 (16.4)
Disease grade (Hoehn and Yahr stage)	
1–1.9	28 (11.2)
2–2.9	165 (66)
3–3.9	27 (10.8)
Not reported	30 (12)
Average % of women	
< 50	162 (64.8)
50–59	42 (16.8)
60–69	13 (5.2)
≥ 70	12 (4.8)
Not reported	21 (8.4)

### Network meta-analysis

#### Motor symptoms

**Fig. 2:**
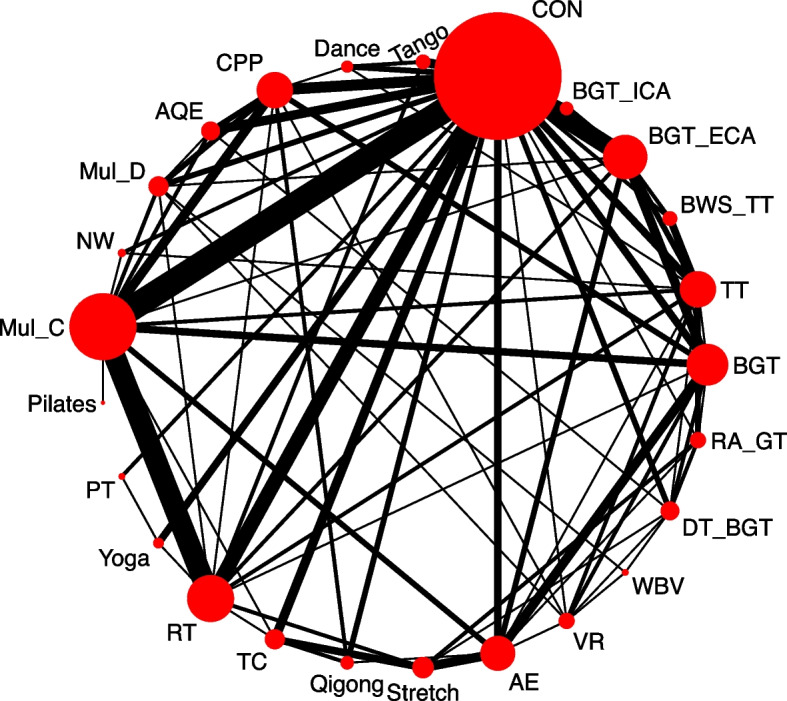
Network plot of clinical symptoms. The size of the nodes corresponds to the number of participants randomized to each Exercise type. Exercise type with direct comparisons are linked with a line; its thickness corresponds to the number of trials evaluating the comparison. *AE* Aerobic Exercise, *AQE* Aquatic Exercise, *BGT* Balance and Gait Training, *BGT_ECA* Balance and Gait Training with External Cue or Attention, *BGT_ICA* Balance and Gait Training with Internal Cue or Attention, *BWS_TT* Body Weight Support Treadmill Training, *CON* Control group, *CPP* Classic Physiotherapy Program, *DT_BGT* Dual Task Balance and Gait Training, *Mul_C* Multicomponent Exercise Program, *Mul_D* Multidisciplinary Exercise Program, *NW* Nordic Walking, *PT* Power Training, *RA_GT* Robotic Assisted Gait Training, *RT* Resistance Training, *TC* Tai Chi, *TT* Treadmill Training, *VR* Virtual Reality, *WBV* Whole Body Vibration

**Fig. 3:**
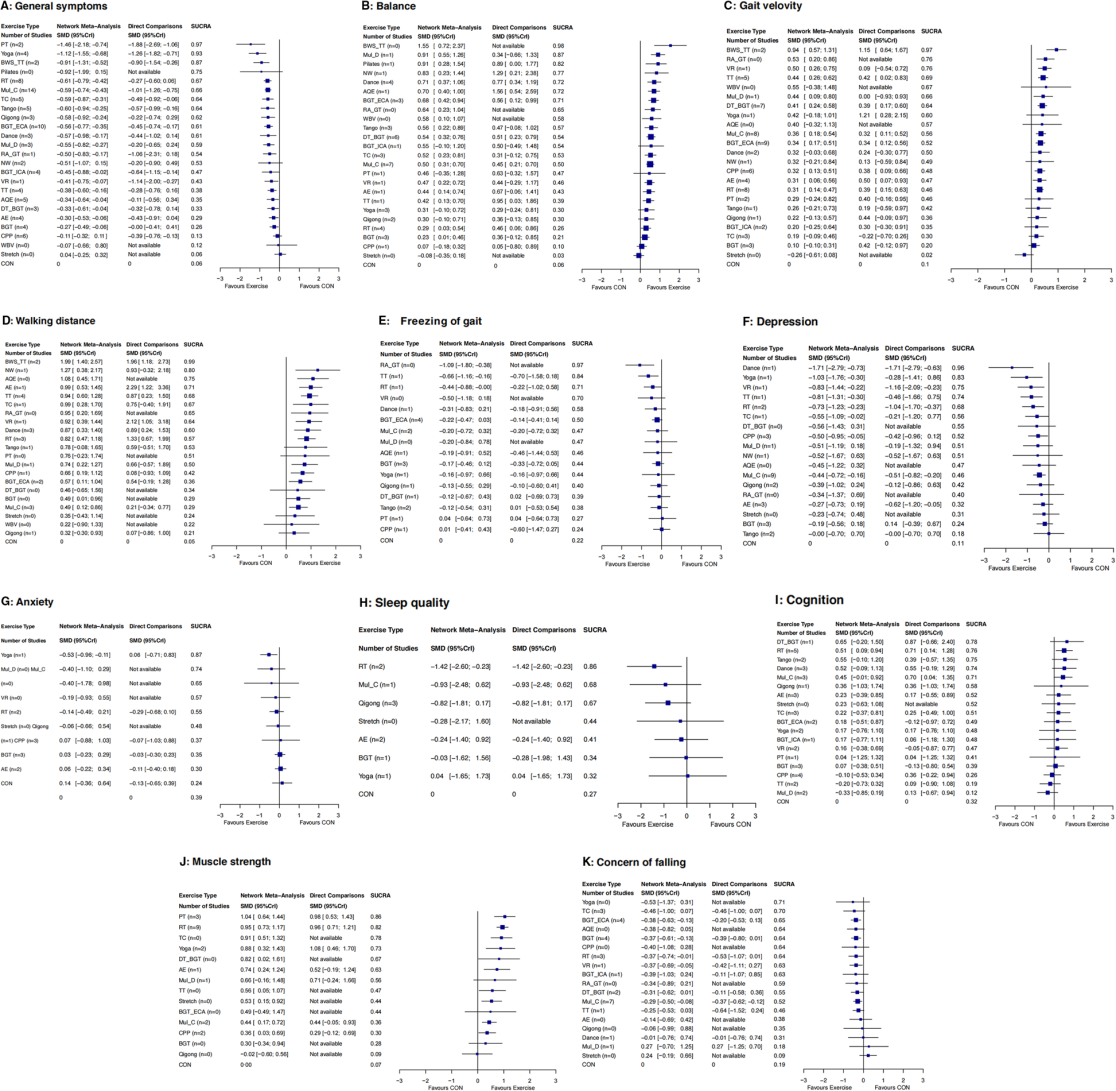
Forest plot change in efficacy of 11 outcomes. Exercise type are ranked according to the surface under the curved cumulative ranking probabilities. Treatments crossing the y-axis are not significantly different from CON. The n value represents the number of studies that were directly compared to the control group. *SMD* standardized Mean Difference, *CrI* Credible Interval, *AE* Aerobic Exercise, *AQE* Aquatic Exercise, *BGT* Balance and Gait Training, *BGT_ECA* Balance and Gait Training with External Cue or Attention, *BGT_ICA* Balance and Gait Training with Internal Cue or Attention, *BWS_TT* Body Weight Support Treadmill Training, *CON* Control group, *CPP* Classic Physiotherapy Program, *DT_BGT* Dual Task Balance and Gait Training, *Mul_C* Multicomponent Exercise Program, *Mul_D* Multidisciplinary Exercise Program, *NW* Nordic Walking, *PT* Power Training, *RA_GT* Robotic Assisted Gait Training, *RT* Resistance Training, *TC* Tai Chi, *TT* Treadmill Training, *VR* Virtual Reality, *WBV* Whole Body Vibration

Figure 2 shows the network plot of eligible comparisons for motor symptoms. Available results were shown in 141 (56.4%) studies with 7 983 (61.4%) participants for changes of motor symptoms in the primary outcome. Except for Pilates, whole body vibration (WBV), and Stretch, other types had at least one direct comparison with the control group (CON) (Fig. 3A). Compared with the CON, 19 (79%) of 24 exercise types significantly relieved the motor symptoms of participants, and the SMDs (95% Credible Interval (CrI)) ranged between -1.46 (-2.18 to -0.74) for power training (PT) to -0.27 (-0.49 to -0.06) for balance and gait training (BGT) (Fig. 3A), and PT ranks first (the surface under the curved cumulative ranking probabilities (SUCRA): 0.97). In addition, the NMA results showed that PT, Yoga, BWS_TT, RT, and multicomponent Exercise Program (Mul_C) could better relieve their motor symptoms than many other exercise types (more than 2 types) (Table 2). Differences between the remaining exercise types were mostly small or very uncertain.

**Table 2:**
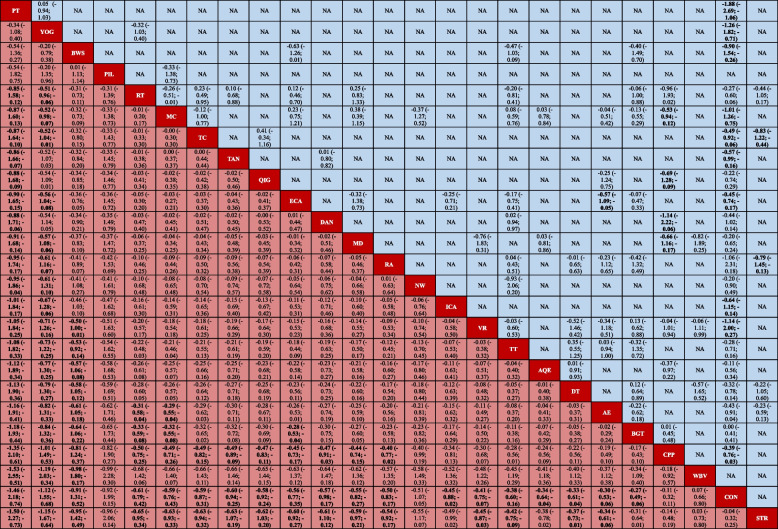
League table of motor symptoms

All results are presented in the form of SMD (95% CrI). Exercise types are ranked according to the surface under the curve cumulative for overall symptoms starting with the best from left to right. The results of the network meta-analysis are showed in the lower left part, and results from pairwise comparisons in the upper right half (if available). Cells shown in bold indicate significant results

*NA* Not available, *SMD* Standardized Mean Difference, *CrI* Credible Interval, *AE* Aerobic Exercise, *AQE* Aquatic Exercise, *BGT* Balance and Gait Training, *BWS* Body Weight Support Treadmill Training, *CON* Control group, *CPP* Classic Physiotherapy Program, *DAN* Dance, DT Dual Task Balance and Gait Training, *ECA* Balance and Gait Training with External Cue or Attention, *ICA* Balance and Gait Training with Internal Cue or Attention, *MC* Multicomponent Exercise Program, *MD* Multidisciplinary Exercise Program, *PIL* Pilates, *NW* Nordic Walking, PT Power Training, *QIG* Qigong, *RA_GT* Robotic Assisted Gait Training, *RT* Resistance Training, *STR* Stretch, *TAN* Tango, *TC* Tai Chi, *TT* Treadmill Training, *VR* Virtual Reality, *WBV* Whole Body Vibration, *YOG* Yoga

#### Balance

One hundred thirteen studies (45.2%) with 5 201 participants (40.0%) showed available results of changes in their balance abilities. Compared with CON, 18 (75%) exercise types significantly improved their balance abilities, with SMDs (95% CrI) ranging from 1.55 (0.72 to 2.37) for BWS_TT to 0.23 (0.01 to 0.46) for BGT (Fig. 3B), and BWS_TT ranks first (SUCRA: 0.98). BWS_TT, multidisciplinary exercise program (Mul_D), Pilates, Dance, aquatic exercise (AQE), BGT_ECA, RA_GT, and dual task balance and gait training (DT_BGT) were significantly more beneficial than other types of exercise in improving the balance abilities of PD patients (Additional file 1: Appendix 10 pp 124–6).

#### Gait velocity

Available results found in 106 studies (42.4%) with 4 780 participants (36.7%) showed their gait velocity changes (23 exercise types). Compared with CON, 11 (48%) exercise types significantly improved the gait velocity of the participants, with SMDs (95% CrI) ranging from 1.15 (0.57 to 1.31) for BWS_TT to 0.31 (0.14 to 0.47) for RT (Fig. 3C), and BWS_TT ranks first (SUCRA: 0.97). Only BWS_TT was significantly more effective than other types of exercise in improving the gait velocity of patients (Additional file 1: Appendix 10 pp 127–129). Differences between the remaining exercise types were mostly small or very uncertain.

#### Walking distance

A total of 55 studies (22%) with 2 955 participants (22.7%) showed available results of the changes in their walking distance (21 exercise types). Compared with CON, 15 (71.4%) exercise types significantly improved the walking distance of the participants, with SMDs (95% CrI) ranging from 1.96 (1.18 to 2.73) for BWS_TT to 0.49 (0.12 to 0.86) for Mul_C (Fig. 3D). The effects of BWS_TT was more significant than other exercise types in improving the walking distance of patients (Additional file 1: Appendix 10 pp 130–2). The differences between the remaining exercise types were mostly very uncertain.

#### Freezing of gait

Available results found in 35 studies (14%) with 1 146 participants (8.8%) showed the changes of freezing of gait (16 exercise types). Compared with CON, 3 (19%) exercise types significantly relieved their freezing of gait, with SMDs (95% CrI) ranging from -1.09 (-1.80 to -0.38) for RA_GT to -0.44 (-0.88 to -0.00) for RT (Fig. 3E), and RA_GT ranks first (SUCRA: 0.97). Only RA_GT was significantly more effective than other types of exercise in relieving the freezing of gait of PD patients (Additional file 1: Appendix 10 pp 133–5).

#### Non-motor symptoms

In terms of non-motor symptoms, 34 studies (13.6%) with 2 007 participants (15.4%) showed available results of the changes of depression (18 exercise types). Compared with CON, 8 (44%) exercise types significantly relieved the depression of the participants, with SMDs (95% CrI) ranging from -1.71 (-2.79 to -0.73) for Dance to -0.44 (-0.72 to -0.16) for Mul_C (Fig. 3F). Only the effect of Dance was more obvious than other exercise types in relieving the depression of patients (Additional file 1: Appendix 10 pp 136–8).

Only 13 studies (5.2%) with 757 participants (5.8%) showed available results of the changes in anxiety (10 exercise types). Compared with CON, only Yoga significantly relieved the anxiety of the participants (SMDs 95% CrI: -0.53 (-0.96 to -0.11)) (Fig. 3G) and was significantly more effective than other exercise types in this aspect (Additional file 1: Appendix 10 pp 139–140).

Only 10 studies (4%) with 452 participants (3.5%) showed available results of changes in sleep quality (7 exercise types). Compared with CON, only RT significantly improved the sleep quality (SMDs 95% CrI: -1.42 (-2.60 to -0.23)) (Fig. 3H). Besides, the results did not indicate which exercise was significantly better than others in improving sleep quality (Additional file 1: Appendix 9 pp 141–2).

A total of 50 studies (20%), with 3 044 participants (23.4%), showed available results of the changes in the cognitive abilities (18 exercise types). Compared with CON, only RT significantly improved their cognitive abilities (SMDs 95% CrI: 0.51 (0.09 to 0.94)) (Fig. 3I). Additionally, RT was significantly superior to other types of exercise in improving cognitive abilities (Additional file 1: Appendix 10 pp 143–5).

#### Muscle strength

A total of 25 studies (10%) with 856 participants (6.6%) showed available results of the muscle strength changes (14 exercise types). Compared with CON, 10 (71.4%) exercise types significantly improved the muscle strength of participants, with SMDs (95% CrI) ranging from 1.04 (0.64 to 1.44) for PT to 0.40 (0.15 to 0.64) for classic physiotherapy program (CPP) (Fig. 3J). The NMA results showed that the improvement effect of PT, RT, and TC in muscle strength was significantly greater than that of other exercise types (Additional file 1: Appendix 10 pp 146–148).

#### Concern of falling

A total of 49 studies (19.6%) with 2 858 participants (22%) showed available results of the changes in concern of falling (18 exercise types). Compared with CON, 5 (28%) exercise types significantly reduced the concern of falling, with SMDs (95% CrI) ranging from -0.38 (-0.63 to -0.13) for BGT_ECA to -0.29 (-0.50 to -0.08) for Mul_C (Fig. 3K). Mostly, differences between the exercise types were small or very uncertain (Additional file 1: Appendix 10 pp 149–151).

### Heterogeneity, inconsistency and sensitivity analysis

The heterogeneity of most outcomes was moderate to high (Additional file 1: Appendix 11 p 152). Only depression was significantly inconsistent according to the design-by-treatment interaction test. The SIDE test of all outcomes showed that the percentage of comparisons with evidence of inconsistency ranged from 0–9.1% (Additional file 1: Appendix 11 p 153). Potential threats to the transitivity assumption and the source of heterogeneity from baseline characteristics, exercise training doses, and the ON/OFF state of outcome tests of the included studies were resolved by meta-regression and sensitivity analysis of the primary outcome. Exercise period and frequency were significant factors affecting the network meta-analysis results (Additional file 1: Appendix 13 pp 166–176). Sensitivity analyses excluded studies with TESTEX scale < 11 points (*n* = 89), sample size less than 20 (*n* = 82), exercise duration less than 4 weeks and more than 24 weeks (*n* = 23), exercise frequency less than 2 and more than 4 (*n* = 42), OFF state during testing (*n* = 19), Chinese studies (*n* = 6) and extracted data using GetData with estimated standard deviation values (*n* = 21) not affecting the results (Additional file 1: Appendix 14 pp 177–184).

### Certainty of the evidence and small study effects

**Fig. 4:**
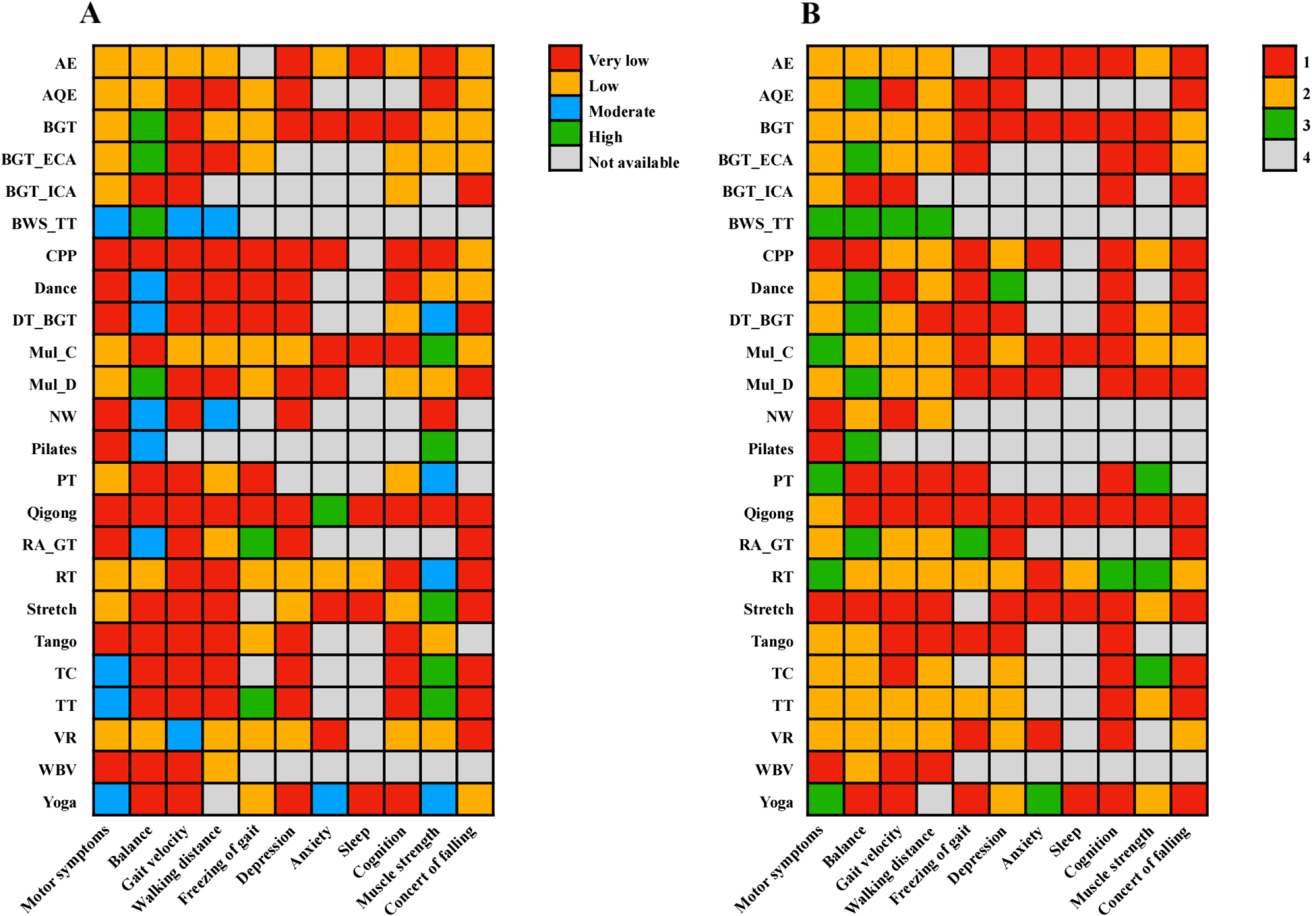
Heat map in 2 dimensions of 11 outcomes for 24 exercise types. A: Confidence in evidence. B: Whether the results are significantly better than the control group or many exercise types (≥ 2 exercise types), 1 means vs. CON (*p* > .05), 2 means vs. CON (*p* < .05), 3 means vs. CON and many exercise types (*p* < .05), 4 not available. *AE* Aerobic Exercise, *AQE* Aquatic Exercise, *BGT* Balance and Gait Training, *BWS* Body Weight Support Treadmill Training, *CON* Control group, *CPP* Classic Physiotherapy Program, *DAN* Dance, *DT* Dual Task Balance and Gait Training, *ECA* Balance and Gait Training with External Cue or Attention, *ICA* Balance and Gait Training with Internal Cue or Attention, *MC* Multicomponent Exercise Program, *MD* Multidisciplinary Exercise Program, *PIL* Pilates, *NW* Nordic Walking, *PT* Power Training, *QIG* Qigong*, RA_GT* Robotic Assisted Gait Training, *RT* Resistance Training, *STR* Stretch, *TAN* Tango, *TC* Tai Chi, *TT* Treadmill Training, *VR* Virtual Reality, *WBV* Whole Body Vibration, *YOG* Yoga

For all outcomes, the certainty of the evidence was overall low (Fig. 4A). For the primary outcome, we judged the confidence of the evidence of 88% for comparison with CON was low or very low. For the comparisons of two exercise types, the confidence of evidence of 57% was very low (Additional file 1: Appendix 16 pp 189–208). In addition, our comparison-adjusted funnel plot had good symmetry, and the linear fitting line (green) was not perpendicular to quadrant 0. Therefore, no small study effect was found in the primary outcome (Additional file 1: Appendix 12 pp 165).

## Discussion

In this systematic review and network meta-analysis, 250 RCTs were included, and 13 011 participants were randomly assigned to 24 different exercise types or control groups. The study results showed that 19 (79%) of 24 exercise types significantly relieved the motor symptoms of participants, and PT, Yoga, BWS_TT, RT and Mul_C more effective than many other exercise types in this aspect. BWS_TT showed the best effect in improving the balance, gait velocity and walking distance of participants, and RA_GT had the most benefits in relieving their freezing of gait. For non-motor symptoms, Dance showed the best benefits for depression. Only Yoga significantly relieved the anxiety symptom, and only RT significantly enhanced the sleep quality and cognitive abilities of participants. For muscle strength, PT showed the best effects. In the concern of falling, five types of exercise (BGT_ECA, BGT, RT, VR, Mul_C) were more effective than CON, but their SMD values were small. The meta-regression results indicated that exercise period and frequency could dramatically affect the effect of exercises on the improvement of motor symptoms, with ≥ 24 weeks and 5 times/week being more effective. However, for all outcomes, the certainty of the evidence was overall low.

### Detailed consideration of better performing exercise types

#### Motor symptoms

For motor symptoms, PT showed the best benefits, and ranked first according to the SUCRA (Fig. 3A). In the form of PT that performs concentric contraction as quickly as possible, the muscle strength of PD patients (SMD 95% CrI: 1.04 (0.64 to 1.44), Fig. 3J) and the neuromuscular adaptation of healthy elderly [29] and EMG activity levels was improved [30]. Previous studies showed that a stronger relationship between muscle power and physical function in older adults than muscle strength [31–33]. In addition, many studies have demonstrated that PT with concentric contractions performed at the fastest possible rate is significantly superior to other types of exercise (e.g., RT) in improving muscle power, speed of movement, and neuromuscular activity in older adults [34, 35]. All these may be the main reasons why PT additionally relieves the motor symptoms, even though there is no direct evidence of the internal mechanism by which PT improves the overall PD symptoms. However, since the evidence was only from two studies, the confidence of evidence was low. More homogeneous studies are needed in the future to validate our findings.

Our results showed that Yoga, BWS_TT, RT and Mul_C also significantly improved motor symptoms in PD patients compared with many other exercise types. Previous studies have shown that yoga is a complex form of exercise, such as movement initiation, moving at different speeds, static and dynamic balance training, and flexibility training. These had significant effects on bradykinesia and stiffness of the upper and lower extremities [36]. Although the mechanisms for improvement with yoga have not been examined yet, it is reasonable to postulate that yoga exercise may mediate the function of basal ganglia. Since both bradykinesia and rigidity are considered a deficiency in nigrostriatal dopamine, yoga may facilitate the activity of dopaminergic neurons, thereby reducing bradykinesia and rigidity, and ultimately improving motor symptoms in PD patients. At the same time, Mul_C is also a complex form of exercise, involving balance, muscle strength, cardiopulmonary exercise and flexibility training. Previous studies have showed that Mul_C significantly improves motor symptoms in PD patients, and the UPDRS III decrease (6.3–9.8 points) [37, 38] exceeds the minimal clinically important difference (3.1 points) [39], and also exceeds high-intensity aerobic exercise (AE) [40], BGT [41] and Stretch [42], but less than PT and Yoga [43]. In addition, BWS_TT may be a good option for PD patients who cannot receive conventional ground gait training due to severe postural instability, orthostatic hypotension, or balance impairment [44]. Compared to traditional treadmill training, BWS_TT provides more repetitions, higher exercise intensity, and task-oriented exercises in the same amount of time [45]. Previous studies have showed that BWS_TT was a promising rehabilitation modality for improving gait disturbance, balance dysfunction, and postural instability in PD patients [46, 47]. The results of Miyai, Fujimoto, Ueda, Yamamoto, Nozaki, Saito, Kang [48] showed that BWS_TT had a greater positive effects on walking, motor performance, and activities of daily living compared to conventional physiotherapy. Furthermore, the results of Fisher, Wu, Salem, Song, Lin, Yip, Cen, Gordon, Jakowec, Petzinger [49] suggested that high-intensity BWS_TT had a positive effects on normalizing cortical motor excitability in PD patients. In conclusion, BWS_TT had an important role in improving motor functional abilities such as postural stability, gait and balance function in PD patients. For RT, previous studies showed effective improvement in muscle strength in PD patients [50, 51], which is consistent with our results that RT significantly improved muscle strength in PD patients (SMD 95% CrI: 0.95 (0.73 to 1.17), Fig. 3J). In addition, previous study showed that RT (12 weeks, 24 sessions) effectively improved the EMG amplitude of the vastus lateralis and the motor unit discharge frequency of the gastrocnemius medialis in PD patients, and ultimately improved the knee and ankle rate of torque development (RTD) in PD patients. These findings are interesting because basal ganglia regions (i.e., the internal globus pallidus and subthalamic nucleus) are directly associated with RTD [52]. To sum up, these may be the reason why Yoga, BWS_TT, RT and Mul_C in this study significantly improved the motor symptoms of PD patients compared with many other types of exercise.

#### Balance, gait velocity, walking distance and freezing of gait

BWS_TT ranked first according to the SUCRA on the overall balance ability, gait velocity and walking distance of PD patients (Fig. 3B, C). As described above, compared with the traditional TT, BWS_TT has a higher TT speed [45, 49]. In addition, the BWS_TT increases the safety of PD patients during training due to the body support equipment, making them dare to move forward with a larger stride [53]. It is clear that BWS_TT is beneficial in improving the balance ability, gait velocity and walking ability of PD patients. In addition, Previous studies showed that specific ability tests were used as training content, and specific skills were improved to the greatest extent [54]. In our study, the gait velocity was assessed using the 3-20 m walking test (speed or time), and the walking distance was assessed using the 2-6 min walking test. Therefore, the test content similar to BWS_TT may be additionally improved. The effect of BWS_TT on freezing of gait of PD patients is unclear (no relevant studies). Our results showed that only RA_GT, TT, and RT significantly improved the symptoms of freezing of gait, and the effect of RA_GT was more significant than other types (Fig. 4B). A training mode similar to BWS_TT may be the key for RA_GT to improve the freezing of gait of PD patients. However, control studies were unavailable, thus this evidence was entirely indirectly obtained (Fig. 3E). In addition, as the upper limit of the CrI of RT is 0, its effect in improving the freezing of gait was not so stable.

#### Non-motor symptoms

For non-motor symptoms, 8 (44%) exercise types significantly relieved the symptom of depression. Previous studies have shown that exercises have important effects on depression by increasing the release of β-endorphins, the availability of brain neurotransmitters (such as serotonin, dopamine, and norepinephrine), or by increasing brain-derived neurotrophic factors [55]. Besides, exercises can improve the self-esteem, self-evaluation, and sense of accomplishment of people [56], and exercises are positively related to self-efficacy [57]. These may be internal reasons why exercises reduce the depression symptom of PD patients. Our results showed that only Dance had a more significant effect in alleviating depression than other types (Fig. 4B). The increased social interaction during the group exercise therapy may improve the mood of PD patients [58], which may explain the phenomenon that dancing in a group format additionally improves the depression symptoms. In our analysis, only Yoga significantly relieved the anxiety symptom and showed significantly better effects than other types (Fig. 4B). Additional mindfulness practice during Yoga may be a critical skill to effectively alleviate stress and anxiety symptoms [59]. Only RT significantly improved the sleep quality of PD patients. Previous studies have shown that resistance training had a moderate to a large beneficial effect on the sleep quality of the elderly [60], which was consistent with the results of our research on PD patients. These findings also have a certain significance in clinical practices because sleep latency, mid-sleep disturbance, and sleep efficiency are associated with increased mortality [61]. In the effect of exercises on the cognitive ability of PD patients, the results showed that only RT significantly improved the cognitive ability of PD patients. However, the lower limit of CrI of the combined SMD values approached quadrant 0 (Fig. 3I), which led to a less stable effect of RT in improving the cognitive ability of PD patients. More studies are needed for the verification of the RT effect in the future.

#### Muscle strength, and concern of falling

For muscle strength, our results showed that PT ranked first according to the SUCRA, and significantly better than many other exercise types (Figs. 3J and 4B). In the past ten years, a large number of studies have confirmed that PT using a lower training load (40–60% 1RM) and completing contractions as quickly as possible is significantly better than RT in improving the power, muscle strength, movement speed and neuromuscular activity of older adults [34, 35, 62–64]. All these may be the main reasons why PT additionally improved muscle strength of PD patients. In addition, our results showed that RT and TC were also significantly better than many other exercise types for improving muscle strength in PD patients. RT applies a certain exercise intensity during the repetition of certain movements, allows a certain time to recover between movements, and adjusts the exercise intensity as the muscle strength increases. The purpose is to improve the muscle strength, even in PD patients [50, 51]. In addition, previous studies showed that RT was a more effective form of exercise than other exercise types (e.g., Stretch [42], Mul_C [65]) in improving muscle strength in PD patients. Notably, TC also significantly better than many other exercise types. The tai chi protocol stresses weight shifting and ankle sway to effectively move the person’s center of gravity toward the limits of stability, alternating between a narrow stance and a wide stance to continually change the base of support, increasing support-leg standing time and trailing-leg swing time. These inherent training features have led to improved postural control and walking ability (Fig. 3B, D). These improvements indicate that TC would be effective in enhancing neuromuscular rehabilitation [66]. Therefore, these provide the possibility for TC to effectively improve muscle strength in PD patients. The results of Li, Harmer, Fitzgerald, Eckstrom, Stock, Galver, Maddalozzo, Batya [42] showed that 24 weeks TC and RT had similar effects on the improvement of lower extremity muscle strength in PD patients, and were significantly better than Stretch. In summary, these are the reason why RT and TC significantly improve muscle strength compared with many other exercise types in PD patients.

There was no doubt that exercise can effectively reduce the concern of falling of PD patients, as confirmed in previous systematic review study [67]. Our results showed that BGT_ECA, BGT, RT, VR and Mul_C significantly reduced the concern of falling compared with CON. Previous studies showed that improvements in balance [68] and muscle strength [69] had positive effects on preventing falls in older adults. These provide the basis for this study to reduce the concern of falling in PD patients with BGT_ECA, BGT, VR and Mul_C involving balance training, and RT involving strength training. However, the effect size for reducing the concern of falling in PD patients in this study were small (SMD: -0.29 to -0.38, Fig. 3K). In addition, this study used a subjective scale (e.g., falls efficacy scale) to evaluate the concern of falling, which was difficult for these scales to truly reflect the effect of preventing falls. Fewer studies have assessed the effects of exercise on reducing the number of fallers or number of falls in PD patients. More high-quality literatures are needed to verify the effect of exercise on preventing falls of PD patients in the future.

### Strengths and weaknesses of this review

Our study has strengths. First and foremost, this study is the most comprehensive and systematic comparative meta-analysis of the effects of exercises on PD patients. After rigorous and detailed searching, we identified 250 studies including 13 011 participants to collect data and investigated motor symptoms, balance, gait velocity, walking distance and freezing of gait, and non-motor symptoms included: depression, anxiety, poor sleep quality, and cognitive problems. Besides, muscle strength, and concern of falling were also the outcomes of our evaluation. This provides a better evidence-based basis for physical therapists to choose appropriate exercise types to improve the corresponding symptoms of PD patients.

However, our analysis also had limitations. In the literature exclusion criteria, we did not limit exercise dose factors such as exercise period, which resulted in large differences in exercise doses included in the literature (Additional file 1: Appendix 8 Table 8.1). The network meta-regression and sensitivity analysis showed that exercise period and exercise frequency significantly affected the results and heterogeneity of exercise in improving motor symptoms. Fortunately, the meta-regression and sensitivity analyses did not affect the overall ranking. Additionally, the results indicated that long-term and high-frequency (more than 24 weeks, more than 5 times/week) exercises were more effective in improving the PD motor symptoms (Additional file 1: Appendix 13).

Previous studies have shown that exercise intensity was also an important factor affecting exercise effects [70, 71]. Unfortunately, different exercise types set different standards for exercise intensity, and many included studies did not report exercise intensity (Additional file 1: Appendix 8 and 9). Therefore, we were unable to assess the effect of exercise intensity on the results of our study. Moreover, the downgrading of the confidence of the evidence was mostly caused by imprecision. The same exercise type with different exercise doses may be the main reason affecting the precision of the combined results. In addition, although we included 250 RCTs in network meta-analysis, some studies of the same type of exercise (PT, WBV, or Pilates) were as low as 5, which also affected the precision of the results. The 172 studies (67.8%) included in the network meta-analysis involved small samples (< 20 participants). Therefore, the lack of statistical power might have prevented the detection of between-group differences in isolated studies. According to the sensitivity analysis after excluding Chinese literature, it was found that the heterogeneity was significantly reduced (18.3%, Additional file 1: Appendix 14). At first, we decided to include the Chinese literature (10 studies) to increase the universality of the results of this study in China. However, when we collated the data and found that the SD values of the results for all indicators were smaller than those in the English literature, making the final combined SMD values larger and possibly exaggerating the effect of treatment to some extent. The above limitations led to low or very low confidence of evidence (Fig. 4A).

## Conclusions

Many exercise types had additional treatment effects in corresponding symptoms. Among them, PT showed better results in motor symptoms and muscle strength, while BWS_TT was the most effective in improving balance, gait velocity and walking distance of PD patients. In terms of non-motor symptoms, Dance and Yoga showed the best effects in alleviating depression and anxiety. RT was the only type of exercise that could improve the cognitive ability and sleep quality of PD patients. Exercises effectively reduced the concern of falling of PD patients, but the effect sizes were low. In summary, exercise have obvious effects on improving PD symptoms, but the inner molecular mechanism and the connection and regulation of neural circuits need to be further explored. In addition, the certainty of the evidence was overall low for all outcomes.

## Supplementary Information

**Additional file 1:** **Appendix 1.** PRISMA Checklist. **Appendix 2.** Protocol. **Appendix 3.** Search Strategy. **Appendix 4.** Outcomes. **Appendix 5.** Definitions of exercise types and non-exercise training control. **Appendix 6.** Statistical methods in details. **Appendix 7.** Assessment of the transitivity. **Appendix 8.** Characteristics of studies and subjects included in the review. **Appendix 9.** Risk of Bias. **Appendix 10.** Results from network meta-analyses. **Appendix 11.** Evaluation of heterogeneity and inconsistency. **Appendix 12.** Publication bias. **Appendix 13.** Network Meta-Regression. **Appendix 14.** Sensitivity analyses. **Appendix 15.** Characteristics of the sample. **Appendix 16.** Grading the evidence for outcome (motor symptoms) of the network meta-analysis using CINeMA.

## Data Availability

All data generated or analysed during this study are included in this published article, and appendix file.

## Abbreviations

RCT: Randomized controlled trials
SMD: Standardized Mean Difference
CrI: Credible Interval
AE: Aerobic Exercise
AQE: Aquatic Exercise
BGT: Balance and Gait Training
BGT_ECA: Balance and Gait Training with External Cue or Attention
BGT_ICA: Balance and Gait Training with Internal Cue or Attention
BWS_TT: Body Weight Support Treadmill Training
CON: Control group,
CPP: Classic Physiotherapy Program
DT_BGT: Dual Task Balance and Gait Training
Mul_C: Multicomponent Exercise Program
Mul_D: Multidisciplinary Exercise Program
NW: Nordic Walking
PT: Power Training
RA_GT: Robotic Assisted Gait Training
RT: Resistance Training
TC: Tai Chi
TT: Treadmill Training
VR: Virtual Reality
WBV: Whole Body Vibration
