# Supplementary material for: Efficacy and evaluation of therapeutic exercises on adults with Parkinson’s disease: a systematic review and network meta-analysis
Source: BMC Geriatr. 2022 Oct 21;22:813. doi: 10.1186/s12877-022-03510-9 (PMC9587576; doi:10.1186/s12877-022-03510-9)
Supplement: Supplementary file 1 — Additional file 1: Appendix 1. PRISMA Checklist. Appendix 2. Protocol. Appendix 3. Search Strategy. Appendix 4. Outcomes. Appendix 5. Definitions of exercise types and non-exercise training control. Appendix 6. Statistical methods in details. Appendix 7. Assessment of the transitivity. Appendix 8. Characteristics of studies and subjects included in the review. Appendix 9. Risk of Bias. Appendix 10. Results from network meta-analyses. Appendix 11. Evaluation of heterogeneity and inconsistency. Appendix 12. Publication bias. Appendix 13. Network Meta-Regression. Appendix 14. Sensitivity analyses. Appendix 15. Characteristics of the sample. Appendix 16. Grading the evidence for outcome (motor symptoms) of the network meta-analysis using CINeMA. [file 12877_2022_3510_MOESM1_ESM.docx]

**Appendix**

Table of Contents:

[Appendix 1: PRISMA Checklist 1](#_Toc113555175)

[Appendix 2: Protocol 5](#_Toc113555176)

[Appendix 3: Search Strategy 18](#_Toc113555177)

[3.1 Database: PubMed <inception to June 30, 2022> 18](#_Toc113555178)

[3.2 Database: Ovid MEDLINE(R) <1946 to June 30, 2022> 21](#_Toc113555179)

[3.3 Database: Embase <1974 to June 30, 2022> 22](#_Toc113555180)

[3.4 Database: PsycINFO <1806 to June 30, 2022 > 23](#_Toc113555181)

[3.5 Cochrane 25](#_Toc113555182)

[3.6 Database: Web of Science <1965 to June 30, 2022> 26](#_Toc113555183)

[Appendix 4: Outcomes 29](#_Toc113555184)

[Primary outcomes 29](#_Toc113555185)

[Secondary outcomes 29](#_Toc113555186)

[Appendix 5: Definitions of physical activity types and non-exercise training control 31](#_Toc113555187)

[Appendix 6: Statistical methods in details 33](#_Toc113555188)

[Network meta-analysis 33](#_Toc113555189)

[Continuous variable-random effects model: 33](#_Toc113555190)

[Network meta-regression: In the network meta-regression models we set 36](#_Toc113555191)

[Assessment of the transitivity assumption 39](#_Toc113555192)

[Assessment of heterogeneity and inconsistency 39](#_Toc113555193)

[Publication bias 40](#_Toc113555194)

[Assessment of sensitivity 40](#_Toc113555195)

[Statistical software 40](#_Toc113555196)

[Appendix 7: Assessment of the transitivity 41](#_Toc113555197)

[7.1 Publish years 41](#_Toc113555198)

[7.2 Mean age 42](#_Toc113555199)

[7.3 Years of diagnosis 43](#_Toc113555200)

[7.4 Hoehn and Yahr stage 44](#_Toc113555201)

[7.5 Percentage male 45](#_Toc113555202)

[7.6 Sample size 46](#_Toc113555203)

[Appendix 8: Characteristics of studies and subjects included in the review 47](#_Toc113555204)

[8.1 Characteristics of eligible RCTs included in network meta-analysis 47](#_Toc113555205)

[8.2 List of available outcomes for included studies 73](#_Toc113555206)

[8.3 List of included studies 100](#_Toc113555207)

[Appendix 9: Risk of Bias 117](#_Toc113555208)

[9.1 The risk of bias assessment for the individual included studies 117](#_Toc113555209)

[Appendix 10: Results from network meta-analyses 124](#_Toc113555210)

[10.1 Secondary outcomes: balance 124](#_Toc113555211)

[10.2 Secondary outcomes: Gait Velocity 127](#_Toc113555212)

[10.3 Secondary outcomes: Walking distance 130](#_Toc113555213)

[10.4 Secondary outcomes: Freezing of gait 133](#_Toc113555214)

[10.5 Secondary outcomes: depression 136](#_Toc113555215)

[10.6 Secondary outcomes: anxiety 139](#_Toc113555216)

[10.7 Secondary outcomes: sleep quality 141](#_Toc113555217)

[10.8 Secondary outcomes: cognition 143](#_Toc113555218)

[10.9 Secondary outcomes: muscle strength 146](#_Toc113555219)

[10.10 Secondary outcomes: concern of falling 149](#_Toc113555220)

[Appendix 11: Evaluation of heterogeneity and inconsistency 152](#_Toc113555221)

[11.1 Quantifying heterogeneity 152](#_Toc113555222)

[11.2 Evaluation of inconsistency 153](#_Toc113555223)

[Appendix 12: Publication bias 165](#_Toc113555224)

[Appendix 13: Network Meta-Regression 166](#_Toc113555225)

[13.1 Publication year 167](#_Toc113555226)

[13.2 Mean Age 168](#_Toc113555227)

[13.3 Years of Diagnosis 169](#_Toc113555228)

[13.4 Hoehn and Yahr stage 170](#_Toc113555229)

[13.5 Percentage Male 171](#_Toc113555230)

[13.6 Sample Size 172](#_Toc113555231)

[13.7 Exercise Period 173](#_Toc113555232)

[13.8 Exercise Frequency 174](#_Toc113555233)

[13.9 Time of single session 175](#_Toc113555234)

[13.10 ON/OFF 176](#_Toc113555235)

[Appendix 14: Sensitivity analyses 177](#_Toc113555236)

[14.1 Exclude studies at TESTEX scale <11 points 178](#_Toc113555237)

[14.2 Exclude studies with sample size of less than 20 179](#_Toc113555238)

[14.3 Exclude studies with exercise period less than 4 and more than 24 180](#_Toc113555239)

[14.4 Exclude studies with exercise frequency less than 2 and more than 4 181](#_Toc113555240)

[14.5 Exclude studies that were OFF state during testing 182](#_Toc113555241)

[14.6 Exclude Chinese literature 183](#_Toc113555242)

[14.7 Exclude studies that use GetData to extract data and estimated standard deviations value 184](#_Toc113555243)

[Appendix 15: Characteristics of the sample 185](#_Toc113555244)

[Appendix 16: Grading the evidence for primary outcome (motor symptoms) of 189](#_Toc113555245)

[16.1 Summary of study limitations of the included studies 189](#_Toc113555246)

[16.2 Contribution percentage of low, moderate, and high RoB comparisons to each network estimate 190](#_Toc113555247)

[16.3 Reasons for downgrading 199](#_Toc113555248)

[16.4 CINeMA for the primary outcome “clinical symptoms” 201](#_Toc113555249)

# Appendix 1: PRISMA Checklist

| **Section/Topic** | **Item #** | **Checklist Item** | **Reported on Page #** |
| --- | --- | --- | --- |
| **TITLE** |  |  |  |
| Title | 1 | Identify the report as a systematic review *incorporating a network meta-analysis (or related form of meta-analysis).* | 1 |
| **ABSTRACT** |  |  |  |
| Structured summary | 2 | Provide a structured summary including, as applicable:  **Background:** main objectives  **Methods:** data sources; study eligibility criteria, participants, and interventions; study appraisal; and *synthesis methods, such as network meta-analysis.*  **Results:** number of studies and participants identified; summary estimates with corresponding confidence/credible intervals; *treatment rankings may also be discussed. Authors may choose to summarize pairwise comparisons against a chosen treatment included in their analyses for brevity.*  **Discussion/Conclusions:** limitations; conclusions and implications of findings.  **Other:** primary source of funding; systematic review registration number with registry name. | 1-2 |
| **INTRODUCTION** |  |  |  |
| Rationale | 3 | Describe the rationale for the review in the context of what is already known*, including mention of why a network meta-analysis has been conducted.* | 4 |
| Objectives | 4 | Provide an explicit statement of questions being addressed, with reference to participants, interventions, comparisons, outcomes, and study design (PICOS). | 4 |
|  |  |  |  |
| **METHODS** |  |  |  |
| Protocol and registration | 5 | Indicate whether a review protocol exists and if and where it can be accessed (e.g., Web address); and, if available, provide registration information, including registration number. | 2 PROSPERO (CRD420212220052) |
| Eligibility criteria | 6 | Specify study characteristics (e.g., PICOS, length of follow-up) and report characteristics (e.g., years considered, language, publication status) used as criteria for eligibility, giving rationale. *Clearly describe eligible treatments included in the treatment network, and note whether any have been clustered or merged into the same node (with justification).* | 5 |
| Information sources | 7 | Describe all information sources (e.g., databases with dates of coverage, contact with study authors to identify additional studies) in the search and date last searched. | 5 |
| Search | 8 | Present full electronic search strategy for at least one database, including any limits used, such that it could be repeated. | Appendix 3 |
| Study selection | 9 | State the process for selecting studies (i.e., screening, eligibility, included in systematic review, and, if applicable, included in the meta-analysis). | 5, and Appendix 3 |
| Data collection process | 10 | Describe method of data extraction from reports (e.g., piloted forms, independently, in duplicate) and any processes for obtaining and confirming data from investigators. | 7 |
| Data items | 11 | List and define all variables for which data were sought (e.g., PICOS, funding sources) and any assumptions and simplifications made. | Appendix 2 |
| **Geometry of the network** | **S1** | Describe methods used to explore the geometry of the treatment network under study and potential biases related to it. This should include how the evidence base has been graphically summarized for presentation, and what characteristics were compiled and used to describe the evidence base to readers. | Appendix 6 |
| Risk of bias within individual studies | 12 | Describe methods used for assessing risk of bias of individual studies (including specification of whether this was done at the study or outcome level), and how this information is to be used in any data synthesis. | 6, and Appendix 6 |
| Summary measures | 13 | State the principal summary measures (e.g., risk ratio, difference in means). *Also describe the use of additional summary measures assessed, such as treatment rankings and surface under the cumulative ranking curve (SUCRA) values, as well as modified approaches used to present summary findings from meta-analyses.* | Appendix 6 |
| Planned methods of analysis | 14 | Describe the methods of handling data and combining results of studies for each network meta-analysis. This should include, but not be limited to:   - *Handling of multi-arm trials;* - *Selection of variance structure;* - *Selection of prior distributions in Bayesian analyses; and* - *Assessment of model fit.* | Appendix 6 |
| **Assessment of Inconsistency** | **S2** | Describe the statistical methods used to evaluate the agreement of direct and indirect evidence in the treatment network(s) studied. Describe efforts taken to address its presence when found. | Appendix 6 and 12 |
| Risk of bias across studies | 15 | Specify any assessment of risk of bias that may affect the cumulative evidence (e.g., publication bias, selective reporting within studies). | Appendix 16 |
| Additional analyses | 16 | Describe methods of additional analyses if done, indicating which were pre-specified. This may include, but not be limited to, the following:   - Sensitivity or subgroup analyses; - Meta-regression analyses; - *Alternative formulations of the treatment network; and* - *Use of alternative prior distributions for Bayesian analyses (if applicable).* | Appendix 13, 14 |
| **RESULTS†** |  |  |  |
| Study selection | 17 | Give numbers of studies screened, assessed for eligibility, and included in the review, with reasons for exclusions at each stage, ideally with a flow diagram. | 7, Figure 1 |
| **Presentation of network structure** | **S3** | Provide a network graph of the included studies to enable visualization of the geometry of the treatment network. | Figure 2, Appendix 10 |
| **Summary of network geometry** | **S4** | Provide a brief overview of characteristics of the treatment network. This may include commentary on the abundance of trials and randomized patients for the different interventions and pairwise comparisons in the network, gaps of evidence in the treatment network, and potential biases reflected by the network structure. | Appendix 10 |
| Study characteristics | 18 | For each study, present characteristics for which data were extracted (e.g., study size, PICOS, follow-up period) and provide the citations. | Table 1, Appendix 8 |
| Risk of bias within studies | 19 | Present data on risk of bias of each study and, if available, any outcome level assessment. | Appendix 9 |
| Results of individual studies | 20 | For all outcomes considered (benefits or harms), present, for each study: 1) simple summary data for each intervention group, and 2) effect estimates and confidence intervals. *Modified approaches may be needed to deal with information from larger networks.* | Figure 2 and 3, Table 2 |
| Synthesis of results | 21 | Present results of each meta-analysis done, including confidence/credible intervals. *In larger networks, authors may focus on comparisons versus a particular comparator (e.g. placebo or standard care), with full findings presented in an appendix. League tables and forest plots may be considered to summarize pairwise comparisons.* If additional summary measures were explored (such as treatment rankings), these should also be presented. | Figure 2, 3 and 4, Table 2, and Appendix 10 |
| **Exploration for inconsistency** | **S5** | Describe results from investigations of inconsistency. This may include such information as measures of model fit to compare consistency and inconsistency models, *P* values from statistical tests, or summary of inconsistency estimates from different parts of the treatment network. | Appendix 11 |
| Risk of bias across studies | 22 | Present results of any assessment of risk of bias across studies for the evidence base being studied. | 7, Appendix 9 |
| Results of additional analyses | 23 | Give results of additional analyses, if done (e.g., sensitivity or subgroup analyses, meta-regression analyses*, alternative network geometries studied, alternative choice of prior distributions for Bayesian analyses,* and so forth). | 9, Appendix 13, 14 |
| **DISCUSSION** |  |  |  |
| Summary of evidence | 24 | Summarize the main findings, including the strength of evidence for each main outcome; consider their relevance to key groups (e.g., healthcare providers, users, and policy-makers). | 10 |
| Limitations | 25 | Discuss limitations at study and outcome level (e.g., risk of bias), and at review level (e.g., incomplete retrieval of identified research, reporting bias). *Comment on the validity of the assumptions, such as transitivity and consistency. Comment on any concerns regarding network geometry (e.g., avoidance of certain comparisons).* | 12-13 |
| Conclusions | 26 | Provide a general interpretation of the results in the context of other evidence, and implications for future research. | 14 |
| **FUNDING** |  |  |  |
| Funding | 27 | Describe sources of funding for the systematic review and other support (e.g., supply of data); role of funders for the systematic review. This should also include information regarding whether funding has been received from manufacturers of treatments in the network and/or whether some of the authors are content experts with professional conflicts of interest that could affect use of treatments in the network. | 15 |

PICOS = population, intervention, comparators, outcomes, study design.

# Appendix 2: Protocol

Review title and timescale

1. Review title.

Give the title of the review in English

Effects of exercise in Parkinson’s disease: systematic review and network meta-analysis

2. Original language title.

For reviews in languages other than English, give the title in the original language. This will be displayed with the English language title.

3. Anticipated or actual start date.

Give the date the systematic review started or is expected to start.

30/11/2020

4. Anticipated completion date.

Give the date by which the review is expected to be completed.

31/05/2022

5. Stage of review at time of this submission.

Tick the boxes to show which review tasks have been started and which have been completed. Update this field each time any amendments are made to a published record.

Reviews that have started data extraction (at the time of initial submission) are not eligible for inclusion in PROSPERO. If there is later evidence that incorrect status and/or completion date has been supplied, the published PROSPERO record will be marked as retracted.

This field uses answers to initial screening questions. It cannot be edited until after registration.

The review has not yet started:

| Review stage | Started | Completed |
| --- | --- | --- |
| Preliminary searches | Yes | No |
| Piloting of the study selection process | Yes | No |
| Formal screening of search results against eligibility criteria | No | No |
| Data extraction | No | No |
| Risk of bias (quality) assessment | No | No |
| Data analysis | No | No |

Provide any other relevant information about the stage of the review here.

6. Named contact.

The named contact is the guarantor for the accuracy of the information in the register record. This may be any member of the review team.

Yong Yang

Email salutation (e.g. "Dr Smith" or "Joanne") for correspondence:

Dr Yang

7. Named contact email.

Give the electronic email address of the named contact.

yangyong@henu.edu.cn

8. Named contact address

Give the full institutional/organisational postal address for the named contact.

Minglun Street, Shunhe Hui District, Kaifeng City, Henan Province, China

9. Named contact phone number.

Give the telephone number for the named contact, including international dialling code.

13581796876

10. Organisational affiliation of the review.

Full title of the organisational affiliations for this review and website address if available. This field may be completed as 'None' if the review is not affiliated to any organisation.

Institute of Physical Education, Henan University, Kaifeng475001, China

Organisation web address:
11. Review team members and their organisational affiliations.

Give the personal details and the organisational affiliations of each member of the review team. Affiliation refers to groups or organisations to which review team members belong. **NOTE: email and country now MUST be entered for each person, unless you are amending a published record.**

Dr Yong Yang. Institute of Physical Education, Henan University, Kaifeng 475001, China
Professor Jianshe Wei. Institute of Neurological Disorder, Henan University, Kaifeng 475000, China Dr Wensheng Zhou. Nanjing Xiao-Zhuang University, Nanjing 211171, China;
Dr Guotuan Wang. Institute of Physical Education, Henan University, Kaifeng475001, China

12. Funding sources/sponsors.

Details of the individuals, organizations, groups, companies or other legal entities who have funded or sponsored the review.

None

13. Conflicts of interest.

List actual or perceived conflicts of interest (financial or academic).

None

14. Collaborators.

Give the name and affiliation of any individuals or organisations who are working on the review but who are not listed as review team members. NOTE: email and country must be completed for each person, unless you are amending a published record.

15. Review question.

State the review question(s) clearly and precisely. It may be appropriate to break very broad questions down into a series of related more specific questions. Questions may be framed or refined using PI(E)COS or similar where relevant.

1) Are exercise interventions more effective than non-exercise interventions for quality of life, improving strength, endurance and function, improving balance ability and reducing rate of fall in patients with Parkinson's disease?
2) Are there specific physical activities or exercise interventions that are more effective than others for quality of life, improving strength, endurance and function, improving balance ability and reducing rate of fall in patients with Parkinson's disease?

16. Searches.

State the sources that will be searched (e.g. Medline). Give the search dates, and any restrictions (e.g. language or publication date). Do NOT enter the full search strategy (it may be provided as a link or attachment below.)

The systematic review and meta-analysis were conducted in accordance with the Preferred Reporting Items for Systematic Reviews and Meta-Analyses (PRISMA) guidelines. We will search PubMed, EMBASE, PsycINFO, The Cochrane Library (Cochrane Database of Systematic Reviews, Cochrane Central Register of Controlled Trials (CENTRAL), Cochrane Methodology Register), and Web of Science.

There will be no language restrictions. Studies published between inception and the date the searches are run will be sought. The searches will be re-run just before the final analyses and further studies retrieved for inclusion. We will use a Boolean search strategy with the operators AND, OR, NOT, and the search strategy will include terms describing or relating to intervention, participants, and study design.

17. URL to search strategy.

Upload a file with your search strategy, or an example of a search strategy for a specific database, (including the keywords) in pdf or word format. In doing so you are consenting to the file being made publicly accessible. Or provide a URL or link to the strategy. Do NOT provide links to your search results.

I give permission for this file to be made publicly available

Yes

18. Condition or domain being studied.

Give a short description of the disease, condition or healthcare domain being studied in your systematic review.

Parkinson's disease and exercise

19. Participants/population.

Specify the participants or populations being studied in the review. The preferred format includes details of both inclusion and exclusion criteria.

Age: 'Middle-aged and elderly' ('45+') . Sex: Males and females

Race: Any

Diagnosed Parkinson's disease

Years of diagnosis: 10-

Hoehn and Yahr scale (H&Y): 3-

20. Intervention(s), exposure(s).

Give full and clear descriptions or definitions of the interventions or the exposures to be reviewed. The preferred format includes details of both inclusion and exclusion criteria.

The intervention to be reviewed is: I. Exercise based intervention and II. Types of exercise intervention in multi-treatment comparison, including
1) Resistance exercise (based on ACSM definition, Garber (2011) Med Sci Sports Exerc 43: 1334-1335);

2) balance training (e.g. video game balance; Virtual reality training; Dual Task balance Training et al.)

3) Pilates;

4) Yoga;

5) mind body exercise;

6) Flexion exercise;

7) Aerobic exercise (walking, cycling, jogging in any land-based mode);

8) Water based exercise;

9) Stretching;

10) Tai Chi;

11) Qigong

12) Physical therapy with exercise;

13) Multi-modal exercise (i.e. any combination of physical activity types 1-9).

21. Comparator(s)/control.

Where relevant, give details of the alternatives against which the intervention/exposure will be compared (e.g. another intervention or a non-exposed control group). The preferred format includes details of both inclusion and exclusion criteria.

1. ‘Usual care’ control was determined based on the report. In ‘usual care’, participants were expected to continue the routine standard of care provided by their general practitioners.

2. Control groups that were not given any specific intervention such as ‘waiting list’ or usual physical activity or where the authors did not specify the nature of the control were also classified as ‘usual care’. ‘Waiting-list’ controls were given active intervention after a period of observation, with no new intervention being delivered during the trial period.

22. Types of study to be included.

Give details of the study designs (e.g. RCT) that are eligible for inclusion in the review. The preferred format includes both inclusion and exclusion criteria. If there are no restrictions on the types of study, this should be stated.

Include only randomized controlled trials

23. Context.

Give summary details of the setting or other relevant characteristics, which help define the inclusion or exclusion criteria.

24. Main outcome(s).

Give the pre-specified main (most important) outcomes of the review, including details of how the outcome is

1. quality of life (e.g. Parkinson’s Disease Quality of life scale-39)

2. UPDRS: Unified Parkinson’s Disease Rating Scale (TOT = total score; II = part II: ADL score; III = part III: motor score)

3. freezing of gait (the freezing of Gait Questionnaire [FOG-Q])

Measures of effect

Please specify the effect measure(s) for you main outcome(s) e.g. relative risks, odds ratios, risk difference, and/or 'number needed to treat.

Timing: The specified end point of the trial will be used as the end-point in the assessment.

Effect measure: Standardised mean difference at the end of the study

25. Additional outcome(s).

List the pre-specified additional outcomes of the review, with a similar level of detail to that required for main outcomes. Where there are no additional outcomes please state ‘None’ or ‘Not applicable’ as appropriate to the review

1. rate of fall, rate of faller

2. function performance

Measures of effect

Please specify the effect measure(s) for you additional outcome(s) e.g. relative risks, odds ratios, risk difference, and/or 'number needed to treat.

1. rate of fall, rate of faller

2. function performance: muscle strength; endurance; balance((a) static steady-state balance (e.g., CoP displacements during single leg stance), (b) dynamic steady-state balance (e.g., 10-m gait speed test), (c) proactive balance (e.g., Functional-Reach-Test or TUG), (d) reactive balance (e.g., CoP displacements after an unexpected perturbation), and (e) balance test batteries (e.g., Berg Balance Scale))

26. Data extraction (selection and coding).

Describe how studies will be selected for inclusion. State what data will be extracted or obtained. State how this will be done and recorded.

All search results will be exported into EndNote and duplicates will be removed. Titles and abstracts from the initial literature search will be independently assessed by two reviewers (Y.Y. and W.S.Z.). Full texts for articles deemed eligible for inclusion from the title and abstract search by either reviewer, in addition to those where no decision could be reached by the reviewers from this initial screen, will be screened independently by two researchers (Y.Y. and W.S.Z.). Any discrepancies will be resolved by discussion with all researchers in the review team. Two reviewers (Y.Y. and W.S.Z.) will independently extract data from the final inclusion list of articles into a standardised data extraction spreadsheet in Excel. At this stage, two authors extracted information on (1) relevant data regarding participant characteristics (e.g., the sample size, age, and sex); (2) training pattern; (3) training variable (e.g., duration, frequency, sets, repetitions, and intensity); (4) Years of diagnosis; (5) Hoehn and Yahr scale (H&Y) and (6) the main result of the study. In case of incomplete raw data availability, we contacted the corresponding author of the manuscript. We excluded the studies of which the authors could not be reached. All studies were assessed independently in this systematic review by two researchers (Y.Y. and W.S.Z.) based on the extracted information. If there were any disagreements about the inclusion of a study, a third reviewer (J.S.W.) was consulted.

27. Risk of bias (quality) assessment.

State which characteristics of the studies will be assessed and/or any formal risk of bias/quality assessment tools that will be used.

The study quality was assessed with the PEDro scale, based on the list of Delphi (Verhagen et al., 1998). The PEDro scale includes 11 items with three items from the Jadad scale (Jadad et al., 1996) and nine items from the Delphi list (Verhagen et al., 1998). PEDro rates RCTs on a scale from 0 (low quality) to 10 (high quality), and scores less than 6 are considered to have low methodological quality, as per the PEDro database statistics (Maher, Sherrington, Herbert, Moseley, & Elkins, 2003). Interrater reliability was shown to be fair to good (Intraclass Correlation Coefficient = 0.68). Two reviewers (Y.Y. and W.S.Z.) scored the studies according to the proposed scale. In case of disagreements, a consensus was adopted or, if necessary, a third reviewer evaluated the article (J.S.W.).

28. Strategy for data synthesis.

Describe the methods you plan to use to synthesise data. This must not be generic text but should be specific to your review and describe how the proposed approach will be applied to your data.
If meta-analysis is planned, describe the models to be used, methods to explore statistical heterogeneity, and software package to be used.

Aggregated data will be used and a narrative synthesis will be presented. In addition, a quantitative synthesis is planned. A pairwise meta-analysis is planned to compare exercise-based interventions to non-exercise based interventions (Part I). Random-effects meta-analysis will be used as it is anticipated that there will be heterogeneity in the populations considered in the included studies. Heterogeneity will be assessed using the χ² test and I² statistic. Forest plots will be used to graphically depict the individual and pooled effect sizes and funnel plots will be used to assess potential publication bias. Network meta-analysis is planned for the multiple treatment comparison (Part II). Heterogeneity will be assessed, in addition to consistency which will be examined by fitting consistency and inconsistency models. This will determine if the treatment effect for a pair of treatments estimated from an indirect comparison is consistent with the treatment effect estimated from a direct comparison.

29. Analysis of subgroups or subsets.

State any planned investigation of ‘subgroups’. Be clear and specific about which type of study or participant will be included in each group or covariate investigated. State the planned analytic approach.

Potential sub-group analyses pending I² statistic (heterogeneity) and number of studies available: e.g. medication use, severity of pain, Years of diagnosis, Disease grade: Hoehn and Yahr scale (H&Y), age and gender. In addition, a sensitivity analysis will be conducted which will exclude studies deemed to be high risk of bias.

30. Type and method of review.

Select the type of review, review method and health area from the lists below.

Network meta-analysis

31. Language.

Select each language individually to add it to the list below, use the bin icon to remove any added in error.

English

32. Country.

Select the country in which the review is being carried out. For multi-national collaborations select all the countries involved.

China

33. Other registration details.

Name any other organisation where the systematic review title or protocol is registered (e.g. Campbell, or The Joanna Briggs Institute) together with any unique identification number assigned by them. If extracted data will be stored and made available through a repository such as the Systematic Review Data Repository (SRDR), details and a link should be included here. If none, leave blank.

34. Reference and/or URL for published protocol.

If the protocol for this review is published provide details (authors, title and journal details, preferably in Vancouver format)

I give permission for this file to be made publicly available

Yes

35  Dissemination plans

Give brief details of plans for communicating essential messages from the review to the appropriate audiences.

Do you intend to publish the review on completion?

Yes

36  Keywords
Give words or phrases that best describe the review. (One word per box, create a new box for each term)

37. Details of any existing review of the same topic by the same authors.

If you are registering an update of an existing review give details of the earlier versions and include a full bibliographic reference, if available.

38. Current review status.

Update review status when the review is completed and when it is published.
New registrations must be ongoing so this field is not editable for initial submission.

Ongoing

39. Any additional information.

Provide any other information relevant to the registration of this review.

This study is only part of a randomized controlled experiment. Through this study, we want to find the best physical activity type to improve the ability of daily living of Parkinson's patients, and use this as the experimental group of our randomized controlled trial, and compare different exercise doses to Parkinson's patients the impact of daily living.

40. Details of final report/publication(s) or preprints if available.

Leave empty until publication details are available OR you have a link to a preprint (NOTE: this field is not editable for initial submission).
List authors, title and journal details preferably in Vancouver format.

Give the link to the published review or preprint.

# Appendix 3: Search Strategy

## 3.1 Database: PubMed <inception to June 30, 2022>

***Search Strategy:***

| #25 | Search: ((Parkinson disease[MeSH Terms]) AND ((((((((((exercise*[MeSH Terms]) OR (resistance training[MeSH Terms])) OR (Tai Ji[MeSH Terms])) OR (Qigong[MeSH Terms])) OR (Exercise Movement Techniques[MeSH Terms])) OR (Yoga[MeSH Terms])) OR (Virtual Reality[MeSH Terms])) OR (hydrotherapy[MeSH Terms])) OR (Dance Therapy[MeSH Terms])) OR ("aerobic exercise" or "aquatic exercise" or "balance training" or "body weight support treadmill" or "gait training" or "high-speed resistance training" or "multicomponent exercise program" or "multidisciplinary exercise program" or "Nordic Walking" or Physiotherapy or pilates or "power training" or "Robotic-assisted gait training" or stretch or Tango or "treadmill training" or "walking" or "whole body vibration"))) AND ((((((((randomized controlled trial[Publication Type]) OR (controlled clinical trial[Publication Type])) OR (randomized[Title/Abstract])) OR (placebo[Title/Abstract])) OR (randomly[Title/Abstract])) OR (trial[Title])) OR (clinical trials as topic[MeSH Terms])) NOT ((animals[MeSH Terms]) NOT (humans[MeSH Terms]))) | 890 |
| --- | --- | --- |
| #24 | Search: (((((((randomized controlled trial[Publication Type]) OR (controlled clinical trial[Publication Type])) OR (randomized[Title/Abstract])) OR (placebo[Title/Abstract])) OR (randomly[Title/Abstract])) OR (trial[Title])) OR (clinical trials as topic[MeSH Terms])) NOT ((animals[MeSH Terms]) NOT (humans[MeSH Terms])) | 1,341,164 |
| #23 | Search: ((((((randomized controlled trial[Publication Type]) OR (controlled clinical trial[Publication Type])) OR (randomized[Title/Abstract])) OR (placebo[Title/Abstract])) OR (randomly[Title/Abstract])) OR (trial[Title])) OR (clinical trials as topic[MeSH Terms]) | 1,449,600 |
| #22 | Search: (((((((((exercise*[MeSH Terms]) OR (resistance training[MeSH Terms])) OR (Tai Ji[MeSH Terms])) OR (Qigong[MeSH Terms])) OR (Exercise Movement Techniques[MeSH Terms])) OR (Yoga[MeSH Terms])) OR (Virtual Reality[MeSH Terms])) OR (hydrotherapy[MeSH Terms])) OR (Dance Therapy[MeSH Terms])) OR ("aerobic exercise" or "aquatic exercise" or "balance training" or "body weight support treadmill" or "gait training" or "high-speed resistance training" or "multicomponent exercise program" or "multidisciplinary exercise program" or "Nordic Walking" or Physiotherapy or pilates or "power training" or "Robotic-assisted gait training" or stretch or Tango or "treadmill training" or "walking" or "whole body vibration") | 551,542 |
| #21 | Search: (animals[MeSH Terms]) NOT (humans[MeSH Terms]) | 4,815,925 |
| #20 | Search: humans[MeSH Terms] | 19,183,084 |
| #19 | Search: animals[MeSH Terms] | 23,999,009 |
| #18 | Search: clinical trials as topic[MeSH Terms] | 355,600 |
| #17 | Search: trial[Title] | 238,308 |
| #16 | Search: randomly[Title/Abstract] | 356,459 |
| #15 | Search: placebo[Title/Abstract] | 223,336 |
| #14 | Search: randomized[Title/Abstract] | 561,707 |
| #13 | Search: controlled clinical trial[Publication Type] | 617,986 |
| #12 | Search: randomized controlled trial[Publication Type] | 528,725 |
| #11 | Search: "aerobic exercise" or "aquatic exercise" or "balance training" or "body weight support treadmill" or "gait training" or "high-speed resistance training" or "multicomponent exercise program" or "multidisciplinary exercise program" or "Nordic Walking" or Physiotherapy or pilates or "power training" or "Robotic-assisted gait training" or stretch or Tango or "treadmill training" or "walking" or "whole body vibration" | 361,398 |
| #10 | Search: Dance Therapy[MeSH Terms] | 396 |
| #9 | Search: hydrotherapy[MeSH Terms] | 20,257 |
| #8 | Search: Virtual Reality[MeSH Terms] | 2,684 |
| #7 | Search: Yoga[MeSH Terms] | 3,002 |
| #6 | Search: Exercise Movement Techniques[MeSH Terms] | 8,700 |
| #5 | Search: Qigong[MeSH Terms] | 229 |
| #4 | Search: Tai Ji[MeSH Terms] | 1,183 |
| #3 | Search: resistance training[MeSH Terms] | 9,538 |
| #2 | Search: exercise*[MeSH Terms] | 297,336 |
| #1 | Search: Parkinson disease[MeSH Terms] | 69,308 |

## 3.2 Database: Ovid MEDLINE(R) <1946 to June 30, 2022>

***Search Strategy: --------------------------------------------------------------------------------***

1 Parkinson$.mp. (136173)

2 exp Parkinson disease/ (69312)

3 (aerobic exercise or aquatic exercise or balance training or body weight support treadmill or Dance Therapy or exercise$ or Exercise Movement Techniques or gait training or high-speed resistance training or hydrotherapy or multicomponent exercise program or multidisciplinary exercise program or Nordic Walking or Physiotherapy or pilates or power training or Qigong or resistance training or Robotic-assisted gait training or stretch or tai ji or Tango or treadmill training or walking or Virtual Reality or whole body vibration or Yoga).mp. (540012)

4 exp resistance training/ (9532)

5 exp exercise$/ (206975)

6 exp tai ji/ (1182)

7 exp Qigong/ (228)

8 exp Exercise Movement Techniques/ (8695)

9 exp Yoga/ (2999)

10 exp Virtual Reality/ (2682)

11 exp hydrotherapy/ (20254)

12 exp Dance Therapy/ (396)

13 randomized controlled trial.pt. (527440)

14 controlled clinical trial.pt. (94123)

15 randomized.ab. (517037)

16 clinical trials as topic.sh. (195553)

17 randomly.ab. (355668)

18 trial.ti. (238446)

19 exp clinical trial/ (888782)

20 exp randomized controlled trials/ (145969)

21 exp cross-over studies/ (49955)

22 (clinic$ adj2 trial).mp. (746815)

23 (random$ adj5 control$ adj5 trial$).mp. (770827)

24 (crossover or cross-over).mp. (100433)

25 randomi$.mp. (943880)

26 (random$ adj5 (assign$ or allocat$ or assort$ or reciev$)).mp. (256419)

27 1 or 2 (136173)

28 3 or 4 or 5 or 6 or 7 or 8 or 9 or 10 or 11 or 12 (586531)

29 13 or 14 or 15 or 16 or 17 or 18 or 19 or 20 or 21 or 22 or 23 or 24 or 25 or 26 (1770322)

30 27 and 28 and 29 (1246)

3.3 Database: Embase <1974 to June 30, 2022>
***Search Strategy:***

--------------------------------------------------------------------------------

1 Parkinson$.mp. (219918)

2 exp Parkinson disease/ (163492)

3 (aerobic exercise or aquatic exercise or balance training or body weight support treadmill or Dance Therapy or exercise$ or Exercise Movement Techniques or gait training or high-speed resistance training or hydrotherapy or multicomponent exercise program or multidisciplinary exercise program or Nordic Walking or Physiotherapy or pilates or power training or Qigong or resistance training or Robotic-assisted gait training or stretch or tai ji or Tango or treadmill training or walking or Virtual Reality or whole body vibration or Yoga).mp. (816955)

4 exp resistance training/ (20137)

5 exp exercise$/ (363106)

6 exp tai ji/ (3173)

7 exp Qigong/ (836)

8 exp Exercise Movement Technique/ (82933)

9 exp Yoga/ (8492)

10 exp Virtual Reality/ (18896)

11 exp hydrotherapy/ (3829)

12 exp Dance Therapy/ (527)

13 randomized.ab. (757440)

14 randomly.ab. (481077)

15 trial.ti. (332106)

16 exp clinical trial/ (1627821)

17 exp randomized controlled trials/ (200725)

18 exp cross-over studies/ (66963)

19 (clinic$ adj2 trial).mp. (1639410)

20 (random$ adj5 control$ adj5 trial$).mp. (961796)

21 (crossover or cross-over).mp. (123532)

22 randomi$.mp. (1323771)

23 (random$ adj5 (assign$ or allocat$ or assort$ or reciev$)).mp. (209949)

24 1 or 2 (219918)

25 3 or 4 or 5 or 6 or 7 or 8 or 9 or 10 or 11 or 12 (841979)

26 13 or 14 or 15 or 16 or 17 or 18 or 19 or 20 or 21 or 22 or 23 (2779253)

27 24 and 25 and 26 (2562)

## 3.4 Database: PsycINFO <1806 to June 30, 2022 >

***Search Strategy:***

| Set No. Searched for Databases Results | | | |
| --- | --- | --- | --- |
| S1 | Parkinson* | APA PsycInfo® | 39453 |
| S2 | Mainsubject(Parkinson disease) | APA PsycInfo® | 25842 |
| S3 | su((aerobic exercise or aquatic exercise or balance training or body weight support treadmill or Dance Therapy or exercise$ or Exercise Movement Techniques or gait training or high-speed resistance training or hydrotherapy or multicomponent exercise program or multidisciplinary exercise program or Nordic Walking or Physiotherapy or pilates or power training or Qigong or resistance training or Robotic-assisted gait training or stretch or tai ji or Tango or treadmill training or walking or Virtual Reality or whole body vibration or Yoga)) | APA PsycInfo® | 63328 |
| S4 | su(exercise$) | APA PsycInfo® | 39377 |
| S6 | su(physical activity) | APA PsycInfo® | 39490 |
| S7 | ab(randomized) | APA PsycInfo® | 83500 |
| S8 | ab(randomly) | APA PsycInfo® | 75844 |
| S9 | ti(trial) | APA PsycInfo® | 41193 |
| S10 | ab(clinical trial) | APA PsycInfo® | 51602 |
| S11 | ab(randomized controlled trials) | APA PsycInfo® | 36991 |
| S12 | ab(cross-over studies) | APA PsycInfo® | 2076 |
| S13 | ab(crossover studies) | APA PsycInfo® | 5363 |
| S14 | ab(randomi*) | APA PsycInfo® | 83917 |
| S15 | su(animals) | APA PsycInfo® | 459210 |
| S16 | S1 OR S2 | APA PsycInfo® These databases are searched for part of your query. | 39453 |
| S17 | S3 OR S4 OR "S5" | APA PsycInfo® These databases are searched for part of your query. | 63473 |
| S18 | S6 OR S7 OR "S8" OR "S9" OR "S10" OR "S11" OR "S12" OR "S13" OR "S14" | APA PsycInfo® These databases are searched for part of your query. | 120428 |
| S19 | S16 AND S17 | APA PsycInfo® These databases are searched for part of your query. | 1074 |
| S20 | S18 AND S19 | APA PsycInfo® These databases are searched for part of your query. | 277 |
| S21 | S20 NOT S15 | APA PsycInfo® These databases are searched for part of your query. | 251 |

## 3.5 Cochrane

#1  MeSH descriptor: [Parkinson disease] explode all trees (4376)

#2 (aerobic exercise or aquatic exercise or balance training or body weight support treadmill or Dance Therapy or exercise* or Exercise Movement Techniques or gait training or high-speed resistance training or hydrotherapy or multicomponent exercise program or multidisciplinary exercise program or Nordic Walking or Physiotherapy or pilates or power training or Qigong or resistance training or Robotic-assisted gait training or stretch or tai ji or Tango or treadmill training or walking or Virtual Reality or whole body vibration or Yoga) in Trials (Word variations have been searched) (155706)

#3 MeSH descriptor: [resistance training] explode all trees (3641)

#4 MeSH descriptor: [exercise] explode all trees (25628)

#5 MeSH descriptor: [tai ji] explode all trees (373)

#6 MeSH descriptor: [Qigong] explode all trees (79)

#7 MeSH descriptor: [Exercise Movement Technique] explode all trees (2215)

#8 MeSH descriptor: [Yoga] explode all trees (699)

#9 MeSH descriptor: [Virtual Reality] explode all trees (284)

#10 MeSH descriptor: [hydrotherapy] explode all trees (1575)

#11 MeSH descriptor: [Dance Therapy] explode all trees (89)

#12 #2 or #3 or #4 or #5 or #6 or #7 or #8 or #9 or #10 or #11 (147882)

#13 #1 and #12 (906)

## 3.6 Database: Web of Science <1965 to June 30, 2022>

| # 13 | 2,403 | #12 AND #11 AND #1  Indexes=SCI-EXPANDED, SSCI, A&HCI, CPCI-S, CPCI-SSH, BKCI-S, BKCI-SSH, ESCI, CCR-EXPANDED, IC Timespan=All years |  |  |
| --- | --- | --- | --- | --- |
| # 12 | 981,618 | #10 OR #9 OR #8 OR #7 OR #6 OR #5 OR #4 OR #3 OR #2  Indexes=SCI-EXPANDED, SSCI, A&HCI, CPCI-S, CPCI-SSH, BKCI-S, BKCI-SSH, ESCI, CCR-EXPANDED, IC Timespan=All years |  |  |
| # 11 | 6,290,817 | TOPIC: ((“randomized controlled trial*” or “controlled clinical trial” or “random*” or “clinical trial*” or randomly or trial or “clinical trial” or “randomized controlled trial*” or “cross-over studies” or clinic*) )  Indexes=SCI-EXPANDED, SSCI, A&HCI, CPCI-S, CPCI-SSH, BKCI-S, BKCI-SSH, ESCI, CCR-EXPANDED, IC Timespan=All years |  |  |
| # 10 | 7,671 | TOPIC: ((Yoga or “Muscle Stretching Exercises”) )  Indexes=SCI-EXPANDED, SSCI, A&HCI, CPCI-S, CPCI-SSH, BKCI-S, BKCI-SSH, ESCI, CCR-EXPANDED, IC Timespan=All years |  |  |
| # 9 | 373 | TOPIC: ((“Dance Therapy” or “Therapy, Dance” or “Dance Therapies” or “Therapies, Dance”) )  Indexes=SCI-EXPANDED, SSCI, A&HCI, CPCI-S, CPCI-SSH, BKCI-S, BKCI-SSH, ESCI, CCR-EXPANDED, IC Timespan=All years |  |  |
| # 8 | 1,197 | TOPIC: ((hydrotherapy or Hydrotherapies or “Whirlpool Baths” or “Bath, Whirlpool” or “Baths, Whirlpool” or “Whirlpool Bath”) )  Indexes=SCI-EXPANDED, SSCI, A&HCI, CPCI-S, CPCI-SSH, BKCI-S, BKCI-SSH, ESCI, CCR-EXPANDED, IC Timespan=All years |  |  |
| # 7 | 46,480 | TOPIC: (("Virtual Reality" or "Reality, Virtual" or "Virtual Reality, Educational" or "Educational Virtual Realities" or "Educational Virtual Reality" or "Reality, Educational Virtual" or "Virtual Realities, Educational" or "Virtual Reality, Instructional" or "Instructional Virtual Realities" or "Instructional Virtual Reality" or "Realities, Instructional Virtual" or "Reality, Instructional Virtual" or "Virtual Realities, Instructional") )  Indexes=SCI-EXPANDED, SSCI, A&HCI, CPCI-S, CPCI-SSH, BKCI-S, BKCI-SSH, ESCI, CCR-EXPANDED, IC Timespan=All years |  |  |
| # 6 | 234 | TOPIC: (("Exercise Movement Techniques" or "Movement Techniques, Exercise" or "Exercise Movement Technics" or "Pilates-Based Exercises" or "Exercises, Pilates-Based" or "Pilates Based Exercises" or "Pilates Training" or "Training, Pilates"）)  Indexes=SCI-EXPANDED, SSCI, A&HCI, CPCI-S, CPCI-SSH, BKCI-S, BKCI-SSH, ESCI, CCR-EXPANDED, IC Timespan=All years |  |  |
| # 5 | 4,058 | TOPIC: (“Tai-ji” or “Tai Chi” or “Chi, Tai” or “Tai Ji Quan” or “Ji Quan, Tai” or “Quan, Tai Ji” or Taiji or Taijiquan or “T'ai Chi” or “Tai Chi Chuan” Qigong or “Qi Gong” or “Ch'i Kung”)  Indexes=SCI-EXPANDED, SSCI, A&HCI, CPCI-S, CPCI-SSH, BKCI-S, BKCI-SSH, ESCI, CCR-EXPANDED, IC Timespan=All years |  |  |
| # 4 | 625,989 | TOPIC: (Exercise* or “Exercise Program, Weight-Bearing” or “Exercise Programs, Weight-Bearing” or “Weight Bearing Exercise Program” or “Weight-Bearing Exercise Programs” Exercise* or “Physical Activity” or “Activities, Physical” or “Activity, Physical” or “Physical Activities” or “Exercise, Physical” or “Exercises, Physical” or “Physical Exercise” or “Physical Exercises” or “Exercise, Isometric” or “Exercises, Isometric” or “Isometric Exercises” or “Isometric Exercise” or “Exercise, Aerobic” or “Aerobic Exercise” or “Aerobic Exercises” or “Exercises, Aerobic” or “Exercise Training” or “Exercise Trainings” or “Training, Exercise” or “Trainings, Exercise”)  Indexes=SCI-EXPANDED, SSCI, A&HCI, CPCI-S, CPCI-SSH, BKCI-S, BKCI-SSH, ESCI, CCR-EXPANDED, IC Timespan=All years |  |  |
| # 3 | 17,738 | TOPIC: ("Resistance training” or “Training, Resistance” or “Strength Training” or “Training, Strength” or “Weight-Lifting Strengthening Program” or “Strengthening Program, Weight-Lifting” or “Strengthening Programs, Weight-Lifting” or “Weight Lifting Strengthening Program” or “Weight-Lifting Strengthening Programs” or “Weight-Lifting Exercise Program” or “Exercise Program, Weight-Lifting” or “Exercise Programs, Weight-Lifting” or “Weight Lifting Exercise Program” or “Weight-Lifting Exercise Programs” or “Weight-Bearing Strengthening Program” or “Strengthening Program, Weight-Bearing” or “Strengthening Programs, Weight-Bearing” or “Weight Bearing Strengthening Program” or “Weight-Bearing Strengthening Programs” or “Weight-Bearing Exercise Program”)  Indexes=SCI-EXPANDED, SSCI, A&HCI, CPCI-S, CPCI-SSH, BKCI-S, BKCI-SSH, ESCI, CCR-EXPANDED, IC Timespan=All years |  |  |
| # 2 | 424,147 | TOPIC: (“aerobic exercise” or “aquatic exercise” or “balance training” or “body weight support treadmill” or “Dance Therapy or exercise*” or “Exercise Movement Techniques” or “gait training” or “high-speed resistance training” or “hydrotherapy” or “multicomponent exercise program” or “multidisciplinary exercise program” or “Nordic Walking” or “Physiotherapy” or pilates or “power training” or Qigong or “resistance training” or “Robotic-assisted gait training” or stretch or “tai ji” or Tango or “treadmill training” or “walking” or “Virtual Reality” or “whole body vibration” or Yoga)  Indexes=SCI-EXPANDED, SSCI, A&HCI, CPCI-S, CPCI-SSH, BKCI-S, BKCI-SSH, ESCI, CCR-EXPANDED, IC Timespan=All years |  |  |
| # 1 | 113,262 | TOPIC: ("Idiopathic Parkinson's Disease" or "Lewy Body Parkinson's Disease" or "Parkinson's Disease, Idiopathic" or "Parkinson's Disease, Lewy Body" or "Parkinson Disease, Idiopathic" or "Parkinson's Disease" or "Idiopathic Parkinson Disease" or "Lewy Body Parkinson Disease" or "Primary Parkinsonism" or "Parkinsonism, Primary" or "Paralysis Agitans")  Indexes=SCI-EXPANDED, SSCI, A&HCI, CPCI-S, CPCI-SSH, BKCI-S, BKCI-SSH, ESCI, CCR-EXPANDED, IC Timespan=All years |  |  |

# Appendix 4: Outcomes

## Primary outcomes

Motor symptoms: such as unified Parkinson’s disease rating scale (UPDRS) ^1^ and the Movement Disability Society (MDS) released in 2007 the revised MDS-UPDRS based on the UPDRS.^2^ These scales are made up of these sections: Part I: evaluation of mentation, behavior, and mood; Part II: self-evaluation of the activities of daily life (ADLs) including speech, swallowing, handwriting, dressing, hygiene, falling, salivating, turning in bed, walking, and cutting food; Part III: clinician-scored monitored motor evaluation; Part IV: complications of therapy. Both scales that was developed as effort to incorporate elements from existing scales to provide comprehensive but efficient and flexible means to monitor Parkinson’ s disease (PD)-related disability and impairment. Due to the low UPDRS total (n=36) reported in the included literature, we only selected UPDRS Ⅲ for motor symptoms (Appendix 8).

## Secondary outcomes

Parkinson’s disease is a neuro-degenerative disease with early prominent death of dopaminergic neurons in the substantia nigra pars compacta (SNpc). The lack of dopamine produced in the basal ganglia can cause movement disorders, which are characterized by classic Parkinson's disease motor symptoms. Parkinson's disease is also associated with many non-motor symptoms, some of which have existed for more than a decade before motor dysfunction. We use the changes in gait and balance ability before and after interventional physical activity as the outcomes of the effect of various types of physical activity on motor symptoms. The Berg Balance Scale (BBS) was used as the most prominent balance test battery. BBS is a functional scale assessing static and dynamic postural control associated with 14 different activities.^3^ It has been validated for patients with PD.^4^ In addition, Balance Evaluation Systems Test (BESTest), Mini-Balance Evaluation Systems Test (Mini-BESTest), Tinetti assessment scale, and Fullerton Advanced Balance, are often used as a scale to evaluate the balance in PD, when BBS was not reported in the included literature, we used these as a surrogate outcome for balance. Gait outcomes: (a) The freezing of gait questionnaire (FOGQ) was developed to provide an overall assessment of gait difficulties related to FOG;^5^ (b) using the speed or time of walking test as an objective outcome to evaluate PD gait (e.g., 3-20m walking test); (c) using the 2-6 min walking test to evaluate walking distance. In addition, we evaluate non-motor symptoms from four directions: depression (e.g., Beck Depression Inventory, Geriatric Depression Scale, and hospital anxiety and depression scale-depression etc.), anxiety (e.g., Beck anxiety inventory, Geriatric Anxiety Inventory, and hospital anxiety and depression scale-anxiety etc.), sleep quality (e.g., PD Sleep Scale, and Pittsburgh Sleep Quality Index etc.), and cognition (e.g., Montreal Cognitive Assessment (MOCA), PDQ-39 cognition, Frontal assessment battery, and Color Trails Test etc.) (appendix 8, Table 8.2). We also evaluated the effect of physical activity on PD muscle strength (e.g., leg press etc.). We use the falls efficacy scale and activities-specific balance confidence scale as a subjective indicator to assess concern of falling.^6^

**Reference**

1. Ramaker C, Marinus J, Stiggelbout AM, Van Hilten BJ. Systematic evaluation of rating scales for impairment and disability in Parkinson's disease. *Mov Disord* 2002; **17**(5): 867-76.

2. Goetz CG, Fahn S, Martinez-Martin P, et al. Movement Disorder Society-sponsored revision of the Unified Parkinson's Disease Rating Scale (MDS-UPDRS): Process, format, and clinimetric testing plan. *Mov Disord* 2007; **22**(1): 41-7.

3. Berg KO, Maki BE, Williams JI, Holliday PJ, Wood-Dauphinee SL. Clinical and laboratory measures of postural balance in an elderly population. *Arch Phys Med Rehabil* 1992; **73**(11): 1073-80.

4. Qutubuddin AA, Pegg PO, Cifu DX, Brown R, McNamee S, Carne W. Validating the Berg Balance Scale for patients with Parkinson's disease: a key to rehabilitation evaluation. *Arch Phys Med Rehabil* 2005; **86**(4): 789-92.

5. Giladi, Shabtai, Simon, Biran, Tal, Korczyn. Construction of freezing of gait questionnaire for patients with Parkinsonism. *Parkinsonism Relat Disord* 2000; **6**(3): 165-70.

6. Bloem BR, Marinus J, Almeida Q, et al. Measurement instruments to assess posture, gait, and balance in Parkinson's disease: Critique and recommendations. *Mov Disord* 2016; **31**(9): 1342-55.

# Appendix 5: Definitions of physical activity types and non-exercise training control

| abbreviation | Full name | Definitions |
| --- | --- | --- |
| AE | Aerobic Exercise | Aerobic exercise is performed by repeating sequences of light-to-moderate intensity activities for extended periods of time.^1^ e.g., walking, bicycle, etc. Exclude treadmill training, because this study treats treadmill training as a separate physical activity type |
| AQE | Aquatic Exercise | Gait training, balance training, resistance training, or aerobic training performed in deep or shallow water.^2^ |
| BGT | Balance and Gait Training | Single-task balance and gait training without external cues or internal and external attention |
| BGT_ECA | Balance and Gait Training with external Cue or Attention | Focus on external cues or things while doing balance and gait training.^3^ |
| BGT_ICA | Balance and Gait Training with Internal Cue or Attention | Participants have been asked to focus on the movement of their limbs in the physical space while performing balance and gait training. An example of guidance given to this group during knee lift exercises is "focus on raising the knee in a slow, controlled manner.^4^ |
| BWS_TT | Body Weight Support Treadmill Training | Walking on a treadmill after reducing the weight of the body through the equipment |
| CON | Control group | Non-exercise intervention, usual care,^5^ or health education |
| CPP | Classic Physiotherapy Program | This program was performed according to the guidelines for physical therapy in patients with PD and included flexibility, strengthening, posture, breathing balance, walking exercises, and other functional activities.^6^ |
| Dance | - | Group dances other than tango, such as waltz, Irish set dancing etc. |
| DT-BGT | Dual Task Balance and Gait Training | The dual-task paradigm entails the simultaneous performance of two tasks with different objectives.^7^ This research is mainly to perform cognitive or motor tasks in addition to balance and gait training. |
| Mul_C | Multicomponent exercise program | Two or more of the above specific types of exercise training (if it is only part of warm-up or relaxation, it is not considered as multi-mode) |
| Mul_D | Multidisciplinary exercise program | The content of intervention includes the above types of exercise, in addition to other disciplines of intervention content (e.g., cognitive training, nutritional supplements, electrical stimulation, etc.) |
| NW | Nordic Walking | A walking exercise that uses walking poles with both hands |
| Pilates | - | Pilates exercise focuses on posture symmetry, breathing control, abdominal strength, spine, pelvic and shoulder stability, muscle flexibility, joint flexibility, and enhancement of the full range of motion through all joints. Not isolated muscle groups, but whole body training, integrating the upper and lower limbs with the trunk.^8^ |
| PT | Power Training | Similar to resistance training, however participants were instructed to exert force as fast as possible during the concentric phase and move slowly through the eccentric phase.^9^ |
| Qigong | - | It is a system of coordinated body-posture and movement, breathing, and meditation used for the purposes of health, spirituality, and martial-arts training. |
| RA_GT | Robotic-Assisted Gait Training | Robot-assisted gait training uses electromechanical equipment to assist the stepping cycle by supporting body weight, while automating the gait process by supporting and promoting the movement of one or more lower limb joints.^10^ |
| RT | Resistance Training | Exercise training designed to improve the strength, power, endurance and size of skeletal muscles.^11^ |
| Stretch | - | Reverse extension of muscle length to improve joint mobility |
| Tango | - | An argentine couple dance. When dancing tango, the two sides lean closely together. The right arm of the man and the left arm of the woman should be inward. The body is in contact with each other and the center of gravity is shifted. The man is mainly on the right foot and the woman is on the left foot. Both men and women do not look at each other, and both men and women look to their left side when positioning. |
| TC | Tai Chi | It is an internal Chinese martial art practiced for defense training, health benefits, and meditation. |
| TT | Treadmill Training | Walking on a treadmill at a constant speed. |
| VR | Virtual Reality | Use computer simulation to generate a virtual environment, provide users with a simulation of vision and other senses, so that users can feel as if they are immersed in the environment, and perform the above types of exercises on this basis |
| WBV | Whole Body Vibration | Exposure to low-amplitude, low-frequency mechanical stimulation throughout the body for a certain period of time^12^ |
| Yoga | - | Mainly a series of methods for self-cultivation, including body-adjusting asanas (refer to yoga asana collection), breathing-adjusting breathing methods, and mind-adjusting meditation, etc., to achieve the unity of body and mind.^13^ |

**Reference**

1. Plowman SA, Smith DL. Exercise physiology for health fitness and performance: Lippincott Williams & Wilkins; 2013.

2. Konlian C. Aquatic therapy: making a wave in the treatment of low back injuries. *Orthop Nurs* 1999; **18**(1).

3. Abdollahipour R, Wulf G, Psotta R, Palomo Nieto M. Performance of gymnastics skill benefits from an external focus of attention. *J Sports Sci* 2015; **33**(17): 1807-13.

4. Beck EN, Intzandt BN, Almeida QJ. Can Dual Task Walking Improve in Parkinson's Disease After External Focus of Attention Exercise? A Single Blind Randomized Controlled Trial. *Neurorehabil Neural Repair* 2018; **32**(1): 18-33.

5. Goh S-L, Persson MSM, Stocks J, et al. Relative Efficacy of Different Exercises for Pain, Function, Performance and Quality of Life in Knee and Hip Osteoarthritis: Systematic Review and Network Meta-Analysis. *Sports Med* 2019; **49**(5): 743-61.

6. Keus SHJ, Bloem BR, Hendriks EJM, Bredero-Cohen AB, Munneke M. Evidence-based analysis of physical therapy in Parkinson's disease with recommendations for practice and research. *Mov Disord* 2007; **22**(4).

7. McIsaac TL, Lamberg EM, Muratori LM. Building a framework for a dual task taxonomy. *Biomed Res Int* 2015; **2015**: 591475.

8. Muscolino JE, Cipriani S. Pilates and the “powerhouse”—I. *Journal of bodywork and movement therapies* 2004; **8**(1): 15-24.

9. Ni M, Signorile JF, Mooney K, et al. Comparative Effect of Power Training and High-Speed Yoga on Motor Function in Older Patients With Parkinson Disease. *Arch Phys Med Rehabil* 2016; **97**(3).

10. Nedergård H, Arumugam A, Sandlund M, Bråndal A, Häger CK. Effect of robotic-assisted gait training on objective biomechanical measures of gait in persons post-stroke: a systematic review and meta-analysis. *J Neuroeng Rehabil* 2021; **18**(1): 64.

11. Powell KE, Paluch AE, Blair SN. Physical activity for health: What kind? How much? How intense? On top of what? *Annu Rev Public Health* 2011; **32**: 349-65.

12. Bidonde J, Busch AJ, van der Spuy I, Tupper S, Kim SY, Boden C. Whole body vibration exercise training for fibromyalgia. *Cochrane Database Syst Rev* 2017; **9**: CD011755.

13. Cramer H, Lauche R, Haller H, Dobos G. A systematic review and meta-analysis of yoga for low back pain. *Clin J Pain* 2013; **29**(5): 450-60.

# Appendix 6: Statistical methods in details

## Network meta-analysis

We will use R software gemtc and rjags packages to perform Bayesian network meta-analysis. Using arm-level data and import into the R software in CSV format. The effect size measure for continuous outcomes chooses the standardized mean difference (SMD) of the change score (end-point minus baseline score) because the studies use different rating scales or units of clinician disability, motor outcomes, non-motor outcomes, muscle strength, and concern of falling. The normal likelihood for continuous outcomes. The study effect sizes were then synthesized using a random-effects network meta-analysis model. In addition, we will present the summary SMD, 95% credible intervals (CrIs) for all pairwise comparisons in the league table, and we show the results of comparing the outcomes of each exercise intervention group and the control group in the form of a forest plot. To rank the various treatments for each outcome, we will use the surface under the cumulative ranking curve (SUCRA). In the process of extracting data, if the original study reported a standard error in the experimental and control groups, the standard deviation was calculated by the formula: standard deviation (SD) = standard error (SE) × √n. If both are missing, we will estimate SD based on the confidence interval, t-value, quartile, range, or p-values as described in section 7.7.3 of the Cochrane Handbook for Systematic Reviews. When only figures were presented, data were extracted using GetData (http://getdata-graph-digitizer.com) to measure the length (in pixels) of the axes to calibrate and then the length in pixels from the relevant axis to the data points of interest. If the data needed for the study cannot be extracted from the above methods, we will ask the authors about the data at least 4 times within 6 weeks.

## Continuous variable-random effects model:

For any physical activity intervention x in any randomized controlled experiment i, the sample size is n_i, x_. The effect of treatment is y_i, x_ (change from baseline), and standard error is se_i, x_. Then the normal

likelihood is employed to y_i, x_ ~ N (𝜃_i, y_, se_i, x_) in each arm. In addition, for any randomized controlled experiment, there should be a basic physical activity type b(i), and its effect is represented by y_i, b(i)_

y_i, b(i)_ = 𝑢_𝑖_

In the random effects model, for any interventional physical activity k that is not a basic physical activity type, its exercise effect is:

𝜃_i, k_= 𝑢_𝑖_+𝛿_i, b(i), k_ for k ≥ 2

Where 𝛿_i, b(i), k_ is the difference between the effect of physical activity k and basic physical activity b, and conforms to the following normal distribution 𝛿_i, x, y_ ~ N(d_x,y_, 𝞼^2^_x, y_), where d_x,y_ is the relative effect of physical activity intervention y and x, 𝞼^2^_x, y_ is the variance of the relative effect of physical activity intervention y and x. In addition, this study presented the final treatment effect with standardised mean difference (SMD), so the above formula is modified:

y_i, b(i)_ = 𝑢_𝑖_/S_i_

𝜃_i, k_= (𝑢_𝑖_+𝛿_i, b(i), y_)/ S_i_ for k ≥ 2

where S_i_ is the pooled standard deviation in the study arms.

model {

# Likelihood for arm-based data

## OMITTED

# Likelihood for contrast-based data (univariate for 2-arm trials)

for(i in studies.r2) {

for (k in 2:na[i]) {

mest[i, k] <- delta[i, k]

}

m[i, 2] ~ dnorm(mest[i, 2], prec[i, 2])

prec[i, 2] <- 1 / (e[i, 2] * e[i, 2])

dev[i, 1] <- pow(m[i, 2] - mest[i, 2], 2) * prec[i, 2]

}

# Likelihood for contrast-based data (multivariate for multi-arm trials)

for(i in studies.rm) {

for (k in 2:na[i]) {

mest[i, k] <- delta[i, k]

}

for (k in 1:(na[i]-1)) {

for (j in 1:(na[i]-1)) {

Sigma[i,j,k] <- ifelse(equals(j, k), pow(e[i,k+1], 2), pow(e[i,1], 2))

}

}

Omega[i,1:(na[i]-1),1:(na[i]-1)] <- inverse(Sigma[i,1:(na[i]-1),1:(na[i]-1)])

m[i,2:na[i]] ~ dmnorm(mest[i,2:na[i]], Omega[i,1:(na[i]-1),1:(na[i]-1)])

mdiff[i, 2:na[i]] <- m[i, 2:na[i]] - mest[i, 2:na[i]]

dev[i, 1] <- t(mdiff[i, 2:na[i]]) %*% Omega[i, 1:(na[i]-1),1:(na[i]-1)] %*% mdiff[i, 2:na[i]]

}

# Random effects model

for (i in studies) {

# Study-level relative effects

w[i, 1] <- 0

delta[i, 1] <- 0

for (k in 2:na[i]) { # parameterize multi-arm trials using a trick to avoid dmnorm

delta[i, k] ~ dnorm(md[i, k], taud[i, k])

md[i, k] <- d[t[i, 1], t[i, k]] + sw[i, k]

taud[i, k] <- tau.d * 2 * (k - 1) / k

w[i, k] <- delta[i, k] - (d[t[i, 1], t[i, k]])

sw[i, k] <- sum(w[i, 1:(k-1)]) / (k - 1)

}

}

# Random effects variance prior

sd.d ~ dunif(0, om.scale)

tau.d <- pow(sd.d, -2)

# Relative effect matrix

d[1, 1] <- 0

d[1, 2] <- d.AE.CON + d.CON.AQE

d[1, 3] <- d.AE.BGT

d[1, 4] <- d.AE.BGT_ECA

d[1, 5] <- d.AE.BGT_ECA + d.BGT_ECA.BGT_ICA

d[1, 6] <- d.AE.BGT + d.BGT.BWS_TT

d[1, 7] <- d.AE.CON

d[1, 8] <- d.AE.BGT + d.BGT.CPP

d[1, 9] <- d.AE.CON + d.CON.Dance

d[1, 10] <- d.AE.BGT + d.BGT.DT_BGT

d[1, 11] <- d.AE.Mul_C

d[1, 12] <- d.AE.BGT_ECA + d.BGT_ECA.Mul_D

d[1, 13] <- d.AE.CON + d.CON.NW

d[1, 14] <- d.AE.Mul_C + d.Mul_C.Pilates

d[1, 15] <- d.AE.CON + d.CON.PT

d[1, 16] <- d.AE.Qigong

d[1, 17] <- d.AE.RA_GT

d[1, 18] <- d.AE.RT

d[1, 19] <- d.AE.Stretch

d[1, 20] <- d.AE.CON + d.CON.Tango

d[1, 21] <- d.AE.CON + d.CON.TC

d[1, 22] <- d.AE.TT

d[1, 23] <- d.AE.VR

d[1, 24] <- d.AE.VR + d.VR.WBV

d[1, 25] <- d.AE.CON + d.CON.Yoga

for (i in 2:nt) {

for (j in 1:nt) {

d[i, j] <- d[1, j] - d[1, i]

}

}

prior.prec <- pow(re.prior.sd, -2)

# Study baseline priors

## OMITTED

# Effect parameter priors

d.AE.BGT ~ dnorm(0, prior.prec)

d.AE.BGT_ECA ~ dnorm(0, prior.prec)

d.AE.CON ~ dnorm(0, prior.prec)

d.AE.Mul_C ~ dnorm(0, prior.prec)

d.AE.Qigong ~ dnorm(0, prior.prec)

d.AE.RA_GT ~ dnorm(0, prior.prec)

d.AE.RT ~ dnorm(0, prior.prec)

d.AE.Stretch ~ dnorm(0, prior.prec)

d.AE.TT ~ dnorm(0, prior.prec)

d.AE.VR ~ dnorm(0, prior.prec)

d.BGT.BWS_TT ~ dnorm(0, prior.prec)

d.BGT.CPP ~ dnorm(0, prior.prec)

d.BGT.DT_BGT ~ dnorm(0, prior.prec)

d.BGT_ECA.BGT_ICA ~ dnorm(0, prior.prec)

d.BGT_ECA.Mul_D ~ dnorm(0, prior.prec)

d.CON.AQE ~ dnorm(0, prior.prec)

d.CON.Dance ~ dnorm(0, prior.prec)

d.CON.NW ~ dnorm(0, prior.prec)

d.CON.PT ~ dnorm(0, prior.prec)

d.CON.Tango ~ dnorm(0, prior.prec)

d.CON.TC ~ dnorm(0, prior.prec)

d.CON.Yoga ~ dnorm(0, prior.prec)

d.Mul_C.Pilates ~ dnorm(0, prior.prec)

d.VR.WBV ~ dnorm(0, prior.prec)

}

## Network meta-regression: In the network meta-regression models we set

𝜃 𝑖_,𝑘_ =𝜃 +𝛽_1,𝜅_ ×(𝑥 _𝑖_ −𝑟𝑒𝑓 _𝑥_)

In the model with independent and consistent coefficients we define 𝛽_1,k_ = 𝛽_k_. if treatment 1 is control group (non-exercise intervention or usual care); otherwise 𝛽_1, k_ ≡ 𝛽_AB_ = 𝛽_B_ − 𝛽_A_ for any treatments 𝐴, 𝐵. In the model with exchangeable coefficients we set 𝛽_K_ ~𝑁(𝛣, 𝜏^2^_B_ ).

model {

# Likelihood for arm-based data

## OMITTED

# Likelihood for contrast-based data (univariate for 2-arm trials)

for(i in studies.r2) {

for (k in 2:na[i]) {

mest[i, k] <- delta[i, k] + (beta[t[i, k]] - beta[t[i, 1]]) * x[i]

}

m[i, 2] ~ dnorm(mest[i, 2], prec[i, 2])

prec[i, 2] <- 1 / (e[i, 2] * e[i, 2])

dev[i, 1] <- pow(m[i, 2] - mest[i, 2], 2) * prec[i, 2]

}

# Likelihood for contrast-based data (multivariate for multi-arm trials)

for(i in studies.rm) {

for (k in 2:na[i]) {

mest[i, k] <- delta[i, k] + (beta[t[i, k]] - beta[t[i, 1]]) * x[i]

}

for (k in 1:(na[i]-1)) {

for (j in 1:(na[i]-1)) {

Sigma[i,j,k] <- ifelse(equals(j, k), pow(e[i,k+1], 2), pow(e[i,1], 2))

}

}

Omega[i,1:(na[i]-1),1:(na[i]-1)] <- inverse(Sigma[i,1:(na[i]-1),1:(na[i]-1)])

m[i,2:na[i]] ~ dmnorm(mest[i,2:na[i]], Omega[i,1:(na[i]-1),1:(na[i]-1)])

mdiff[i, 2:na[i]] <- m[i, 2:na[i]] - mest[i, 2:na[i]]

dev[i, 1] <- t(mdiff[i, 2:na[i]]) %*% Omega[i, 1:(na[i]-1),1:(na[i]-1)] %*% mdiff[i, 2:na[i]]

}

# Random effects model

for (i in studies) {

# Study-level relative effects

w[i, 1] <- 0

delta[i, 1] <- 0

for (k in 2:na[i]) { # parameterize multi-arm trials using a trick to avoid dmnorm

delta[i, k] ~ dnorm(md[i, k], taud[i, k])

md[i, k] <- d[t[i, 1], t[i, k]] + sw[i, k]

taud[i, k] <- tau.d * 2 * (k - 1) / k

w[i, k] <- delta[i, k] - (d[t[i, 1], t[i, k]])

sw[i, k] <- sum(w[i, 1:(k-1)]) / (k - 1)

}

}

# Random effects variance prior

sd.d ~ dunif(0, om.scale)

tau.d <- pow(sd.d, -2)

# Relative effect matrix

d[1, 1] <- 0

d[1, 2] <- d.AE.AQE

d[1, 3] <- d.AE.BGT

d[1, 4] <- d.AE.BGT_ECA

d[1, 5] <- d.AE.BGT_ECA + d.BGT_ECA.BGT_ICA

d[1, 6] <- d.AE.BGT + d.BGT.BWS_TT

d[1, 7] <- d.AE.CON

d[1, 8] <- d.AE.AQE + d.AQE.CPP

d[1, 9] <- d.AE.CON + d.CON.Dance

d[1, 10] <- d.AE.AQE + d.AQE.DT_BGT

d[1, 11] <- d.AE.Mul_C

d[1, 12] <- d.AE.AQE + d.AQE.Mul_D

d[1, 13] <- d.AE.CON + d.CON.NW

d[1, 14] <- d.AE.Mul_C + d.Mul_C.Pilates

d[1, 15] <- d.AE.CON + d.CON.PT

d[1, 16] <- d.AE.Qigong

d[1, 17] <- d.AE.RA_GT

d[1, 18] <- d.AE.RT

d[1, 19] <- d.AE.Stretch

d[1, 20] <- d.AE.CON + d.CON.Tango

d[1, 21] <- d.AE.CON + d.CON.TC

d[1, 22] <- d.AE.TT

d[1, 23] <- d.AE.VR

d[1, 24] <- d.AE.VR + d.VR.WBV

d[1, 25] <- d.AE.CON + d.CON.Yoga

for (i in 2:nt) {

for (j in 1:nt) {

d[i, j] <- d[1, j] - d[1, i]

}

}

prior.prec <- pow(re.prior.sd, -2)

# Study baseline priors

## OMITTED

# Effect parameter priors

d.AE.AQE ~ dnorm(0, prior.prec)

d.AE.BGT ~ dnorm(0, prior.prec)

d.AE.BGT_ECA ~ dnorm(0, prior.prec)

d.AE.CON ~ dnorm(0, prior.prec)

d.AE.Mul_C ~ dnorm(0, prior.prec)

d.AE.Qigong ~ dnorm(0, prior.prec)

d.AE.RA_GT ~ dnorm(0, prior.prec)

d.AE.RT ~ dnorm(0, prior.prec)

d.AE.Stretch ~ dnorm(0, prior.prec)

d.AE.TT ~ dnorm(0, prior.prec)

d.AE.VR ~ dnorm(0, prior.prec)

d.AQE.CPP ~ dnorm(0, prior.prec)

d.AQE.DT_BGT ~ dnorm(0, prior.prec)

d.AQE.Mul_D ~ dnorm(0, prior.prec)

d.BGT.BWS_TT ~ dnorm(0, prior.prec)

d.BGT_ECA.BGT_ICA ~ dnorm(0, prior.prec)

d.CON.Dance ~ dnorm(0, prior.prec)

d.CON.NW ~ dnorm(0, prior.prec)

d.CON.PT ~ dnorm(0, prior.prec)

d.CON.Tango ~ dnorm(0, prior.prec)

d.CON.TC ~ dnorm(0, prior.prec)

d.CON.Yoga ~ dnorm(0, prior.prec)

d.Mul_C.Pilates ~ dnorm(0, prior.prec)

d.VR.WBV ~ dnorm(0, prior.prec)

# Regression priors

reg.prior.prec <- pow(om.scale, -2)

for (k in c(1:(reg.control-1), (reg.control+1):nt)) {

beta[k] <- B

}

beta[reg.control] <- 0

B ~ dt(0, reg.prior.prec, 1)

## Assessment of the transitivity assumption

Different clinical trials need to ensure that their baseline levels are consistent. If the baseline levels are inconsistent, the results cannot be transitive. Therefore, the transitivity assumption was evaluated by comparing the distribution of potential effect modifiers (publication year, sample size, mean age, percentage male, years of diagnosis, and disease grade) (appendix 7) across studies grouped before analyzing the results.

## Assessment of heterogeneity and inconsistency

We use the tau square (τ^2^) test and p-value to qualitatively analyze the statistical heterogeneity between the studies. The larger the τ^2^ and the smaller the p-value, the greater the possibility of heterogeneity; on the contrary, the smaller the existence heterogeneity. In addition, I^2^ is a parameter for quantitative analysis of the heterogeneity between the results of each study. It’ s value is distributed from 0-100%. When I^2^ is less than 25%, it means that the heterogeneity is low; 25%-50% means that the heterogeneity is moderate; I^2^ > 75% means high heterogeneity. In summary, when I^2^ > 50%, it means that there is substantial heterogeneity. We will use global and local methods to test the inconsistency of the research results. For global inconsistency, we evaluated inconsistency statistically using the design-by-treatment test. In addition, we will assessment of local inconsistency by separating indirect from direct evidence (SIDE test) using the R netmeta package. The potential reasons of heterogeneity (publish year, sample size, mean age, percentage male, years of diagnosis, disease grade, exercise duration, exercise frequency, the total time of single session, and outcomes test ON/OFF dopaminergic medication) will be explored by network meta-regression.

## Publication bias

We compared the adjusted funnel plot to assess the risk of publication bias under specific circumstances. In addition, we made a linear fit for the primary outcome (appendix 13). When the fitted line coincides with the 0 quadrant, it means that there is no published bias. For multi-arm trails, for example, physical activity type A, B and control, we plotted control group vs physical activity type A, and control group and physical activity type B

## Assessment of sensitivity

We assessed the sensitivity of our findings by repeating each network meta-analysis after excluding studies at overall high risk of bias, sample size less than 20, exercise duration less than 4 weeks and more than 24 weeks, exercise frequency less than 2 and more than 4, OFF state during the test, Chinese studies, unpublished studies, and data were extracted using GetData and estimated standard deviations value. In addition, we present the results from the changes in heterogeneity in each sensitivity analysis (appendix 15).

## Statistical software

The analysis and presentation of results will be performed using the Stata packages network, the R package rjags, gemtc, netmeta, ggplot2, and forestplot.

# Appendix 7: Assessment of the transitivity

Different clinical trials need to ensure that their baseline levels are consistent. If the baseline levels are inconsistent, the results cannot be transitive. Therefore, the transitivity assumption was evaluated by comparing the distribution of potential effect modifiers (publication year, sample size, mean age, percentage male, years of diagnosis, and disease grade) across studies grouped before analyzing the results, and we use the R ggplot2 package to draw boxplots between the above potential influencing factors and various types of physical activity.

## 7.1 Publish years

We checked the publication year distribution of the included studies. The range is from 1996 to 2021, with a median of 2017. In addition, we examined the impact of the included study’s publication year as a potential influencing factor in a meta-regression (appendix 14.1).

**Figure 7.1:** Boxplot for distribution of publication year. *PT* Power Training, *TC* Tai Chi, *RA_GT* Robotic Assisted Gait Training, *BWS_TT* Body Weight Support Treadmill Training, *Mul_D* Multidisciplinary Exercise Program *AE* Aerobic Exercise, *BGT_ECA* Balance and Gait Training with External Cue or Attention, *DT_BGT* Dual Task Balance and Gait Training, *BGT_ICA* Balance and Gait Training with Internal Cue or Attention, *Mul_C* Multicomponent Exercise Program, RT Resistance Training, *AQE* Aquatic Exercise, *VR* Virtual Reality, *TT* Treadmill Training, *BGT* Balance and Gait Training, *WBV* Whole Body Vibration, *CPP* Classic Physiotherapy Program, *NW* Nordic Walking, *CON* Control group.

## 7.2 Mean age

We checked the mean age distribution of the included study participants . The range is from 53.2 to 81.4, with a median of 67.5. In addition, we examined the impact of the mean age as a potential influencing factor in a metaregression (appendix 14.2).

**Figure 7.2 :** Boxplot for distribution of mean age. *PT* Power Training, *TC* Tai Chi, *RA_GT* Robotic Assisted Gait Training, *BWS_TT* Body Weight Support Treadmill Training, *Mul_D* Multidisciplinary Exercise Program *AE* Aerobic Exercise, *BGT_ECA* Balance and Gait Training with External Cue or Attention, *DT_BGT* Dual Task Balance and Gait Training, *BGT_ICA* Balance and Gait Training with Internal Cue or Attention, *Mul_C* Multicomponent Exercise Program, RT Resistance Training, *AQE* Aquatic Exercise, *VR* Virtual Reality, *TT* Treadmill Training, *BGT* Balance and Gait Training, *WBV* Whole Body Vibration, *CPP* Classic Physiotherapy Program, *NW* Nordic Walking, *CON* Control group.

## 7.3 Years of diagnosis

We checked the year of diagnosis distribution of the included study participants . The range is from 1 to 17, with a median of 6.4. In addition, we examined the impact of the mean age as a potential influencing factor in a metaregression (appendix 14.3).

**Figure 7.3 :** Boxplot for distribution of year of diagnosis. *PT* Power Training, *TC* Tai Chi, *RA_GT* Robotic Assisted Gait Training, *BWS_TT* Body Weight Support Treadmill Training, *Mul_D* Multidisciplinary Exercise Program *AE* Aerobic Exercise, *BGT_ECA* Balance and Gait Training with External Cue or Attention, *DT_BGT* Dual Task Balance and Gait Training, *BGT_ICA* Balance and Gait Training with Internal Cue or Attention, *Mul_C* Multicomponent Exercise Program, RT Resistance Training, *AQE* Aquatic Exercise, *VR* Virtual Reality, *TT* Treadmill Training, *BGT* Balance and Gait Training, *WBV* Whole Body Vibration, *CPP* Classic Physiotherapy Program, *NW* Nordic Walking, *CON* Control group.

## 7.4 Hoehn and Yahr stage

We checked the Hoehn and Yahr stage distribution of the included study participants . The range is from 1 to 3.5, with a median of 2.4 In addition, we examined the impact of the Hoehn and Yahr stage as a potential influencing factor in a metaregression (appendix 14.4).

**Figure 7.4 :** Boxplot for distribution of Hoehn and Yahr stage. *PT* Power Training, *TC* Tai Chi, *RA_GT* Robotic Assisted Gait Training, *BWS_TT* Body Weight Support Treadmill Training, *Mul_D* Multidisciplinary Exercise Program *AE* Aerobic Exercise, *BGT_ECA* Balance and Gait Training with External Cue or Attention, *DT_BGT* Dual Task Balance and Gait Training, *BGT_ICA* Balance and Gait Training with Internal Cue or Attention, *Mul_C* Multicomponent Exercise Program, RT Resistance Training, *AQE* Aquatic Exercise, *VR* Virtual Reality, *TT* Treadmill Training, *BGT* Balance and Gait Training, *WBV* Whole Body Vibration, *CPP* Classic Physiotherapy Program, *NW* Nordic Walking, *CON* Control group.

## 7.5 Percentage male

We checked the percentage male distribution of the included study participants . The range is from 0 to 100%, with a median of 60. In addition, we examined the impact of the percentage male as a potential influencing factor in a metaregression (appendix 14.5).

**Figure 7.5 :** Boxplot for distribution of percentage male. *PT* Power Training, *TC* Tai Chi, *RA_GT* Robotic Assisted Gait Training, *BWS_TT* Body Weight Support Treadmill Training, *Mul_D* Multidisciplinary Exercise Program *AE* Aerobic Exercise, *BGT_ECA* Balance and Gait Training with External Cue or Attention, *DT_BGT* Dual Task Balance and Gait Training, *BGT_ICA* Balance and Gait Training with Internal Cue or Attention, *Mul_C* Multicomponent Exercise Program, RT Resistance Training, *AQE* Aquatic Exercise, *VR* Virtual Reality, *TT* Treadmill Training, *BGT* Balance and Gait Training, *WBV* Whole Body Vibration, *CPP* Classic Physiotherapy Program, *NW* Nordic Walking, *CON* Control group.

## 7.6 Sample size

We checked the sample size distribution of the included studies. The range is from 4 to 381, with a median of 18. In addition, we examined the impact of the included study’s sample size as a potential influencing factor in a meta-regression (appendix 14.6)

**Figure 7.6 :** Boxplot for distribution of sample size. *PT* Power Training, *TC* Tai Chi, *RA_GT* Robotic Assisted Gait Training, *BWS_TT* Body Weight Support Treadmill Training, *Mul_D* Multidisciplinary Exercise Program *AE* Aerobic Exercise, *BGT_ECA* Balance and Gait Training with External Cue or Attention, *DT_BGT* Dual Task Balance and Gait Training, *BGT_ICA* Balance and Gait Training with Internal Cue or Attention, *Mul_C* Multicomponent Exercise Program, RT Resistance Training, *AQE* Aquatic Exercise, *VR* Virtual Reality, *TT* Treadmill Training, *BGT* Balance and Gait Training, *WBV* Whole Body Vibration, *CPP* Classic Physiotherapy Program, *NW* Nordic Walking, *CON* Control group.

# Appendix 8: Characteristics of studies and subjects included in the review

After screening the studies, all relevant articles were assessed for eligibility based on their full texts. At this stage, Three pairs of investigator extracted information on (1) relevant data regarding participant characteristics (e.g., the sample size, age, sex, years of diagnosis, and disease grade); (2) types and daily doses of medicines taken; (3) OFF (>12 hour withdrawal from dopaminergic medication) and ON (1-2 hour after taking their normal dopaminergic medication); (3) physical activity type; (4) training variable (e.g., duration, frequency, and single course time); and (5) the main result of the study. The extracted data of the included studies were depicted in Table 8.1, and 8.2

## 8.1 Characteristics of eligible RCTs included in network meta-analysis

| **Author** | **Publish**  **Year** | **Age**  **(Mean ± SD)** | **Number**  **(men)** | **duration of diagnosis**  **(year/month)** | **Hoehn and Yahr stage**  **(1-5)** | **Drug**  **(mg/d)** | **ON/OFF** | **Period**  **(week)** | **Frequency** | **Single course time (minute)** |
| --- | --- | --- | --- | --- | --- | --- | --- | --- | --- | --- |
| Abraham, Hart ^1^ | 2018 | Mul_D: 66.4±12.5 CON: 65.1±7.5 | Mul_D:10(9) CON:10(7) | Mul_D: 6.1±3.8 CON: 8.5±4.5 | Mul_D: 2.0±0.52 CON: 2.0±0.37 | stable medication use | ON | 2 | 5 | 120 |
| Ajimsha, Majeed ^2^ | 2014 | Mul_C: 61.4±2.6  CPP: 60.8±2.1 | Mul_C: 32(17) CPP: 33(16) | Mul_C: 3.0±0.6 CPP: 3.1±0.5 | 2-3 | stable medication usage | ON | 8 | 5 | 75 |
| Allen, Canning ^3^ | 2010 | MUL_C: 66.0±10.0 CON: 68.0±7.0 | Mul_C: 24(13) CON: 24(13) | Mul_C: 7.0±5.0  CON: 9.0±6.0 | NA | stable medication use | ON | 24 | 3 | 50 |
| Allen, Song ^4^ | 2017 | VR: 67.5±7.3 CON: 68.4±8.5 | VR: 19(12) CON: 19(11) | VR: 7.9±3.9 CON: 8.7±6.1 | NA | Levodopa equivalent dose  VR: 939±531 CON: 711±703 | ON | 12 | 3 | NA |
| Altmann, Stegemöller ^5^ | 2016 | AE: 62.8±8.6 BGT: 63.3±7.3 CON: 67.8±9.8 | AE: 11(NA) BGT: 9(NA) CON: 10(NA) | NA | AE: 2.1±0.5 BGT: 2.2±0.4 CON: 2.4±0.7 | stable medication usage | ON | 16 | 3 | 35 |
| Amano, Nocera ^6^ | 2013 | TC: 64±13 Qigong: 68±7 TC: 66±11  CON: 66±7 | TC: 12(5) Qigong: 9(2) TC: 15(8) CON: 9(2) | TC: 7±7 Qigong: 12±7 TC: 8±5 CON: 5±3 | TC: 2.3±0.4 Qigong: 2.2±0.4 TC: 2.4±0.6 CON: 2.4±0.4 | NA | ON | 16 | 2 | 60 |
| Amara, Wood ^7^ | 2020 | Mul_C: 65.3±8.2 CON: 65.8±5.2 | Mul_C: 27(16) CON: 28(19) | Mul_C: 6.0±4.4 CON: 3.0±4.8 | NA | levodopa Mul_C: 640±307.4 CON: 482.5±332.4 | ON | 16 | 3 | NA |
| Arcolin, Pisano ^8^ | 2016 | TT: 67.8±8.8 AE: 68.7±8.3 | TT: 13(6) AE: 16(9) | TT: 6.5±2.9 AE: 4.7±2.9 | TT: 2.3±0.5 AE: 2.3±0.5 | stable medication usage | ON | 3 | 5 | 60 |
| Arfa-Fatollahkhani, Safar Cherati ^9^ | 2019 | CON: 61.55±8.57  TT: 60.63±9.36 | CON: 9(7) TT: 11(8) | CON: 8.50±6.34 TT: 8.89±5.14 | CON: 2.0±0.35 TT: 2.13±0.32 | Dopamine agonists CON: 1019.44±430.62 TT: 1000.0±474.34 | ON | 10 | 2 | 30 |
| Ashburn, Fazakarley ^10^ | 2007 | Mul_C: 72.7±9.6 CON: 71.6±8.8 | Mul_C: 70(38) CON: 72(48) | Mul_C: 7.7±5.8 CON: 9.0±5.8 | Mul_C: 3.14±0.59 CON: 3.09±0.55 | NA | NA | 6 | NA | 60 |
| Atan, Özyemişci Taşkıran ^11^ | 2019 | TT: 69.7±8 BWS_TT 72.2±7.9 BWS_TT: 68.6±8.2 | TT: 10(3) BWS_TT: 10(4) BWS_TT: 10(4) | TT: 69.7±8 BWS_TT: 72.2±7.9 BWS_TT: 68.6±8.2 | TT: 2.6±0.7 BWS_TT: 2.8±0.6 BWS_TT: 2.7±0.7 | Levodopa equivalent dose TT: 698.1±207.2 BWS_TT: 696.5±195.5 BWS_TT: 884±253.6 | ON | 6 | 5 | 30 |
| Bakhshayesh, Sayyar ^12^ | 2017 | Pilates: 57.0±6.24  BGT: 58.31±7.37 | 30(18) | Pilates: 7.27±3.80  BGT: 8.19±3.14 | 2-3 | stable medication usage | OFF | 8 | 3 | 60 |
| Bang and Shin ^13^ | 2016 | NW: 58.30±7.71 TT: 60.60±6.74 | NW: 10(5) TT: 10(4) | months NW: 18.10±6.77 TT: 17.98±3.28 | NW: 2.32±0.52 TT: 2.56±0.51 | stable medication use | NA | 4 | 5 | 60 |
| Barboza, Terra ^14^ | 2019 | CPP: 64.33±7.77  Mul_D: 67.11±8.14 | CPP: 26(12) Mul_D: 28(10) | CPP: 6.0±1.25 Mul_D: 4.5±1.81 | CPP: 2.5±0.25 Mul_D: 2.5±0.375 | Levodopa CPP: 500 ±125 Mul_D: 500±93.75 | ON | 16 | 2 | 75 |
| Beck, Intzandt ^15^ | 2017 | BGT_ECA: 68.6±9.9 BGT_ICA: 73.1±7.8 CON: 71.3±6.6 | BGT_ECA: 19 (15) BGT_ICA: 20 (16) CON: 11 (10） | BGT_ECA: 7.0 ±5.0 BGT_ICA: 6.7±4.2 CON: 8.4±5.9 | NA | levodopa  BGT_ECA: 648.0±232.9 BGT_ICA: 594.0±358.3 CON: 867.7±674.6 | ON/OFF | 11 | 3 | 60 |
| Beck, Wang ^16^ | 2020 | BGT_ICA 73.0±8.06 BGT_ECA: 65.4±6.21  CON: 72.0±5.52 | BGT_ICA: 15(12) BGT_ECA: 15(12) CON: 9(8) | BGT_ICA: 6.73±3.73  BGT_ECA: 6.60±5.18 CON: 8.67±6.02 | NA | levodopa equivalent dose BGT_IC: 599.38±369.47 BGT_ECA: 614.27±242.53  CON: 901.67±712.54 | ON/OFF | 11 | 3 | NA |
| Bekkers, Mirelman ^17^ | 2020 | TT: 70.9±6.0 VR: 71.1±6.3 | TT: 59(37) VR: 62(37) | TT: 9.6±7.2 VR: 9.1±5.5 | TT: 2.5±0.5 VR: 2.4±0.5 | NA | NA | 6 | 3 | 45 |
| Bello, Sanchez ^18^ | 2013 | TT: 59.45±11.32 BGT_ECA: 58±9.38 | TT: 11(7) BGT_ECA: 11(5) | TT: 4.82±3.28 BGT_ECA: 4.95±2.59 | TT: 2.27±0.41 BGT_ECA: 2.05±0.52 | stable medication use | ON | 5 | 3 | 25 |
| Burini, Farabollini ^19^ | 2006 | AE: 65.7±7.0 Qigong: 62.7±4.0 | AE: 13(5) Qigong: 13(4) | AE: 11.2±5.4 Qigong: 10.6±4.8 | AE: 2.8±0.4 Qigong: 2.7±0.5 | stable medication usage | ON | 7 | 3 | 40 |
| Cabrera-Martos, Jiménez-Martín ^20^ | 2020 | RT: 77.2±6.2 Stretch: 75.9±1.2 | RT: 22(15) Stretch: 22(11) | NA | 2-3 | Levodopa RT: 278.7±132.5 Stretch: 334.1±86.4 | ON | 8 | 3 | 45 |
| Cakit, Saracoglu ^21^ | 2007 | 71.8±6.4 (n=31) | CON: 10(NA) TT: 21(NA) | 5.58±2.9 | NA | stable medication usage | ON | 8 | NA | 30 |
| Calabrò, Naro ^22^ | 2019 | BGT_ECA: 70±8 TT: 73±8 | BGT_ECA: 25(11) TT: 25(14) | BGT_ECA: 10.0±3.0 TT: 9.3±3.0 | BGT_ECA: 3.0±1.0 TT: 3.0±1.0 | levodopa  BGT_ECA: 450±55 TT: 435±49 | ON | 8 | 5 | 25 |
| Cancela, Mollinedo ^23^ | 2019 | AQE: 67.7±4.6 Mul_C: 69.2±4.4 | AQE: 7(6) Mul_C: 5(3) | NA | AQE: 2.3±0.8 Mul_C: 2.2±0.5 | NA | NA | 8 | 3 | 50 |
| Canning, Allen ^24^ | 2012 | TT: 60.7±5.9  CON: 62.9±9.9 | TT: 10(5) CON: 10(6) | TT: 6.1±4.0  CON: 5.2±4.1 | 1-2 | levodope: 100 to 1200 mg/day; | ON | 6 | 4 | 35 |
| Capato, de Vries ^25^ | 2020 | BGT_ECA: 74±8 BGT: 67±13 CON: 73±10 | BGT_ECA: 56(27) BGT: 50(32) CON: 48(29) | BGT_ECA: 5±5.2 BGT: 6±5.9 CON: 8±9.6 | BGT_ECA: 2.3±0.8 BGT: 2.2±0.8 CON: 2.3±0.7 | Levodopa BGT_ECA: 615±424 BGT: 701±466 CON: 698±389 | ON | 5 | 2 | 45 |
| Capato, Nonnekes ^26^ | 2020 | BGT_ECA: 77±7 BGT: 78±10 | BGT_ECA: 17(9) BGT: 18(12) | BGT_ECA: 17±9 BGT: 11±4 | NA | Levodopa BGT_ECA: 749±381 BGT: 869±327 | ON | 5 | 2 | 45 |
| Capecci, Pournajaf ^27^ | 2019 | RA_GT: 68.1±9.8 TT: 67.0±7.6 | RA_GT: 48(19) TT: 48(24) | RA_GT: 8.9±5.3 TT: 8.9±4.3 | RA_GT: 3±0.5 TT: 3±0.5 | levodopa equivalent daily dose RA_GT: 739.8±328 TT: 739.4±301 dopamine-agonists-LEDD RA_GT 105.3±112 TT: 115.3±96 | ON | 4 | 5 | 45 |
| Capecci, Serpicelli ^28^ | 2014 | BGT: 66.8±4.9 CON: 68.1±5.6 | BGT: 7(4) CON: 7(4) | BGT: 9.5±7.4 CON: 9.6±4.9 | BGT: 3.3±0.7 CON: 3.3±0.9 | stable medication usage | ON | 4 | 3 | 40 |
| Carda, Invernizzi ^29^ | 2012 | RA_GT: 67.8±7.1 TT: 66.9±5.1 | RA_GT: 15(NA) TT: 15(NA) | RA_GT: 3.7±2.5 TT: 3.7±1.9 | RA_GT: 2.2±0.2 TT: 2.2±0.3 | Levodopa RA_GT: 393.8±165.7 TT: 371.4±99.4 | ON | 4 | 3 | 30 |
| Carpinella, Cattaneo ^30^ | 2016 | BGT_ECA: 73.0±7.1 CON: 75.6±8.2 | BGT_ECA: 17(14) CON: 20(9) | BGT_ECA: 7.5±3.2 CON: 10.3±5.7 | BGT_ECA: 12.7±0.7 CON: 2.9±0.5 | NA | ON | 7 | 3 | 45 |
| Carroll, Volpe ^31^ | 2017 | AQE: 69.5±2.96 CON: 74±7.41 | AQE: 10(7) CON: 8(5) | AQE: 7±6.68 CON: 10.5±6.85 | AQE: 2±0.56 CON: 2±0.93 | NA | ON | 6 | 2 | 45 |
| Carvalho, Barbirato ^32^ | 2015 | AE: 64.8±11.9 RT: 64.1±9.9 BGT: 62.1±11.7 | AE: 5(4) RT: 8(6) BGT: 9(5) | AE: 6.6±1.5 RT: 6.0±2.6 BGT: 4.3±2.8 | AE: 2.6±0.5 RT: 2.1±0.6 BGT: 2.3±0.5 | stable medication use | ON | 12 | 2 | 40 |
| Cheng, Yang ^33^ | 2016 | Mul_C: 66.4±7.8 DT_BGT: 65.8±11.5 BGT: 67.3±6.4 | Mul_C: 12(8) DT_BGT: 12(9) BGT: 12(8) | Mul_C: 6.5±2.4 DT_BGT: 6.1±4.1 BGT: 8.1±4.6 | Mul_C: 2.25±0.58 DT_BGT: 2.17±0.62 BGT: 2.21±0.66 | NA | ON | 5 | 2.5 | 30 |
| Cheng, Yang ^34^ | 2017 | TT: 65.8 ± 11.5 CON: 67.3 ± 6.4 | TT: 12(9) CON: 12(8) | TT: 6.1 ± 4.1 CON: 8.1 ± 4.6 | TT: 1.8 ± 0.6 CON: 2.0 ± 0.8 | Daily levodopa dosage TT: 401.7 ± 199.4 CON: 420.8 ± 243.3 | ON | 5 | 2.5 | 40 |
| Cherup, Strand ^35^ | 2020 | Yoga: 69.8±7.3   BGT: 71.4±12.1 | Yoga: 15(10)  BGT: 18(11) | NA | Yoga: 1.7±0.5 BGT: 2±0.8 | NA | ON | 12 | 2 | 45 |
| Cheung, Bhimani ^36^ | 2018 | Yoga: 63.5±8.5 CON: 65.8±6.6 | Yoga: 10(NA) CON: 10(NA) | Yoga: 7±3.5 CON: 8±2.59 | Yoga : 2±0.8 CON: 2±0.8 | stable medication usage | ON | 12 | 2 | 60 |
| Chivers Seymour, Pickering ^37^ | 2018 | Mul_C: 71 ±7.7 CON: 73 ±7.7 | Mul_C: 238(147) CON: 236(119) | Mul_C: 8±6.6 CON: 8±5.8 | Mul_C: 2.59±2.67 CON: 0.86±0.89 | stable medication usage | NA | 26 | 7 | 30 |
| Choi ^38^ | 2016 | TC: 60.8 ± 7.6  CON: 65.5 ± 6.8 | TC: 11 (NA) CON: 9 (NA) | TC: 5.2± 2.7  CON: 5.2± 2.7 | TC: 1.6±0.6  CON: 1.8±0.3 | NA | NA | 12 | 3 | 60 |
| Clarke, Patel ^39^ | 2016 | Mul_D: 70.0±9.1 CON: 70.0±9.3 | Mul_D: 381(240) CON: 381(258) | Mul_D: 4.5±4.9 CON: 4.6±4.5 | Mul_D: 2.3±0.5 CON: 2.3±0.5 | Levodopa equivalent dose Mul_D: 453.0±357.9 CON: 498.0±372.8 | ON | 12 | NA | NA |
| Clerici, Maestri ^40^ | 2019 | Mul_D: 67.0±8.0 AQE: 67.0±11.0 | Mul_D: 27(19) AQE: 25(20) | NA | Mul_D: 2.7±0.4 AQE: 2.7±0.7 | Levodopa equivalent dose Mul_D: 919±407 AQE: 951±328 | ON | 4 | 6 | 60 |
| Combs, Diehl ^41^ | 2013 | Mul_C: 68.0±7.75 AE: 66.5±7 | Mul_C: 14(10) AE: 17(11) | Mul_C: 12.5±5.6 AE: 10.4±9.1 | Mul_C: 2.0±0.75 AE: 2.0±0.75 | NA | ON | 12 | NA | 90 |
| Conradsson, Löfgren ^42^ | 2015 | DT_BGT: 72.9±6.0 CON: 73.6±5.3 | DT_BGT: 47 (28)  CON: 44(23) | DT_BGT: 6.0±5.1 CON: 5.6±5.0 | DT_BGT: 2.6±0.5 CON: 2.6±0.5 | levodopa equivalent dosage DT_BGT: 581±295 CON: 645±404 | ON | 10 | 3 | 60 |
| Corcos, Robichaud ^43^ | 2013 | Mul_C: 58.66±5.6 RT: 59.06±4.6 | Mul_C: 24(14) RT: 24(14) | Mul_C: 6.5±4.7 RT: 6.5±4.1 | Mul_C: 2.3±0.53 RT: 2.2±0.41 | Levodopa: Mul_C: 705±405 RT: 589±355 | OFF/ON | 96 | 2 | NA |
| Costa-Ribeiro, Maux ^44^ | 2016 | Mul_D: 61.1±9.1 BGT_ECA: 62.0±16.7 | Mul_D: 11(8) BGT_ECA: 11(7) | Mul_D: 2.4±0.7 BGT_ECA: 2.3±0.4 | Mul_D: 6.1±3.8 BGT_ECA: 6.3±3.7 | Levodopa Mul_D: 740.9±924.3 BGT_ECA: 890.9±836.0 | ON | 4 | 3 | 43 |
| Cugusi, Solla ^45^ | 2015 | NW: 68.1 ± 8.7 CON: 66.6±7.3 | NW: 10( 8) CON: 10(8) | NW: 7±2 CON: 7±4 | NW: 2.4 ± 0.8 CON: 2.3 ± 0.5 | NA | ON | 12 | 2 | 60 |
| Daneshmandi, Sayyar ^46^ | 2017 | Pilates: 57±6.2 CON: 58.3±7.4 | Pilates: 15(8) CON: 15(10) | Pilates: 7.3±3.8 CON: 8.2±3.1 | Pilates: 2.7±0.5 CON: 2.6±0.5 | stable medication usage | ON | 8 | 3 | 60 |
| Dashtipour, Johnson ^47^ | 2015 | Mul_C: 64±4.2  Mul_D: 62.8±13.9 | Mul_C: 5(NA) Mul_D: 6(NA) | Mul_C: 4.5±3.3 Mul_D: 2.9±1.5 | Mul_C: 1.3±0.5 Mul_D: 1.8±0.5 | stable medication usage | ON | 4 | 4 | 60 |
| David, Robichaud ^48^ | 2015 | Mul_C: 58.6±5.6 RT: 59.0±4.6 | Mul_C: 24(14) RT: 24(14) | Mul_C: 6.5±4.7 RT: 6.5±4.1 | Mul_C: 2.3±0.53 RT: 2.2±0.41 | Mul_C: 705±405 RT: 598±355 | OFF | 96 | 2 | NA |
| David, Robichaud ^49^ | 2016 | Mul_C: 58.6±5.6 RT: 59.0±4.6 | Mul_C: 24(14) RT: 24(14) | Mul_C: 6.5±4.7 RT: 6.5±4.1 | Mul_C: 2.3±0.53 RT: 2.2±0.41 | Mul_C: 705±405 RT: 598±355 | OFF | 96 | 2 | NA |
| de Bruin, Doan ^50^ | 2010 | BGT_ECA: 64.1± 4.2 CON: 67±8.1 | BGT_ECA: 11(6)  CON: 11(5) | BGT_ECA: 6.4± 4.2 CON: 4.5±3.3 | BGT_ECA: 2.3± 0.4 CON: 2.1±0.4 | stable medication usage | ON | 13 | 3 | 30 |
| de Lima, Ferreira-Moraes ^51^ | 2019 | CON: 67.2 ± 5.2 RT: 66.2 ± 5.5 | CON: 16(NA) RT: 17(NA) | NA | CON: 1.93±0.80 RT: 2.07±0.80 | stable medication usage | ON | 20 | 2 | 35 |
| de Melo, Kleiner ^52^ | 2018 | BGT: 65.6±13.0 TT: 61±10.7 VR: 60.3±9.3 | BGT: 12(5) TT: 13(12)  VR: 12(11) | NA | BGT: 2.1±0.9 TT: 1.5±0.7 VR: 1.4±0.5 | stable medication usage | ON | 4 | 3 | 20 |
| Demonceau, Maquet ^53^ | 2017 | AE: 65±8 RT: 67±10 CON: 63.3±6 | AE: 16(12) RT:15(8) CON: 15(10) | AE: 5±4.07 RT: 7±5.08 CON: 5±2.96 | AE: 1.5±1.11 RT: 2±1.11 CON: 1.5±0.74 | levodopa equivalent dose  AE: 402±240.7 RT: 594±517.03 CON: 381±439.25 | ON | 12 | 2-3 | 75 |
| Dibble, Foreman ^54^ | 2015 | RT: 66.00±14.78 Mul_C: 70.71±9.19 | RT: 20(11) Mul_C: 21(14) | RT: 8.00 ±4.48 Mul_C: 5.70 ±4.23 | 2.5±0.5 | stable medication usage | ON/OFF | 12 | 2 | 60 |
| Dibble, Hale ^55^ | 2006 | RT: 64.3 ±9.6 CPP: 67.0±10.2 | RT: 10(NA) CPP: 9(NA) | RT: 6.1±3.9 CPP: 6.5±4.3 | RT: 2.5±0.5 CPP: 2.5±0.7 | stable medication usage | ON | 12 | 3 | 50 |
| DiFrancisco-Donoghue, Lamberg ^56^ | 2012 | Mul_D: 67±6 Mul_C: 68±7 CON: 68±8 | Mul_D: 9(5) Mul_C: 9(7) CON: 9(3) | Mul_D: 7±4 Mul_C: 8±5 CON: 9±6 | 2 | stable medication useage | NA | 6 | 2 | 40 |
| Dipasquale, Meroni ^57^ | 2016 | CPP: 69.9±6.42 CON: 66.4±9.32 | CPP: 20(13) CON: 20(13） | months CPP: 27±7 CON: 28±8 | 2 | NA | ON | 16 | 2 | 60 |
| Droby, Maidan ^58^ | 2020 | VR: 72.8±6.7 TT: 73.6±6.5 | VR: 18(11)  TT: 19(11) | VR: 8.4±6.0 TT: 9.6±6.6 | VR: 2.5±0.4 TT: 2.6±0.4 | levodopa equivalent dose  VR: 770 ±563 TT: 1390±1434 | ON | 6 | 3 | NA |
| Duncan and Earhart ^59^ | 2012 | Tango: 69.3 ±9.96 CON: 69.0 ±7.65 | Tango: 26(15) CON: 6(15) | Tango: 5.8 ± 1.1 CON: 7.0 ± 1.0 | Tango: 2.6 ± 0.1 CON: 2.5 ± 0.1 | NA | OFF | 48 | 2 | 60 |
| Ebersbach, Ebersbach ^60^ | 2010 | Mul_C: 67.1±3.6 NW: 65.5 ±9.0 CON: 69.3 ±8.4 | Mul_C: 20(7) NW: 19(7) CON: 19(8) | Mul_C: 6.1±3.0 NW: 7.8±4.4 CON: 7.4±5.9 | Mul_C: 2.8±0.37 NW: 2.6±0.4 CON: 2.5±0.7 | Levodopa equivalent dose  Mul_C: 486±301 NW: 530±288 CON: 463±260 | ON | 5 | 3 | 60 |
| Ebersbach, Edler ^61^ | 2008 | WBV: 72.5±6.0 DT_BGT: 75.0±6.8 | WBV: 10(7) DT_BGT 11(7) | WBV: 7.0 ± 3.3 DT_BGT: 7.5 ± 2.7 | NA | levodopa WBV: 532.0±226.0 DT_BGT: 600.0±207.0 | ON | 4 | 5 | 150 |
| Ellis, de Goede ^62^ | 2005 | Mul_C: 64±8.4  CON: 63±8.8 | Mul_C: 35(25) CON: 33(26) | NA | Mul_C: 2.5±0.5 CON: 2.4±0.5 | NA | ON | 6 | 2 | 90 |
| El-Tamawy, Darwish ^63^ | 2012 | CPP: 61.4±7.3 Mul_D: 63.2±5.6 | CPP: 15(NA) Mul_D: 15(NA) | CPP: 4.0±0.9 Mul_D: 3.8±0.9 | NA | NA | ON | 8 | 3 | 60 |
| Feng, Li ^64^ | 2019 | VR: 67.47±4.79 CPP: 66.93±4.64 | VR: 14(8) CPP: 14(9) | VR: 7.07±1.44 CPP: 6.60±1.45 | VR: 3.03±0.55 CPP: 2.97±0.58 | levodopa equivalent dose  VR: 203.6±47.2 CPP: 227.5±54.3 | ON | 12 | 5 | 45 |
| Fernandes, Rocha ^65^ | 2015 | BGT: 62.3±12.9 DT_BGT: 63.4±9.5 | BGT: 8(6) DT_BGT: 7(5) | BGT: 7.7±7.5 DT_BGT: 8.8±4.3 | 3 | NA | ON | 6 | 2 | NA |
| Ferraz, Trippo ^66^ | 2018 | BGT: 71±6.67 AE: 67±5.18 VR: 67±1.49 | BGT: 22(16) AE: 20(11) VR: 20(10) | BGT: 4  AE: 6  VR: 4 | BGT: 2.5 AE: 2.5  VR: 2.5 | stable medication usage | ON | 8 | 3 | 50 |
| Ferrazzoli, Ortelli ^67^ | 2018 | CON: 66.9±10.5 Mul_C: 66.5±8.6 | CON: 48(30) Mul_C: 186(106) | CON: 7.4±5.3 Mul_C: 9.0±5.6 | CON: 2.6±0.6 Mul_C: 2.6±0.5 | NA | ON | 4 | 5 | 60 |
| Ferreira, Alves ^68^ | 2018 | CO: 67.6±8.9 RT: 64.1±7.0 | NA | CON: 4.5±4.0 RT: 6.4±2.7 | 1-3 | stable medication use | ON | 24 | 2 | 35 |
| Fietzek, Schroeteler ^69^ | 2014 | BGT_ECA: 69.8±6.5 CON: 64.2±5.9 | BGT_ECA: 14(9) CON: 8(7) | BGT_ECA: 12.1±6.4 CON: 13.3±3.6 | BGT_ECA: 3±0.7 CON: 3±0.7 | Levodopa equivalent dose  BGT_ECA: 664±242.9 CON: 556±195.4 | ON | 2 | 3 | 30 |
| Fil-Balkan, Salci ^70^ | 2018 | Mul_C: 71.83±9.71 CPP: 72.75±9.23 | Mul_C: 12(7) CPP: 12(6) | Mul_C: 6.83±3.78 CPP: 6.91±5.07 | Mul_C: 2.75±0.26 CPP: 2.67±0.25 | stable medication usage | ON | 6 | 2 | 75 |
| Fisher, Wu ^71^ | 2008 | TT: 63.1±11.5 CPP: 61.5±9.8 BWS_TT: 64.0±14.5 | TT: 10(8) CPP: 10(5) BWS_TT: 10(6) | months TT: 17.7±13.3 CPP: 8.8±7.9 BWS_TT: 14.7±9.9 | TT: 1.9±0.3 CPP: 1.9±0.3 BWS_TT: 1.9±0.5 | stable medication use | ON | 8 | 3 | 45 |
| Franzoni, Monteiro ^72^ | 2018 | NW: 64.4±8.7 AE: 71.1±6.2 | NW: 14(NA) AE: 11(NA) | NA | NW: 1.6±0.6 AE: 2.0±1.1 | stable medication usage | ON | 9 | 4 | NA |
| Frazzitta, Bertotti ^73^ | 2012 | CPP: 72±7 CON: 70±7 | CPP: 25(11) CON: 25(13) | CPP: 8±3 CON: 9±3 | 3 | levodopa CPP: 602±268 CON: 647±245 | ON | 4 | 15 | NA |
| Frazzitta, Bossio ^74^ | 2015 | BGT_ECA: 66.6±10.0  AE: 65.0±8.8 | BGT_ECA: 30(13) AE: 30(17) | NA | BGT_ECA: 2.8 ±0.4 AE: 2.8±0.4 | dopaminergic replacement therapy BGT_ECA:608.7 ±307.6 AE: 740.9 ±297.8 | NA | 4 | 6 | 35 |
| Frazzitta, Maestri ^75^ | 2009 | TT: 71±8 BGT_ECA: 71±7 | TT: 20(8) BGT_ECA: 20(9) | TT: 13.2±4.1 BGT_ECA: 12.9±4.6 | 3 | TT: 685±246 BGT_ECA: 720±232 | NA | 4 | 7 | 20 |
| Frazzitta, Maestri ^76^ | 2014 | CPP: 67±5  CON: 65±4 | CPP: 14(NA) CON: 10(NA) | CPP: 8±5 CON: 8±2 | range: 1-1.5 | stable medication useage | NA | 4 | 15 | 60 |
| Furnari, Calabrò ^77^ | 2017 | RA_GT: 71.5 ±11.7  BGT: 77.7 ±8.3 | RA_GT:19(11) BGT:19(10) | NA | RA_GT: 3.1±0.9  BGT: 2.2±0.5 | stable medication useage | ON | 4 | 6 | 60 |
| Galli, Cimolin ^78^ | 2016 | RA_GT: 68.8 ±6.9  TT: 66.4±9.7 | RA_GT: 25(14) TT: 25(12) | RA_GT: 9.9 TT: 8.1 | NA | Levodopa equivalent dose RA_GT: 650.8±176.2 TT: 781.8±321.2 | ON | 4 | 5 | 45 |
| Gandolfi, Geroin ^79^ | 2017 | VR: 67.5±7.2 BGT: 69.8±9.4 | VR: 38(23) BGT: 38(28) | VR: 6.2±3.8 BGT: 7.5±3.9 | 2.5 | NA | ON | 7 | 3 | 50 |
| Gandolfi, Tinazzi ^80^ | 2019 | Mul_C: 72.42±6.4 CPP: 70.72±6.6 | Mul_C: 19(9) CPP: 18(15) | Mul_C: 8.01±5.9 CPP: 6.57±4.29 | ≤4 | Levodopa equivalent daily dose Mul_C: 803.31 ±405.81 CPP: 623.44±447.38 | ON | 4 | 5 | 60 |
| Ganesan, Sathyaprabha ^81^ | 2015 | 58.15 ± 8.7 | BWS_TT:20 (NA) BGT_ECA: 20 (NA) CON: 20 (NA) | NA | >3 | stable medication useage | ON | 4 | 4 | 30 |
| Gao, Leung ^82^ | 2014 | TC: 69.5±7.3 CON: 68.3±8.5 | TC: 37(23) CON: 39(27) | TC: 9.2±8.6 CON: 8.4±8.2 | TC: 2.4±0.5 CON: 2.4±0.7 | stable medication usage | ON | 12 | 3 | 60 |
| Gaßner, Steib ^83^ | 2019 | DT_BGT: 67.6±8.2 TT: 62.5±7.9 | DT_BGT: 18(11) TT: 20(16) | DT_BGT: 7.9±4.0 TT: 7.3±4.4 | DT_BGT: 1.9±0.2 TT: 1.9±0.2 | levodopa equivalent dose DT_BGT: 630.4±331.1 TT: 645.7±280.8 | ON | 8 | 2 | 30 |
| Ghielen, van Wegen ^84^ | 2017 | CPP: 66.6 ±8.4 Mul_D: 59.6±9.7 | CPP: 19(9) Mul_D: 19(7) | CPP: 12.3±4.3 Mul_D: 10.5±5.7 | 2-3 | CPP: 1340±534 Mul_D 1165±405 | ON | 6 | 2 | 60 |
| Ginis, Nieuwboer ^85^ | 2016 | NA | BGT_ECA: 20(NA) BGT: 18(NA) | NA | 2-3 | NA | ON | 6 | 3 | 30 |
| Gobbi, Pelicioni ^86^ | 2020 | CPP: 69.2±8.2  BGT: 67.8±9.1 CON: 69.5±7.6 | CPP: 57(31) BGT: 48(28) CON: 47(19) | CPP: 8.0±5.7 BGT: 5.0±3.0 CON: 5.9±3.2 | CPP 1.9±0.6 BGT: 1.8±0.6 CON: 1.7±0.5 | Levodopa equivalent dose  CPP: 557.9±413.3 BGT: 552.3±385.2 CON: 605.8±376.1 | ON | 32 | 2 | 60 |
| Goodwin, Richards ^87^ | 2014 | Mul_C: 72.0±8.6 CON: 70.1±8.3 | Mul_C: 64(39) CON: 66(35) | Mul_C: 9.1±6.4 CON 8.2±6.4 | Mul_C: 2.6 ±0.9 CON: 2.4 ±0.9 | stable medication useage | NA | 10 | 1 | 60 |
| Grobbelaar, Venter ^88^ | 2017 | BGT: 70 ± 11 DT_BGT: 72 ± 6 | BGT: 14(10) DT_BGT: 15(9) | BGT: 7 ± 6  DT_BGT: 5 ± 3 | BGT: 2.7 ± 0.5  DT_BGT: 2.7 ± 0.9 | stable medication usage | NA | 8 | 3 | 50 |
| Hackney and Earhart ^89^ | 2009 | Tango: 68.2±1.4  CON: 66.5±2.8 Dance: 66.8±2.4 | Tango: 14(11)  CON: 17(12)  Dance 17(11) | Tango: 6.9±1.3  CON: 5.9±1.0 Dance: 9.2±1.5 | Tango: 2.1±0.1  CON: 2.2±0.2 Dance: 2.0±0.2 | stable medication useage | ON | 13 | 2 | NA |
| Hackney and Earhart ^90^ | 2008 | TC: 64.9± 8.3 CON: 62.6±10.2 | TC: 17(11) CON: 15(10) | TC: 8.7±4.7 CON: 5.5±3.3 | TC: 2±0.4 CON: 1.9± 0.2 | stable medication usage | ON | 13 | 2 | 60 |
| Hackney and Earhart ^91^ | 2009 | Dance: 66.8±9.9  Tango: 68.2±5.2TC: 64.9±8.3 CON: 66.8±11.5 | Dance: 17(11)  Tango: 14(11) TC: 13(11) CON: 17(12) | Dance: 9.2±5.8  Tango: 6.9±4.9 TC: 8.7±4.7  CON: 5.9±4.1 | Dance: 2.0±0.8  Tango: 2.1±0.4 TC: 2.0±0.4 CON: 2.2±0.8 | stable medication usage | ON | 13 | 2 | 60 |
| Hackney, Kantorovich ^92^ | 2007 | Tango: 72.6±2.2 RT: 69.6±2.1 | Tango: 9(6) RT: 10(6) | Tango: 6.2±1.5 RT: 3.3±0.5 | Tango: 2.3±0.7 RT: 2.2±0.6 | NA | ON | 13 | 2 | 60 |
| Harro, Shoemaker ^93^ | 2014 | BGT_ECA: 67.3±10.9 TT: 64.9±9.0 | BGT_ECA: 10(8) TT: 10(5) | BGT_ECA: 4.0±2.1 TT: 4.3±2.3 | BGT_ECA: 1.9±0.5 TT: 2.0±0.6 | NA | ON | 6 | 3 | 30 |
| Hashimoto, Takabatake ^94^ | 2015 | Dance: 67.9±7.0 CPP: 62.7±14.9 CON: 69.7±4.0 | Dance: 15(3) CPP: 17(2) CON: 14(7) | Dance: 6.3±4.6 CPP: 7.8±6.2 CON: 6.9±4.0 | Dance: 2.7±0.46 CPP: 2.7±0.47 CON: 3±0.56 | stable medication useage | ON | 12 | 1 | 60 |
| Hass, Collins ^95^ | 2007 | RT: 62.8±2.6 Mul_D: 62.2±2.6 | RT: 10(9) Mul_D: 10(8) | months RT: 59.0±14.8 Mul_D: 47.8±8.3 | RT: 2.2±0.2 Mul_D: 2.1±0.2 | stable medication useage | ON | 12 | 2 | NA |
| Helgerud, Thomsen ^96^ | 2020 | PT: 72±8.0 CPP: 62.0±11 | PT: 15(7) CPP: 7(2) | PT: 8.8±4.9 CPP: 7.3±2.5 | PT: 2.3±0.1 CPP: 2.7±0.7 | Levodopa equivalent dose  PT: 693.0±473.0 CPP: 692.0±322.0 | ON | 4 | 3 | 60 |
| Hirsch, Toole ^97^ | 2003 | BGT: 75.7±1.8 Mul_C: 70.8±2.8 | BGT: 9(NA) Mul_C: 6(NA) | BGT: 8.3±9.8 Mul_C: 5.5±3.91 | BGT: 1.9±0.6 Mul_C: 1.8±0.3 | stable medication usage | ON | 10 | 3 | 40 |
| Hubble, Naughton ^98^ | 2017 | Mul_C: 67.5±5.8 CON: 63.3±4.9 | Mul_C: 11(8) CON: 11(7) | Mul_C: 7.0 ±5.0 CON: 6.5±5.2 | Mul_C: 2.0±0.7  CON: 1.8±0.6 | Levodopa Daily Equivalent Dose (mg) Mul_C: 868.2±475.7 CON: 564.8±327.6 | ON | 12 | 1 | 90 |
| Johansson, Cameron ^99^ | 2021 | AE: 68.7±6.9 Stretch: 67.5±9.4 | AE: 26 (20) Stretch: 31 (17) | AE: 3.7±3.2 Stretch: 4.0±2.7 | NA | stable medication usage | OFF | 24 | 3 | 45 |
| Johansson, Freidle ^100^ | 2020 | DT_BGT: 72±13.3 CON: 67.5±5.2 | DT_BGT: 7(6)  CON: 6(3) | DT_BGT: 10±7.4 CON: 7±5.9 | DT_BGT: 2±NA CON: 2.5±NA | levodopa equivalent dose  DT_BGT: 700±400 CON: 765.5±478.5 | ON | 10 | 2 | 60 |
| Joseph, Brodin ^101^ | 2018 | DT_BGT: 73.1±5.8  CON: 73.0±5.5 | DT_BGT: 51(32) CON: 49(25) | DT_BGT: 5.9±5.1 CON: 5.6±4.8 | DT_BGT: 2.55±0.5 CON: 2.57±0.5 | levodopa equivalent dose DT_BGT: 578±299 CON: 640±380 | ON | 10 | 3 | 60 |
| Jung, Hasegawa ^102^ | 2020 | Mul_C: 67.7±6.7 CON: 70.0±8.2 | Mul_C: 44(30) CON: 42(28) | Mul_C: 6.2±4.4 CON: 6.7±5.5 | Mul_C: 2.11±0.44 CON: 2.42±0.76 | NA | OFF | 6 | 3 | 80 |
| Kadivar, Corcos ^103^ | 2011 | BGT_ECA: 73.3±2.2 BGT: 70.5±2.2 | BGT_ECA: 8(5) BGT: 8(6) | BGT_ECA: 8.9±1.8 BGT: 7.5±1.2 | BGT_ECA: 2.69±0.56 BGT: 2.69±0.56 | levodopa BGT_ECA: 456.25±317.8 BGT: 509.4±207.0 | ON | 6 | 3 | 50 |
| Keus, Bloem ^104^ | 2007 | CPP: 65.4±6.74 CON: 70.5±13.56 | CPP: 14(11) CON: 13(11) | CPP: 7 ±6.44 CON: 6±4.22 | CPP: 2.35±0.84 CON: 2.38±0.86 | stable medication usage | ON | 10 | 1.5 | 45 |
| Khalil, Busse ^105^ | 2017 | Mul_C: 58.4±13.5 CON: 60.7±15.4 | Mul_C: 16(12) CON: 14(7) | Mul_C: 8.0±6.4 CON: 7.5±4.0 | Mul_C: 2.4±0.72 CON: 2.2±0.8 | NA | ON | 8 | 3 | 45 |
| Khuzema, Brammatha ^106^ | 2020 | TC: 72±5.22 Yoga: 68.11± 4.23 CON: 70.89±6.01 | TC: 9(6) Yoga: 9(6) CON: 9(7) | TC: 5.67±2.33 Yoga: 6.2±1.67 CON: 5.23±3.12 | TC: 2.83±0.24 Yoga: 2.83±0.24 CON: 2.78±0.25 | NA | ON | 8 | 5 | 35 |
| Kim, Kim ^107^ | 2022 | RA_GT: 68.7±6.9 TT: 67.5±9.3 | RA_GT: 22 (6) TT: 22 (7) | RA_GT: 9.3±5.8 TT: 8.7±4.5 | RA_GT: 2.4±0.2 TT: 2.5±0.3 | stable medication useage | ON | 4 | 3 | 45 |
| King, Salarian ^108^ | 2013 | Mul_C:65.7 ± 8.3 TT: 65.1 ± 7.3 | Mul_C: 20(12) TT: 19(13) | NA | Mul_C: 2.5 ± 0.8 TT: 2.4 ± 0.6 | NA | ON | 4 | 4 | 75 |
| Kunkel, Fitton ^109^ | 2017 | Dance: 71.3±7.7 CON: 69.7±6.0 | Dance: 36(19) CON: 15(6) | Dance: 4.7±3.5 CON: 7.0±4.9 | Dance: 2.11±0.84 CON: 2.13±0.72 | Dance: 5.8±2.7 CON: 4.7±3.0 | ON | 10 | 2 | 60 |
| Kurt, Büyükturan ^110^ | 2017 | AQE: 62.41 ± 6.76 Mul_C: 63.61 ± 7.18 | AQE: 20(11) Mul_C: 20(13) | NA | AQE: 2.37±0.39 Mul_C: 2.32±0.40 | Levodopa equivalent dose AQE: 764.36 ± 96.09 Mul_C: 742.67 ± 113.64 | ON | 5 | 5 | 60 |
| Kurtais, Kutlay ^111^ | 2008 | TT: 63.8±10.6 CON: 65.7±5.3 | TT: 12(5) CON: 12(7) | TT: 5.3±0.8 CON: 5.4±1.2 | TT: 2.5±0.7 CON: 2.2±0.8 | NA | ON | 6 | 3 | 40 |
| Kwok, Kwan ^112^ | 2019 | Yoga: 63.7±8.2  CON: 63.5±9.3 | Yoga: 71(37)  CON: 67( 28) | NA | Yoga: 2.68 ± 0.47  CON: 2.66 ±0.54 | levodopa equivalent dose Yoga: 2685±7870.6  CON: 2541.1 ±6442.0 | NA | 8 | 3 | 85 |
| Landers, Hatlevig ^113^ | 2015 | BGT_ECA: 72.2±4.4 BGT_ICA: 70.2±4.4 BGT: 70.1± 9.5 CON: 74.3±8.8 | BGT_ECA: 10(4) BGT_ICA: 11(8) BGT: 10(7) CON: 10(6) | NA | BGT_ECA: 2.25±0.86 BGT_ICA: 2.75±0.75 BGT: 2.45±0.44 CON: 2.75±0.63 | NA | ON | 4 | 3 | 45 |
| Leal, Abrahin ^114^ | 2019 | CON: 64.9±2.32  RT: 65.2±2.05 | CON: 27(13)  RT: 27(14) | NA | CON: 2±0.5  RT: 2±0.5 | stable medication usage | ON | 24 | 2 | 32.5 |
| Lee, Kim ^115^ | 2017 | Qigong: 65.8±7.2 CON: 65.7±6.4 | Qigong: 25(10) CON: 16(7) | Qigong: 4.5±3.3 CON: 4.4±3.0 | Qigong: 2.0±0.7 CON: 1.8±0.8 | NA | ON | 8 | 2 | 60 |
| Lei, Ma ^116^ | 2021 | CPP: 58.3±5.5 WBV: 56.8±3.4 | CPP: 40(24) WBV: 40(22) | CPP: 5.53±2.11 WBV: 5.26±1.58 | 2-4 | NA | NA | 9 | 6 | 40 |
| Li, Harmer ^117^ | 2013 | TC: 68±9 RT: 69±8 Stretch: 69±9 | TC: 65 (20) RT: 65 (27) Stretch: 65 (26) | TC: 8±9 RT: 8±9 Stretch: 6±5 | TC: 2.2-2.87 RT: 2.2-2.84 Stretch: 2.3-3.05 | stable medication usage | ON | 24 | 2 | 60 |
| Liao, Yang ^118^ | 2015 | Mul_C: 64.6 ± 8.6 TT: 65.1 ± 6.7 VR: 67.3 ± 7.1 | Mul_C: 12(5) TT: 12(6) VR: 12(6) | Mul_C: 6.4 ± 3.0 TT: 6.9 ± 2.8 VR: 7.9 ± 2.7 | Mul_C: 1.9 ± 0.8 TT: 2.0 ± 0.8 VR: 2.0 ± 0.7 | stable medication usage | ON | 6 | 2 | 60 |
| Liu, Yan ^119^ | 2020 | DT_BGT: 63.90±5.82 VR: 60.9±7.20 | DT_BGT: 21(12) VR: 21(11) | DT_BGT: 8.10±3.42 VR: 7.76±3.99 | 3 | NA | NA | 4 | 5 | 30 |
| Löfgren, Conradsson ^120^ | 2019 | DT_BGT: 72.5±5.8 CON: 73.5±5.6 | DT_BGT: 45(27) CON: 42(21) | DT_BGT: 5.8±5.3 CON: 5.4±4.7 | DT_BGT: 2.5 ± 0.4 CON: 2.4 ± 0.4 | Levodopa equivalent doseDT_BGT: 591.8±287.1 CON: 639.5±422.2 | ON | 10 | 3 | 60 |
| Maidan, Nieuwhof ^121^ | 2018 | TT: 73.1 ± 1.1 VR: 70.1 ± 1.3 | TT: 34(23) VR: 30(22) | TT: 9.7 ± 1.0 VR: 8.9 ± 1.1 | 2-3 | levodopa equivalent dose TT: 1186 ± 238 VR: 833 ± 102 | ON | 6 | NA | 45 |
| Marumoto, Yokoyama ^122^ | 2019 | CPP: 69.0±5.9 Mul_D: 68.2±8.9 | CPP: 40(NA) Mul_D: 40(NA) | CPP: 10.6±5.7 Mul_D: 9.4±5.1 | CPP: 3.0±0.7 Mul_D: 3.1±0.8 | Levodopa equivalent dose CPP: 634±366.2 Mul_D: 663=2±421.9 | ON | 8 | 6 | 40 |
| McNeely, Mai ^123^ | 2015 | Dance: 68.25 ± 10.90 Tango: 67.66 ± 8.62 | Dance: 8(4) Tango: 8(4) | Dance: 10.06 ± 4.14 Tango: 5.38 ± 4.83 | Dance: 2.25 ± 0.27 Tango: 2.13 ± 0.58 | NA | OFF | 12 | 2 | 50 |
| Medijainen, Pääsuke ^124^ | 2019 | CPP: 77.1±4.2 CON: 69.9±5.1 | CPP: 12(5) CON: 12(5) | CPP: 8.0±6.9 CON: 7.7±5.4 | CPP: 2.2±0.5 CON: 2.3±0.7 | stable medication usage | ON | 8 | 2 | 60 |
| Michels, Dubaz ^125^ | 2018 | Tango: 66.44±NA CON: 75.50±NA | Tango: 9(NA) CON: 4(NA) | NA | Tango: 2.11±0.33 CON: 2.50±1.00 | stable medication usage | ON | 10 | 2 | 60 |
| Miyai, Fujimoto ^126^ | 2002 | BWS_TT: 69.5±6.3  BGT: 69.8±4.5 | BWS_TT: 11(5) BGT: 9(5) | BWS_TT: 4.1±2.7 BGT: 4.5±2.1 | BWS_TT: 2.9±0.3 BGT: 2.8±0.3 | Levodopa equivalent dose BWS_TT: 241.0±97.8  BGT: 255.6±68.4 | ON | 4 | 3 | 45 |
| Mollinedo-Cardalda, Cancela-Carral ^127^ | 2018 | Pilates: 62.85 ± 9.75 CON: 66.00 ± 13.14 | Pilates: 13(5) CON: 13(4) | Pilates: 5.77 ± 3.39 CON: 5.69 ± 4.40 | Pilates: 2.08 ± 0.49 CON: 2.00 ± 0.82 | stable medication usage | ON | 12 | 2 | 60 |
| Monticone, Ambrosini ^128^ | 2015 | Mul_D: 74.1±6.0 CPP: 73.4±7.0 | Mul_D: 35(24) CPP: 35(22) | Mul_D: 15.7±2.6 CPP: 15.3±3.0 | Mul_D: 3.08±0.50 CPP: 3.07±0.47 | Levodopa equivalent dose Mul_D: 928.7±86.7 CPP: 937.6±91.1 | ON | 8 | 2 | 90 |
| Moon, Sarmento ^129^ | 2020 | Qigong: 66.4± 8.1 CON: 65.9± 5.4 | Qigong: 8(4) CON: 9(6) | Qigong: 4.25 ±2.1 CON: 5.33± 3.3 | Qigong: 2±0 CON: 2±0 | levodopa equivalent dose Qigong: 682.6±301.1 CON: 712.6±332.4 | NA | 12 | 7 | 20 |
| Moon, Schmidt ^130^ | 2017 | Qigong: 61.8 ± 5.7 CON: 68.0 ± 5.3 | Qigong: 5(NA) CON: 5(NA) | NA | NA | stable medication usage | NA | 6 | 12 | 20 |
| Morris, Iansek ^131^ | 2009 | BGT_ECA: 72.5±5.8 Mul_C: 73.5±5.7 | BGT_ECA: 14(NA) Mul_C: 14(NA) | NA | NA | stable medication usage | ON | 2 | 8 | 40 |
| Morris, Menz ^132^ | 2015 | RT: 67.4±10.4 BGT_ECA: 68.4±9.9 CON: 67.9±8.4 | RT: 70(42) BGT_ECA 69(46) CON: 71(52) | RT: 7.2±6.2 BGT_ECA: 6±5.5 CON: .9±5.2 | RT: 2.39±0.77 BGT_ECA: 2.40±0.81 CON: 2.61±0.90 | stable medication usage | ON | 8 | 1 | 120 |
| Morris, Taylor ^133^ | 2017 | Mul_D: 71±8 CPP: 71±10 | Mul_D: 67(22) CPP: 66(31) | NA | Mul_D: 2±0.25 CPP: 2±0.25 | stable medication usage | ON | 6 | 2 | 60 |
| Morrone, Miccinilli ^134^ | 2016 | Mul_D: 75.0±2.6 CPP: 70.0±3.9 | Mul_D: 10(6) CPP: 10(6) | Mul_D: 6.3±3.3 CPP: 6.5±3.5 | Mul_D: 3.0±0 CPP: 3.0±0.7 | stable medication usage | ON | 10 | 3 | 45 |
| Myers, Harrison ^135^ | 2020 | CON: 65.0±8.7 Yoga: 70.5±8.7 | CON: 13(8) Yoga: 13(7) | NA | A:2(2-3) B:2(2-3) | stable medication usage | NA | 12 | 2 | 60 |
| Nadeau, Pourcher ^136^ | 2014 | Mul_C: 64.3±5.6 TT: 64.0±6.6 BGT: 60.1±6.8 | Mul_C: 9(2) TT: 8(4) BGT: 10(1) | NA | Mul_C: 1.86±0.23 TT: 1.92±0.20 BGT: 1.95±0.15 | NA | ON | 24 | 3 | 60 |
| Ni, Mooney ^137^ | 2015 | Yoga: 71.2±6.5 CON: 74.9± 8.3 | Yoga: 15(11) CON: 12(6) | Yoga: 6.9±6.3 CON: 5.9±6.2 | Yoga: 2.2±0.7 CON: 2.1±0.7 | stable medication usage | ON | 12 | 2 | 60 |
| Ni, Signorile ^138^ | 2016 | PT: 71.6±6.6 CON: 74.9±8.3 | PT: 14(9) CON: 10(4) | PT: 6.6±4.4 CON: 5.9±6.2 | PT: 2.2±0.6 CON: 2.1±0.7 | stable medication usage | ON | 12 | 2 | 60 |
| Ni, Signorile ^139^ | 2016 | PT: 71.6±6.6 Yoga: 71.2±6.5 CON: 74.9± 8.3 | PT: 14(9) Yoga: 13(11) CON: 10(4) | PT: 6.6±4.4 Yoga: 6.9±6.3 CON: 5.9±6.2 | PT: 2.2±0.6 Yoga: 2.2±0.7 CON: 2.1± 0.7 | stable medication usage | ON | 12 | 2 | 60 |
| Nieuwboer, Kwakkel ^140^ | 2014 | BGT_ECA: 67.5±7.8 CON: 69±7.8 | BGT_ECA: 76(48) CON: 77(40) | BGT_ECA: 7±5.2 CON: 8±5.9 | BGT_ECA: 2.6±0.7 CON: 2.7±0.7 | Levodopa equivalent dose  BGT_ECA: 500±296.3 CON: 350±259.3 | ON | 3 | 3 | 30 |
| Nocera, Amano ^141^ | 2013 | TC: 66±11 CON: 65±7 | TC: 15(7) CON: 6(4) | TC: 8.1±5.4 CON: 6.8±1.8 | TC: 2–3 CON: 2–3 | stable medication usage | ON | 16 | 3 | 60 |
| Oliveira, Iraci ^142^ | 2020 | AQE: 65.5±2.2 Mul_D: 68.3±0.4 | AQE: 9(8) Mul_D: 10(9) | NA | AQE: 1.7±0.8 Mul_D: 2.0±1.0 | stable medication usage | ON | 4 | 2 | 60 |
| Ortiz-Rubio, Cabrera-Martos ^143^ | 2017 | RT: 74.2±5.8 CON: 75.4±6.5 | RT: 23(NA) CON: 23(NA) | RT: 4.0±2.2 CON: 4.3±2.0 | RT: 2.5±0.5 CON: 2.4±0.5 | Levodopa:  RT: 683.6±316.2 CON: 743.1±256.8 | ON | 8 | 2 | 60 |
| Palamara, Gotti ^144^ | 2017 | AQE: 70.9±5.7 Mul_C: 70.8±5.3 | AQE: 17(9) Mul_C: 17(11) | NA | AQE: 2.8±0.5 Mul_C: 3.1±0.2 | Levodopa AQE: 583.2±235.3 Mul_C: 720.4±269.9 | ON | 4 | 4 | 60 |
| Paolucci, Zangrando ^145^ | 2017 | Mul_C: 67.0±8.1 Mul_D: 66.0±13.7 | Mul_C: 17(9) Mul_C: 17(10) | 3.0±1.2 | 1.5±0.8 | NA | ON | 5 | 2 | 60 |
| Park, Zid ^146^ | 2013 | RT: 60.1±6.6 CON: 59.8±6.3 | RT: 15(10) CON: 16(10) | NA | NA | NA | NA | 48 | 3 | 60 |
| Paul, Canning ^147^ | 2014 | PT: 68.1±5.6 CON: 64.5±7.4 | PT: 20(13) CON: 20(12) | PT: 7.8±5.2 CON: 7.8±5.9 | PT: 2.0±0.7 CON: 1.9±0.9 | NA | ON | 12 | 2 | 45 |
| Pazzaglia, Imbimbo ^148^ | 2020 | VR: 72±7 CPP: 70±10 | VR: 25(18) CPP: 26(17) | months VR: 89±92 CPP: 57±53 | NA | NA | NA | 6 | 3 | 40 |
| Pelosin, Cerulli ^149^ | 2019 | TT: 71.9±4.1 VR: 73.2±3.6 | TT: 22(7) VR: 17(6) | TT: 9.8±4.7 VR: 11.1±4.2 | NA | levodopa equivalent dose TT: 819.8±318.2 VR: 796.5±341.5 | ON | 6 | 3 | 45 |
| Pérez de la Cruz ^150^ | 2017 | AQE: 66.8±5.3 CPP: 67.5 ±9.9 | AQE: 15(6) CPP: 15(7) | AQE: 6.2±2.5 CPP: 6.7 ±3.2 | AQE: 2.8±0.2 CPP: 2.7±1.0 | NA | OFF | 10 | 2 | 35 |
| Pérez-de la Cruz ^151^ | 2017 | AQE: 65.87±7.09 CPP: 66.44 ± 5.73 | AQE: 14(5) CPP: 15(7) | > 2 years | NA | Non have | OFF | 11 | 2 | 45 |
| Pérez-de la Cruz ^152^ | 2019 | AQE: 64.4±5.2 Mul_C: 65.8±8.9 | AQE: 15(NA) Mul_C: 15(NA) | AQE: 7.1±2.0 Mul_C: 7.7±3.0 | AQE: 2.8±0.2 Mul_C: 2.8±1.0 | NA | OFF | 10 | 2 | 45 |
| Picelli, Melotti ^153^ | 2012 | 68.3 | RA_GT: 16(NA) Stretch: 15(NA) | 7.5 | 3.45 | NA | ON | 4 | 3 | 40 |
| Picelli, Melotti ^154^ | 2013 | RA_GT: 68.50±10.10 TT: 68.80±7.72 BGT: 67.55±7.08 | RA_GT: 20(9) TT: 20(6) BGT: 20(8) | RA_GT: 6.52±5.30 TT: 6.99±6.17 BGT: 6.79±6.30 | 3 | NA | ON | 4 | 3 | 45 |
| Picelli, Melotti ^155^ | 2015 | RA_GT: 68.2±9.2 DT_BGT: 69.7±7.2 | RA_GT: 33(26) DT_BGT: 33(22) | RA_GT: 7.5±5.6 DT_BGT: 8.3±4.1 | NA | NA | ON | 4 | 3 | 45 |
| Picelli, Varalta ^156^ | 2016 | TT: 71.2±9.2 CON: 71.6±7.2 | TT: 9(5) CON: 8(4) | TT: 11.2±5.6 CON: 10.8±4.1 | NA | NA | ON | 4 | 3 | 45 |
| Pohl, Dizdar ^157^ | 2013 | 68.2±5.1 | Mul_D: 12(NA) CON: 6(NA) | 8.8±3.8 | 2.4±0.7 | stable medication usage | ON | 6 | 2 | 60 |
| Pohl, Wressle ^158^ | 2020 | BGT_ECA: 69.7 ± 7.0 CON: 70.4 ± 6.0 | BGT_ECA: 26(19) CON: 20(13) | BGT_ECA 6.0 ± 4.4 CON: 6.8 ± 3.6 | BGT_ECA: 2.4±0.69 CON: 2.3±0.65 | levodopa equivalent dosage BGT_ECA: 727.7±327.3 CON: 690.0±231.0 | ON | 12 | 2 | 60 |
| Poier, Rodrigues Recchia ^159^ | 2019 | Tango: 68.50±8.07 TC: 68.87±10.96 | Tango: 14(9) TC: 15(3) | Tango: 7.93±3.05 TC: 6.28±3.67 | NA | NA | NA | 10 | 1 | 60 |
| Poliakoff, Galpin ^160^ | 2013 | Mul_C: 68.8±7.3 CON: 66.6±7.3 | Mul_C: 12(9) CON: 10(8) | Mul_C: 7.9±3.0 CON: 4.6±3.9 | NA | stable medication useage | ON | 10 | 1 | 60 |
| Pompeu, Mendes ^161^ | 2012 | 67.4±8.1 | VR: 16(NA)  BGT: 16(NA)  (Men: 17) | NA | 1.7±0.5 | stable medication useage | ON | 7 | 2 | 60 |
| Prodoehl, Rafferty ^162^ | 2014 | CPP: 58.6±5.6 RT: 59.0±4.6 | CPP: 24 (14) RT: 24(14) | CPP: 6.5±4.7 RT: 6.5±4.1 | CPP: 1.9±0.3 RT: 2.0±0.4 | Levodopa equivalent dose CPP: 705±405 RT: 598±355 | ON/OFF | 96 | 2 | 75 |
| Protas, Mitchell ^163^ | 2005 | BGT: 71.3±7.4 CON: 73.7 ±8.5 | BGT: 9 (9) CON: 9 (9) | BGT: 7.1±5.1 CON: 8.1±4.4 | BGT: 2.8±0.35 CON: 2.9±0.17 | stable medication usage | ON | 8 | 3 | 60 |
| Qutubuddin, Cifu ^164^ | 2007 | CON 71.89±8.46 BGT: 73.67±5.13 | CON: 9(NA) BGT: 6(NA) | NA | NA | stable medication usage | ON | 4 | 2 | 30 |
| Rafferty, Prodoehl ^165^ | 2017 | RT: 59.0±4.6 CPP: 58.6±5.6 CON: 61.2±7.7 | RT: 24(14) CPP: 24(14) CON: 23(12) | NA | NA | stable medication usage | ON/OFF | 96 | 1.5 | 75 |
| Rennie, Opheim ^166^ | 2020 | DT_BGT: 73.1±5.8 CON: 73.0±5.5 | DT_BGT: 51(32) CON: 49(25) | DT_BGT: 5.9±5.1 CON: 5.6±4.8 | DT_BGT: 2.5±0.5 CON: 2.6±0.5 | DT_BGT: 578±299 CON: 640±380 | ON | 10 | 3 | 60 |
| Ribas, Alves da Silva ^167^ | 2017 | VR: 61.7±6.8 RT: 60.2±11.2 | VR: 10 (4) RT: 10 (4) | VR: 6.5±4 RT: 7±2.8 | VR: 1.4±0.5 RT: 1.5±0.5 | stable medication usage | ON | 12 | 2 | 30 |
| Ridgel and Ault ^168^ | 2019 | AE: 69.9±7.4  Stretch: 70.0±6.4 | AE: 8(4)  Stretch: 8(5) | AE: 4.5±1.6  Stretch: 6.4±2.6 | AE: 1.4±0.5  Stretch: 1.9±0.4 | stable medication usage | ON | 2 | 3 | 40 |
| Rios Romenets, Anang ^169^ | 2015 | CON: 64.3±8.1 Tango: 63.2±9.9 | CON: 15(7) Tango: 18(12) | CON 7.7±4.6 Tango: 5.5±4.4 | CON: 2.0±0.5 Tango: 1.7±0.6 | Levodopa: CON: 485±347.5 Tango: 450±349.7 | ON | 12 | 2 | 60 |
| Rocha, Aguiar ^170^ | 2017 | Tango: 70.2±5.5 Dance: 72.9±5.5 | Tango: 10(NA) Dance: 11(NA) | Tango: 7.2±4.9 Dance: 8.4±5.2 | Tango: 2.5±0.5 Dance: 2.5±0.62 | NA | ON | 8 | 1 | 60 |
| Sacheli, Neva ^171^ | 2019 | AE: 66.76±5.98 Stretch: 67.85±8.50 | AE: 20(13) Stretch: 15(9) | AE: 3.91±2.85  Stretch: 5.17±4.26 | 1-3 | stable medication usage | OFF | 12 | 3 | 40-60 |
| Sage and Almeida ^172^ | 2009 | BGT_ECA: 64.2±10.3 AE: 65.1±9.3 CON: 68.6±8.7 | BGT_ECA: 18(12) AE: 13(6) CON: 15(7) | BGT_ECA: 4.7±4.9 AE: 3.2±2.9  CON: 2.5±2.2 | NA | NA | ON | 12 | 3 | 50 |
| Sale, De Pandis ^173^ | 2013 | RA_GT: 70.3±9.8 DT_BGT: 68.4±9.4 | RA_GT: 10(6) DT_BGT: 10(5) | RA_GT: 8.4±5.0 DT_BGT: 8.7±4.7 | NA | stable medication usage | ON | 4 | 5 | 40 |
| San Martín Valenzuela, Moscardó ^174^ | 2020 | BGT: 64.8±8.8 DT_BGT: 66.4±7.1 | BGT: 17(12) DT_BGT: 23(11) | BGT: 5.3±3.8 DT_BGT: 6.3±6.0 | BGT: 2.5±0.7 DT_BGT: 2.7±0.6 | stable medication usage | ON | 10 | 2 | 60 |
| Sangarapillai, Norman ^175^ | 2021 | AE: 64.2±9.8 BGT_ECA: 65.1±9.2 | AE: 20 (NA) BGT_ECA: 20 (NA) | AE: 6.4±4.9 BGT_ECA: 7.8±5.2 | 2.5 | Levodopa AE: 612.1±220.8 BGT_ECA: 608.1±238.4 | ON | 10 | 3 | 60 |
| Santos, da Silva ^176^ | 2017 | BGT: 68.5±6.5  RT: 67.0±7.9 | BGT: 21(7) RT: 19(11) | BGT: 5.4±5.3 RT: 5.6±4.2 | BGT: 2.3±0.6 RT: 2.3±0.5 | NA | NA | 8 | 2 | 60 |
| Santos, Fernandez-Rio ^177^ | 2017 | BGT: 73±9.8 CON: 78±5.2 | BGT: 11(6) CON: 11(5) | BGT: 10.7±4.1 CON: 10.9±3.2 | BGT: 2.2±0.6 CON: 1.9±0.5 | stable medication usage | ON | 6 | 2 | 23 |
| Santos, Fernandez-Rio ^178^ | 2017 | RT: 73.4±8.8 CON: 73.8±7.1 | RT: 13(5) CON: 15(10) | RT: 10.8±4.1 CON: 10.5±4.0 | RT: 1.9±0.5 CON: 1.9±0.4 | Levodopa dosage RT: 457.5±164 CON: 473.7±178 | ON | 8 | 2 | 75 |
| Santos, Machado ^179^ | 2019 | VR: 61.7±7.3 Stretch: 64.5±9.8 VR: 66.6±8.2 | VR: 13(11) Stretch: 14(11) VR: 14(9) | VR: 7±2.8 Stretch: 6.5±2.0 VR: 7.8±3.7 | VR: 1.4±0.6 Stretch: 1.3±0.3 VR: 1.5±0.4 | stable medication usage | ON | 8 | 2 | 50 |
| Schabrun, Lamont ^180^ | 2016 | Mul_D: 72±4.9 DT_BGT: 63.0±11.0 | Mul_D: 8(8) DT_BGT: 8(2) | Mul_D: 6.9±4.4 DT_BGT: 4.6±3.9 | Mul_D: 2.0±1.5 DT_BGT: 2.0±1.5 | Levodopa equivalent daily dose Mul_D: 730±341 DT_BGT: 523±398 | ON | 3 | 3 | 60 |
| Schenkman, Cutson ^181^ | 1998 | CPP: 70.6±6.2 CON: 71.2±27.3 | CPP: 23(18) CON: 23(16) | NA | CPP: 2.7±0.7 CON: 2.5±0.6 | stable medication usage | ON | 10 | 3 | NA |
| Schenkman, Hall ^182^ | 2012 | Mul_C: 66.3±10.1 BGT: 64.5±10 AE: 63.4±11.2 | Mul_C: 41(26) BGT: 39 (24) AE: 41 (26) | Mul_C: 4.5±3.8 BGT: 4.9±3.7 AE: 3.9±4.2 | Mul_C: 2.3±0.4 BGT: 2.3±0.4 AE: 2.2±0.5 | NA | ON | 16 | 3 | 50 |
| Schenkman, Moore ^183^ | 2017 | TT: 64.0±9.0 TT: 63.0±10.0 CON: 64.0±10.0 | TT: 43(22) TT: 45(27) CON: 40(24) | TT 1.5±1.2 TT: 1.5±1.6 CON: 1.4±0.9 | TT: 1.72±0.45 TT: 1.71±0.45 CON: 1.80±0.40 | NA | OFF | 26 | 4 | 50 |
| Schilling, Pfeiffer ^184^ | 2010 | RT: 61.3±8.6 CON: 57.0±7.1 | RT: 8(5) CON:7 (4) | NA | RT: 2 ±0.1 CON: 1.9 ±0.3 | stable medication usage | ON | 8 | 2 | NA |
| Schlenstedt, Paschen ^185^ | 2018 | RT: 78.3±5.8 BGT: 81.4±7.3 | RT: 12(9) BGT: 8(6) | RT: 11.2±6.6 BGT: 8.4 ±7.3 | RT: 2.8±0.3 BGT: 2.9±0.5 | Levodopa  RT: 765±448 BGT: 652±286 | ON | 7 | 2 | 60 |
| Schlenstedt, Paschen ^186^ | 2015 | RT: 75.7 ± 5.5 BGT: 75.7 ± 7.2 | RT:17(12) BGT: 15(9) | RT: 10.1 ± 6.0 BGT: 9.3 ± 7.9 | RT: 2.8 ± 0.26 BGT: 2.7 ± 0.4 | levodopa equivalent dose RT: 817.4 ± 468.0 BGT: 674.7 ± 294.9 | ON | 7 | 2 | 60 |
| Schlick, Ernst ^187^ | 2016 | BGT_ECA: 71.2±10.9  TT: 68.9±6.8 | BGT_ECA: 10(2) TT: 10(4) | BGT_ECA: 10.4±5.2 TT: 9.1±3.1 | BGT_ECA: 2.8±0.9 TT: 2.7±0.7 | stable medication usage | ON | 5 | 2.5 | 35 |
| Sedaghati, Goudarzian ^188^ | 2018 | BGT: 64.9±2.6 CON: 63.2±3.3 | BGT: 13(8) CON: 13(6) | BGT: 5.3±1.8 CON: 4.3±0.7 | BGT: 2.6±0.5 CON: 2.7±0.5 | stable medication usage | ON | 8 | 3 | 60 |
| Serrao, Pierelli ^189^ | 2019 | Mul_C: 68.9±8.6 CPP: 71.2±7.5 | Mul_C: 21(11) CPP: 19(11) | Mul_C: 9.0±4.9 CPP: 8.5±3.5 | Mul_C: 2.9±0.9 CPP: 2.9±1.2 | Mul_C: 593.7±331.5 CPP: 623.5±328.6 | ON | 8 | 3 | 60 |
| Shahmohammadi, Sharifi ^190^ | 2017 | AQE: 60.5±5.4 AE: 53.2±4.9 | AQE: 10(10) AE: 10(10) | NA | NA | stable medication usage | ON | 8 | 3 | 55 |
| Shen and Mak ^191^ | 2014 | BGT_ECA: 63.3±8.0 RT: 65.3±8.5 | BGT_ECA: 22(13) RT: 23(12) | BGT_ECA: 8.1±4.3 RT: 6.6±4.0 | BGT_ECA: 2.5±0.5 RT: 2.5±0.5 | levodopa BGT_ECA: 505.1±347.4 RT: 618.1±619.6 | ON | 12 | 4 | 80 |
| Shen and Mak ^192^ | 2012 | BGT_ECA: 63.0±8.5  RT: 66.5±8.6 | BGT_ECA: 14(9) RT: 14(7) | BGT_ECA: 7.1±3.2 RT: 5.8±2.2 | BGT_ECA: 2.2±0.5 RT: 2.3±0.5 | Daily levodopa dosage BGT_ECA: 267.0±177.2 RT: 289.3±249.7 | ON | 4 | 3 | 60 |
| Shih, Wang ^193^ | 2016 | VR: 67.5±10.0 BGT: 68.8±9.7 | VR: 10(9) BGT: 10(7) | VR: 4.0±3.7 BGT: 5.2±4.9 | VR: 1.6±0.8 BGT: 1.4±0.5 | NA | ON | 8 | 2 | 50 |
| Shulman, Katzel ^194^ | 2013 | TT: 66.1±9.7 TT: 65.8±11.5 RT: 65.3±11.3 | TT: 23(16) TT: 22(16) RT: 22(18) | TT: 5.9±3.9 TT: 6.3±3.5 RT: 6.3±4.0 | TT: 2.15±0.34 TT: 2.16±0.35 RT: 2.23±0.39 | stable medication usage | ON | 12 | 3 | 45 |
| Silva and Israel ^195^ | 2018 | AQE: 63.12 ± 13.61 CON: 64.23 ± 13.45 | AQE: 14(6) CON: 11(5) | NA | AUR: 3±1 CON: 3±1 | stable medication usage | ON | 10 | 2 | 60 |
| Silva-Batista, Corcos ^196^ | 2016 | CON: 64.2±8.3  RT: 64.1±9.1  RT: 64.2±10.6 | CON: 13(9)  RT: 13(10)  RT: 13(10) | CON: 10.7±6.1  RT: 9.6±3.9  RT: 10.5±4.1 | CON: 2.5±0.4  RT: 2.5±0.5  RT: 2.5±0.4 | Levodopa equivalent dose CON: 796.7±151.3  RT: 835.8±287.0  RT: 875.9±223.4 | ON | 12 | 2 | 60 |
| Silva-Batista, Corcos ^197^ | 2018 | CON: 64.2±8.3  RT: 64.1±9.1  RT: 64.2±10.6 | CON: 13(9)  RT: 13(10)  RT: 13(10) | CON: 10.7±6.1  RT: 9.6±3.9  RT: 10.5±4.1 | CON: 2.5±0.4  RT: 2.5±0.5  RT: 2.5±0.4 | Levodopa equivalent dose CON: 796.7±151.3  RT: 835.8±287.0  RT: 875.9±223.4 | ON | 12 | 2 | 60 |
| Silva-Batista, Corcos ^198^ | 2016 | CON: 64.2±8.3  RT: 64.1±9.1  RT: 64.2±10.6 | CON: 13(9)  RT: 13(10)  RT: 13(10) | CON: 10.7±6.1  RT: 9.6±3.9  RT: 10.5±4.1 | CON: 2.5±0.4  RT: 2.5±0.5  RT: 2.5±0.4 | Levodopa equivalent dose CON: 796.7±151.3  RT: 835.8±287.0  RT: 875.9±223.4 | ON | 12 | 2 | 60 |
| Silva-Batista, de Brito ^199^ | 2016 | CON: 64.4±9.1 RT: 64.6±9.7 | CON: 11(8) RT: 11(8) | CON: 11.6±6.0 RT: 10.0±4.1 | CON: 2.5±0.4 RT: 2.5±0.5 | Levodopa  CON: 753.0±141.6 RT: 786.4±283.4 | ON | 12 | 2 | 60 |
| Silva-Batista, de Lima-Pardini ^200^ | 2020 | RT: 64.6±10.5 CPP: 66.8±8.9 | RT: 17(12) CPP: 15(9) | RT: 7.7±4.0 CPP: 10.0±5.6 | RT: 3.1±0.3 CPP: 3.2±0.4 | Levodopa RT: 437.5±211.7 CPP: 503.3±185.6 | ON | 12 | 3 | 85 |
| Smania, Corato ^201^ | 2010 | DT_BGT: 67.64±7.41 Stretch: 67.26±7.18 | DT_BGT: 28（14） Stretch:27（15） | DT_BGT: 10.39±4.76 Stretch: 8.63±5.39 | DT_BGT: 14.6±5.9 Stretch: 3.1±0.3 | NA | ON | 7 | 3 | 50 |
| Solla, Cugusi ^202^ | 2019 | Dance: 67.8±5.9 CON: 67.1±6.3 | Dance: 10(6) CON: 10(7) | Dance: 4.4±4.5 CON: 5.0±2.9 | Dance: 2.1±0.6 CON: 2.3±0.4 | levodopa  Dance: 481.1±213.1 CON: 487.5±198.5 | ON | 12 | 2 | 90 |
| Song, Paul ^203^ | 2018 | VR: 68±7 CON: 65±7 | VR: 31(15) CON: 29(9) | VR: 7±4 CON: 9±6 | NA | levodopa equivalent dose VR: 668±405 CON: 757±498 | ON | 12 | 3 | 15 |
| Steib, Klamroth ^204^ | 2017 | DT_BGT: 67.5 ±8.2 TT: 62.5±7.9 | DT_BGT: 18(11) TT: 20(16) | DT_BGT: 7.9±4.0 TT: 7.3±4.4 | DT_BGT: 2.6±0.5 TT: 2.5±0.5 | levodopa DT_BGT: 630.4±331.1 TT: 645.7±280.8 | ON | 8 | 2 | 40 |
| Steib, Klamroth ^205^ | 2019 | DT_BGT: 67.6±8.2 TT: 62.5±7.9 | DT_BGT: 18(11) TT: 20(16) | DT_BGT: 7.9±4.0 TT: 7.3±4.4 | DT_BGT: 2.6±0.5 TT: 2.5±0.5 | levodopa DT_BGT: 630.4±331.1 TT: 645.7±280.8 | ON | 8 | 2 | 40 |
| Stożek, Rudzińska ^206^ | 2015 | CPP: 64.0±9.9 CON: 67.0 ± 11.3 | CPP: 30(13) CON: 31(16) | CPP: 4.6 ± 2.7 CON: 4.3 ± 2.6 | CPP: 2.3 ± 0.6 CON: 2.3 ± 0.6 | stable medication usage | ON | 4 | 2.5 | 120 |
| Strand, Cherup ^207^ | 2021 | RT: 70.2±9.1 Mul_C: 68.6±10.5 | RT: 17 (9) Mul_C: 18 (11) | RT: 5.5±4.1 Mul_C: 5.8±3.5 | RT: 2.0±0.7 Mul_C: 1.9±0.6 | stable medication useage | ON | 12 | 3 | 60 |
| Strouwen, Molenaar ^208^ | 2017 | BGT: 66.0±9.3 DT_BGT: 65.8±9.2 | BGT: 65(49) DT_BGT: 56(39) | BGT: 8.9±6.3 DT_BGT: 8.4±5.3 | BGT: 2.4±0.5 DT_BGT: 2.3±0.5 | levodopa BGT: 752.3±453.2 DT_BGT: 613.0±396.1 | ON | 6 | 4 | 40 |
| Stuckenschneider, Helmich ^209^ | 2015 | AE: 71.0±4.6 CON: 71.5±5.7  AE: 71.4±4.9 | AE: 10(4) CON: 10(4) AE: 12(5) | NA | AE: 3±0.4 CON: 3.0±3 AE: 3.0±3 | stable medication usage | ON | 12 | 3 | 40 |
| Tang, Peng ^210^ | 2020 | BGT: 66. 85±9. 27 CON: 67. 13±8. 94 | BGT: 21(10) CON: 21(9) | BGT: 8.04±4.45 CON: 8.66±4.11 | BGT: 2.3±0.9 CON: 2.2±0.8 | NA | NA | 12 | 4 | 35 |
| Teixeira-Machado, Araújo ^211^ | 2015 | CPP: 60.70±9.88 CON: 61.0±10.46 | CPP: 15(NA) CON: 15(NA) | < 1 year | 2-3 | stable medication usage | ON | 25 | 2 | 60 |
| Terrens, Soh ^212^ | 2020 | AQE: 74.1±6.6 AQE: 65.6±7.7 CPP: 76.4±7.4 | AQE: 11(10) AQE: 10(7)  CPP: 9(7) | AQE: 6.7±6.3 AQE: 5.2±7.1  CPP: 4.2±3.1 | AQE: 3±1.5 AQE: 2±1.5  CPP: 3±0.7 | stable medication usage | ON | 12 | NA | 60 |
| Thaut, McIntosh ^213^ | 1996 | BGT_ECA: 69.0±8.0 CON: 71.0±8.0  BGT: 74.0±3.0 | BGT_ECA: 15(10) CON: 11(8) BGT: 11(8) | BGT_ECA: 7.2±4.0 CON: 8.5±4.0 BGT: 5.4±3.0 | BGT_ECA: 2.4±NA CON: 2.6±NA BGT: 2.5±NA | stable medication usage | ON | 3 | NA | 30 |
| Tollár, Nagy ^214^ | 2018 | VR: 70.0±4.69 AE: 70.6±4.10 CON: 67.5±4.28 | VR: 25(12) AE: 25(11) CON: 24(13) | VR: 7.5±1.76 AE: 7.5±2.16 CON: 7.3±2.21 | VR: 2.3±0.48 AE: 2.4±0.51 CON: 2.4±0.51 | Levodopa equivalent dose VR: 805.2±130.83 AE: 786.4±120.93 CON: 825.4±126.55 | ON | 5 | 5 | 60 |
| Tollár, Nagy ^215^ | 2018 | Mul_C: 67.3±3.4 CON: 67.6±4.1 | Mul_C: 35(17) CON: 20(12) | Mul_C: 6.7±2.3 CON: 7.1±2.8 | 2-3 | levodopa equivalent dose Mul_C: 843.4±308.8 CON: 884.8±332.0 | ON | 3 | 5 | 60 |
| Tollár, Nagy ^216^ | 2018 | Mul_C: 67.5±3.9 Mul_C: 67.6±3.3 CON: 67.6±4.1 | Mul_C: 19(11) Mul_C: 16(6) CON: 20(12) | Mul_C: 6.5±2.7 Mul_C: 6.8±1.8 CON: 7.1±2.8 | Mul_C: 2.5±0.5 Mul_C: 2.3±0.5 CON: 2.4±0.5 | levodopa equivalent dose  Mul_C: 774.2±381.5 Mul_C: 912.6±380.1 CON: 884.8±332.0 | ON | 3 | 5 | NA |
| Tramontano, Bonnì ^217^ | 2016 | BGT: 70.1 ± 8.5 CPP: 69.0 ± 10.3 | BGT: 15 (NA) CPP: 15 (NA) | BGT: 7.9 ± 5.0 CPP: 8.8 ± 6.6 | NA | Levodopa  ALL: 719 ± 356 | ON | 5 | 5 | 90 |
| van den Heuvel, Kwakkel ^218^ | 2014 | BGT_ECA: 66.3±6.39 DT_BGT: 68.8±9.68 | BGT_ECA: 17(12) DT_BGT: 16(8) | BGT_ECA: 9.0±2.3 DT_BGT: 8.8±2.3 | BGT_ECA: 2.5±0.4 DT_BGT: 2.5±0.4 | stable medication usage | ON | 5 | 2 | 60 |
| van der Kolk, de Vries ^219^ | 2019 | AE: 59.3±8.3 Stretch 59.4±9.3 | AE: 65(42) Stretch: 65(38) | AE: 3.4±4.4 Stretch: 3.1±3.8 | AE: 1.94±0.24 Stretch: 1.95±0.21 | levodopa  AE: 600±128.75 Stretch: 532±134.50 | ON/OFF | 24 | 3 | 30-45 |
| van der Kolk, de Vries ^220^ | 2018 | NA | AE: 22(NA) CON: 15(NA) | NA | NA | NA | OFF | 24 | 3 | 30 |
| van Nimwegen, Speelman ^221^ | 2013 | Mul_D: 65.1±7.9 CPP: 65.9±7.2 | Mul_D: 273(194) CPP: 267(188) | Mul_D: 5.0±4.5 CPP: 5.5±4.6 | Mul_D: 2.1±0.3 CPP: 2.1±0.3 | levodopa Mul_D: 458 (362) CPP: 499 (414) | ON | 96 | 3 | 100 |
| Van Puymbroeck, Walter ^222^ | 2018 | Yoga: 65.5±6.1 CON: 70.5±4.4 | Yoga: 15(10) CON: 12(7) | NA | NA | NA | NA | 8 | 2 | NA |
| Vanbellingen, Nyffeler ^223^ | 2017 | Mul_C: 67.15±7.94 RT: 68.16±7.38 | RT: 51(29) | Mul_C: 6.12±3.52 RT: 6.35±3.99 | Mul_C: 1.94±0.90 RT: 2.00±0.82 | levodopa Mul_C: 741.63±471.8 RT: 745.43±502.69 | ON | 4 | 5 | 30 |
| Vergara-Diaz, Osypiuk ^224^ | 2018 | TC: 65.7±3.9 CON: 62.0±7.8 | TC: 16(9) CON: 16(7) | TC: 2.9±2.4 CON: 2.9±2.2 | TC: 2.2±0.2 CON: 2.1±0.2 | NA | OFF | 24 | 2 | 60 |
| Vieira de Moraes Filho, Chaves ^225^ | 2020 | RT: 64.7±9.0 CON: 64.4±5.8 | RT: 25(20) CON: 15(10) | RT: 5.7±4.0 CON: 7.2±7.4 | NA | NA | NA | 9 | 2 | 55 |
| Vieira-Yano, Martini ^226^ | 2021 | CPP: 66.8±9.0 RT: 64.6±10.6 | CPP: 15(9) RT: 17(12) | CPP: 10.0±5.6 RT: 7.7±4.0 | CPP: 3.2±0.4 RT: 3.1±0.3 | Levodopa CPP: 503.3±185.6 RT: 437.5±211.7 | ON | 12 | 3 | 85 |
| Vitório, Teixeira-Arroyo ^227^ | 2011 | CPP: 67.5±8.3 CON: 71.3±8.1 | CPP: 19(NA) CON: 10(NA) | CPP: 3.8±3.9 CON: 4.4±2.8 | CPP: 1.5±0.8 CON: 1.6±0.9 | stable medication usage | ON | 24 | 3 | 60 |
| Vivas, Arias ^228^ | 2011 | AQE: 65.67±3.67 CPP: 68.33±6.92 | AQE: 6(3) CPP: 6(4) | AQE: 4.17±1.6 CPP: 7.83±3.92 | AQE: 2.67±0.58 CPP: 2.4±0.55 | NA | OFF | 4 | 2 | 45 |
| Volpe, Giantin ^229^ | 2014 | BGT_ECA: 66.5±10.4 BGT: 69.5±6.5 | BGT_ECA: 20(7) BGT: 20(9) | BGT_ECA: 6.0±5.0 BGT: 6.5±3.7 | BGT_ECA: 3.0±0.0 BGT: 3.0±0.7 | Levodopa equivalent dose  BGT_ECA: 487.5±277.8 BGT: 450.0±226.8 | ON | 8 | 5 | 60 |
| Volpe, Giantin ^230^ | 2014 | AQE: 68 ± 7 DT_BGT: 66 ± 8 | AQE:17(NA) DT_BGT:17(NA) | AQE: 7.5 ± 5.1 DT_BGT: 7.6 ± 4.63 | AQE: 2.82 ± 0.3 DT_BGT: 2.65 ± 0.49 | Levodopa equivalent dose AQE: 645.4 ± 206 DT_BGT: 625.2 ± 244.3 | ON | 8 | 5 | 60 |
| Volpe, Giantin ^231^ | 2016 | AQE: 70.6 ± 7.8  Mul_C: 70 ± 7.8 | AQE: 15(9) Mul_C: 15(10) | AQE: 9.4 ± 7.5  Mul_C: 9 ± 7.0 | AQE: 2.6 ± 0.5 Mul_C: 2.7 ± 0.5 | Levodopa equivalent dose AQE: 437.2 ± 179.9  Mul_C: 353.1 ± 280.9 | ON | 8 | 5 | 60 |
| Volpe, Signorini ^232^ | 2013 | Dance: 61.6±4.5 CPP: 65.0±5.3 | Dance: 12(7)  CPP: 12(6) | Dance: 9.0±3.6 CPP: 8.9±2.5 | Dance: 2.2±0.4 CPP: 2.2±0.4 | Levodopa Dance: 725.0 ±234 CPP: 645.0±216 | ON | 24 | 1 | 90 |
| Wallén, Hagströmer ^233^ | 2018 | DT_BGT: 73.1±5.8  CON: 73.0±5.5 | DT_BGT: 51(32) CON: 49(25) | DT_BGT: 5.9±5.1 CON: 5.6±4.8 | DT_BGT: 2.55±0.5 CON: 2.57±0.5 | levodopa equivalent dose DT_BGT: 578±299 CON: 640±380 | ON | 10 | 3 | 60 |
| Wang, Bi ^234^ | 2020 | CPP: 64.64±7.43 Qigong: 64.23±5.14 | CPP: 21(11) Qigong: 20(12) | CPP: 6.57±3.43 Qigong: 7.14±3.57 | CPP: 2.26±0.46 Qigong: 2.17±0.47 | NA | NA | 6 | 10 | 60 |
| White, Wagenaar ^235^ | 2009 | CON: 66.0±8.4  Mul_C: 65.6±9.2  Mul_C: 68.2±9.8 | CON: 25(17) Mul_C: 21(15) Mul_C: 28(20) | CON: 5.6±3.5  Mul_C: 5.1±4.1 Mul_C: 6.5±4.3 | CON: 2.3±0.4 Mul_C: 2.3±0.4 Mul_C: 2.3±0.3 | NA | ON | 6 | 2 | 90 |
| Wong-Yu and Mak ^236^ | 2015 | Mul_C: 59.4±9.0 CON: 62.6±8.9 | Mul_C: 41(25) CON: 39(21) | Mul_C: 7.1±4.3 CON: 5.6±3.8 | Mul_C: 2.5±0.3 CON: 2.4±0.3 | Daily levodopa equivalent dosage Mul_C: 409.3±288.2 CON: 320.8±391.0 | ON | 8 | 1 | 120 |
| Wong-Yu and Mak ^237^ | 2015 | DT_BGT: 60.2±9.0  Stretch: 61.9±8.5 | DT_BGT: 32(19) Stretch: 36(20) | DT_BGT: 7.3±4.6  Stretch: 5.4±3.6 | DT_BGT: 2.5±0.3  Stretch: 2.4±0.3 | levodopa DT_BGT: 362.9±260.8  Stretch: 294.7±362.7 | ON | 8 | NA | 120 |
| Xiao and Zhuang ^238^ | 2015 | CON: 66.5±2.1 Qigong: 68.1±2.3 | CON: 48(34) Qigong: 48(33) | CON: 6.2±2.6 Qigong: 5.5±3.6 | CON: 2.1±0.2 Qigong: 2.2±0.2 | NA | OFF | 24 | 4 | 50 |
| Xiao, Zhuang ^239^ | 2016 | 67.8±9.4 | Qigong: 49 CON: 49 | NA | NA | NA | NA | 24 | 4 | 60 |
| Yang, Cheng ^240^ | 2019 | DT_BGT: 65.0±57.5 DT_BGT: 69.5±65.0 BGT: 66.5±55.5 | DT_BGT: 6(4) DT_BGT: 6(4) BGT: 6(4) | DT_BGT: 5.5±2.8 DT_BGT: 5.0±0.1 BGT: 3.0±0.3 | DT_BGT: 2.0±1.6 DT_BGT: 2.0±1.8 BGT: 1.5±0.9 | levodopa equivalent dose DT_BGT: 892.0(432.2-1307.5) DT_BGT: 798.0(534.8-1074.2) BGT: 557(205.7-1234.7) | ON | 4 | 3 | 30 |
| Yang, Lee ^241^ | 2010 | DT_BGT: 68.07±7.51 CPP: 66.27±10.72 | DT_BGT: 15(9) CPP: 15(7) | DT_BGT: 4.77±4.83 CPP: 5.27±5.55 | DT_BGT: 2.23±0.53 CPP: 2.17±0.72 | Levodopa DT_BGT: 440.0±186.5 CPP: 571.6±250.1   Ropinirole  DT_BGT: 4.3±2.3 CPP: 4.0±1.7 | ON | 4 | 3 | 30 |
| Yang, Wang ^242^ | 2016 | VR: 72.5±8.4 BGT: 75.4±6.3 | VR: 11(7) BGT: 12(7） | VR: 9.4±3.6 BGT: 8.3±4.1 | VR: 3±0 BGT: 3±0 | stable medication usage | ON | 6 | 2 | 50 |
| Yi-zhao, Hua ^243^ | 2017 | CPP: 64.5±6.8 AQE: 63.4±7.2 | CPP: 20(12) AQE: 20(14) | CPP: 4.3±1.7 AQE: 3.8±1.4 | CPP: 2.0±0.6 AQE: 1.9±0.5 | NA | NA | 8 | 5 | 50 |
| Yotnuengnit, Bhidayasiri ^244^ | 2017 | Mul_D: 68.2±9.8 CPP: 62.7±8.8 | Mul_D: 17(11) CPP: 18(12) | Mul_D: 9.4±5.3 CPP: 6.6±3.6 | Mul_D: 2.5±0.5 CPP: 2.4±0.5 | Levodopa equivalent dose Mul_D: 829.0±360.6 CPP: 912.0±472.9 | ON | 2 | 3 | 30 |
| You and She ^245^ | 2020 | BGT: 68.49±5.27 TC: 68.81±5.02 | BGT: 35(19) TC: 35(18) | BGT: 4.17±0.35 TC: 4.21±0.24 | 3 | NA | NA | 24 | 2 | 60 |
| Youm, Kim ^246^ | 2020 | RT: 68.0±6.8 Stretch: 72.1±6.0 | RT: 10(6) Stretch: 7(4) | RT: 6.4±3.6 Stretch: 8.0±4.0 | RT: 2.4±0.3 Stretch: 2.3±0.4 | Levodopa equivalent dose RT: 567.0±274.6 Stretch: 852.9±564.4 | ON | 12 | 3 | 75 |
| Zeng, Tian ^247^ | 2020 | WBV: 63.27±4.54 VR: 63.72±3.37 Mul_C: 62.36±3.10 | WBV: 11(6)  VR: 11(5) Mul_C: 11(7) | WBV: 6.36±1.28  VR: 6.81±1.60 Mul_C: 6.18±1.32 | WBV: 2.77±0.46 VR: 2.72±0.46 Mul_C: 2.68±0.25 | stable medication usage | ON | 6 | 5 | 30 |
| Zhang, Hu ^248^ | 2015 | TC: 66.0±11.8 Mul_C: 64.4±10.5 | TC: 20(13) Mul_C: 20(11) | TC: 6.8±5.4 Mul_C: 4.9±3.7 | TC: 2.0±0.5 Mul_C: 2.2±0.4 | levodopa equivalent dose TC: 474.0±331.9 Mul_C: 389.3±322.8 | ON | 12 | 2 | 60 |
| Zhang, Zhou ^249^ | 2020 | Mul_C: 66.8±2.53 BGT: 66.59±2.73 | Mul_C: 42(26) BGT: 41(26) | 3~6 | NA | NA | NA | 12 | 7 | 35 |
| Zheng, Zheng ^250^ | 2020 | RT: 72.34±5.39 CON: 74.39±5.38 | RT: 35(21) CON: 35(20) | RT: 6.34±0.27 CON: 6.39±0.25 | 1~3 | NA | NA | 4 | 3 | 25 |

*NA* not available, *AE* Aerobic Exercise, *AQE* Aquatic Exercise, *BGT* Balance and Gait Training, *BGT_ECA* Balance and Gait Training with External Cue or Attention, *BGT_ICA* Balance and Gait Training with Internal Cue or Attention, *BWS_TT* Body Weight Support Treadmill Training, *CON* Control group, *CPP* Classic Physiotherapy Program, *DT_BGT* Dual Task Balance and Gait Training, *Mul_C* Multicomponent Exercise Program, *Mul_D* Multidisciplinary Exercise Program, *NW* Nordic Walking, *PT* Power Training, *RA_GT* Robotic Assisted Gait Training, *RT* Resistance Training, *TC* Tai Chi, *TT* Treadmill Training, *VR* Virtual Reality, *WBV* Whole Body Vibration.

## 8.2 List of available outcomes for included studies

| **Author** | **Motor symptoms** | **Balance test batteries** | **Gait velocity** | **Freezing of gait** | **Depression** | **Anxiety** | **Sleep** | **Cognitive** | **Walking distance** | **Muscle strength** | **Concern of falling** |
| --- | --- | --- | --- | --- | --- | --- | --- | --- | --- | --- | --- |
| Abraham, Hart ^1^ | UPDRS motor, score | Mini-Balance Evaluation Systems Test | 6m walking test, velocity | NA | NA | NA | NA | cognition functional test, time | 6-min walk test, m | NA | Activity Specific Balance Confidence scale, score |
| Ajimsha, Majeed ^2^ | UPDRS motor, score | NA | NA | NA | NA | NA | NA | NA | NA | NA | NA |
| Allen, Canning ^3^ | NA | NA | 2.5m walking test, velocity | freezing of gait questionnaire, score | NA | NA | NA | NA | NA | knee extension strength, kg | Falls Efficacy Scale International, score |
| Allen, Song ^4^ | NA | NA | NA | NA | NA | NA | NA | Montreal Cognitive Assessment, score | NA | NA | NA |
| Altmann, Stegemöller ^5^ | UPDRS motor, score | NA | NA | NA | Beck Depression Inventory | Beck Anxiety Index | Pittsburgh Sleep Quality Index, score | Mattis Dementia Rating Scale-2 | NA | NA | NA |
| Amano, Nocera ^6^ | UPDRS motor, score | NA | eight gait trials, gait velocity | NA | NA | NA | NA | NA | NA | NA | NA |
| Amara, Wood ^7^ | NA | NA | NA | NA | NA | NA | sleep efficiency, score | NA | NA | NA | NA |
| Arcolin, Pisano ^8^ | UPDRS motor, score | mini-Balance Evaluation Systems Test, score | 2m walking test, gait velocity | NA | NA | NA | NA | NA | 6 min walk test, m | NA | NA |
| Arfa-Fatollahkhani, Safar Cherati ^9^ | NA | NA | NA | NA | NA | NA | NA | NA | 6-min walk test, m | NA | NA |
| Ashburn, Fazakarley ^10^ | NA | Berg Balance Scale, score | NA | NA | NA | NA | NA | NA | NA | NA | NA |
| Atan, Özyemişci Taşkıran ^11^ | UPDRS motor, score | Berg Balance Scale, score | NA | NA | NA | NA | NA | NA | 6-min walk test, m | NA | NA |
| Bakhshayesh, Sayyar ^12^ | NA | fullerton advanced balance，score | NA | NA | NA | NA | NA | NA | NA | NA | NA |
| Bang and Shin ^13^ | UPDRS motor, score | Berg Balance Scale, score | 10m walking test, time | NA | NA | NA | NA | NA | 6-min walk test, m | NA | NA |
| Barboza, Terra ^14^ | UPDRS motor, score | NA | NA | NA | NA | NA | NA | Cognitive and Perceptual Assessment by pictures, time | NA | NA | NA |
| Beck, Intzandt ^15^ | UPDRS motor, score ＃ | NA | walking test, gait velocity | NA | NA | NA | NA | Montreal Cognitive Assessment, score | NA | NA | NA |
| Beck, Wang ^16^ | UPDRS motor, score ＃ | NA | NA | NA | NA | Parkinson Anxiety Scale Total, score | NA | Montreal Cognitive Assessment, score | NA | NA | NA |
| Bekkers, Mirelman ^17^ | NA | mini-Balance Evaluation Systems Test, score | NA | New Freezing of Gait Questionnaire, score | NA | NA | NA | Executive cognitive function, score | NA | NA | Falls Efficacy Scale, score |
| Bello, Sanchez ^18^ | UPDRS motor, score | NA | 4 min walking test, velocity | NA | NA | NA | NA | NA | NA | knee extension strength, N | NA |
| Burini, Farabollini ^19^ | UPDRS motor, score | NA | NA | NA | Beck Depression Inventory, score | NA | NA | NA | 6 min walk test, m | NA | NA |
| Cabrera-Martos, Jiménez-Martín ^20^ | NA | Mini-Balance Evaluation Systems Test, score | NA | NA | NA | NA | NA | NA | NA | NA | Activity Specific Balance Confidence scale, score |
| Cakit, Saracoglu ^21^ | NA | Berg Balance Scale, score | maximum velocity on treadmill | NA | NA | NA | NA | NA | walking test, m | NA | Falls Efficacy Scale International, score |
| Calabrò, Naro ^22^ | NA | Berg Balance Scale, score | 10m walking test, velocity | NA | NA | NA | NA | NA | NA | NA | Tinetti Falls Efficacy Scale |
| Cancela, Mollinedo ^23^ | UPDRS motor, score | Tinetti assessment scale, score | NA | NA | NA | NA | NA | NA | 6 min walking test, m | NA | NA |
| Canning, Allen ^24^ | UPDRS motor, score | NA | 10m walking test comfortable pace, gait velocity | NA | NA | NA | NA | NA | 6-min walk test, m | NA | NA |
| Capato, de Vries ^25^ | UPDRS motor, score | Mini-Balance Evaluation Systems Test, score | NA | Freezing of Gait Questionnaire, score | NA | NA | NA | NA | NA | NA | Falls Efficacy Scale International, score |
| Capato, Nonnekes ^26^ | UPDRS motor, score | Berg Balance Scale, score | NA | Freezing of Gait Questionnaire, score | NA | NA | NA | NA | NA | NA | Falls Efficacy Scale International, score |
| Capecci, Pournajaf ^27^ | UPDRS motor, score | NA | 10m walking test, gait velocity | Freezing of Gait Questionnaire, score | NA | NA | NA | NA | 6-min walk test, m | NA | NA |
| Capecci, Serpicelli ^28^ | NA | Berg Balance Scale, score | NA | NA | NA | NA | NA | NA | NA | NA | NA |
| Carda, Invernizzi ^29^ | UPDRS motor, score | NA | 10m walking test, velocity | NA | NA | NA | NA | NA | 6-min walk test, m | NA | NA |
| Carpinella, Cattaneo ^30^ | UPDRS motor, score | Berg Balance Scale, score | 10m walking test, gait velocity | Freezing of Gait Questionnaire, score | NA | NA | NA | NA | NA | NA | Activity Specific Balance Confidence scale |
| Carroll, Volpe ^31^ | UPDRS motor, score | NA | NA | Freezing of Gait Questionnaire, score | NA | NA | NA | NA | NA | NA | NA |
| Carvalho, Barbirato ^32^ | UPDRS motor, score | Berg Balance Scale, score | 10m walking test, time | NA | NA | NA | NA | NA | 2 min step test, repetitions | NA | NA |
| Cheng, Yang ^33^ | NA | Tinetti assessment scale, score | NA | NA | NA | NA | NA | NA | NA | NA | NA |
| Cheng, Yang ^34^ | UPDRS motor, score | NA | 6m walking test, gait velocity | Freezing of Gait Questionnaire, score | NA | NA | NA | NA | NA | NA | NA |
| Cherup, Strand ^35^ | NA | Tinetti assessment scale, score | NA | NA | NA | NA | NA | NA | NA | NA | Falls Efficacy Scale International, score |
| Cheung, Bhimani ^36^ | UPDRS motor, score | NA | NA | NA | Beck Depression Inventory, score | NA | PD Sleep Scale, score | Montreal Cognitive Assessment, score | NA | NA | NA |
| Chivers Seymour, Pickering ^37^ | NA | Mini-Balance Evaluation Systems Test, score | NA | NA | Geriatric Depression Scale, score | NA | NA | NA | NA | NA | Falls Efficacy Scale International, score |
| Choi ^38^ | UPDRS motor, score | NA | NA | NA | NA | NA | NA | NA | 6 min walk test, m | NA | NA |
| Clarke, Patel ^39^ | NA | NA | NA | NA | NA | NA | NA | PDQ 39-cognitive | NA | NA | NA |
| Clerici, Maestri ^40^ | UPDRS motor, score | Berg Balance Scale, score | NA | Freezing of Gait Questionnaire, score | NA | NA | NA | NA | 6 min walk test, m | NA | NA |
| Combs, Diehl ^41^ | NA | Berg Balance Scale, score | 4.8m walking test, gait velocity | NA | NA | NA | NA | NA | 6 min walk test, m | NA | Activity Specific Balance Confidence scale, score |
| Conradsson, Löfgren ^42^ | UPDRS motor, score | Mini-Balance Evaluation Systems Test, score | 9m walking test, velocity | NA | NA | NA | NA | NA | NA | NA | Falls Efficacy Scale International, score |
| Corcos, Robichaud ^43^ | UPDRS motor, score ＃ | NA | NA | NA | NA | NA | NA | NA | NA | NA | NA |
| Costa-Ribeiro, Maux ^44^ | UPDRS motor, score | Berg Balance Scale, score | 10m walking test, gait velocity | NA | NA | NA | NA | NA | NA | NA | NA |
| Cugusi, Solla ^45^ | UPDRS motor, score | Berg Balance Scale, score | NA | NA | Beck Depression Inventory, score | NA | NA | NA | 6 min walk test, m | NA | NA |
| Daneshmandi, Sayyar ^46^ | NA | Fullerton Advanced Balance, score | NA | NA | NA | NA | NA | NA | NA | NA | NA |
| Dashtipour, Johnson ^47^ | UPDRS motor, score | NA | NA | NA | Beck Depression Inventory, score | Beck anxiety inventory, score | NA | NA | NA | NA | NA |
| David, Robichaud ^48^ | NA | NA | NA | NA | NA | NA | NA | Stroop Color-Word Interference, score | NA | NA | NA |
| David, Robichaud ^49^ | NA | NA | NA | NA | NA | NA | NA | NA | NA | elbow extension strength, Nm | NA |
| de Bruin, Doan ^50^ | UPDRS motor, score | NA | walking test, velocity | NA | NA | NA | NA | NA | NA | NA | NA |
| de Lima, Ferreira-Moraes ^51^ | NA | NA | 10m walking test, gait velocity | NA | Hamilton Depression Rating Scale, score | NA | NA | NA | 2 min step test, repetitions | handgrip strength, kg | NA |
| de Melo, Kleiner ^52^ | NA | NA | 6 min walking test, velocity | NA | NA | NA | NA | NA | 6 min walk test, m | NA | NA |
| Demonceau, Maquet ^53^ | NA | NA | 30m walking test, velocity | NA | NA | NA | NA | PDQ-39 cognition, score | NA | knee extension strength, Nm/kg | NA |
| Dibble, Foreman ^54^ | UPDRS motor, score ＃ | NA | NA | NA | NA | NA | NA | NA | 6 min walk test, m ＃ | Quadriceps force, N ＃ | NA |
| Dibble, Hale ^55^ | NA | NA | NA | NA | NA | NA | NA | NA | 6-min walk test, m | knee extension, average torque | NA |
| DiFrancisco-Donoghue, Lamberg ^56^ | NA | NA | NA | NA | NA | NA | NA | NA | NA | leg press, lb | NA |
| Dipasquale, Meroni ^57^ | UPDRS motor, score | NA | NA | NA | NA | Hamilton Rating Scale, score | NA | NA | NA | NA | NA |
| Droby, Maidan ^58^ | NA | Mini-Balance Evaluation Systems Test | 30m walking test, velocity | NA | NA | NA | NA | Color Trails Test, score | NA | NA | NA |
| Duncan and Earhart ^59^ | UPDRS motor, score | NA | NA | NA | NA | NA | NA | NA | NA | NA | NA |
| Ebersbach, Ebersbach ^60^ | UPDRS motor, score | NA | 10m walking test, time | NA | NA | NA | NA | NA | NA | NA | NA |
| Ebersbach, Edler ^61^ | UPDRS motor, score | Tinetti assessment scale, score | 10m walking test, gait velocity | NA | NA | NA | NA | NA | NA | NA | NA |
| Ellis, de Goede ^62^ | UPDRS motor, score | NA | Walking Test, velocity | NA | NA | NA | NA | NA | NA | NA | NA |
| El-Tamawy, Darwish ^63^ | NA | NA | walking test, velocity | NA | NA | NA | NA | NA | walking test, m | NA | NA |
| Feng, Li ^64^ | UPDRS motor, score | Berg Balance Scale, score | NA | NA | NA | NA | NA | NA | NA | NA | NA |
| Fernandes, Rocha ^65^ | UPDRS motor, score | NA | NA | NA | NA | NA | NA | Rule Shift Cards Test, score | NA | NA | NA |
| Ferraz, Trippo ^66^ | NA | NA | 10m walking test, velocity | NA | Geriatric Depression Scale, score | NA | NA | NA | 6 min walk test, m | NA | NA |
| Ferrazzoli, Ortelli ^67^ | NA | NA | NA | NA | NA | NA | NA | PDQ-39 cognition, score | NA | NA | NA |
| Ferreira, Alves ^68^ | UPDRS motor, score | NA | NA | NA | NA | Beck anxiety inventory, score | NA | NA | NA | NA | NA |
| Fietzek, Schroeteler ^69^ | NA | NA | NA | freezing of gait questionnaire, score | NA | NA | NA | NA | NA | NA | NA |
| Fil-Balkan, Salci ^70^ | UPDRS motor, score | Berg Balance Scale, score | NA | NA | NA | NA | NA | NA | NA | NA | NA |
| Fisher, Wu ^71^ | UPDRS motor, score | NA | 10m walking test, gait velocity | NA | NA | NA | NA | NA | NA | NA | NA |
| Franzoni, Monteiro ^72^ | NA | Berg Balance Scale, score | NA | NA | NA | NA | NA | NA | NA | NA | NA |
| Frazzitta, Bertotti ^73^ | UPDRS motor, score | NA | NA | NA | NA | NA | NA | NA | NA | NA | NA |
| Frazzitta, Bossio ^74^ | UPDRS motor, score | Berg Balance Scale, score | NA | NA | NA | NA | NA | NA | 6 min walk test, m | NA | NA |
| Frazzitta, Maestri ^75^ | UPDRS motor, score | NA | walking test, velocity | Freezing of Gait Questionnaire, score | NA | NA | NA | NA | 6 min walk test, m | NA | NA |
| Frazzitta, Maestri ^76^ | UPDRS motor, score | NA | NA | NA | NA | NA | NA | NA | NA | NA | NA |
| Furnari, Calabrò ^77^ | UPDRS motor, score | Tinetti assessment scale, score | NA | NA | Geriatric Depression Scale, score | NA | NA | NA | NA | NA | NA |
| Galli, Cimolin ^78^ | UPDRS motor, score | NA | 10m walking test, gait velocity | NA | NA | NA | NA | NA | NA | NA | NA |
| Gandolfi, Geroin ^79^ | NA | Berg Balance Scale, score | 10m walking test, velocity | NA | NA | NA | NA | NA | NA | NA | Activity Specific Balance Confidence scale |
| Gandolfi, Tinazzi ^80^ | UPDRS motor, score | Mini-Balance Evaluation Systems Test, score | NA | NA | NA | NA | NA | NA | NA | NA | NA |
| Ganesan, Sathyaprabha ^81^ | UPDRS motor, score | NA | 10m walking test, gait velocity | NA | NA | NA | NA | NA | walking test, m | NA | NA |
| Gao, Leung ^82^ | UPDRS motor, score | Mini-Balance Evaluation Systems Test, score | NA | NA | NA | NA | NA | NA | NA | NA | NA |
| Gaßner, Steib ^83^ | UPDRS motor, score | NA | NA | NA | NA | NA | NA | NA | NA | NA | NA |
| Ghielen, van Wegen ^84^ | NA | NA | 10m walking test, time | Freezing of Gait Questionnaire, score | Beck Depression Inventory, score | Beck anxiety inventory, score | NA | PDQ-39 cognition, score | NA | NA | NA |
| Ginis, Nieuwboer ^85^ | UPDRS motor, score | Mini-Balance Evaluation Systems Test, score | 1min walking test, gait velocity | Freezing of Gait Questionnaire, score | NA | NA | NA | Cognition task test, time | 2 min walk test, m | NA | Falls Efficacy Scale International, score |
| Gobbi, Pelicioni ^86^ | UPDRS motor, score | NA | NA | NA | hospital anxiety and depression scale-depression, score | hospital anxiety and depression scale-anxiety, score | NA | Mini-Mental State Examination, score | NA | NA | NA |
| Goodwin, Richards ^87^ | NA | Berg Balance Scale, score | NA | NA | NA | NA | NA | NA | NA | NA | Falls Efficacy Scale International, score |
| Grobbelaar, Venter ^88^ | UPDRS motor, score | NA | 10-m walk test, velocity | NA | NA | NA | NA | NA | NA | NA | NA |
| Hackney and Earhart ^89^ | UPDRS motor, score | Berg Balance Scale, score | 5m walking test, gait velocity | Freezing of Gait Questionnaire, score | NA | NA | NA | NA | 6 min walk test, m | NA | NA |
| Hackney and Earhart ^90^ | UPDRS motor, score | Berg Balance Scale, score | walking test, velocity | NA | NA | NA | NA | NA | 6 min walk test, m | NA | NA |
| Hackney and Earhart ^91^ | NA | NA | NA | NA | NA | NA | NA | PDQ 39-cognitive | NA | NA | NA |
| Hackney, Kantorovich ^92^ | UPDRS motor, score | Berg Balance Scale, score | 5m walking test, gait velocity | Freezing of Gait Questionnaire, score | NA | NA | NA | NA | NA | NA | NA |
| Harro, Shoemaker ^93^ | NA | Berg Balance Scale, score | NA | NA | NA | NA | NA | NA | NA | NA | Activity Specific Balance Confidence scale, score |
| Hashimoto, Takabatake ^94^ | NA | Berg Balance Scale, score | NA | NA | NA | NA | NA | Frontal assessment battery, score | NA | NA | NA |
| Hass, Collins ^95^ | UPDRS motor, score | NA | NA | NA | NA | NA | NA | NA | NA | NA | NA |
| Helgerud, Thomsen ^96^ | NA | NA | NA | NA | NA | NA | NA | NA | 6 min walk test, m | 1RM leg press, kg | NA |
| Hirsch, Toole ^97^ | NA | NA | NA | NA | NA | NA | NA | NA | NA | muscle strength, 4-repetition maximum test | Latency to Fall |
| Hubble, Naughton ^98^ | UPDRS motor, score | NA | NA | Freezing of Gait Questionnaire, score | NA | NA | NA | NA | NA | NA | Gait and Falls Questionnaire, score |
| Johansson, Cameron ^99^ | MDSUPDRS motor, score | NA | NA | NA | NA | NA | NA | Montreal Cognitive Assessment, score | NA | NA | NA |
| Johansson, Freidle ^100^ | NA | Mini-Balance Evaluation Systems Test, score | walking test, velocity | NA | NA | NA | NA | color word interference test, time | NA | NA | NA |
| Joseph, Brodin ^101^ | NA | Mini-Balance Evaluation Systems Test, score | 9m walking test, gait velocity | NA | NA | NA | NA | NA | NA | NA | NA |
| Jung, Hasegawa ^102^ | UPDRS motor, score | mini-Balance Evaluation Systems Test, score | NA | NA | NA | NA | NA | Scales for Outcomes in Parkinson’s disease-Cognition | NA | NA | NA |
| Kadivar, Corcos ^103^ | UPDRS motor, score | Tinetti-gait and balance tests, score | NA | Freezing of Gait Questionnaire, score | NA | NA | NA | NA | NA | NA | NA |
| Keus, Bloem ^104^ | NA | NA | NA | NA | NA | NA | NA | NA | NA | NA | NA |
| Khalil, Busse ^105^ | MDS-UPDRS motor, score | Mini-Balance Evaluation Systems Test, score | 10m walking test, gait velocity | NA | NA | NA | NA | NA | 6 min walk test, m | NA | Falls Efficacy Scale International, score |
| Khuzema, Brammatha ^106^ | NA | Berg Balance Scale, score | 10m walking test, time | NA | NA | NA | NA | NA | NA | NA | NA |
| Kim, Kim ^107^ | MDSUPDRS motor, score | Berg Balance Scale, score | walking test, velocity | Freezing of Gait Questionnaire | NA | NA | NA | NA | NA | NA | NA |
| King, Salarian ^108^ | UPDRS motor, score | mini-Balance Evaluation Systems Test, score | 7 meters away, Stride velocity | NA | NA | NA | NA | NA | NA | NA | Activity Specific Balance Confidence scale, score |
| Kunkel, Fitton ^109^ | NA | Berg Balance Scale, score | NA | NA | NA | NA | NA | NA | 6 min walking test, m | NA | Activity Specific Balance Confidence scale |
| Kurt, Büyükturan ^110^ | UPDRS motor, score | Berg Balance Scale, score | NA | NA | NA | NA | NA | NA | NA | NA | NA |
| Kurtais, Kutlay ^111^ | NA | NA | 20m walking test, gait velocity | NA | NA | NA | NA | NA | NA | NA | NA |
| Kwok, Kwan ^112^ | UPDRS motor, score | NA | NA | NA | hospital anxiety and depression scale-depression, score | hospital anxiety and depression scale-anxiety, score | NA | NA | NA | NA | NA |
| Landers, Hatlevig ^113^ | NA | Berg Balance Scale, score | walking test, Gait Velocity | NA | NA | NA | NA | NA | NA | NA | Activity Specific Balance Confidence scale, score |
| Leal, Abrahin ^114^ | NA | Tinetti mobility test, score | 6-m walking velocity test, velocity | NA | NA | NA | NA | NA | 2-min step test, repetitions | NA | NA |
| Lee, Kim ^115^ | UPDRS motor, score | Berg Balance Scale, score | NA | NA | Beck Depression Inventory, score | NA | NA | NA | NA | NA | NA |
| Lei, Ma ^116^ | NA | Berg Balance Scale, score | NA | NA | NA | NA | NA | NA | NA | NA | NA |
| Li, Harmer ^117^ | UPDRS motor, score | NA | 4.3m walking test, gait velocity | NA | NA | NA | NA | NA | NA | Peak torque knee extension, Nm | NA |
| Liao, Yang ^118^ | NA | NA | 10m walking test, velocity | NA | NA | NA | NA | NA | NA | NA | Falls Efficacy Scale International, score |
| Liu, Yan ^119^ | NA | Berg Balance Scale, score | NA | NA | NA | NA | NA | NA | NA | NA | NA |
| Löfgren, Conradsson ^120^ | NA | NA | 8.3m walking test, gait velocity | NA | NA | NA | NA | NA | NA | NA | NA |
| Maidan, Nieuwhof ^121^ | NA | NA | walking test, Gait velocity | NA | NA | NA | NA | NA | NA | NA | NA |
| Marumoto, Yokoyama ^122^ | NA | NA | NA | NA | NA | NA | NA | PDQ 39-cognitive | NA | NA | NA |
| McNeely, Mai ^123^ | UPDRS motor, score | Mini-Balance Evaluation Systems Test, score | walking test, velocity | NA | NA | NA | NA | NA | 6 min walking test, m | NA | NA |
| Medijainen, Pääsuke ^124^ | NA | NA | 3-meter gait test, velocity | Freezing of Gait Questionnaire | NA | NA | NA | NA | NA | NA | NA |
| Michels, Dubaz ^125^ | UPDRS motor, score | Berg Balance Scale, score | NA | NA | Beck Depression Inventory, score | NA | NA | Montreal Cognitive Assessment, score | NA | NA | NA |
| Miyai, Fujimoto ^126^ | UPDRS motor, score | NA | 10m walking test, time | NA | NA | NA | NA | NA | NA | NA | NA |
| Mollinedo-Cardalda, Cancela-Carral ^127^ | UPDRS motor, score | NA | NA | NA | NA | NA | NA | NA | NA | NA | NA |
| Monticone, Ambrosini ^128^ | MDSUPDRS motor, score | Berg Balance Scale, score | NA | NA | NA | NA | NA | PDQ-39, Cognitive | NA | NA | NA |
| Moon, Sarmento ^129^ | UPDRS motor, score | NA | NA | NA | Geriatric Depression Scale, score | Geriatric Anxiety Inventory, score | PD Sleep Scale, score | Frontal assessment battery, score | NA | NA | NA |
| Moon, Schmidt ^130^ | NA | NA | NA | NA | NA | NA | PD Sleep Scale, score | NA | NA | NA | NA |
| Morris, Iansek ^131^ | UPDRS motor, score | NA | 10m walking test, velocity | NA | NA | NA | NA | NA | 2 min walk test, m; | NA | NA |
| Morris, Menz ^132^ | UPDRS motor, score | NA | 6-m walk test, velocity | NA | NA | NA | NA | NA | NA | NA | NA |
| Morris, Taylor ^133^ | UPDRS motor, score | NA | NA | NA | NA | NA | NA | NA | NA | NA | NA |
| Morrone, Miccinilli ^134^ | NA | NA | Walking Test, velocity | NA | NA | NA | NA | NA | NA | NA | Fall Risk Index, score |
| Myers, Harrison ^135^ | NA | Balance Evaluation Systems Test, score | NA | NA | NA | Beck anxiety inventory, score | NA | NA | NA | NA | NA |
| Nadeau, Pourcher ^136^ | UPDRS motor, score | NA | NA | NA | Beck Depression Inventory, score | NA | NA | PDQ-39, Cognition | 6-min walk test, m | NA | Activity Specific Balance Confidence scale, score |
| Ni, Mooney ^137^ | UPDRS motor, score | NA | NA | NA | NA | NA | NA | PDQ-39 cognition, score | NA | leg press, kg | NA |
| Ni, Signorile ^138^ | UPDRS bradykinesia, score | NA | NA | NA | NA | NA | NA | PDQ-39, Cognition | NA | Leg press, kg | NA |
| Ni, Signorile ^139^ | UPDRS motor, score | Berg Balance Scale, score | 10m walking test, gait velocity | NA | NA | NA | NA | NA | NA | leg press, kg/body weight | NA |
| Nieuwboer, Kwakkel ^140^ | NA | NA | 10m walking test, gait velocity | Freezing of Gait Questionnaire, score | NA | NA | NA | NA | NA | NA | Falls Efficacy Scale, score |
| Nocera, Amano ^141^ | NA | NA | NA | NA | NA | NA | NA | stroop color word, score | NA | NA | Falls Efficacy Scale International, score |
| Oliveira, Iraci ^142^ | NA | Berg Balance Scale, score | NA | NA | NA | NA | NA | NA | 6 min walk test, m | NA | NA |
| Ortiz-Rubio, Cabrera-Martos ^143^ | NA | Mini-Balance Evaluation Systems Test, score | NA | NA | NA | NA | NA | Piper Fatigue Scale, Cognitive | NA | NA | NA |
| Palamara, Gotti ^144^ | UPDRS motor, score | Berg Balance Scale, score | NA | NA | NA | NA | NA | NA | NA | NA | NA |
| Paolucci, Zangrando ^145^ | UPDRS motor, score | Berg Balance Scale, score | NA | NA | NA | NA | NA | NA | 6 min walk test, m | NA | NA |
| Park, Zid ^146^ | UPDRS motor, score | Tinetti Mobility Test, score | NA | NA | NA | NA | NA | NA | NA | NA | NA |
| Paul, Canning ^147^ | NA | NA | 10m walking test, gait velocity | Freezing of Gait Questionnaire, score | NA | NA | NA | NA | NA | leg extension, 1RM | NA |
| Pazzaglia, Imbimbo ^148^ | NA | Berg Balance Scale, score | NA | NA | NA | NA | NA | NA | NA | NA | NA |
| Pelosin, Cerulli ^149^ | UPDRS motor, score | NA | NA | NA | NA | NA | NA | NA | NA | NA | NA |
| Pérez de la Cruz ^150^ | UPDRS motor, score | Berg Balance Scale, score | NA | NA | NA | NA | NA | NA | NA | NA | NA |
| Pérez-de la Cruz ^151^ | NA | NA | NA | NA | Geriatric Depression Scale, score | NA | NA | NA | NA | NA | NA |
| Pérez-de la Cruz ^152^ | NA | NA | NA | NA | Geriatric Depression Scale | NA | NA | NA | NA | NA | NA |
| Picelli, Melotti ^153^ | UPDRS motor, score | Berg Balance Scale, score; | 10m walking test, time | NA | NA | NA | NA | NA | NA | NA | Activity Specific Balance Confidence scale, score |
| Picelli, Melotti ^154^ | NA | Berg Balance Scale, score | 10m walking test, velocity | NA | NA | NA | NA | NA | 6 min walk test, m | NA | NA |
| Picelli, Melotti ^155^ | UPDRS motor, score | Berg Balance Scale, score | NA | NA | NA | NA | NA | NA | NA | NA | Activity Specific Balance Confidence scale |
| Picelli, Varalta ^156^ | NA | NA | 10m walking test, time | NA | Beck Depression Inventory, score | NA | NA | Montreal Cognitive Assessment, score | 6 min walk test, m | NA | NA |
| Pohl, Dizdar ^157^ | UPDRS motor, score | NA | NA | NA | NA | NA | NA | Stroop Color-Word test (s) | NA | NA | NA |
| Pohl, Wressle ^158^ | NA | Mini-Balance Evaluation Systems Test, score | NA | Freezing of Gait Questionnaire, score | NA | NA | NA | Montreal Cognitive Assessment, score | NA | NA | Falls Efficacy Scale International, score |
| Poier, Rodrigues Recchia ^159^ | NA | NA | NA | NA | NA | NA | NA | PDQ-39 cognition, score | NA | NA | NA |
| Poliakoff, Galpin ^160^ | UPDRS motor, score | NA | NA | NA | NA | NA | NA | NA | NA | NA | NA |
| Pompeu, Mendes ^161^ | UPDRS motor, score | Berg Balance Scale, score | NA | NA | NA | NA | NA | Montreal Cognitive Assessment, score | NA | NA | NA |
| Prodoehl, Rafferty ^162^ | NA | Berg Balance Scale, score ＃ | Walking Test, velocity ＃ | NA | NA | NA | NA | NA | 6-Minute Walk Test, m ＃ | NA | NA |
| Protas, Mitchell ^163^ | NA | NA | 3m walking test, velocity | NA | NA | NA | NA | NA | NA | NA | NA |
| Qutubuddin, Cifu ^164^ | NA | Berg Balance Scale, score | NA | NA | NA | NA | NA | NA | NA | NA | NA |
| Rafferty, Prodoehl ^165^ | NA | NA | walking test, step velocity ＃ | NA | NA | NA | NA | NA | NA | Plantarflexion, Nm ＃ | NA |
| Rennie, Opheim ^166^ | NA | NA | 10m walking test, gait velocity | NA | NA | NA | NA | NA | NA | NA | NA |
| Ribas, Alves da Silva ^167^ | NA | Berg Balance Scale, score | NA | NA | NA | NA | NA | PDQ-39 cognition, score | 6-min walk test, m | NA | NA |
| Ridgel and Ault ^168^ | UPDRS motor scores | NA | NA | NA | NA | NA | NA | NA | NA | NA | NA |
| Rios Romenets, Anang ^169^ | UPDRS motor, score | Mini-Balance Evaluation Systems Test, score | NA | Freezing of Gait Questionnaire, score | Beck Depression Inventory, score | NA | NA | Montreal Cognitive Assessment, score | NA | NA | NA |
| Rocha, Aguiar ^170^ | UPDRS motor, score | Berg Balance Scale, score | NA | Freezing of Gait Questionnaire, score | NA | NA | NA | NA | NA | NA | NA |
| Sacheli, Neva ^171^ | MDSUPDRS motor, score | NA | NA | NA | Beck Depression Inventory, score | NA | NA | Montreal Cognitive Assessment, score | NA | maximum power, W | NA |
| Sage and Almeida ^172^ | UPDRS motor, score | NA | 4m walking test, Velocity | NA | NA | NA | NA | NA | NA | NA | NA |
| Sale, De Pandis ^173^ | UPDRS motor, score | NA | 10 meter walking test, velocity | NA | NA | NA | NA | NA | NA | NA | NA |
| San Martín Valenzuela, Moscardó ^174^ | NA | NA | 10m walking test, velocity | NA | NA | NA | NA | Executive cognitive function, TMTa | NA | NA | NA |
| Sangarapillai, Norman ^175^ | UPDRS motor, score | NA | walking test, velocity | NA | NA | NA | NA | NA | NA | NA | NA |
| Santos, da Silva ^176^ | NA | Balance Evaluation Systems Test, score | NA | NA | NA | NA | NA | NA | NA | NA | NA |
| Santos, Fernandez-Rio ^177^ | NA | NA | NA | Freezing of Gait Questionnaire, score | NA | NA | NA | NA | NA | NA | Falls Efficacy Scale International, score |
| Santos, Fernandez-Rio ^178^ | MDSUPDRS, score | NA | 10m walking test, velocity | freezing of gait questionnaire, score | NA | NA | NA | NA | NA | NA | NA |
| Santos, Machado ^179^ | NA | Berg Balance Scale, score | NA | NA | NA | NA | NA | NA | NA | NA | NA |
| Schabrun, Lamont ^180^ | NA | NA | 8m walking test, gait velocity | NA | NA | NA | NA | NA | NA | NA | NA |
| Schenkman, Cutson ^181^ | NA | NA | 10m walking test, time | NA | NA | NA | NA | NA | 6 min walk test, m | NA | NA |
| Schenkman, Hall ^182^ | UPDRS motor, score | NA | NA | NA | NA | NA | NA | NA | NA | NA | NA |
| Schenkman, Moore ^183^ | UPDRS motor, score ＃ | NA | NA | NA | NA | NA | NA | NA | NA | NA | NA |
| Schilling, Pfeiffer ^184^ | NA | NA | NA | NA | NA | NA | NA | NA | 6 min walk test, m; | leg press, kg/body weight | Activity Specific Balance Confidence scale, score |
| Schlenstedt, Paschen ^185^ | NA | Fullerton Advanced Balance, score | NA | Freezing of Gait Questionnaire, score | NA | NA | NA | NA | NA | NA | NA |
| Schlenstedt, Paschen ^186^ | UPDRS motor, score | Fullerton Advanced Balance, score | 5m walking test, velocity | NA | NA | NA | NA | NA | NA | leg press, Nm | NA |
| Schlick, Ernst ^187^ | UPDRS motor, score | NA | walking test, velocity | Freezing of Gait, score | NA | NA | NA | NA | NA | NA | NA |
| Sedaghati, Goudarzian ^188^ | NA | NA | NA | Freezing of Gait Questionnaire, score | NA | NA | NA | NA | NA | NA | Falls Efficacy Scale International, score |
| Serrao, Pierelli ^189^ | UPDRS motor, score | NA | walking test, velocity | NA | NA | NA | NA | NA | NA | NA | NA |
| Shahmohammadi, Sharifi ^190^ | NA | NA | NA | NA | NA | NA | NA | NA | NA | NA | NA |
| Shen and Mak ^191^ | NA | NA | Gait velocity | NA | NA | NA | NA | NA | NA | NA | NA |
| Shen and Mak ^192^ | UPDRS motor, score | NA | 5-m walk test, velocity | NA | NA | NA | NA | NA | NA | NA | NA |
| Shih, Wang ^193^ | NA | Berg Balance Scale, score | NA | NA | NA | NA | NA | NA | NA | NA | NA |
| Shulman, Katzel ^194^ | UPDRS motor, score | NA | 10m walking test, time | NA | Beck Depression Inventory, score | NA | NA | NA | 6 min walking test, m | leg press, kg | Falls Efficacy Scale International, score |
| Silva and Israel ^195^ | NA | Berg Balance Scale, score | NA | NA | NA | NA | NA | NA | NA | NA | NA |
| Silva-Batista, Corcos ^196^ | NA | NA | NA | NA | NA | NA | NA | NA | NA | peak torque knee extensor, Nm | NA |
| Silva-Batista, Corcos ^197^ | NA | Balance Evaluation Systems Test, score | NA | NA | NA | NA | NA | Montreal Cognitive Assessment, score | NA | NA | Falls Efficacy Scale International, score |
| Silva-Batista, Corcos ^198^ | UPDRS motor, score | NA | NA | NA | NA | NA | NA | Montreal Cognitive Assessment, score | NA | Leg press, kg | NA |
| Silva-Batista, de Brito ^199^ | NA | NA | NA | NA | NA | NA | PD Sleep Scale, score | NA | NA | NA | NA |
| Silva-Batista, de Lima-Pardini ^200^ | UPDRS motor, score | NA | NA | New Freezing of Gait Questionnaire, score | NA | NA | NA | Stroop-III test, score | NA | NA | NA |
| Smania, Corato ^201^ | NA | Berg Balance Scale, score | NA | NA | Geriatric Depression Scale, score | NA | NA | NA | NA | NA | Activity Specific Balance Confidence scale |
| Solla, Cugusi ^202^ | UPDRS motor, score | Berg Balance Scale, score | 7m walking test, velocity | NA | Beck Depression Inventory, score | NA | NA | Montreal Cognitive Assessment, score | 6 min walking test, m | NA | NA |
| Song, Paul ^203^ | NA | NA | 6m walking test, velocity | NA | NA | NA | NA | Montreal Cognitive Assessment, score | NA | NA | Falls Efficacy Scale International, score |
| Steib, Klamroth ^204^ | NA | Mini-Balance Evaluation Systems Test, score | 6m walking test, velocity | NA | NA | NA | NA | NA | 2-min walking test, m | NA | Activity Specific Balance Confidence scale, score |
| Steib, Klamroth ^205^ | NA | NA | 1 min walking test, velocity | NA | NA | NA | NA | NA | NA | NA | NA |
| Stożek, Rudzińska ^206^ | NA | NA | 10m walking test, time | NA | NA | NA | NA | NA | NA | NA | NA |
| Strand, Cherup ^207^ | UPDRS motor, score | Mini-Balance Evaluation Systems Test, score | NA | NA | NA | NA | NA | NA | NA | Leg press, kg | NA |
| Strouwen, Molenaar ^208^ | NA | NA | 7.92m walking test, velocity | NA | NA | NA | NA | NA | NA | NA | NA |
| Stuckenschneider, Helmich ^209^ | NA | NA | walking test, velocity | NA | NA | NA | NA | NA | NA | NA | NA |
| Tang, Peng ^210^ | NA | Berg Balance Scale, score | NA | NA | NA | NA | NA | NA | NA | NA | NA |
| Teixeira-Machado, Araújo ^211^ | NA | NA | NA | NA | Beck Depression Inventory, score | NA | NA | NA | NA | NA | NA |
| Terrens, Soh ^212^ | UPDRS motor, score | mini-Balance Evaluation Systems Test, score | NA | NA | NA | NA | NA | NA | NA | NA | Modified Falls Efficacy Scale, score |
| Thaut, McIntosh ^213^ | NA | NA | walking test, velocity | NA | NA | NA | NA | NA | NA | NA | NA |
| Tollár, Nagy ^214^ | UPDRS motor, score | Mini-Balance Evaluation Systems Test, score | NA | NA | Beck Depression Inventory, score | NA | NA | NA | 6 min walk test, m | NA | NA |
| Tollár, Nagy ^215^ | UPDRS motor, score | NA | NA | NA | Beck Depression Inventory, score | NA | NA | NA | NA | NA | NA |
| Tollár, Nagy ^216^ | MDSUPDRS, score | NA | NA | NA | Beck Depression Index, score | NA | NA | NA | NA | NA | NA |
| Tramontano, Bonnì ^217^ | UPDRS motor, score | NA | NA | NA | NA | NA | NA | NA | NA | NA | NA |
| van den Heuvel, Kwakkel ^218^ | UPDRS motor, score | Berg Balance Scale, score | 10m walking test, velocity | NA | hospital anxiety and depression scale-depression, score | hospital anxiety and depression scale-anxiety, score | NA | NA | NA | NA | Falls Efficacy Scale International, score |
| van der Kolk, de Vries ^219^ | UPDRS motor, score ＃ | Mini-Balance Evaluation Systems Test, score | NA | NA | hospital anxiety and depression scale-depression, score | hospital anxiety and depression scale-anxiety, score | PD Sleep Scale, night score | Montreal Cognitive Assessment, score | 6 min walking test, m | NA | number of falls |
| van der Kolk, de Vries ^220^ | UPDRS motor, score | NA | NA | NA | hospital anxiety and depression scale-depression, score | hospital anxiety and depression scale-anxiety, score | PD Sleep Scale, score | PD cognition scale, score | NA | NA | NA |
| van Nimwegen, Speelman ^221^ | NA | NA | NA | NA | NA | NA | NA | NA | 6 min walking test, m | NA | NA |
| Van Puymbroeck, Walter ^222^ | UPDRS motor, score | Mini-Balance Evaluation Systems Test, score | NA | Freezing of Gait Questionnaire, score | NA | NA | NA | NA | NA | NA | NA |
| Vanbellingen, Nyffeler ^223^ | UPDRS motor, score | NA | NA | NA | NA | NA | NA | NA | NA | NA | NA |
| Vergara-Diaz, Osypiuk ^224^ | UPDRS motor, score | NA | 15m walking test, velocity | NA | NA | NA | NA | Executive cognitive function, score | NA | NA | Activity Specific Balance Confidence scale, score |
| Vieira de Moraes Filho, Chaves ^225^ | NA | NA | 10m walking test, velocity | NA | NA | NA | NA | NA | NA | NA | NA |
| Vieira-Yano, Martini ^226^ | NA | NA | walking test, velocity | NA | NA | NA | NA | NA | NA | NA | NA |
| Vitório, Teixeira-Arroyo ^227^ | UPDRS, motor, score | NA | 8m walking test, velocity | NA | NA | NA | NA | Mini-Exam of Mental Status | NA | NA | NA |
| Vivas, Arias ^228^ | UPDRS motor, score | Berg Balance Scale, score | NA | NA | NA | NA | NA | NA | NA | NA | NA |
| Volpe, Giantin ^229^ | UPDRS motor, score | Berg Balance Scale, score | NA | NA | NA | NA | NA | NA | NA | NA | Falls Efficacy Scale, score |
| Volpe, Giantin ^230^ | UPDRS motor, score | Berg Balance Scale, score | NA | NA | NA | NA | NA | NA | NA | NA | Falls Efficacy Scale International, score |
| Volpe, Giantin ^231^ | UPDRS motor, score | Berg Balance Scale, score | NA | NA | NA | NA | NA | NA | NA | NA | Falls Efficacy Scale International, score |
| Volpe, Signorini ^232^ | UPDRS motor, score | Berg Balance Scale, score | NA | freezing of gait questionnaire, score | NA | NA | NA | NA | NA | NA | NA |
| Wallén, Hagströmer ^233^ | UPDRS motor, score | Mini-Balance Evaluation Systems Test, score | 9m walking test, gait velocity | NA | NA | NA | NA | NA | NA | NA | NA |
| Wang, Bi ^234^ | UPDRS motor, score | NA | NA | NA | NA | NA | NA | NA | NA | NA | NA |
| White, Wagenaar ^235^ | NA | NA | 10s walking test, velocity | NA | NA | NA | NA | NA | 2 min walk test, m | NA | NA |
| Wong-Yu and Mak ^236^ | NA | Balance Evaluation Systems Test total, score | 7m walking test, Gait velocity | NA | NA | NA | NA | NA | NA | NA | Activity Specific Balance Confidence scale, score |
| Wong-Yu and Mak ^237^ | NA | Mini-Balance Evaluation Systems Test, score | NA | NA | NA | NA | NA | NA | NA | NA | NA |
| Xiao and Zhuang ^238^ | UPDRS motor, score | Berg Balance Scale, score | walking test, velocity | Freezing of Gait Questionnaire, score | NA | NA | PD Sleep Scale, score | NA | 6-min walk test, m | NA | NA |
| Xiao, Zhuang ^239^ | UPDRS motor, score | Berg Balance Scale, score | 6 min walking test, velocity | Freezing of Gait Questionnaire, score | NA | NA | NA | NA | 6 min walk test, m | knee extension strength, kg | Activity Specific Balance Confidence scale, score |
| Yang, Cheng ^240^ | NA | NA | walking test, velocity | Freezing of Gait Questionnaire, score | NA | NA | NA | NA | NA | NA | Falls Efficacy Scale International, score |
| Yang, Lee ^241^ | NA | NA | 3.66m walking test, velocity | NA | NA | NA | NA | NA | NA | knee extensor, N | NA |
| Yang, Wang ^242^ | UPDRS motor, score | Berg Balance Scale, score | NA | NA | NA | NA | NA | NA | NA | NA | NA |
| Yi-zhao, Hua ^243^ | UPDRS motor, score | Berg Balance Scale, score | 10m walking test, velocity | NA | NA | NA | NA | NA | 6 min walk test, m | NA | NA |
| Yotnuengnit, Bhidayasiri ^244^ | UPDRS motor, scores | NA | walking test, Gait velocity | NA | NA | NA | NA | NA | NA | NA | NA |
| You and She ^245^ | UPDRS motor, score | Berg Balance Scale, score | NA | NA | hospital anxiety and depression scale-depression, score | NA | NA | NA | NA | NA | NA |
| Youm, Kim ^246^ | UPDRS motor, score | NA | Walk Test, Step velocity | NA | NA | NA | NA | Mini-Mental State Examination, score | NA | NA | NA |
| Zeng, Tian ^247^ | UPDRS motor, score | Berg Balance Scale, score | NA | NA | NA | NA | NA | NA | 6 min walk test, m | NA | NA |
| Zhang, Hu ^248^ | UPDRS motor, score | Berg Balance Scale, score | 10m walking test, velocity | NA | NA | NA | NA | NA | NA | NA | NA |
| Zhang, Zhou ^249^ | NA | NA | NA | NA | NA | NA | NA | NA | 6 min walk test, m | NA | NA |
| Zheng, Zheng ^250^ | NA | NA | NA | NA | hospital anxiety and depression scale-depression, score | hospital anxiety and depression scale-anxiety, score | Parkinson’s Disease Sleep Scale, score | NA | NA | NA | NA |

*NA* not available, *＃* outcomes evaluation in ON and OFF states, *AE* Aerobic Exercise, *AQE* Aquatic Exercise, *BGT* Balance and Gait Training, *BGT_ECA* Balance and Gait Training with External Cue or Attention, *BGT_ICA* Balance and Gait Training with Internal Cue or Attention, *BWS_TT* Body Weight Support Treadmill Training, *CON* Control group, *CPP* Classic Physiotherapy Program, *DT_BGT* Dual Task Balance and Gait Training, *Mul_C* Multicomponent Exercise Program, *Mul_D* Multidisciplinary Exercise Program, *NW* Nordic Walking, *PT* Power Training, *RA_GT* Robotic Assisted Gait Training, *RT* Resistance Training, *TC* Tai Chi, *TT* Treadmill Training, *VR* Virtual Reality, *WBV* Whole Body Vibration.

## 8.3 List of included studies

1. Abraham A, Hart A, Andrade I, Hackney ME. Dynamic Neuro-Cognitive Imagery Improves Mental Imagery Ability, Disease Severity, and Motor and Cognitive Functions in People with Parkinson's Disease. *Neural Plast* 2018; **2018**: 6168507.

2. Ajimsha MS, Majeed NA, Chinnavan E, Thulasyammal RP. Effectiveness of autogenic training in improving motor performances in Parkinson's disease. *Complement Ther Med* 2014; **22**(3): 419-25.

3. Allen NE, Canning CG, Sherrington C, et al. The effects of an exercise program on fall risk factors in people with Parkinson's disease: a randomized controlled trial. *Mov Disord* 2010; **25**(9): 1217-25.

4. Allen NE, Song J, Paul SS, et al. An interactive videogame for arm and hand exercise in people with Parkinson's disease: A randomized controlled trial. *Parkinsonism Relat Disord* 2017; **41**: 66-72.

5. Altmann LJP, Stegemöller E, Hazamy AA, et al. Aerobic Exercise Improves Mood, Cognition, and Language Function in Parkinson's Disease: Results of a Controlled Study. *J Int Neuropsychol Soc* 2016; **22**(9): 878-89.

6. Amano S, Nocera JR, Vallabhajosula S, et al. The effect of Tai Chi exercise on gait initiation and gait performance in persons with Parkinson's disease. *Parkinsonism Relat Disord* 2013; **19**(11): 955-60.

7. Amara AW, Wood KH, Joop A, et al. Randomized, Controlled Trial of Exercise on Objective and Subjective Sleep in Parkinson's Disease. *Mov Disord* 2020; **35**(6): 947-58.

8. Arcolin I, Pisano F, Delconte C, et al. Intensive cycle ergometer training improves gait speed and endurance in patients with Parkinson's disease: A comparison with treadmill training. *Restor Neurol Neurosci* 2016; **34**(1): 125-38.

9. Arfa-Fatollahkhani P, Safar Cherati A, Habibi SAH, Shahidi GA, Sohrabi A, Zamani B. Effects of treadmill training on the balance, functional capacity and quality of life in Parkinson's disease: A randomized clinical trial. *J Complement Integr Med* 2019; **17**(1).

10. Ashburn A, Fazakarley L, Ballinger C, Pickering R, McLellan LD, Fitton C. A randomised controlled trial of a home based exercise programme to reduce the risk of falling among people with Parkinson's disease. *J Neurol Neurosurg Psychiatry* 2007; **78**(7): 678-84.

11. Atan T, Özyemişci Taşkıran Ö, Bora Tokçaer A, Kaymak Karataş G, Karakuş Çalışkan A, Karaoğlan B. Effects of different percentages of body weight-supported treadmill training in Parkinson’s disease: a double-blind randomized controlled trial. *Turk J Med Sci* 2019; **49**(4).

12. Bakhshayesh B, Sayyar S, Daneshmandi H. Pilates Exercise and Functional Balance in Parkinson's Disease. *Caspian Journal of Neurological Sciences* 2017; **3**(1): 25-38.

13. Bang D-H, Shin W-S. Effects of an intensive Nordic walking intervention on the balance function and walking ability of individuals with Parkinson's disease: a randomized controlled pilot trial. *Aging Clin Exp Res* 2017; **29**(5): 993-9.

14. Barboza NM, Terra MB, Bueno MEB, Christofoletti G, Smaili SM. Physiotherapy Versus Physiotherapy Plus Cognitive Training on Cognition and Quality of Life in Parkinson Disease: Randomized Clinical Trial. *Am J Phys Med Rehabil* 2019; **98**(6): 460-8.

15. Beck EN, Intzandt BN, Almeida QJ. Can Dual Task Walking Improve in Parkinson's Disease After External Focus of Attention Exercise? A Single Blind Randomized Controlled Trial. *Neurorehabil Neural Repair* 2018; **32**(1): 18-33.

16. Beck EN, Wang MTY, Intzandt BN, Almeida QJ, Ehgoetz Martens KA. Sensory focused exercise improves anxiety in Parkinson's disease: A randomized controlled trial. *PLoS One* 2020; **15**(4): e0230803.

17. Bekkers EMJ, Mirelman A, Alcock L, et al. Do Patients With Parkinson's Disease With Freezing of Gait Respond Differently Than Those Without to Treadmill Training Augmented by Virtual Reality? *Neurorehabil Neural Repair* 2020; **34**(5): 440-9.

18. Bello O, Sanchez JA, Lopez-Alonso V, et al. The effects of treadmill or overground walking training program on gait in Parkinson's disease. *Gait Posture* 2013; **38**(4): 590-5.

19. Burini D, Farabollini B, Iacucci S, et al. A randomised controlled cross-over trial of aerobic training versus Qigong in advanced Parkinson's disease. *Eura Medicophys* 2006; **42**(3): 231-8.

20. Cabrera-Martos I, Jiménez-Martín AT, López-López L, Rodríguez-Torres J, Ortiz-Rubio A, Valenza MC. Effects of a core stabilization training program on balance ability in persons with Parkinson's disease: a randomized controlled trial. *Clin Rehabil* 2020; **34**(6): 764-72.

21. Cakit BD, Saracoglu M, Genc H, Erdem HR, Inan L. The effects of incremental speed-dependent treadmill training on postural instability and fear of falling in Parkinson's disease. *Clin Rehabil* 2007; **21**(8): 698-705.

22. Calabrò RS, Naro A, Filoni S, et al. Walking to your right music: a randomized controlled trial on the novel use of treadmill plus music in Parkinson's disease. *J Neuroeng Rehabil* 2019; **16**(1): 68.

23. Cancela JM, Mollinedo I, Montalvo S, Vila Suárez ME. Effects of a High-Intensity Progressive-Cycle Program on Quality of Life and Motor Symptomatology in a Parkinson's Disease Population: A Pilot Randomized Controlled Trial. *Rejuvenation Res* 2020; **23**(6): 508-15.

24. Canning CG, Allen NE, Dean CM, Goh L, Fung VSC. Home-based treadmill training for individuals with Parkinson's disease: a randomized controlled pilot trial. *Clin Rehabil* 2012; **26**(9): 817-26.

25. Capato TTC, de Vries NM, IntHout J, Barbosa ER, Nonnekes J, Bloem BR. Multimodal Balance Training Supported by Rhythmical Auditory Stimuli in Parkinson's Disease: A Randomized Clinical Trial. *J Parkinsons Dis* 2020; **10**(1): 333-46.

26. Capato TTC, Nonnekes J, de Vries NM, IntHout J, Barbosa ER, Bloem BR. Effects of multimodal balance training supported by rhythmical auditory stimuli in people with advanced stages of Parkinson's disease: a pilot randomized clinical trial. *J Neurol Sci* 2020; **418**: 117086.

27. Capecci M, Pournajaf S, Galafate D, et al. Clinical effects of robot-assisted gait training and treadmill training for Parkinson's disease. A randomized controlled trial. *Ann Phys Rehabil Med* 2019; **62**(5): 303-12.

28. Capecci M, Serpicelli C, Fiorentini L, et al. Postural rehabilitation and Kinesio taping for axial postural disorders in Parkinson's disease. *Arch Phys Med Rehabil* 2014; **95**(6): 1067-75.

29. Carda S, Invernizzi M, Baricich A, Comi C, Croquelois A, Cisari C. Robotic gait training is not superior to conventional treadmill training in parkinson disease: a single-blind randomized controlled trial. *Neurorehabil Neural Repair* 2012; **26**(9): 1027-34.

30. Carpinella I, Cattaneo D, Bonora G, et al. Wearable Sensor-Based Biofeedback Training for Balance and Gait in Parkinson Disease: A Pilot Randomized Controlled Trial. *Arch Phys Med Rehabil* 2017; **98**(4).

31. Carroll LM, Volpe D, Morris ME, Saunders J, Clifford AM. Aquatic Exercise Therapy for People With Parkinson Disease: A Randomized Controlled Trial. *Arch Phys Med Rehabil* 2017; **98**(4): 631-8.

32. Carvalho A, Barbirato D, Araujo N, et al. Comparison of strength training, aerobic training, and additional physical therapy as supplementary treatments for Parkinson's disease: pilot study. *Clin Interv Aging* 2015; **10**: 183-91.

33. Cheng F-Y, Yang Y-R, Chen L-M, Wu Y-R, Cheng S-J, Wang R-Y. Positive Effects of Specific Exercise and Novel Turning-based Treadmill Training on Turning Performance in Individuals with Parkinson's disease: A Randomized Controlled Trial. *Sci Rep* 2016; **6**: 33242.

34. Cheng F-Y, Yang Y-R, Wu Y-R, Cheng S-J, Wang R-Y. Effects of curved-walking training on curved-walking performance and freezing of gait in individuals with Parkinson's disease: A randomized controlled trial. *Parkinsonism Relat Disord* 2017; **43**: 20-6.

35. Cherup NP, Strand KL, Lucchi L, Wooten SV, Luca C, Signorile JF. Yoga Meditation Enhances Proprioception and Balance in Individuals Diagnosed With Parkinson's Disease. *Percept Mot Skills* 2021; **128**(1): 304-23.

36. Cheung C, Bhimani R, Wyman JF, et al. Effects of yoga on oxidative stress, motor function, and non-motor symptoms in Parkinson's disease: a pilot randomized controlled trial. *Pilot Feasibility Stud* 2018; **4**: 162.

37. Chivers Seymour K, Pickering R, Rochester L, et al. Multicentre, randomised controlled trial of PDSAFE, a physiotherapist-delivered fall prevention programme for people with Parkinson's. *J Neurol Neurosurg Psychiatry* 2019; **90**(7): 774-82.

38. Choi H-J. Effects of therapeutic Tai chi on functional fitness and activities of daily living in patients with Parkinson disease. *J Exerc Rehabil* 2016; **12**(5): 499-503.

39. Clarke CE, Patel S, Ives N, et al. Physiotherapy and Occupational Therapy vs No Therapy in Mild to Moderate Parkinson Disease: A Randomized Clinical Trial. *JAMA Neurol* 2016; **73**(3): 291-9.

40. Clerici I, Maestri R, Bonetti F, et al. Land Plus Aquatic Therapy Versus Land-Based Rehabilitation Alone for the Treatment of Freezing of Gait in Parkinson Disease: A Randomized Controlled Trial. *Phys Ther* 2019; **99**(5): 591-600.

41. Combs SA, Diehl MD, Chrzastowski C, et al. Community-based group exercise for persons with Parkinson disease: a randomized controlled trial. *NeuroRehabilitation* 2013; **32**(1): 117-24.

42. Conradsson D, Löfgren N, Nero H, et al. The Effects of Highly Challenging Balance Training in Elderly With Parkinson's Disease: A Randomized Controlled Trial. *Neurorehabil Neural Repair* 2015; **29**(9): 827-36.

43. Corcos DM, Robichaud JA, David FJ, et al. A two-year randomized controlled trial of progressive resistance exercise for Parkinson's disease. *Mov Disord* 2013; **28**(9): 1230-40.

44. Costa-Ribeiro A, Maux A, Bosford T, et al. Transcranial direct current stimulation associated with gait training in Parkinson's disease: A pilot randomized clinical trial. *Dev Neurorehabil* 2017; **20**(3): 121-8.

45. Cugusi L, Solla P, Serpe R, et al. Effects of a Nordic Walking program on motor and non-motor symptoms, functional performance and body composition in patients with Parkinson's disease. *NeuroRehabilitation* 2015; **37**(2): 245-54.

46. Daneshmandi H, Sayyar S, Bakhshayesh B. The effect of a selective Pilates program on functional balance and falling risk in patients with Parkinson’s disease. *Zahedan Journal of Research in Medical Sciences* 2017; **19**(4).

47. Dashtipour K, Johnson E, Kani C, et al. Effect of exercise on motor and nonmotor symptoms of Parkinson's disease. *Parkinsons Dis* 2015; **2015**: 586378.

48. David FJ, Robichaud JA, Leurgans SE, et al. Exercise improves cognition in Parkinson's disease: The PRET-PD randomized, clinical trial. *Mov Disord* 2015; **30**(12): 1657-63.

49. David FJ, Robichaud JA, Vaillancourt DE, et al. Progressive resistance exercise restores some properties of the triphasic EMG pattern and improves bradykinesia: the PRET-PD randomized clinical trial. *J Neurophysiol* 2016; **116**(5): 2298-311.

50. de Bruin N, Doan JB, Turnbull G, et al. Walking with music is a safe and viable tool for gait training in Parkinson's disease: the effect of a 13-week feasibility study on single and dual task walking. *Parkinsons Dis* 2010; **2010**: 483530.

51. de Lima TA, Ferreira-Moraes R, Alves WMGdC, et al. Resistance training reduces depressive symptoms in elderly people with Parkinson disease: A controlled randomized study. *Scand J Med Sci Sports* 2019; **29**(12): 1957-67.

52. de Melo GEL, Kleiner AFR, Lopes JBP, et al. Effect of virtual reality training on walking distance and physical fitness in individuals with Parkinson's disease. *NeuroRehabilitation* 2018; **42**(4): 473-80.

53. Demonceau M, Maquet D, Jidovtseff B, et al. Effects of twelve weeks of aerobic or strength training in addition to standard care in Parkinson's disease: a controlled study. *Eur J Phys Rehabil Med* 2017; **53**(2): 184-200.

54. Dibble LE, Foreman KB, Addison O, Marcus RL, LaStayo PC. Exercise and medication effects on persons with Parkinson disease across the domains of disability: a randomized clinical trial. *J Neurol Phys Ther* 2015; **39**(2): 85-92.

55. Dibble LE, Hale TF, Marcus RL, Droge J, Gerber JP, LaStayo PC. High-intensity resistance training amplifies muscle hypertrophy and functional gains in persons with Parkinson's disease. *Mov Disord* 2006; **21**(9): 1444-52.

56. DiFrancisco-Donoghue J, Lamberg EM, Rabin E, Elokda A, Fazzini E, Werner WG. Effects of exercise and B vitamins on homocysteine and glutathione in Parkinson's disease: a randomized trial. *Neurodegener Dis* 2012; **10**(1-4): 127-34.

57. Dipasquale S, Meroni R, Sasanelli F, et al. Physical Therapy Versus a General Exercise Programme in Patients with Hoehn Yahr Stage II Parkinson's Disease: A Randomized Controlled Trial. *J Parkinsons Dis* 2017; **7**(1): 203-10.

58. Droby A, Maidan I, Jacob Y, Giladi N, Hausdorff JM, Mirelman A. Distinct Effects of Motor Training on Resting-State Functional Networks of the Brain in Parkinson's Disease. *Neurorehabil Neural Repair* 2020; **34**(9): 795-803.

59. Duncan RP, Earhart GM. Randomized controlled trial of community-based dancing to modify disease progression in Parkinson disease. *Neurorehabil Neural Repair* 2012; **26**(2): 132-43.

60. Ebersbach G, Ebersbach A, Edler D, et al. Comparing exercise in Parkinson's disease--the Berlin LSVT®BIG study. *Mov Disord* 2010; **25**(12): 1902-8.

61. Ebersbach G, Edler D, Kaufhold O, Wissel J. Whole body vibration versus conventional physiotherapy to improve balance and gait in Parkinson's disease. *Arch Phys Med Rehabil* 2008; **89**(3): 399-403.

62. Ellis T, de Goede CJ, Feldman RG, Wolters EC, Kwakkel G, Wagenaar RC. Efficacy of a physical therapy program in patients with Parkinson's disease: a randomized controlled trial. *Arch Phys Med Rehabil* 2005; **86**(4): 626-32.

63. El-Tamawy MS, Darwish MH, Khallaf ME. Effects of augmented proprioceptive cues on the parameters of gait of individuals with Parkinson's disease. *Ann Indian Acad Neurol* 2012; **15**(4): 267-72.

64. Feng H, Li C, Liu J, et al. Virtual Reality Rehabilitation Versus Conventional Physical Therapy for Improving Balance and Gait in Parkinson's Disease Patients: A Randomized Controlled Trial. *Med Sci Monit* 2019; **25**: 4186-92.

65. Fernandes Â, Rocha N, Santos R, Tavares JMRS. Effects of dual-task training on balance and executive functions in Parkinson's disease: A pilot study. *Somatosens Mot Res* 2015; **32**(2): 122-7.

66. Ferraz DD, Trippo KV, Duarte GP, Neto MG, Bernardes Santos KO, Filho JO. The Effects of Functional Training, Bicycle Exercise, and Exergaming on Walking Capacity of Elderly Patients With Parkinson Disease: A Pilot Randomized Controlled Single-blinded Trial. *Arch Phys Med Rehabil* 2018; **99**(5): 826-33.

67. Ferrazzoli D, Ortelli P, Zivi I, et al. Efficacy of intensive multidisciplinary rehabilitation in Parkinson's disease: a randomised controlled study. *J Neurol Neurosurg Psychiatry* 2018; **89**(8): 828-35.

68. Ferreira RM, Alves WMGdC, de Lima TA, et al. The effect of resistance training on the anxiety symptoms and quality of life in elderly people with Parkinson's disease: a randomized controlled trial. *Arq Neuropsiquiatr* 2018; **76**(8): 499-506.

69. Fietzek UM, Schroeteler FE, Ziegler K, Zwosta J, Ceballos-Baumann AO. Randomized cross-over trial to investigate the efficacy of a two-week physiotherapy programme with repetitive exercises of cueing to reduce the severity of freezing of gait in patients with Parkinson's disease. *Clin Rehabil* 2014; **28**(9): 902-11.

70. Fil-Balkan A, Salci Y, Keklicek H, et al. Sensorimotor integration training in Parkinson`s disease. *Neurosciences (Riyadh)* 2018; **23**(4): 208-15.

71. Fisher BE, Wu AD, Salem GJ, et al. The effect of exercise training in improving motor performance and corticomotor excitability in people with early Parkinson's disease. *Arch Phys Med Rehabil* 2008; **89**(7): 1221-9.

72. Franzoni LT, Monteiro EP, Oliveira HB, et al. A 9-Week Nordic and Free Walking Improve Postural Balance in Parkinson's Disease. *Sports Med Int Open* 2018; **2**(2): E28-E34.

73. Frazzitta G, Bertotti G, Riboldazzi G, et al. Effectiveness of intensive inpatient rehabilitation treatment on disease progression in parkinsonian patients: a randomized controlled trial with 1-year follow-up. *Neurorehabil Neural Repair* 2012; **26**(2): 144-50.

74. Frazzitta G, Bossio F, Maestri R, Palamara G, Bera R, Ferrazzoli D. Crossover versus Stabilometric Platform for the Treatment of Balance Dysfunction in Parkinson's Disease: A Randomized Study. *Biomed Res Int* 2015; **2015**: 878472.

75. Frazzitta G, Maestri R, Uccellini D, Bertotti G, Abelli P. Rehabilitation treatment of gait in patients with Parkinson's disease with freezing: a comparison between two physical therapy protocols using visual and auditory cues with or without treadmill training. *Mov Disord* 2009; **24**(8): 1139-43.

76. Frazzitta G, Maestri R, Ghilardi MF, et al. Intensive rehabilitation increases BDNF serum levels in parkinsonian patients: a randomized study. *Neurorehabil Neural Repair* 2014; **28**(2): 163-8.

77. Furnari A, Calabrò RS, De Cola MC, et al. Robotic-assisted gait training in Parkinson's disease: a three-month follow-up randomized clinical trial. *Int J Neurosci* 2017; **127**(11).

78. Galli M, Cimolin V, De Pandis MF, et al. Robot-assisted gait training versus treadmill training in patients with Parkinson's disease: a kinematic evaluation with gait profile score. *Funct Neurol* 2016; **31**(3): 163-70.

79. Gandolfi M, Geroin C, Dimitrova E, et al. Virtual Reality Telerehabilitation for Postural Instability in Parkinson's Disease: A Multicenter, Single-Blind, Randomized, Controlled Trial. *Biomed Res Int* 2017; **2017**: 7962826.

80. Gandolfi M, Tinazzi M, Magrinelli F, et al. Four-week trunk-specific exercise program decreases forward trunk flexion in Parkinson's disease: A single-blinded, randomized controlled trial. *Parkinsonism Relat Disord* 2019; **64**: 268-74.

81. Ganesan M, Sathyaprabha TN, Pal PK, Gupta A. Partial Body Weight-Supported Treadmill Training in Patients With Parkinson Disease: Impact on Gait and Clinical Manifestation. *Arch Phys Med Rehabil* 2015; **96**(9): 1557-65.

82. Gao Q, Leung A, Yang Y, et al. Effects of Tai Chi on balance and fall prevention in Parkinson's disease: a randomized controlled trial. *Clin Rehabil* 2014; **28**(8): 748-53.

83. Gaßner H, Steib S, Klamroth S, et al. Perturbation Treadmill Training Improves Clinical Characteristics of Gait and Balance in Parkinson's Disease. *J Parkinsons Dis* 2019; **9**(2): 413-26.

84. Ghielen I, van Wegen EEH, Rutten S, et al. Body awareness training in the treatment of wearing-off related anxiety in patients with Parkinson's disease: Results from a pilot randomized controlled trial. *J Psychosom Res* 2017; **103**: 1-8.

85. Ginis P, Nieuwboer A, Dorfman M, et al. Feasibility and effects of home-based smartphone-delivered automated feedback training for gait in people with Parkinson's disease: A pilot randomized controlled trial. *Parkinsonism Relat Disord* 2016; **22**: 28-34.

86. Gobbi LTB, Pelicioni PHS, Lahr J, Lirani-Silva E, Teixeira-Arroyo C, Santos PCRD. Effect of different types of exercises on psychological and cognitive features in people with Parkinson's disease: A randomized controlled trial. *Ann Phys Rehabil Med* 2021; **64**(1): 101407.

87. Goodwin VA, Richards SH, Henley W, Ewings P, Taylor AH, Campbell JL. An exercise intervention to prevent falls in people with Parkinson's disease: a pragmatic randomised controlled trial. *J Neurol Neurosurg Psychiatry* 2011; **82**(11): 1232-8.

88. Grobbelaar R, Venter R, Welman KE. Backward compared to forward over ground gait retraining have additional benefits for gait in individuals with mild to moderate Parkinson's disease: A randomized controlled trial. *Gait Posture* 2017; **58**: 294-9.

89. Hackney ME, Earhart GM. Effects of dance on movement control in Parkinson's disease: a comparison of Argentine tango and American ballroom. *J Rehabil Med* 2009; **41**(6): 475-81.

90. Hackney ME, Earhart GM. Tai Chi improves balance and mobility in people with Parkinson disease. *Gait Posture* 2008; **28**(3): 456-60.

91. Hackney ME, Earhart GM. Health-related quality of life and alternative forms of exercise in Parkinson disease. *Parkinsonism Relat Disord* 2009; **15**(9): 644-8.

92. Hackney ME, Kantorovich S, Levin R, Earhart GM. Effects of tango on functional mobility in Parkinson's disease: a preliminary study. *J Neurol Phys Ther* 2007; **31**(4): 173-9.

93. Harro CC, Shoemaker MJ, Frey O, et al. The effects of speed-dependent treadmill training and rhythmic auditory-cued overground walking on balance function, fall incidence, and quality of life in individuals with idiopathic Parkinson's disease: a randomized controlled trial. *NeuroRehabilitation* 2014; **34**(3): 541-56.

94. Hashimoto H, Takabatake S, Miyaguchi H, Nakanishi H, Naitou Y. Effects of dance on motor functions, cognitive functions, and mental symptoms of Parkinson's disease: a quasi-randomized pilot trial. *Complement Ther Med* 2015; **23**(2): 210-9.

95. Hass CJ, Collins MA, Juncos JL. Resistance training with creatine monohydrate improves upper-body strength in patients with Parkinson disease: a randomized trial. *Neurorehabil Neural Repair* 2007; **21**(2): 107-15.

96. Helgerud J, Thomsen SN, Hoff J, et al. Maximal strength training in patients with Parkinson's disease: impact on efferent neural drive, force-generating capacity, and functional performance. *J Appl Physiol (1985)* 2020; **129**(4): 683-90.

97. Hirsch MA, Toole T, Maitland CG, Rider RA. The effects of balance training and high-intensity resistance training on persons with idiopathic Parkinson's disease. *Arch Phys Med Rehabil* 2003; **84**(8): 1109-17.

98. Hubble RP, Naughton G, Silburn PA, Cole MH. Trunk Exercises Improve Gait Symmetry in Parkinson Disease: A Blind Phase II Randomized Controlled Trial. *Am J Phys Med Rehabil* 2018; **97**(3): 151-9.

99. Johansson ME, Cameron IGM, Van der Kolk NM, et al. Aerobic Exercise Alters Brain Function and Structure in Parkinson's Disease: A Randomized Controlled Trial. *Ann Neurol* 2022; **91**(2): 203-16.

100. Johansson H, Freidle M, Ekman U, et al. Feasibility Aspects of Exploring Exercise-Induced Neuroplasticity in Parkinson's Disease: A Pilot Randomized Controlled Trial. *Parkinsons Dis* 2020; **2020**: 2410863.

101. Joseph C, Brodin N, Leavy B, Hagströmer M, Löfgren N, Franzén E. Cost-effectiveness of the HiBalance training program for elderly with Parkinson's disease: analysis of data from a randomized controlled trial. *Clin Rehabil* 2019; **33**(2): 222-32.

102. Jung SH, Hasegawa N, Mancini M, et al. Effects of the agility boot camp with cognitive challenge (ABC-C) exercise program for Parkinson's disease. *NPJ Parkinsons Dis* 2020; **6**(1): 31.

103. Kadivar Z, Corcos DM, Foto J, Hondzinski JM. Effect of step training and rhythmic auditory stimulation on functional performance in Parkinson patients. *Neurorehabil Neural Repair* 2011; **25**(7): 626-35.

104. Keus SHJ, Bloem BR, van Hilten JJ, Ashburn A, Munneke M. Effectiveness of physiotherapy in Parkinson's disease: the feasibility of a randomised controlled trial. *Parkinsonism Relat Disord* 2007; **13**(2): 115-21.

105. Khalil H, Busse M, Quinn L, et al. A pilot study of a minimally supervised home exercise and walking program for people with Parkinson's disease in Jordan. *Neurodegener Dis Manag* 2017; **7**(1): 73-84.

106. Khuzema A, Brammatha A, Arul Selvan V. Effect of home-based Tai Chi, Yoga or conventional balance exercise on functional balance and mobility among persons with idiopathic Parkinson's disease: An experimental study. *Hong Kong Physiother J* 2020; **40**(1): 39-49.

107. Kim H, Kim E, Yun SJ, et al. Robot-assisted gait training with auditory and visual cues in Parkinson's disease: A randomized controlled trial. *Ann Phys Rehabil Med* 2022; **65**(3): 101620.

108. King LA, Salarian A, Mancini M, et al. Exploring outcome measures for exercise intervention in people with Parkinson's disease. *Parkinsons Dis* 2013; **2013**: 572134.

109. Kunkel D, Fitton C, Roberts L, et al. A randomized controlled feasibility trial exploring partnered ballroom dancing for people with Parkinson's disease. *Clin Rehabil* 2017; **31**(10): 1340-50.

110. Kurt EE, Büyükturan B, Büyükturan Ö, Erdem HR, Tuncay F. Effects of Ai Chi on balance, quality of life, functional mobility, and motor impairment in patients with Parkinson's disease<sup/>. *Disabil Rehabil* 2018; **40**(7): 791-7.

111. Kurtais Y, Kutlay S, Tur BS, Gok H, Akbostanci C. Does treadmill training improve lower-extremity tasks in Parkinson disease? A randomized controlled trial. *Clin J Sport Med* 2008; **18**(3): 289-91.

112. Kwok JYY, Kwan JCY, Auyeung M, et al. Effects of Mindfulness Yoga vs Stretching and Resistance Training Exercises on Anxiety and Depression for People With Parkinson Disease: A Randomized Clinical Trial. *JAMA Neurol* 2019; **76**(7): 755-63.

113. Landers MR, Hatlevig RM, Davis AD, Richards AR, Rosenlof LE. Does attentional focus during balance training in people with Parkinson's disease affect outcome? A randomised controlled clinical trial. *Clin Rehabil* 2016; **30**(1): 53-63.

114. Leal LC, Abrahin O, Rodrigues RP, et al. Low-volume resistance training improves the functional capacity of older individuals with Parkinson's disease. *Geriatr Gerontol Int* 2019; **19**(7): 635-40.

115. Lee H-J, Kim S-Y, Chae Y, et al. Turo (Qi Dance) Program for Parkinson's Disease Patients: Randomized, Assessor Blind, Waiting-List Control, Partial Crossover Study. *Explore (NY)* 2018; **14**(3): 216-23.

116. Lei ZG, Ma Y, Yu LN, Bao XB, Xiong R. Observation on the curative effect of whole body vibration training combined with extremity linkage training on the motor function of patients with Parkinson's disease. *Chinese Journal of Convalescent Medicine* 2021; **30**(01): 75-7.

117. Li F, Harmer P, Fitzgerald K, et al. Tai chi and postural stability in patients with Parkinson's disease. *N Engl J Med* 2012; **366**(6): 511-9.

118. Liao Y-Y, Yang Y-R, Cheng S-J, Wu Y-R, Fuh J-L, Wang R-Y. Virtual Reality-Based Training to Improve Obstacle-Crossing Performance and Dynamic Balance in Patients With Parkinson's Disease. *Neurorehabil Neural Repair* 2015; **29**(7): 658-67.

119. Liu J, Yan Z, Liao RS, Yu C, Shao M. The effect of virtual reality training on balance function in patients with Parkinson’s disease. *Chinese Journal of Rehabilitation Medicine* 2020; **35**(06): 682-7.

120. Löfgren N, Conradsson D, Rennie L, Moe-Nilssen R, Franzén E. The effects of integrated single- and dual-task training on automaticity and attention allocation in Parkinson's disease: A secondary analysis from a randomized trial. *Neuropsychology* 2019; **33**(2): 147-56.

121. Maidan I, Nieuwhof F, Bernad-Elazari H, et al. Evidence for Differential Effects of 2 Forms of Exercise on Prefrontal Plasticity During Walking in Parkinson's Disease. *Neurorehabil Neural Repair* 2018; **32**(3): 200-8.

122. Marumoto K, Yokoyama K, Inoue T, et al. Inpatient Enhanced Multidisciplinary Care Effects on the Quality of Life for Parkinson Disease: A Quasi-Randomized Controlled Trial. *J Geriatr Psychiatry Neurol* 2019; **32**(4): 186-94.

123. McNeely ME, Mai MM, Duncan RP, Earhart GM. Differential Effects of Tango Versus Dance for PD in Parkinson Disease. *Front Aging Neurosci* 2015; **7**: 239.

124. Medijainen K, Pääsuke M, Lukmann A, Taba P. Versatile guideline-based physiotherapy intervention in groups to improve gait speed in Parkinson's disease patients. *NeuroRehabilitation* 2019; **44**(4): 579-86.

125. Michels K, Dubaz O, Hornthal E, Bega D. "Dance Therapy" as a psychotherapeutic movement intervention in Parkinson's disease. *Complement Ther Med* 2018; **40**: 248-52.

126. Miyai I, Fujimoto Y, Yamamoto H, et al. Long-term effect of body weight-supported treadmill training in Parkinson's disease: a randomized controlled trial. *Arch Phys Med Rehabil* 2002; **83**(10): 1370-3.

127. Mollinedo-Cardalda I, Cancela-Carral JM, Vila-Suárez MH. Effect of a Mat Pilates Program with TheraBand on Dynamic Balance in Patients with Parkinson's Disease: Feasibility Study and Randomized Controlled Trial. *Rejuvenation Res* 2018; **21**(5): 423-30.

128. Monticone M, Ambrosini E, Laurini A, Rocca B, Foti C. In-patient multidisciplinary rehabilitation for Parkinson's disease: A randomized controlled trial. *Mov Disord* 2015; **30**(8): 1050-8.

129. Moon S, Sarmento CVM, Steinbacher M, et al. Can Qigong improve non-motor symptoms in people with Parkinson's disease - A pilot randomized controlled trial? *Complement Ther Clin Pract* 2020; **39**: 101169.

130. Moon S, Schmidt M, Smirnova IV, Colgrove Y, Liu W. Qigong Exercise May Reduce Serum TNF-α Levels and Improve Sleep in People with Parkinson's Disease: A Pilot Study. *Medicines (Basel)* 2017; **4**(2).

131. Morris ME, Iansek R, Kirkwood B. A randomized controlled trial of movement strategies compared with exercise for people with Parkinson's disease. *Mov Disord* 2009; **24**(1): 64-71.

132. Morris ME, Menz HB, McGinley JL, et al. A Randomized Controlled Trial to Reduce Falls in People With Parkinson's Disease. *Neurorehabil Neural Repair* 2015; **29**(8): 777-85.

133. Morris ME, Taylor NF, Watts JJ, et al. A home program of strength training, movement strategy training and education did not prevent falls in people with Parkinson's disease: a randomised trial. *J Physiother* 2017; **63**(2).

134. Morrone M, Miccinilli S, Bravi M, et al. Perceptive rehabilitation and trunk posture alignment in patients with Parkinson disease: a single blind randomized controlled trial. *Eur J Phys Rehabil Med* 2016; **52**(6): 799-809.

135. Myers PS, Harrison EC, Rawson KS, et al. Yoga Improves Balance and Low-Back Pain, but Not Anxiety, in People with Parkinson's Disease. *Int J Yoga Therap* 2020; **30**(1): 41-8.

136. Nadeau A, Pourcher E, Corbeil P. Effects of 24 wk of treadmill training on gait performance in Parkinson's disease. *Med Sci Sports Exerc* 2014; **46**(4): 645-55.

137. Ni M, Mooney K, Signorile JF. Controlled pilot study of the effects of power yoga in Parkinson's disease. *Complement Ther Med* 2016; **25**: 126-31.

138. Ni M, Signorile JF, Balachandran A, Potiaumpai M. Power training induced change in bradykinesia and muscle power in Parkinson's disease. *Parkinsonism Relat Disord* 2016; **23**: 37-44.

139. Ni M, Signorile JF, Mooney K, et al. Comparative Effect of Power Training and High-Speed Yoga on Motor Function in Older Patients With Parkinson Disease. *Arch Phys Med Rehabil* 2016; **97**(3).

140. Nieuwboer A, Kwakkel G, Rochester L, et al. Cueing training in the home improves gait-related mobility in Parkinson's disease: the RESCUE trial. *J Neurol Neurosurg Psychiatry* 2007; **78**(2): 134-40.

141. Nocera JR, Amano S, Vallabhajosula S, Hass CJ. Tai Chi Exercise to Improve Non-Motor Symptoms of Parkinson's Disease. *J Yoga Phys Ther* 2013; **3**.

142. Oliveira GSD, Iraci L, Pinheiro GS, et al. Effect of exercise and grape juice on epigenetic modulation and functional outcomes in PD: A randomized clinical trial. *Physiol Behav* 2020; **227**: 113135.

143. Ortiz-Rubio A, Cabrera-Martos I, Torres-Sánchez I, Casilda-López J, López-López L, Valenza MC. Effects of a resistance training program on balance and fatigue perception in patients with Parkinson's disease: A randomized controlled trial. *Med Clin (Barc)* 2018; **150**(12): 460-4.

144. Palamara G, Gotti F, Maestri R, et al. Land Plus Aquatic Therapy Versus Land-Based Rehabilitation Alone for the Treatment of Balance Dysfunction in Parkinson Disease: A Randomized Controlled Study With 6-Month Follow-Up. *Arch Phys Med Rehabil* 2017; **98**(6): 1077-85.

145. Paolucci T, Zangrando F, Piccinini G, et al. Impact of Mézières Rehabilitative Method in Patients with Parkinson's Disease: A Randomized Controlled Trial. *Parkinsons Dis* 2017; **2017**: 2762987.

146. Park A, Zid D, Russell J, et al. Effects of a formal exercise program on Parkinson's disease: a pilot study using a delayed start design. *Parkinsonism Relat Disord* 2014; **20**(1): 106-11.

147. Paul SS, Canning CG, Song J, Fung VSC, Sherrington C. Leg muscle power is enhanced by training in people with Parkinson's disease: a randomized controlled trial. *Clin Rehabil* 2014; **28**(3): 275-88.

148. Pazzaglia C, Imbimbo I, Tranchita E, et al. Comparison of virtual reality rehabilitation and conventional rehabilitation in Parkinson's disease: a randomised controlled trial. *Physiotherapy* 2020; **106**: 36-42.

149. Pelosin E, Cerulli C, Ogliastro C, et al. A Multimodal Training Modulates Short Afferent Inhibition and Improves Complex Walking in a Cohort of Faller Older Adults With an Increased Prevalence of Parkinson's Disease. *J Gerontol A Biol Sci Med Sci* 2020; **75**(4): 722-8.

150. Pérez de la Cruz S. Effectiveness of aquatic therapy for the control of pain and increased functionality in people with Parkinson's disease: a randomized clinical trial. *Eur J Phys Rehabil Med* 2017; **53**(6): 825-32.

151. Pérez-de la Cruz S. A bicentric controlled study on the effects of aquatic Ai Chi in Parkinson disease. *Complement Ther Med* 2018; **36**: 147-53.

152. Pérez-de la Cruz S. Mental health in Parkinson's disease after receiving aquatic therapy: a clinical trial. *Acta Neurol Belg* 2019; **119**(2): 193-200.

153. Picelli A, Melotti C, Origano F, Waldner A, Gimigliano R, Smania N. Does robotic gait training improve balance in Parkinson's disease? A randomized controlled trial. *Parkinsonism Relat Disord* 2012; **18**(8): 990-3.

154. Picelli A, Melotti C, Origano F, Neri R, Waldner A, Smania N. Robot-assisted gait training versus equal intensity treadmill training in patients with mild to moderate Parkinson's disease: a randomized controlled trial. *Parkinsonism Relat Disord* 2013; **19**(6): 605-10.

155. Picelli A, Melotti C, Origano F, et al. Robot-assisted gait training is not superior to balance training for improving postural instability in patients with mild to moderate Parkinson's disease: a single-blind randomized controlled trial. *Clin Rehabil* 2015; **29**(4): 339-47.

156. Picelli A, Varalta V, Melotti C, et al. Effects of treadmill training on cognitive and motor features of patients with mild to moderate Parkinson's disease: a pilot, single-blind, randomized controlled trial. *Funct Neurol* 2016; **31**(1): 25-31.

157. Pohl P, Dizdar N, Hallert E. The Ronnie Gardiner Rhythm and Music Method - a feasibility study in Parkinson's disease. *Disabil Rehabil* 2013; **35**(26): 2197-204.

158. Pohl P, Wressle E, Lundin F, Enthoven P, Dizdar N. Group-based music intervention in Parkinson's disease - findings from a mixed-methods study. *Clin Rehabil* 2020; **34**(4): 533-44.

159. Poier D, Rodrigues Recchia D, Ostermann T, Büssing A. A Randomized Controlled Trial to Investigate the Impact of Tango Argentino versus Tai Chi on Quality of Life in Patients with Parkinson Disease: A Short Report. *Complement Med Res* 2019; **26**(6): 398-403.

160. Poliakoff E, Galpin AJ, McDonald K, et al. The effect of gym training on multiple outcomes in Parkinson's disease: a pilot randomised waiting-list controlled trial. *NeuroRehabilitation* 2013; **32**(1): 125-34.

161. Pompeu JE, Mendes FADS, Silva KGd, et al. Effect of Nintendo Wii™-based motor and cognitive training on activities of daily living in patients with Parkinson's disease: a randomised clinical trial. *Physiotherapy* 2012; **98**(3): 196-204.

162. Prodoehl J, Rafferty MR, David FJ, et al. Two-year exercise program improves physical function in Parkinson's disease: the PRET-PD randomized clinical trial. *Neurorehabil Neural Repair* 2015; **29**(2): 112-22.

163. Protas EJ, Mitchell K, Williams A, Qureshy H, Caroline K, Lai EC. Gait and step training to reduce falls in Parkinson's disease. *NeuroRehabilitation* 2005; **20**(3): 183-90.

164. Qutubuddin AA, Cifu DX, Armistead-Jehle P, Carne W, McGuirk TE, Baron MS. A comparison of computerized dynamic posturography therapy to standard balance physical therapy in individuals with Parkinson's disease: a pilot study. *NeuroRehabilitation* 2007; **22**(4): 261-5.

165. Rafferty MR, Prodoehl J, Robichaud JA, et al. Effects of 2 Years of Exercise on Gait Impairment in People With Parkinson Disease: The PRET-PD Randomized Trial. *J Neurol Phys Ther* 2017; **41**(1): 21-30.

166. Rennie L, Opheim A, Dietrichs E, Löfgren N, Franzén E. Highly challenging balance and gait training for individuals with Parkinson's disease improves pace, rhythm and variability domains of gait - A secondary analysis from a randomized controlled trial. *Clin Rehabil* 2021; **35**(2): 200-12.

167. Ribas CG, Alves da Silva L, Corrêa MR, Teive HG, Valderramas S. Effectiveness of exergaming in improving functional balance, fatigue and quality of life in Parkinson's disease: A pilot randomized controlled trial. *Parkinsonism Relat Disord* 2017; **38**: 13-8.

168. Ridgel AL, Ault DL. High-Cadence Cycling Promotes Sustained Improvement in Bradykinesia, Rigidity, and Mobility in Individuals with Mild-Moderate Parkinson's Disease. *Parkinsons Dis* 2019; **2019**: 4076862.

169. Rios Romenets S, Anang J, Fereshtehnejad S-M, Pelletier A, Postuma R. Tango for treatment of motor and non-motor manifestations in Parkinson's disease: a randomized control study. *Complement Ther Med* 2015; **23**(2): 175-84.

170. Rocha P, Aguiar L, McClelland JA, Morris ME. Dance therapy for Parkinson's disease: a randomised feasibility trial. *International Journal of Therapy and Rehabilitation* 2018; **25**(2): 64-72.

171. Sacheli MA, Neva JL, Lakhani B, et al. Exercise increases caudate dopamine release and ventral striatal activation in Parkinson's disease. *Mov Disord* 2019; **34**(12): 1891-900.

172. Sage MD, Almeida QJ. Symptom and gait changes after sensory attention focused exercise vs aerobic training in Parkinson's disease. *Mov Disord* 2009; **24**(8): 1132-8.

173. Sale P, De Pandis MF, Le Pera D, et al. Robot-assisted walking training for individuals with Parkinson's disease: a pilot randomized controlled trial. *BMC Neurol* 2013; **13**: 50.

174. San Martín Valenzuela C, Moscardó LD, López-Pascual J, Serra-Añó P, Tomás JM. Effects of Dual-Task Group Training on Gait, Cognitive Executive Function, and Quality of Life in People With Parkinson Disease: Results of Randomized Controlled DUALGAIT Trial. *Arch Phys Med Rehabil* 2020; **101**(11).

175. Sangarapillai K, Norman BM, Almeida QJ. Boxing vs Sensory Exercise for Parkinson's Disease: A Double-Blinded Randomized Controlled Trial. *Neurorehabil Neural Repair* 2021; **35**(9): 769-77.

176. Santos SM, da Silva RA, Terra MB, Almeida IA, de Melo LB, Ferraz HB. Balance versus resistance training on postural control in patients with Parkinson's disease: a randomized controlled trial. *Eur J Phys Rehabil Med* 2017; **53**(2): 173-83.

177. Santos L, Fernandez-Rio J, Winge K, et al. Effects of supervised slackline training on postural instability, freezing of gait, and falls efficacy in people with Parkinson's disease. *Disabil Rehabil* 2017; **39**(16): 1573-80.

178. Santos L, Fernandez-Rio J, Winge K, et al. Effects of progressive resistance exercise in akinetic-rigid Parkinson's disease patients: a randomized controlled trial. *Eur J Phys Rehabil Med* 2017; **53**(5): 651-63.

179. Santos P, Machado T, Santos L, Ribeiro N, Melo A. Efficacy of the Nintendo Wii combination with Conventional Exercises in the rehabilitation of individuals with Parkinson's disease: A randomized clinical trial. *NeuroRehabilitation* 2019; **45**(2): 255-63.

180. Schabrun SM, Lamont RM, Brauer SG. Transcranial Direct Current Stimulation to Enhance Dual-Task Gait Training in Parkinson's Disease: A Pilot RCT. *PLoS One* 2016; **11**(6): e0158497.

181. Schenkman M, Cutson TM, Kuchibhatla M, et al. Exercise to improve spinal flexibility and function for people with Parkinson's disease: a randomized, controlled trial. *J Am Geriatr Soc* 1998; **46**(10): 1207-16.

182. Schenkman M, Hall DA, Barón AE, Schwartz RS, Mettler P, Kohrt WM. Exercise for people in early- or mid-stage Parkinson disease: a 16-month randomized controlled trial. *Phys Ther* 2012; **92**(11): 1395-410.

183. Schenkman M, Moore CG, Kohrt WM, et al. Effect of High-Intensity Treadmill Exercise on Motor Symptoms in Patients With De Novo Parkinson Disease: A Phase 2 Randomized Clinical Trial. *JAMA Neurol* 2018; **75**(2): 219-26.

184. Schilling BK, Pfeiffer RF, Ledoux MS, Karlage RE, Bloomer RJ, Falvo MJ. Effects of moderate-volume, high-load lower-body resistance training on strength and function in persons with Parkinson's disease: a pilot study. *Parkinsons Dis* 2010; **2010**: 824734.

185. Schlenstedt C, Paschen S, Seuthe J, et al. Moderate Frequency Resistance and Balance Training Do Not Improve Freezing of Gait in Parkinson's Disease: A Pilot Study. *Front Neurol* 2018; **9**: 1084.

186. Schlenstedt C, Paschen S, Kruse A, Raethjen J, Weisser B, Deuschl G. Resistance versus Balance Training to Improve Postural Control in Parkinson's Disease: A Randomized Rater Blinded Controlled Study. *PLoS One* 2015; **10**(10): e0140584.

187. Schlick C, Ernst A, Bötzel K, Plate A, Pelykh O, Ilmberger J. Visual cues combined with treadmill training to improve gait performance in Parkinson's disease: a pilot randomized controlled trial. *Clin Rehabil* 2016; **30**(5): 463-71.

188. Sedaghati P, Goudarzian M, Daneshmandi H, Ardjmand A. Effects of Alexander-based corrective techniques on forward flexed posture, risk of fall, and fear of falling in idiopathic Parkinson’s disease. *Archives of Neuroscience* 2018; **5**(2).

189. Serrao M, Pierelli F, Sinibaldi E, et al. Progressive Modular Rebalancing System and Visual Cueing for Gait Rehabilitation in Parkinson's Disease: A Pilot, Randomized, Controlled Trial With Crossover. *Front Neurol* 2019; **10**: 902.

190. Shahmohammadi R, Sharifi G-R, Melvin JM, Sadeghi-Demneh E. A comparison between aquatic and land-based physical exercise on postural sway and quality of life in people with Parkinson’s disease: a randomized controlled pilot study. *Sport Sciences for Health* 2017; **13**(2): 341-8.

191. Shen X, Mak MKY. Technology-assisted balance and gait training reduces falls in patients with Parkinson's disease: a randomized controlled trial with 12-month follow-up. *Neurorehabil Neural Repair* 2015; **29**(2): 103-11.

192. Shen X, Mak MKY. Repetitive step training with preparatory signals improves stability limits in patients with Parkinson's disease. *J Rehabil Med* 2012; **44**(11): 944-9.

193. Shih M-C, Wang R-Y, Cheng S-J, Yang Y-R. Effects of a balance-based exergaming intervention using the Kinect sensor on posture stability in individuals with Parkinson's disease: a single-blinded randomized controlled trial. *J Neuroeng Rehabil* 2016; **13**(1): 78.

194. Shulman LM, Katzel LI, Ivey FM, et al. Randomized clinical trial of 3 types of physical exercise for patients with Parkinson disease. *JAMA Neurol* 2013; **70**(2): 183-90.

195. Silva AZd, Israel VL. Effects of dual-task aquatic exercises on functional mobility, balance and gait of individuals with Parkinson's disease: A randomized clinical trial with a 3-month follow-up. *Complement Ther Med* 2019; **42**: 119-24.

196. Silva-Batista C, Corcos DM, Barroso R, et al. Instability Resistance Training Improves Neuromuscular Outcome in Parkinson's Disease. *Med Sci Sports Exerc* 2017; **49**(4): 652-60.

197. Silva-Batista C, Corcos DM, Kanegusuku H, et al. Balance and fear of falling in subjects with Parkinson's disease is improved after exercises with motor complexity. *Gait Posture* 2018; **61**: 90-7.

198. Silva-Batista C, Corcos DM, Roschel H, et al. Resistance Training with Instability for Patients with Parkinson's Disease. *Med Sci Sports Exerc* 2016; **48**(9): 1678-87.

199. Silva-Batista C, de Brito LC, Corcos DM, et al. Resistance Training Improves Sleep Quality in Subjects With Moderate Parkinson's Disease. *J Strength Cond Res* 2017; **31**(8): 2270-7.

200. Silva-Batista C, de Lima-Pardini AC, Nucci MP, et al. A Randomized, Controlled Trial of Exercise for Parkinsonian Individuals With Freezing of Gait. *Mov Disord* 2020; **35**(9): 1607-17.

201. Smania N, Corato E, Tinazzi M, et al. Effect of balance training on postural instability in patients with idiopathic Parkinson's disease. *Neurorehabil Neural Repair* 2010; **24**(9): 826-34.

202. Solla P, Cugusi L, Bertoli M, et al. Sardinian Folk Dance for Individuals with Parkinson's Disease: A Randomized Controlled Pilot Trial. *J Altern Complement Med* 2019; **25**(3): 305-16.

203. Song J, Paul SS, Caetano MJD, et al. Home-based step training using videogame technology in people with Parkinson's disease: a single-blinded randomised controlled trial. *Clin Rehabil* 2018; **32**(3): 299-311.

204. Steib S, Klamroth S, Gaßner H, et al. Perturbation During Treadmill Training Improves Dynamic Balance and Gait in Parkinson's Disease: A Single-Blind Randomized Controlled Pilot Trial. *Neurorehabil Neural Repair* 2017; **31**(8): 758-68.

205. Steib S, Klamroth S, Gaßner H, et al. Exploring gait adaptations to perturbed and conventional treadmill training in Parkinson's disease: Time-course, sustainability, and transfer. *Hum Mov Sci* 2019; **64**: 123-32.

206. Stożek J, Rudzińska M, Pustułka-Piwnik U, Szczudlik A. The effect of the rehabilitation program on balance, gait, physical performance and trunk rotation in Parkinson's disease. *Aging Clin Exp Res* 2016; **28**(6): 1169-77.

207. Strand KL, Cherup NP, Totillo MC, Castillo DC, Gabor NJ, Signorile JF. Periodized Resistance Training With and Without Functional Training Improves Functional Capacity, Balance, and Strength in Parkinson's Disease. *J Strength Cond Res* 2021; **35**(6): 1611-9.

208. Strouwen C, Molenaar EALM, Münks L, et al. Training dual tasks together or apart in Parkinson's disease: Results from the DUALITY trial. *Mov Disord* 2017; **32**(8): 1201-10.

209. Stuckenschneider T, Helmich I, Raabe-Oetker A, Froböse I, Feodoroff B. Active assistive forced exercise provides long-term improvement to gait velocity and stride length in patients bilaterally affected by Parkinson's disease. *Gait Posture* 2015; **42**(4): 485-90.

210. Tang LJ, Peng YY, Wang CX, Chen M, Yue LC. The effects of Parkinson's exercise and slow walking on the motor function and balance function of the limbs in Parkinson's disease. *Chinese Journal of Gerontology* 2020; **40**(13): 2801-3.

211. Teixeira-Machado L, Araújo FM, Cunha FA, Menezes M, Menezes T, Melo DeSantana J. Feldenkrais method-based exercise improves quality of life in individuals with Parkinson's disease: a controlled, randomized clinical trial. *Altern Ther Health Med* 2015; **21**(1).

212. Terrens AF, Soh S-E, Morgan P. The safety and feasibility of a Halliwick style of aquatic physiotherapy for falls and balance dysfunction in people with Parkinson's Disease: A single blind pilot trial. *PLoS One* 2020; **15**(7): e0236391.

213. Thaut MH, McIntosh GC, Rice RR, Miller RA, Rathbun J, Brault JM. Rhythmic auditory stimulation in gait training for Parkinson's disease patients. *Mov Disord* 1996; **11**(2): 193-200.

214. Tollár J, Nagy F, Hortobágyi T. Vastly Different Exercise Programs Similarly Improve Parkinsonian Symptoms: A Randomized Clinical Trial. *Gerontology* 2019; **65**(2): 120-7.

215. Tollár J, Nagy F, Kovács N, Hortobágyi T. A High-Intensity Multicomponent Agility Intervention Improves Parkinson Patients' Clinical and Motor Symptoms. *Arch Phys Med Rehabil* 2018; **99**(12).

216. Tollár J, Nagy F, Kovács N, Hortobágyi T. Two-Year Agility Maintenance Training Slows the Progression of Parkinsonian Symptoms. *Med Sci Sports Exerc* 2019; **51**(2): 237-45.

217. Tramontano M, Bonnì S, Martino Cinnera A, et al. Blindfolded Balance Training in Patients with Parkinson's Disease: A Sensory-Motor Strategy to Improve the Gait. *Parkinsons Dis* 2016; **2016**: 7536862.

218. van den Heuvel MRC, Kwakkel G, Beek PJ, Berendse HW, Daffertshofer A, van Wegen EEH. Effects of augmented visual feedback during balance training in Parkinson's disease: a pilot randomized clinical trial. *Parkinsonism Relat Disord* 2014; **20**(12): 1352-8.

219. van der Kolk NM, de Vries NM, Kessels RPC, et al. Effectiveness of home-based and remotely supervised aerobic exercise in Parkinson's disease: a double-blind, randomised controlled trial. *Lancet Neurol* 2019; **18**(11).

220. van der Kolk NM, de Vries NM, Penko AL, et al. A remotely supervised home-based aerobic exercise programme is feasible for patients with Parkinson's disease: results of a small randomised feasibility trial. *J Neurol Neurosurg Psychiatry* 2018; **89**(9): 1003-5.

221. van Nimwegen M, Speelman AD, Overeem S, et al. Promotion of physical activity and fitness in sedentary patients with Parkinson's disease: randomised controlled trial. *BMJ* 2013; **346**: f576.

222. Van Puymbroeck M, Walter AA, Hawkins BL, et al. Functional Improvements in Parkinson's Disease Following a Randomized Trial of Yoga. *Evid Based Complement Alternat Med* 2018; **2018**: 8516351.

223. Vanbellingen T, Nyffeler T, Nigg J, et al. Home based training for dexterity in Parkinson's disease: A randomized controlled trial. *Parkinsonism Relat Disord* 2017; **41**: 92-8.

224. Vergara-Diaz G, Osypiuk K, Hausdorff JM, et al. Tai Chi for Reducing Dual-task Gait Variability, a Potential Mediator of Fall Risk in Parkinson's Disease: A Pilot Randomized Controlled Trial. *Glob Adv Health Med* 2018; **7**: 2164956118775385.

225. Vieira de Moraes Filho A, Chaves SN, Martins WR, et al. Progressive Resistance Training Improves Bradykinesia, Motor Symptoms and Functional Performance in Patients with Parkinson's Disease. *Clin Interv Aging* 2020; **15**: 87-95.

226. Vieira-Yano B, Martini DN, Horak FB, et al. The Adapted Resistance Training with Instability Randomized Controlled Trial for Gait Automaticity. *Mov Disord* 2021; **36**(1): 152-63.

227. Vitório R, Teixeira-Arroyo C, Lirani-Silva E, et al. Effects of 6-month, Multimodal Exercise Program on Clinical and Gait Parameters of Patients with Idiopathic Parkinson's Disease: A Pilot Study. *ISRN Neurol* 2011; **2011**: 714947.

228. Vivas J, Arias P, Cudeiro J. Aquatic therapy versus conventional land-based therapy for Parkinson's disease: an open-label pilot study. *Arch Phys Med Rehabil* 2011; **92**(8): 1202-10.

229. Volpe D, Giantin MG, Fasano A. A wearable proprioceptive stabilizer (Equistasi®) for rehabilitation of postural instability in Parkinson's disease: a phase II randomized double-blind, double-dummy, controlled study. *PLoS One* 2014; **9**(11): e112065.

230. Volpe D, Giantin MG, Maestri R, Frazzitta G. Comparing the effects of hydrotherapy and land-based therapy on balance in patients with Parkinson's disease: a randomized controlled pilot study. *Clin Rehabil* 2014; **28**(12): 1210-7.

231. Volpe D, Giantin MG, Manuela P, et al. Water-based vs. non-water-based physiotherapy for rehabilitation of postural deformities in Parkinson's disease: a randomized controlled pilot study. *Clin Rehabil* 2017; **31**(8): 1107-15.

232. Volpe D, Signorini M, Marchetto A, Lynch T, Morris ME. A comparison of Irish set dancing and exercises for people with Parkinson's disease: a phase II feasibility study. *BMC Geriatr* 2013; **13**: 54.

233. Wallén MB, Hagströmer M, Conradsson D, Sorjonen K, Franzén E. Long-term effects of highly challenging balance training in Parkinson's disease-a randomized controlled trial. *Clin Rehabil* 2018; **32**(11): 1520-9.

234. Wang WH, Bi HY, Yue ZG, et al. Effect of Visual Tracking Training combined with Six-character Formula on Motor

Function and Quality of Life in Patients with Parkinson's Disease. *Rehabilitation Medicine* 2020; **30**(06): 474-8.

235. White DK, Wagenaar RC, Ellis TD, Tickle-Degnen L. Changes in walking activity and endurance following rehabilitation for people with Parkinson disease. *Arch Phys Med Rehabil* 2009; **90**(1): 43-50.

236. Wong-Yu ISK, Mak MKY. Multi-dimensional balance training programme improves balance and gait performance in people with Parkinson's disease: A pragmatic randomized controlled trial with 12-month follow-up. *Parkinsonism Relat Disord* 2015; **21**(6): 615-21.

237. Wong-Yu IS, Mak MK. Task- and Context-Specific Balance Training Program Enhances Dynamic Balance and Functional Performance in Parkinsonian Nonfallers: A Randomized Controlled Trial With Six-Month Follow-Up. *Arch Phys Med Rehabil* 2015; **96**(12): 2103-11.

238. Xiao C-M, Zhuang Y-C. Effect of health Baduanjin Qigong for mild to moderate Parkinson's disease. *Geriatr Gerontol Int* 2016; **16**(8): 911-9.

239. Xiao C, Zhuang Y, Kang Y. Effect of Health Qigong Baduanjin on Fall Prevention in Individuals with Parkinson's Disease. *J Am Geriatr Soc* 2016; **64**(11): e227-e8.

240. Yang Y-R, Cheng S-J, Lee Y-J, Liu Y-C, Wang R-Y. Cognitive and motor dual task gait training exerted specific training effects on dual task gait performance in individuals with Parkinson's disease: A randomized controlled pilot study. *PLoS One* 2019; **14**(6): e0218180.

241. Yang Y-R, Lee Y-Y, Cheng S-J, Wang R-Y. Downhill walking training in individuals with Parkinson's disease: a randomized controlled trial. *Am J Phys Med Rehabil* 2010; **89**(9): 706-14.

242. Yang W-C, Wang H-K, Wu R-M, Lo C-S, Lin K-H. Home-based virtual reality balance training and conventional balance training in Parkinson's disease: A randomized controlled trial. *J Formos Med Assoc* 2016; **115**(9): 734-43.

243. Yi-zhao W, Hua Z, Shi-chun F, Wei-jia H, ZHANG Y. Effect of water-based exercise on motor function, balance function and walking ability in patients with Parkinson's disease. *Chinese Journal of Contemporary Neurology & Neurosurgery* 2017; **17**(5): 346.

244. Yotnuengnit P, Bhidayasiri R, Donkhan R, Chaluaysrimuang J, Piravej K. Effects of Transcranial Direct Current Stimulation Plus Physical Therapy on Gait in Patients With Parkinson Disease: A Randomized Controlled Trial. *Am J Phys Med Rehabil* 2018; **97**(1).

245. You H, She JH. Observation on the effect of Tai Chi balance exercise group therapy on improving the balance function and depression state of patients with Parkinson's disease. *Guizhou Medical Journal* 2020; **44**(07): 1071-2.

246. Youm C, Kim Y, Noh B, Lee M, Kim J, Cheon S-M. Impact of Trunk Resistance and Stretching Exercise on Fall-Related Factors in Patients with Parkinson's Disease: A Randomized Controlled Pilot Study. *Sensors (Basel)* 2020; **20**(15).

247. Zeng DC, Tian L, Tan TC, et al. The effects of whole body vibration combined with multiple exercise strategy training on the motor function and daily living ability of patients with Parkinson's disease. *Chinese Journal of Rehabilitation Medicine* 2020; **35**(12): 1486-8.

248. Zhang T-Y, Hu Y, Nie Z-Y, et al. Effects of Tai Chi and Multimodal Exercise Training on Movement and Balance Function in Mild to Moderate Idiopathic Parkinson Disease. *Am J Phys Med Rehabil* 2015; **94**(10 Suppl 1): 921-9.

249. Zhang X, Zhou X, Zhou X. Effect Analysis of Baduanjin Combined with Balance Pad Training in Improving Elderly Patients Balance Function with Parkinson Disease. *Chinese and Foreign Medical Research* 2020; **18**(35): 92-4.

250. Zheng L, Zheng L, Chen J, Zheng F. Effect of Resistance Exercise on Sleep Quality and Emotional State of Elderly Patients with Parkinson Disease Sleep Disorder. *Chinese and Foreign Medical Research* 2020; **18**(34): 175-7.

# Appendix 9: Risk of Bias

## 9.1 The risk of bias assessment for the individual included studies

| **Author** | **1** | **2** | **3** | **4** | **5** | **6** | **7** | **8** | **9** | **10** | **11** | **12** | **Total** |
| --- | --- | --- | --- | --- | --- | --- | --- | --- | --- | --- | --- | --- | --- |
| Abraham, Hart ^1^ | 1 | 1 | 0 | 1 | 1 | 2 | 0 | 2 | 1 | 1 | 0 | 1 | 11 |
| Ajimsha, Majeed ^2^ | 1 | 0 | 0 | 1 | 1 | 3 | 0 | 2 | 1 | 1 | 0 | 1 | 11 |
| Allen, Canning ^3^ | 1 | 1 | 0 | 1 | 1 | 3 | 1 | 2 | 1 | 0 | 1 | 1 | 13 |
| Allen, Song ^4^ | 1 | 1 | 0 | 1 | 1 | 3 | 0 | 2 | 1 | 1 | 0 | 1 | 12 |
| Altmann, Stegemöller ^5^ | 1 | 0 | 0 | 1 | 1 | 1 | 0 | 2 | 1 | 1 | 1 | 1 | 10 |
| Amano, Nocera ^6^ | 1 | 1 | 0 | 1 | 1 | 0 | 0 | 2 | 1 | 1 | 0 | 1 | 9 |
| Amara, Wood ^7^ | 1 | 1 | 0 | 1 | 0 | 2 | 1 | 2 | 1 | 1 | 0 | 1 | 11 |
| Arcolin, Pisano ^8^ | 1 | 1 | 0 | 1 | 1 | 1 | 0 | 2 | 1 | 1 | 1 | 1 | 11 |
| Arfa-Fatollahkhani, Safar Cherati ^9^ | 1 | 1 | 0 | 1 | 1 | 1 | 0 | 2 | 1 | 1 | 1 | 1 | 11 |
| Ashburn, Fazakarley ^10^ | 1 | 1 | 0 | 1 | 1 | 2 | 1 | 2 | 1 | 1 | 1 | 1 | 13 |
| Atan, Özyemişci Taşkıran ^11^ | 1 | 1 | 0 | 1 | 1 | 3 | 0 | 2 | 1 | 1 | 1 | 1 | 13 |
| Bakhshayesh, Sayyar ^12^ | 1 | 0 | 0 | 1 | 0 | 1 | 0 | 2 | 1 | 0 | 1 | 1 | 8 |
| Bang and Shin ^13^ | 1 | 0 | 1 | 1 | 1 | 2 | 0 | 2 | 1 | 1 | 1 | 1 | 12 |
| Barboza, Terra ^14^ | 1 | 1 | 1 | 1 | 1 | 3 | 1 | 2 | 1 | 1 | 0 | 1 | 14 |
| Beck, Intzandt ^15^ | 1 | 1 | 0 | 0 | 1 | 2 | 0 | 2 | 1 | 1 | 0 | 1 | 10 |
| Beck, Wang ^16^ | 1 | 0 | 0 | 1 | 1 | 2 | 1 | 2 | 1 | 0 | 0 | 1 | 10 |
| Bekkers, Mirelman ^17^ | 1 | 0 | 0 | 1 | 1 | 1 | 0 | 2 | 1 | 1 | 1 | 1 | 10 |
| Bello, Sanchez ^18^ | 1 | 0 | 0 | 1 | 0 | 0 | 0 | 2 | 1 | 1 | 1 | 1 | 8 |
| Burini, Farabollini ^19^ | 1 | 1 | 1 | 1 | 1 | 1 | 0 | 2 | 1 | 1 | 1 | 1 | 12 |
| Cabrera-Martos, Jiménez-Martín ^20^ | 1 | 1 | 1 | 1 | 1 | 2 | 1 | 2 | 1 | 1 | 1 | 1 | 14 |
| Cakit, Saracoglu ^21^ | 1 | 0 | 0 | 1 | 1 | 1 | 0 | 2 | 1 | 1 | 1 | 1 | 10 |
| Calabrò, Naro ^22^ | 1 | 1 | 1 | 1 | 1 | 3 | 1 | 2 | 1 | 1 | 1 | 1 | 15 |
| Cancela, Mollinedo ^23^ | 1 | 1 | 0 | 1 | 1 | 2 | 0 | 2 | 1 | 1 | 1 | 1 | 12 |
| Canning, Allen ^24^ | 1 | 0 | 0 | 1 | 1 | 3 | 1 | 2 | 1 | 1 | 1 | 1 | 13 |
| Capato, de Vries ^25^ | 1 | 1 | 1 | 1 | 1 | 3 | 1 | 2 | 1 | 1 | 0 | 1 | 14 |
| Capato, Nonnekes ^26^ | 1 | 1 | 1 | 1 | 1 | 3 | 1 | 2 | 1 | 1 | 0 | 1 | 14 |
| Capecci, Pournajaf ^27^ | 1 | 0 | 0 | 1 | 1 | 2 | 0 | 2 | 1 | 1 | 1 | 1 | 11 |
| Capecci, Serpicelli ^28^ | 1 | 1 | 0 | 1 | 1 | 3 | 0 | 2 | 1 | 0 | 0 | 1 | 11 |
| Carda, Invernizzi ^29^ | 1 | 0 | 0 | 1 | 1 | 3 | 1 | 2 | 1 | 1 | 1 | 1 | 13 |
| Carpinella, Cattaneo ^30^ | 1 | 1 | 0 | 0 | 1 | 2 | 0 | 2 | 1 | 1 | 0 | 1 | 10 |
| Carroll, Volpe ^31^ | 1 | 0 | 1 | 1 | 0 | 3 | 1 | 2 | 1 | 1 | 1 | 1 | 13 |
| Carvalho, Barbirato ^32^ | 1 | 0 | 0 | 0 | 1 | 1 | 0 | 2 | 1 | 1 | 1 | 1 | 9 |
| Cheng, Yang ^33^ | 1 | 0 | 1 | 1 | 0 | 2 | 0 | 2 | 1 | 1 | 1 | 1 | 11 |
| Cheng, Yang ^34^ | 1 | 0 | 1 | 1 | 0 | 3 | 0 | 2 | 1 | 1 | 1 | 1 | 12 |
| Cherup, Strand ^35^ | 1 | 1 | 0 | 1 | 0 | 2 | 0 | 2 | 1 | 1 | 0 | 1 | 10 |
| Cheung, Bhimani ^36^ | 1 | 1 | 1 | 1 | 0 | 3 | 0 | 2 | 1 | 0 | 0 | 1 | 11 |
| Chivers Seymour, Pickering ^37^ | 1 | 1 | 0 | 1 | 1 | 2 | 1 | 2 | 1 | 0 | 0 | 1 | 11 |
| Choi ^38^ | 1 | 1 | 0 | 1 | 0 | 0 | 0 | 2 | 1 | 0 | 0 | 1 | 7 |
| Clarke, Patel ^39^ | 1 | 1 | 0 | 1 | 0 | 3 | 1 | 2 | 1 | 0 | 0 | 1 | 11 |
| Clerici, Maestri ^40^ | 1 | 1 | 0 | 1 | 1 | 2 | 1 | 2 | 1 | 1 | 1 | 1 | 13 |
| Combs, Diehl ^41^ | 1 | 1 | 0 | 1 | 1 | 2 | 1 | 2 | 1 | 1 | 1 | 1 | 13 |
| Conradsson, Löfgren ^42^ | 1 | 1 | 1 | 1 | 0 | 3 | 1 | 2 | 1 | 0 | 1 | 1 | 13 |
| Corcos, Robichaud ^43^ | 1 | 0 | 0 | 1 | 1 | 2 | 0 | 2 | 1 | 1 | 0 | 1 | 10 |
| Costa-Ribeiro, Maux ^44^ | 1 | 1 | 0 | 1 | 1 | 3 | 0 | 2 | 1 | 1 | 1 | 1 | 13 |
| Cugusi, Solla ^45^ | 1 | 0 | 0 | 1 | 0 | 3 | 0 | 2 | 1 | 0 | 1 | 1 | 10 |
| Daneshmandi, Sayyar ^46^ | 1 | 0 | 0 | 1 | 0 | 0 | 0 | 2 | 1 | 1 | 1 | 1 | 8 |
| Dashtipour, Johnson ^47^ | 1 | 0 | 0 | 1 | 1 | 2 | 0 | 2 | 1 | 0 | 1 | 1 | 10 |
| David, Robichaud ^48^ | 1 | 0 | 0 | 1 | 1 | 2 | 0 | 2 | 1 | 0 | 0 | 1 | 9 |
| David, Robichaud ^49^ | 1 | 0 | 0 | 1 | 1 | 0 | 0 | 2 | 1 | 1 | 1 | 1 | 9 |
| de Bruin, Doan ^50^ | 1 | 0 | 0 | 1 | 1 | 1 | 0 | 2 | 1 | 1 | 0 | 1 | 9 |
| de Lima, Ferreira-Moraes ^51^ | 1 | 0 | 0 | 1 | 1 | 1 | 0 | 2 | 1 | 1 | 0 | 1 | 9 |
| de Melo, Kleiner ^52^ | 1 | 1 | 1 | 1 | 0 | 2 | 0 | 2 | 1 | 1 | 1 | 1 | 12 |
| Demonceau, Maquet ^53^ | 1 | 0 | 0 | 1 | 0 | 2 | 0 | 2 | 1 | 1 | 0 | 1 | 9 |
| Dibble, Foreman ^54^ | 1 | 0 | 0 | 1 | 1 | 2 | 1 | 2 | 1 | 1 | 1 | 1 | 12 |
| Dibble, Hale ^55^ | 1 | 0 | 0 | 1 | 1 | 2 | 0 | 2 | 1 | 1 | 1 | 1 | 11 |
| DiFrancisco-Donoghue, Lamberg ^56^ | 1 | 0 | 0 | 1 | 0 | 2 | 0 | 2 | 1 | 1 | 1 | 1 | 10 |
| Dipasquale, Meroni ^57^ | 1 | 0 | 0 | 1 | 0 | 3 | 1 | 2 | 1 | 0 | 0 | 0 | 9 |
| Droby, Maidan ^58^ | 1 | 0 | 0 | 1 | 0 | 1 | 0 | 2 | 1 | 1 | 0 | 1 | 8 |
| Duncan and Earhart ^59^ | 1 | 1 | 0 | 1 | 1 | 2 | 0 | 2 | 1 | 0 | 1 | 1 | 11 |
| Ebersbach, Ebersbach ^60^ | 1 | 0 | 0 | 1 | 1 | 2 | 0 | 2 | 1 | 1 | 1 | 1 | 11 |
| Ebersbach, Edler ^61^ | 1 | 0 | 0 | 1 | 0 | 1 | 1 | 2 | 1 | 1 | 0 | 1 | 9 |
| Ellis, de Goede ^62^ | 1 | 1 | 1 | 1 | 1 | 1 | 0 | 2 | 1 | 0 | 0 | 1 | 10 |
| El-Tamawy, Darwish ^63^ | 1 | 0 | 0 | 1 | 1 | 2 | 0 | 2 | 1 | 1 | 0 | 1 | 10 |
| Feng, Li ^64^ | 1 | 0 | 0 | 1 | 1 | 2 | 0 | 2 | 1 | 1 | 0 | 1 | 10 |
| Fernandes, Rocha ^65^ | 1 | 1 | 0 | 1 | 1 | 1 | 0 | 2 | 1 | 1 | 1 | 1 | 11 |
| Ferraz, Trippo ^66^ | 1 | 1 | 1 | 1 | 0 | 2 | 0 | 2 | 1 | 1 | 1 | 1 | 12 |
| Ferrazzoli, Ortelli ^67^ | 1 | 1 | 0 | 1 | 0 | 2 | 0 | 2 | 1 | 0 | 1 | 1 | 10 |
| Ferreira, Alves ^68^ | 1 | 1 | 0 | 1 | 0 | 2 | 0 | 2 | 1 | 0 | 0 | 1 | 9 |
| Fietzek, Schroeteler ^69^ | 1 | 1 | 0 | 1 | 1 | 2 | 0 | 2 | 1 | 0 | 0 | 1 | 10 |
| Fil-Balkan, Salci ^70^ | 1 | 0 | 0 | 1 | 0 | 1 | 0 | 2 | 1 | 1 | 0 | 1 | 8 |
| Fisher, Wu ^71^ | 1 | 0 | 0 | 1 | 1 | 2 | 0 | 2 | 1 | 1 | 1 | 1 | 11 |
| Franzoni, Monteiro ^72^ | 1 | 1 | 0 | 1 | 1 | 1 | 0 | 2 | 1 | 0 | 1 | 1 | 10 |
| Frazzitta, Bertotti ^73^ | 1 | 1 | 0 | 1 | 0 | 2 | 0 | 2 | 1 | 1 | 0 | 1 | 10 |
| Frazzitta, Bossio ^74^ | 1 | 1 | 0 | 1 | 1 | 0 | 0 | 2 | 1 | 1 | 1 | 1 | 10 |
| Frazzitta, Maestri ^75^ | 1 | 0 | 0 | 1 | 1 | 2 | 0 | 2 | 1 | 1 | 1 | 1 | 11 |
| Frazzitta, Maestri ^76^ | 1 | 0 | 0 | 1 | 0 | 2 | 0 | 2 | 1 | 1 | 0 | 1 | 9 |
| Furnari, Calabrò ^77^ | 1 | 1 | 0 | 0 | 1 | 1 | 0 | 2 | 1 | 1 | 0 | 1 | 9 |
| Galli, Cimolin ^78^ | 1 | 1 | 0 | 1 | 1 | 2 | 0 | 2 | 1 | 1 | 1 | 1 | 12 |
| Gandolfi, Geroin ^79^ | 1 | 1 | 0 | 1 | 1 | 3 | 0 | 2 | 1 | 1 | 1 | 1 | 13 |
| Gandolfi, Tinazzi ^80^ | 1 | 1 | 0 | 0 | 1 | 3 | 1 | 2 | 1 | 1 | 1 | 1 | 13 |
| Ganesan, Sathyaprabha ^81^ | 1 | 0 | 0 | 1 | 0 | 1 | 0 | 2 | 1 | 1 | 1 | 1 | 9 |
| Gao, Leung ^82^ | 1 | 0 | 0 | 1 | 1 | 2 | 0 | 2 | 1 | 0 | 0 | 1 | 9 |
| Gaßner, Steib ^83^ | 1 | 1 | 0 | 1 | 1 | 2 | 1 | 2 | 1 | 1 | 1 | 1 | 13 |
| Ghielen, van Wegen ^84^ | 1 | 0 | 1 | 1 | 1 | 2 | 0 | 2 | 1 | 1 | 0 | 1 | 11 |
| Ginis, Nieuwboer ^85^ | 1 | 1 | 0 | 1 | 0 | 2 | 1 | 2 | 1 | 1 | 0 | 1 | 11 |
| Gobbi, Pelicioni ^86^ | 1 | 0 | 0 | 1 | 0 | 1 | 1 | 2 | 1 | 0 | 0 | 1 | 8 |
| Goodwin, Richards ^87^ | 1 | 1 | 0 | 1 | 1 | 3 | 1 | 2 | 1 | 1 | 1 | 1 | 14 |
| Grobbelaar, Venter ^88^ | 1 | 0 | 0 | 0 | 1 | 1 | 0 | 2 | 1 | 1 | 1 | 1 | 9 |
| Hackney and Earhart ^89^ | 1 | 0 | 0 | 1 | 0 | 1 | 0 | 2 | 1 | 1 | 0 | 1 | 8 |
| Hackney and Earhart ^90^ | 1 | 1 | 0 | 1 | 0 | 1 | 0 | 2 | 1 | 1 | 1 | 1 | 10 |
| Hackney and Earhart ^91^ | 1 | 0 | 0 | 1 | 0 | 1 | 0 | 2 | 1 | 0 | 0 | 1 | 7 |
| Hackney, Kantorovich ^92^ | 1 | 0 | 0 | 1 | 1 | 0 | 0 | 2 | 1 | 1 | 1 | 1 | 9 |
| Harro, Shoemaker ^93^ | 1 | 1 | 0 | 1 | 1 | 2 | 0 | 2 | 1 | 1 | 1 | 1 | 12 |
| Hashimoto, Takabatake ^94^ | 1 | 1 | 0 | 0 | 0 | 1 | 0 | 2 | 1 | 0 | 0 | 1 | 7 |
| Hass, Collins ^95^ | 1 | 0 | 0 | 1 | 1 | 0 | 0 | 2 | 1 | 1 | 1 | 1 | 9 |
| Helgerud, Thomsen ^96^ | 1 | 0 | 0 | 1 | 0 | 2 | 0 | 2 | 1 | 1 | 1 | 1 | 10 |
| Hirsch, Toole ^97^ | 1 | 0 | 0 | 1 | 0 | 3 | 0 | 2 | 1 | 1 | 1 | 1 | 11 |
| Hubble, Naughton ^98^ | 1 | 1 | 0 | 1 | 1 | 2 | 1 | 2 | 1 | 1 | 1 | 1 | 13 |
| Johansson, Cameron ^99^ | 1 | 0 | 0 | 1 | 1 | 0 | 1 | 2 | 1 | 0 | 1 | 1 | 9 |
| Johansson, Freidle ^100^ | 1 | 1 | 1 | 1 | 1 | 2 | 0 | 2 | 1 | 1 | 0 | 0 | 11 |
| Joseph, Brodin ^101^ | 1 | 0 | 0 | 1 | 1 | 2 | 0 | 2 | 1 | 1 | 1 | 1 | 11 |
| Jung, Hasegawa ^102^ | 0 | 1 | 0 | 1 | 0 | 2 | 0 | 2 | 1 | 1 | 0 | 1 | 9 |
| Kadivar, Corcos ^103^ | 1 | 0 | 0 | 1 | 0 | 1 | 0 | 2 | 1 | 0 | 0 | 1 | 7 |
| Keus, Bloem ^104^ | 1 | 1 | 1 | 1 | 1 | 2 | 1 | 2 | 1 | 0 | 0 | 1 | 12 |
| Khalil, Busse ^105^ | 1 | 1 | 1 | 1 | 1 | 3 | 0 | 2 | 1 | 0 | 1 | 1 | 13 |
| Khuzema, Brammatha ^106^ | 1 | 1 | 0 | 1 | 0 | 3 | 0 | 2 | 1 | 1 | 1 | 1 | 12 |
| Kim, Kim ^107^ | 1 | 1 | 0 | 1 | 1 | 3 | 1 | 2 | 1 | 0 | 1 | 1 | 13 |
| King, Salarian ^108^ | 1 | 1 | 0 | 1 | 1 | 1 | 1 | 2 | 1 | 0 | 1 | 1 | 11 |
| Kunkel, Fitton ^109^ | 1 | 1 | 1 | 1 | 1 | 3 | 0 | 2 | 1 | 1 | 0 | 1 | 13 |
| Kurt, Büyükturan ^110^ | 1 | 1 | 1 | 1 | 0 | 2 | 0 | 2 | 1 | 1 | 0 | 1 | 11 |
| Kurtais, Kutlay ^111^ | 1 | 1 | 0 | 1 | 1 | 2 | 0 | 2 | 1 | 0 | 1 | 1 | 11 |
| Kwok, Kwan ^112^ | 0 | 1 | 1 | 0 | 0 | 2 | 1 | 2 | 1 | 1 | 1 | 1 | 11 |
| Landers, Hatlevig ^113^ | 1 | 1 | 1 | 1 | 0 | 1 | 1 | 2 | 1 | 0 | 0 | 1 | 10 |
| Leal, Abrahin ^114^ | 1 | 1 | 0 | 1 | 1 | 1 | 0 | 2 | 1 | 0 | 0 | 1 | 9 |
| Lee, Kim ^115^ | 1 | 1 | 0 | 1 | 1 | 1 | 1 | 2 | 1 | 0 | 0 | 1 | 10 |
| Lei, Ma ^116^ | 1 | 1 | 0 | 1 | 0 | 1 | 0 | 2 | 1 | 1 | 0 | 1 | 9 |
| Li, Harmer ^117^ | 1 | 1 | 0 | 1 | 0 | 3 | 1 | 2 | 1 | 1 | 1 | 1 | 13 |
| Liao, Yang ^118^ | 1 | 1 | 1 | 1 | 1 | 3 | 0 | 2 | 1 | 1 | 1 | 1 | 14 |
| Liu, Yan ^119^ | 1 | 1 | 0 | 1 | 0 | 1 | 0 | 2 | 1 | 0 | 0 | 1 | 8 |
| Löfgren, Conradsson ^120^ | 1 | 0 | 0 | 1 | 0 | 2 | 0 | 2 | 1 | 1 | 1 | 1 | 10 |
| Maidan, Nieuwhof ^121^ | 1 | 0 | 0 | 1 | 1 | 1 | 0 | 2 | 1 | 0 | 0 | 1 | 8 |
| Marumoto, Yokoyama ^122^ | 1 | 0 | 0 | 1 | 1 | 1 | 0 | 2 | 1 | 1 | 0 | 1 | 9 |
| McNeely, Mai ^123^ | 1 | 0 | 0 | 1 | 0 | 1 | 0 | 2 | 1 | 0 | 0 | 1 | 7 |
| Medijainen, Pääsuke ^124^ | 1 | 1 | 0 | 1 | 1 | 1 | 0 | 2 | 1 | 1 | 0 | 1 | 10 |
| Michels, Dubaz ^125^ | 1 | 1 | 0 | 1 | 1 | 1 | 0 | 2 | 1 | 0 | 0 | 1 | 9 |
| Miyai, Fujimoto ^126^ | 1 | 1 | 0 | 1 | 1 | 1 | 0 | 2 | 1 | 0 | 0 | 1 | 9 |
| Mollinedo-Cardalda, Cancela-Carral ^127^ | 1 | 0 | 0 | 1 | 1 | 3 | 0 | 2 | 1 | 1 | 1 | 1 | 12 |
| Monticone, Ambrosini ^128^ | 1 | 1 | 0 | 1 | 1 | 2 | 0 | 2 | 1 | 1 | 0 | 1 | 11 |
| Moon, Sarmento ^129^ | 1 | 1 | 1 | 1 | 1 | 1 | 0 | 2 | 1 | 1 | 0 | 1 | 11 |
| Moon, Schmidt ^130^ | 1 | 1 | 1 | 1 | 1 | 0 | 0 | 2 | 1 | 1 | 0 | 1 | 10 |
| Morris, Iansek ^131^ | 1 | 1 | 0 | 1 | 1 | 2 | 0 | 2 | 1 | 0 | 0 | 1 | 10 |
| Morris, Menz ^132^ | 1 | 1 | 1 | 1 | 1 | 3 | 1 | 2 | 1 | 1 | 1 | 1 | 15 |
| Morris, Taylor ^133^ | 1 | 1 | 0 | 1 | 1 | 3 | 1 | 2 | 1 | 1 | 1 | 1 | 14 |
| Morrone, Miccinilli ^134^ | 1 | 0 | 0 | 1 | 1 | 3 | 0 | 2 | 1 | 1 | 0 | 1 | 11 |
| Myers, Harrison ^135^ | 1 | 1 | 0 | 1 | 0 | 2 | 0 | 2 | 1 | 1 | 0 | 1 | 10 |
| Nadeau, Pourcher ^136^ | 1 | 1 | 0 | 1 | 1 | 2 | 0 | 2 | 1 | 1 | 1 | 1 | 12 |
| Ni, Mooney ^137^ | 1 | 0 | 0 | 0 | 0 | 3 | 0 | 2 | 1 | 1 | 1 | 1 | 10 |
| Ni, Signorile ^138^ | 1 | 0 | 0 | 1 | 0 | 3 | 0 | 2 | 1 | 1 | 1 | 1 | 11 |
| Ni, Signorile ^139^ | 1 | 0 | 0 | 1 | 0 | 2 | 0 | 2 | 1 | 1 | 1 | 1 | 10 |
| Nieuwboer, Kwakkel ^140^ | 1 | 1 | 1 | 1 | 1 | 2 | 1 | 2 | 1 | 0 | 0 | 1 | 12 |
| Nocera, Amano ^141^ | 1 | 0 | 0 | 1 | 1 | 2 | 0 | 2 | 1 | 1 | 0 | 1 | 10 |
| Oliveira, Iraci ^142^ | 1 | 0 | 0 | 1 | 1 | 1 | 0 | 2 | 1 | 0 | 0 | 1 | 8 |
| Ortiz-Rubio, Cabrera-Martos ^143^ | 1 | 1 | 0 | 1 | 1 | 3 | 0 | 2 | 1 | 1 | 1 | 1 | 13 |
| Palamara, Gotti ^144^ | 1 | 1 | 0 | 1 | 1 | 3 | 0 | 2 | 1 | 0 | 0 | 1 | 11 |
| Paolucci, Zangrando ^145^ | 1 | 1 | 1 | 1 | 1 | 2 | 0 | 2 | 1 | 1 | 0 | 1 | 12 |
| Park, Zid ^146^ | 1 | 0 | 0 | 1 | 1 | 3 | 0 | 2 | 1 | 0 | 1 | 1 | 11 |
| Paul, Canning ^147^ | 1 | 1 | 0 | 1 | 1 | 2 | 1 | 2 | 1 | 1 | 0 | 1 | 12 |
| Pazzaglia, Imbimbo ^148^ | 1 | 0 | 0 | 1 | 0 | 1 | 0 | 2 | 1 | 1 | 0 | 1 | 8 |
| Pelosin, Cerulli ^149^ | 1 | 1 | 0 | 1 | 1 | 1 | 1 | 2 | 1 | 1 | 0 | 1 | 11 |
| Pérez de la Cruz ^150^ | 1 | 1 | 0 | 1 | 0 | 3 | 0 | 2 | 1 | 1 | 1 | 1 | 12 |
| Pérez-de la Cruz ^151^ | 1 | 0 | 0 | 1 | 0 | 2 | 0 | 2 | 1 | 1 | 1 | 1 | 10 |
| Pérez-de la Cruz ^152^ | 1 | 0 | 0 | 1 | 1 | 1 | 0 | 2 | 1 | 1 | 0 | 1 | 9 |
| Picelli, Melotti ^153^ | 1 | 1 | 0 | 1 | 1 | 2 | 0 | 2 | 1 | 1 | 0 | 1 | 11 |
| Picelli, Melotti ^154^ | 1 | 1 | 0 | 1 | 0 | 2 | 0 | 2 | 1 | 1 | 0 | 1 | 10 |
| Picelli, Melotti ^155^ | 1 | 1 | 1 | 1 | 0 | 3 | 1 | 2 | 1 | 1 | 0 | 1 | 13 |
| Picelli, Varalta ^156^ | 1 | 1 | 1 | 1 | 1 | 3 | 1 | 2 | 1 | 1 | 0 | 1 | 14 |
| Pohl, Dizdar ^157^ | 1 | 1 | 0 | 1 | 1 | 2 | 0 | 2 | 1 | 0 | 0 | 1 | 10 |
| Pohl, Wressle ^158^ | 1 | 1 | 1 | 1 | 1 | 3 | 0 | 2 | 1 | 0 | 0 | 1 | 12 |
| Poier, Rodrigues Recchia ^159^ | 1 | 1 | 0 | 1 | 0 | 2 | 1 | 2 | 1 | 1 | 0 | 1 | 11 |
| Poliakoff, Galpin ^160^ | 1 | 1 | 1 | 1 | 1 | 2 | 0 | 2 | 1 | 1 | 0 | 1 | 12 |
| Pompeu, Mendes ^161^ | 1 | 0 | 0 | 1 | 1 | 2 | 0 | 2 | 1 | 1 | 0 | 1 | 10 |
| Prodoehl, Rafferty ^162^ | 1 | 0 | 0 | 1 | 1 | 1 | 0 | 2 | 1 | 0 | 1 | 1 | 9 |
| Protas, Mitchell ^163^ | 1 | 0 | 0 | 1 | 0 | 1 | 0 | 2 | 1 | 1 | 1 | 1 | 9 |
| Qutubuddin, Cifu ^164^ | 1 | 0 | 0 | 1 | 0 | 1 | 0 | 2 | 1 | 0 | 1 | 1 | 8 |
| Rafferty, Prodoehl ^165^ | 1 | 1 | 1 | 1 | 1 | 2 | 0 | 2 | 1 | 0 | 0 | 1 | 11 |
| Rennie, Opheim ^166^ | 1 | 1 | 0 | 1 | 1 | 1 | 1 | 2 | 1 | 1 | 0 | 1 | 11 |
| Ribas, Alves da Silva ^167^ | 1 | 1 | 1 | 1 | 1 | 3 | 0 | 2 | 1 | 0 | 0 | 1 | 12 |
| Ridgel and Ault ^168^ | 1 | 0 | 0 | 1 | 0 | 2 | 0 | 2 | 1 | 1 | 1 | 1 | 10 |
| Rios Romenets, Anang ^169^ | 1 | 1 | 0 | 0 | 0 | 2 | 1 | 2 | 1 | 1 | 1 | 1 | 11 |
| Rocha, Aguiar ^170^ | 1 | 1 | 0 | 1 | 1 | 3 | 1 | 2 | 1 | 0 | 0 | 1 | 12 |
| Sacheli, Neva ^171^ | 1 | 1 | 0 | 1 | 0 | 3 | 0 | 2 | 1 | 0 | 1 | 1 | 11 |
| Sage and Almeida ^172^ | 1 | 0 | 0 | 1 | 1 | 2 | 0 | 2 | 1 | 0 | 1 | 1 | 10 |
| Sale, De Pandis ^173^ | 1 | 1 | 0 | 1 | 1 | 2 | 0 | 2 | 1 | 1 | 1 | 1 | 12 |
| San Martín Valenzuela, Moscardó ^174^ | 1 | 0 | 0 | 1 | 1 | 1 | 0 | 2 | 1 | 0 | 1 | 1 | 9 |
| Sangarapillai, Norman ^175^ | 1 | 1 | 0 | 1 | 1 | 1 | 0 | 2 | 1 | 0 | 0 | 1 | 9 |
| Santos, da Silva ^176^ | 1 | 1 | 1 | 1 | 1 | 2 | 1 | 2 | 1 | 0 | 0 | 1 | 12 |
| Santos, Fernandez-Rio ^177^ | 1 | 1 | 0 | 1 | 1 | 2 | 0 | 2 | 1 | 0 | 0 | 1 | 10 |
| Santos, Fernandez-Rio ^178^ | 1 | 1 | 0 | 1 | 0 | 3 | 0 | 2 | 1 | 1 | 1 | 1 | 12 |
| Santos, Machado ^179^ | 1 | 0 | 1 | 1 | 0 | 2 | 0 | 2 | 1 | 1 | 0 | 1 | 10 |
| Schabrun, Lamont ^180^ | 1 | 1 | 1 | 0 | 1 | 3 | 1 | 2 | 1 | 1 | 1 | 1 | 14 |
| Schenkman, Cutson ^181^ | 1 | 0 | 0 | 1 | 1 | 1 | 0 | 2 | 1 | 0 | 0 | 1 | 8 |
| Schenkman, Hall ^182^ | 1 | 1 | 1 | 1 | 1 | 2 | 1 | 2 | 1 | 1 | 1 | 1 | 14 |
| Schenkman, Moore ^183^ | 1 | 1 | 0 | 1 | 0 | 3 | 1 | 2 | 1 | 0 | 1 | 1 | 12 |
| Schilling, Pfeiffer ^184^ | 0 | 0 | 0 | 1 | 0 | 1 | 0 | 2 | 0 | 0 | 1 | 1 | 6 |
| Schlenstedt, Paschen ^185^ | 1 | 0 | 0 | 1 | 1 | 1 | 0 | 2 | 1 | 1 | 0 | 1 | 9 |
| Schlenstedt, Paschen ^186^ | 1 | 1 | 0 | 1 | 1 | 2 | 0 | 2 | 1 | 1 | 1 | 1 | 12 |
| Schlick, Ernst ^187^ | 1 | 1 | 1 | 1 | 0 | 2 | 0 | 2 | 1 | 0 | 1 | 1 | 11 |
| Sedaghati, Goudarzian ^188^ | 1 | 0 | 0 | 1 | 0 | 1 | 0 | 2 | 1 | 0 | 0 | 1 | 7 |
| Serrao, Pierelli ^189^ | 1 | 1 | 0 | 1 | 1 | 1 | 1 | 2 | 1 | 1 | 0 | 1 | 11 |
| Shen and Mak ^190^ | 1 | 0 | 0 | 1 | 1 | 2 | 1 | 2 | 1 | 1 | 1 | 1 | 12 |
| Shen and Mak ^191^ | 1 | 0 | 1 | 1 | 1 | 2 | 0 | 2 | 1 | 1 | 0 | 1 | 11 |
| Shih, Wang ^192^ | 1 | 0 | 1 | 1 | 0 | 3 | 0 | 2 | 1 | 1 | 1 | 1 | 12 |
| Shulman, Katzel ^193^ | 1 | 1 | 0 | 1 | 1 | 2 | 0 | 2 | 1 | 1 | 1 | 1 | 12 |
| Silva and Israel ^194^ | 1 | 0 | 0 | 1 | 1 | 2 | 0 | 2 | 1 | 1 | 0 | 1 | 10 |
| Silva-Batista, Corcos ^195^ | 1 | 1 | 0 | 1 | 1 | 2 | 0 | 2 | 1 | 0 | 0 | 1 | 10 |
| Silva-Batista, Corcos ^196^ | 1 | 0 | 0 | 1 | 0 | 3 | 0 | 2 | 1 | 0 | 1 | 1 | 10 |
| Silva-Batista, Corcos ^197^ | 1 | 0 | 0 | 1 | 1 | 3 | 0 | 2 | 1 | 1 | 1 | 1 | 12 |
| Silva-Batista, de Brito ^198^ | 1 | 0 | 0 | 1 | 1 | 2 | 0 | 2 | 1 | 1 | 1 | 1 | 11 |
| Silva-Batista, de Lima-Pardini ^199^ | 1 | 0 | 0 | 1 | 0 | 2 | 1 | 2 | 1 | 1 | 0 | 1 | 10 |
| Smania, Corato ^200^ | 1 | 1 | 0 | 1 | 1 | 2 | 0 | 2 | 1 | 1 | 1 | 1 | 12 |
| Solla, Cugusi ^201^ | 1 | 1 | 0 | 1 | 1 | 2 | 0 | 2 | 1 | 0 | 0 | 1 | 10 |
| Song, Paul ^202^ | 1 | 1 | 0 | 1 | 1 | 3 | 0 | 2 | 1 | 1 | 1 | 1 | 13 |
| Steib, Klamroth ^203^ | 1 | 1 | 0 | 1 | 1 | 2 | 0 | 2 | 1 | 1 | 1 | 1 | 12 |
| Steib, Klamroth ^204^ | 1 | 1 | 0 | 1 | 1 | 1 | 0 | 2 | 1 | 1 | 1 | 1 | 11 |
| Stożek, Rudzińska ^205^ | 1 | 1 | 0 | 1 | 0 | 1 | 0 | 2 | 1 | 1 | 1 | 1 | 10 |
| Strand, Cherup ^206^ | 1 | 1 | 0 | 1 | 0 | 1 | 0 | 2 | 1 | 1 | 1 | 1 | 10 |
| Strouwen, Molenaar ^207^ | 1 | 1 | 0 | 1 | 1 | 3 | 1 | 2 | 1 | 1 | 0 | 1 | 13 |
| Stuckenschneider, Helmich ^208^ | 1 | 0 | 0 | 1 | 0 | 2 | 0 | 2 | 1 | 1 | 1 | 1 | 10 |
| Tang, Peng ^209^ | 1 | 1 | 0 | 1 | 0 | 2 | 0 | 2 | 1 | 0 | 1 | 1 | 10 |
| Teixeira-Machado, Araújo ^210^ | 1 | 1 | 0 | 1 | 1 | 2 | 0 | 2 | 1 | 1 | 0 | 1 | 11 |
| Terrens, Soh ^211^ | 1 | 1 | 1 | 1 | 1 | 3 | 1 | 2 | 1 | 0 | 0 | 1 | 13 |
| Thaut, McIntosh ^212^ | 1 | 1 | 0 | 1 | 0 | 2 | 0 | 2 | 1 | 1 | 1 | 1 | 11 |
| Tollár, Nagy ^213^ | 1 | 0 | 0 | 0 | 1 | 3 | 0 | 2 | 1 | 1 | 1 | 1 | 11 |
| Tollár, Nagy ^214^ | 1 | 0 | 0 | 1 | 0 | 2 | 0 | 2 | 1 | 0 | 0 | 1 | 8 |
| Tollár, Nagy ^215^ | 1 | 0 | 0 | 1 | 0 | 2 | 0 | 2 | 1 | 1 | 1 | 1 | 10 |
| Tramontano, Bonnì ^216^ | 1 | 1 | 0 | 1 | 0 | 1 | 0 | 2 | 1 | 1 | 0 | 1 | 9 |
| van den Heuvel, Kwakkel ^217^ | 1 | 1 | 1 | 1 | 1 | 3 | 1 | 2 | 1 | 0 | 0 | 1 | 13 |
| van der Kolk, de Vries ^218^ | 1 | 1 | 0 | 1 | 1 | 3 | 1 | 2 | 1 | 1 | 1 | 1 | 14 |
| van der Kolk, de Vries ^219^ | 0 | 0 | 0 | 1 | 1 | 3 | 0 | 2 | 1 | 0 | 1 | 1 | 10 |
| van Nimwegen, Speelman ^220^ | 1 | 0 | 0 | 1 | 1 | 3 | 1 | 2 | 1 | 1 | 0 | 1 | 12 |
| Van Puymbroeck, Walter ^221^ | 1 | 0 | 1 | 0 | 1 | 2 | 0 | 2 | 1 | 1 | 0 | 1 | 10 |
| Vanbellingen, Nyffeler ^222^ | 1 | 1 | 1 | 1 | 1 | 2 | 1 | 2 | 1 | 1 | 0 | 1 | 13 |
| Vergara-Diaz, Osypiuk ^223^ | 1 | 1 | 0 | 1 | 1 | 1 | 0 | 2 | 1 | 0 | 0 | 1 | 9 |
| Vieira de Moraes Filho, Chaves ^224^ | 1 | 0 | 0 | 1 | 1 | 1 | 0 | 2 | 1 | 1 | 0 | 1 | 9 |
| Vieira-Yano, Martini ^225^ | 1 | 0 | 0 | 1 | 1 | 2 | 0 | 2 | 1 | 1 | 0 | 1 | 10 |
| Vitório, Teixeira-Arroyo ^226^ | 1 | 0 | 0 | 1 | 1 | 2 | 0 | 2 | 1 | 0 | 1 | 1 | 10 |
| Vivas, Arias ^227^ | 1 | 0 | 0 | 1 | 0 | 1 | 0 | 2 | 1 | 0 | 0 | 1 | 7 |
| Volpe, Giantin ^228^ | 1 | 1 | 0 | 1 | 1 | 2 | 0 | 2 | 1 | 1 | 1 | 1 | 12 |
| Volpe, Giantin ^229^ | 1 | 1 | 0 | 1 | 1 | 2 | 0 | 2 | 1 | 1 | 0 | 1 | 11 |
| Volpe, Giantin ^230^ | 1 | 1 | 1 | 1 | 1 | 1 | 0 | 2 | 1 | 1 | 0 | 1 | 11 |
| Volpe, Signorini ^231^ | 1 | 1 | 1 | 1 | 1 | 2 | 0 | 2 | 1 | 1 | 0 | 1 | 12 |
| Wallén, Hagströmer ^232^ | 1 | 0 | 0 | 1 | 0 | 1 | 1 | 2 | 1 | 0 | 1 | 1 | 9 |
| Wang, Bi ^233^ | 1 | 1 | 0 | 1 | 0 | 1 | 0 | 2 | 1 | 1 | 0 | 1 | 9 |
| White, Wagenaar ^234^ | 1 | 1 | 0 | 1 | 1 | 2 | 0 | 2 | 1 | 1 | 0 | 1 | 11 |
| Wong-Yu and Mak ^235^ | 1 | 0 | 0 | 1 | 1 | 2 | 1 | 2 | 1 | 1 | 1 | 1 | 12 |
| Wong-Yu and Mak ^236^ | 1 | 0 | 0 | 1 | 1 | 3 | 1 | 2 | 1 | 1 | 0 | 1 | 12 |
| Xiao and Zhuang ^237^ | 1 | 0 | 0 | 1 | 1 | 2 | 0 | 2 | 1 | 1 | 1 | 1 | 11 |
| Xiao, Zhuang ^238^ | 0 | 0 | 0 | 1 | 0 | 1 | 0 | 2 | 1 | 1 | 0 | 1 | 7 |
| Yang, Cheng ^239^ | 1 | 0 | 1 | 1 | 1 | 2 | 0 | 2 | 1 | 1 | 0 | 1 | 11 |
| Yang, Lee ^240^ | 1 | 0 | 1 | 1 | 0 | 1 | 0 | 2 | 1 | 0 | 0 | 1 | 8 |
| Yang, Wang ^241^ | 1 | 0 | 0 | 1 | 1 | 2 | 1 | 2 | 1 | 1 | 1 | 1 | 12 |
| Yi-zhao, Hua ^242^ | 1 | 1 | 0 | 1 | 0 | 1 | 0 | 2 | 1 | 1 | 0 | 1 | 9 |
| Yotnuengnit, Bhidayasiri ^243^ | 1 | 0 | 0 | 1 | 1 | 2 | 0 | 2 | 1 | 0 | 0 | 1 | 9 |
| You and She ^244^ | 1 | 1 | 0 | 1 | 0 | 1 | 0 | 2 | 1 | 1 | 0 | 1 | 9 |
| Youm, Kim ^245^ | 1 | 1 | 1 | 1 | 1 | 1 | 0 | 2 | 1 | 0 | 1 | 1 | 11 |
| Yousefi, Tadibi ^246^ | 1 | 0 | 0 | 1 | 1 | 1 | 0 | 2 | 1 | 1 | 0 | 1 | 9 |
| Zeng, Tian ^247^ | 1 | 1 | 0 | 1 | 0 | 1 | 0 | 2 | 1 | 1 | 0 | 1 | 9 |
| Zhang, Hu ^248^ | 1 | 1 | 1 | 1 | 0 | 3 | 1 | 2 | 1 | 1 | 1 | 1 | 14 |
| Zhang, Zhou ^249^ | 1 | 1 | 0 | 1 | 0 | 2 | 0 | 2 | 1 | 1 | 0 | 1 | 10 |
| Zheng, Zheng ^250^ | 1 | 1 | 0 | 1 | 0 | 1 | 0 | 2 | 1 | 1 | 0 | 1 | 9 |

*1* eligibility criteria specified, *2* randomization specified, *3* allocation concealment, *4* groups similar at baseline, *5* blinding of assessor; *6* outcome measures assessed in 85% of subjects, *7* intention-to-treat analysis, *8* between group statistical comparisons reported, *9* point measures and measures of variability of outcomes reported, *10* activity monitoring in control groups reported, *11* relative exercise intensity retained constant, *12* exercise volume and energy expenditure

# Appendix 10: Results from network meta-analyses

The following shows the network plots and the league table of the secondary outcomes.

## 10.1 Secondary outcomes: balance

Figure 10.1: Network plot of balance. The size of the nodes corresponds to the number of participants randomized to each physical activity type. Physical activity type with direct comparisons are linked with a line; its thickness corresponds to the number of trials evaluating the comparison. *AE* Aerobic Exercise, *AQE* Aquatic Exercise, *BGT* Balance and Gait Training, *BGT_ECA* Balance and Gait Training with External Cue or Attention, *BGT_ICA* Balance and Gait Training with Internal Cue or Attention, *BWS_TT* Body Weight Support Treadmill Training, *CON* Control group, *CPP* Classic Physiotherapy Program, *DT_BGT* Dual Task Balance and Gait Training, *Mul_C* Multicomponent Exercise Program, *Mul_D* Multidisciplinary Exercise Program, *NW* Nordic Walking, *PT* Power Training, *RA_GT* Robotic Assisted Gait Training, *RT* Resistance Training, *TC* Tai Chi, *TT* Treadmill Training, *VR* Virtual Reality, *WBV* Whole Body Vibration

**Table 10.1: League Table of Balance**

| **BWS** | NA | NA | NA | NA | NA | NA | NA | NA | NA | NA | NA | NA | NA | NA | NA | NA | **1.13 ( 0.35; 1.91)** | NA | NA | NA | NA | NA | NA | NA |
| --- | --- | --- | --- | --- | --- | --- | --- | --- | --- | --- | --- | --- | --- | --- | --- | --- | --- | --- | --- | --- | --- | --- | --- | --- |
| 0.64 (-0.24; 1.53) | **MD** | NA | NA | NA | 0.15 (-0.44; 0.73) | -0.22 (-1.18; 0.74) | NA | 0.65 (-0.32; 1.62) | NA | NA | NA | NA | 0.80 (-0.04; 1.64) | NA | 0.61 (-0.36; 1.58) | NA | NA | NA | NA | NA | NA | **0.99 ( 0.31; 1.67)** | 0.34 (-0.66; 1.33) | NA |
| 0.63 (-0.40; 1.66) | -0.01 (-0.72; 0.71) | **PIL** | NA | NA | NA | NA | NA | NA | NA | NA | NA | NA | NA | NA | NA | NA | NA | NA | NA | NA | 0.70 (-0.17; 1.58) | NA | **0.89 ( 0.00; 1.77)** | NA |
| 0.71 (-0.28; 1.70) | 0.07 (-0.61; 0.76) | 0.08 (-0.79; 0.94) | **NW** | NA | NA | NA | NA | NA | NA | NA | NA | NA | NA | NA | NA | -0.20 (-1.12; 0.72) | 0.75 (-0.28; 1.77) | NA | NA | NA | NA | NA | **1.29 ( 0.21; 2.38)** | NA |
| 0.83 (-0.06; 1.72) | 0.19 (-0.29; 0.67) | 0.20 (-0.52; 0.92) | 0.12 (-0.57; 0.81) | **DAN** | NA | NA | NA | NA | -0.03 (-0.78; 0.72) | NA | NA | NA | NA | NA | NA | NA | NA | NA | NA | NA | NA | **0.74 ( 0.10; 1.37)** | **0.77 ( 0.34; 1.19)** | NA |
| 0.85 (-0.02; 1.72) | 0.21 (-0.16; 0.58) | 0.22 (-0.47; 0.91) | 0.14 (-0.52; 0.80) | 0.02 (-0.42; 0.46) | **AQE** | NA | NA | NA | NA | NA | NA | NA | 0.14 (-0.31; 0.59) | NA | NA | NA | NA | NA | NA | NA | 0.71 (-0.12; 1.55) | **0.43 ( 0.00; 0.85)** | **1.56 ( 0.54; 2.59)** | NA |
| **0.87 ( 0.03; 1.71)** | 0.23 (-0.18; 0.63) | 0.23 (-0.43; 0.90) | 0.15 (-0.48; 0.79) | 0.03 (-0.39; 0.46) | 0.02 (-0.35; 0.39) | **ECA** | NA | NA | NA | NA | -0.05 (-1.02; 0.92) | NA | NA | NA | NA | -0.05 (-0.73; 0.64) | 0.12 (-0.46; 0.70) | NA | NA | NA | **0.61 ( 0.28; 0.94)** | 1.04 (-0.17; 2.26) | **0.56 ( 0.12; 0.99)** | NA |
| **0.91 ( 0.01; 1.81)** | 0.27 (-0.25; 0.79) | 0.28 (-0.46; 1.01) | 0.20 (-0.51; 0.90) | 0.07 (-0.45; 0.60) | 0.06 (-0.43; 0.54) | 0.04 (-0.40; 0.49) | **RA** | NA | NA | 0.00 (-0.67; 0.67) | NA | NA | NA | NA | NA | 0.35 (-0.43; 1.12) | NA | NA | NA | NA | 0.30 (-0.26; 0.86) | NA | NA | 1.28 ( 0.37; 2.19) |
| **0.96 ( 0.02; 1.90)** | 0.32 (-0.22; 0.86) | 0.33 (-0.46; 1.12) | 0.25 (-0.51; 1.01) | 0.13 (-0.45; 0.71) | 0.11 (-0.41; 0.64) | 0.09 (-0.43; 0.62) | 0.05 (-0.56; 0.66) | **WBV** | NA | 0.11 (-0.87; 1.08) | NA | NA | NA | NA | -0.04 (-1.00; 0.91) | NA | NA | NA | NA | NA | NA | 0.64 (-0.01; 1.28) | NA | NA |
| **0.99 ( 0.12; 1.86)** | 0.35 (-0.13; 0.82) | 0.35 (-0.36; 1.06) | 0.27 (-0.40; 0.95) | 0.15 (-0.28; 0.58) | 0.14 (-0.30; 0.57) | 0.12 (-0.29; 0.53) | 0.08 (-0.43; 0.59) | 0.02 (-0.55; 0.60) | **TAN** | NA | NA | NA | NA | NA | NA | NA | 0.00 (-0.66; 0.66) | NA | NA | 0.68 (-0.05; 1.42) | NA | NA | 0.47 (-0.08; 1.02) | 0.26 (-0.42; 0.94) |
| **1.01 ( 0.17; 1.85)** | 0.37 (-0.03; 0.77) | 0.38 (-0.28; 1.04) | 0.30 (-0.33; 0.93) | 0.18 (-0.23; 0.58) | 0.16 (-0.20; 0.52) | 0.14 (-0.17; 0.46) | 0.10 (-0.31; 0.51) | 0.05 (-0.45; 0.55) | 0.02 (-0.36; 0.41) | **DT** | NA | NA | NA | NA | -0.53 (-1.10; 0.03) | NA | 0.45 (-0.35; 1.24) | NA | NA | NA | NA | NA | **0.51 ( 0.23; 0.79)** | 0.99 ( 0.49; 1.49) |
| 1.00 (-0.04; 2.04) | 0.36 (-0.37; 1.09) | 0.37 (-0.53; 1.26) | 0.29 (-0.59; 1.16) | 0.17 (-0.57; 0.90) | 0.15 (-0.56; 0.86) | 0.13 (-0.53; 0.79) | 0.09 (-0.66; 0.84) | 0.04 (-0.76; 0.84) | 0.01 (-0.71; 0.74) | -0.01 (-0.69; 0.67) | **ICA** | NA | NA | NA | NA | NA | NA | NA | NA | NA | 0.20 (-0.78; 1.17) | NA | 0.50 (-0.49; 1.48) | NA |
| **1.02 ( 0.16; 1.88)** | 0.38 (-0.06; 0.82) | 0.39 (-0.30; 1.08) | 0.31 (-0.34; 0.96) | 0.19 (-0.26; 0.63) | 0.17 (-0.23; 0.57) | 0.16 (-0.21; 0.52) | 0.11 (-0.36; 0.58) | 0.06 (-0.49; 0.61) | 0.04 (-0.39; 0.46) | 0.01 (-0.33; 0.35) | 0.02 (-0.68; 0.73) | **TC** | -0.20 (-0.97; 0.58) | NA | NA | NA | NA | 0.66 (-0.39; 1.71) | NA | NA | 0.30 (-0.74; 1.33) | NA | 0.31 (-0.12; 0.75) | 0.79 ( 0.40; 1.18) |
| **1.04 ( 0.20; 1.88)** | **0.40 ( 0.04; 0.77)** | 0.41 (-0.25; 1.07) | 0.33 (-0.29; 0.95) | 0.21 (-0.18; 0.60) | 0.19 (-0.10; 0.49) | 0.18 (-0.13; 0.48) | 0.13 (-0.30; 0.57) | 0.08 (-0.42; 0.58) | 0.06 (-0.32; 0.43) | 0.03 (-0.25; 0.31) | 0.04 (-0.63; 0.72) | 0.02 (-0.31; 0.35) | **MC** | NA | NA | 0.04 (-0.81; 0.88) | 0.29 (-0.49; 1.07) | NA | NA | NA | NA | **0.62 ( 0.00; 1.23)** | **0.45 ( 0.21; 0.70)** | NA |
| 1.08 (-0.07; 2.24) | 0.44 (-0.44; 1.32) | 0.45 (-0.58; 1.47) | 0.37 (-0.64; 1.38) | 0.25 (-0.63; 1.13) | 0.23 (-0.63; 1.09) | 0.21 (-0.63; 1.06) | 0.17 (-0.73; 1.07) | 0.12 (-0.82; 1.06) | 0.09 (-0.78; 0.97) | 0.07 (-0.77; 0.91) | 0.08 (-0.95; 1.12) | 0.06 (-0.80; 0.91) | 0.04 (-0.79; 0.87) | **PT** | NA | NA | NA | 0.03 (-0.85; 0.92) | NA | NA | NA | NA | 0.63 (-0.32; 1.57) | NA |
| **1.07 ( 0.24; 1.90)** | **0.43 ( 0.04; 0.83)** | 0.44 (-0.22; 1.10) | 0.36 (-0.26; 0.99) | 0.24 (-0.17; 0.65) | 0.22 (-0.13; 0.58) | 0.21 (-0.10; 0.52) | 0.17 (-0.27; 0.60) | 0.11 (-0.39; 0.61) | 0.09 (-0.30; 0.48) | 0.06 (-0.22; 0.34) | 0.07 (-0.60; 0.75) | 0.05 (-0.30; 0.40) | 0.03 (-0.26; 0.32) | -0.01 (-0.85; 0.84) | **VR** | -0.24 (-0.96; 0.48) | 0.11 (-0.37; 0.58) | NA | NA | NA | 0.06 (-0.32; 0.44) | **0.67 ( 0.10; 1.24)** | 0.44 (-0.29; 1.17) | 0.05 (-0.57; 0.68) |
| **1.10 ( 0.25; 1.95)** | **0.46 ( 0.02; 0.90)** | 0.47 (-0.21; 1.15) | 0.39 (-0.21; 0.99) | 0.27 (-0.18; 0.72) | 0.25 (-0.15; 0.65) | 0.24 (-0.10; 0.57) | 0.19 (-0.25; 0.64) | 0.14 (-0.41; 0.69) | 0.12 (-0.31; 0.54) | 0.09 (-0.25; 0.43) | 0.10 (-0.60; 0.80) | 0.08 (-0.30; 0.46) | 0.06 (-0.27; 0.39) | 0.02 (-0.84; 0.88) | 0.03 (-0.30; 0.36) | **AE** | -0.27 (-1.14; 0.60) | NA | NA | -0.19 (-1.40; 1.02) | 0.51 (-0.15; 1.16) | NA | 0.67 (-0.06; 1.41) | -0.04 (-0.62; 0.53) |
| **1.13 ( 0.35; 1.91)** | **0.49 ( 0.07; 0.92)** | 0.50 (-0.18; 1.18) | 0.42 (-0.19; 1.03) | 0.30 (-0.13; 0.73) | 0.28 (-0.10; 0.67) | 0.26 (-0.06; 0.59) | 0.22 (-0.23; 0.68) | 0.17 (-0.36; 0.70) | 0.14 (-0.25; 0.54) | 0.12 (-0.20; 0.44) | 0.13 (-0.56; 0.82) | 0.11 (-0.27; 0.48) | 0.09 (-0.23; 0.40) | 0.05 (-0.81; 0.90) | 0.06 (-0.24; 0.35) | 0.03 (-0.32; 0.38) | **TT** | NA | NA | NA | NA | NA | **0.95 ( 0.03; 1.86)** | 0.26 (-0.44; 0.96) |
| **1.24 ( 0.32; 2.15)** | **0.60 ( 0.07; 1.13)** | 0.61 (-0.13; 1.35) | 0.53 (-0.19; 1.25) | 0.41 (-0.13; 0.94) | 0.39 (-0.11; 0.89) | 0.37 (-0.09; 0.84) | 0.33 (-0.23; 0.89) | 0.28 (-0.35; 0.90) | 0.25 (-0.27; 0.77) | 0.23 (-0.23; 0.68) | 0.24 (-0.52; 1.00) | 0.22 (-0.26; 0.69) | 0.20 (-0.25; 0.64) | 0.16 (-0.64; 0.96) | 0.17 (-0.29; 0.62) | 0.14 (-0.35; 0.63) | 0.11 (-0.37; 0.59) | **YOG** | NA | NA | 0.16 (-0.49; 0.81) | NA | 0.29 (-0.24; 0.81) | NA |
| **1.24 ( 0.33; 2.16)** | **0.60 ( 0.09; 1.12)** | 0.61 (-0.14; 1.36) | 0.53 (-0.19; 1.25) | 0.41 (-0.11; 0.93) | 0.39 (-0.09; 0.87) | 0.38 (-0.10; 0.85) | 0.33 (-0.23; 0.90) | 0.28 (-0.33; 0.89) | 0.26 (-0.26; 0.77) | 0.23 (-0.22; 0.69) | 0.24 (-0.52; 1.00) | 0.22 (-0.27; 0.71) | 0.20 (-0.24; 0.64) | 0.16 (-0.74; 1.07) | 0.17 (-0.29; 0.63) | 0.14 (-0.35; 0.63) | 0.11 (-0.37; 0.59) | 0.00 (-0.57; 0.57) | **QIG** | NA | NA | 0.12 (-0.54; 0.79) | 0.36 (-0.13; 0.85) | NA |
| **1.26 ( 0.41; 2.11)** | **0.62 ( 0.21; 1.03)** | 0.63 (-0.04; 1.29) | 0.55 (-0.09; 1.19) | **0.43 ( 0.01; 0.84)** | **0.41 ( 0.05; 0.77)** | **0.39 ( 0.06; 0.73)** | 0.35 (-0.10; 0.80) | 0.30 (-0.22; 0.82) | 0.27 (-0.10; 0.65) | 0.25 (-0.07; 0.57) | 0.26 (-0.43; 0.94) | 0.24 (-0.13; 0.60) | 0.22 (-0.09; 0.52) | 0.18 (-0.67; 1.02) | 0.19 (-0.13; 0.50) | 0.16 (-0.20; 0.52) | 0.13 (-0.22; 0.48) | 0.02 (-0.45; 0.49) | 0.02 (-0.45; 0.48) | **RT** | 0.08 (-0.37; 0.54) | -0.05 (-0.57; 0.46) | **0.46 ( 0.06; 0.86)** | 0.64 (-0.13; 1.40) |
| **1.31 ( 0.48; 2.15)** | **0.67 ( 0.28; 1.06)** | **0.68 ( 0.05; 1.31)** | 0.60 (-0.02; 1.22) | **0.48 ( 0.08; 0.88)** | **0.46 ( 0.12; 0.80)** | **0.44 ( 0.19; 0.70)** | **0.40 ( 0.00; 0.80)** | 0.35 (-0.16; 0.86) | 0.32 (-0.06; 0.71) | **0.30 ( 0.02; 0.58)** | 0.31 (-0.34; 0.97) | 0.29 (-0.05; 0.63) | 0.27 (-0.01; 0.55) | 0.23 (-0.60; 1.06) | 0.24 (-0.02; 0.49) | 0.21 (-0.11; 0.53) | 0.18 (-0.13; 0.49) | 0.07 (-0.35; 0.50) | 0.07 (-0.38; 0.52) | 0.05 (-0.23; 0.33) | **BGT** | NA | 0.36 (-0.12; 0.85) | NA |
| **1.48 ( 0.63; 2.33)** | **0.84 ( 0.48; 1.19)** | **0.84 ( 0.18; 1.51)** | **0.76 ( 0.13; 1.40)** | **0.64 ( 0.26; 1.03)** | **0.63 ( 0.33; 0.92)** | **0.61 ( 0.28; 0.94)** | **0.57 ( 0.12; 1.02)** | **0.52 ( 0.05; 0.98)** | **0.49 ( 0.10; 0.89)** | **0.47 ( 0.16; 0.78)** | 0.48 (-0.21; 1.16) | **0.46 ( 0.09; 0.82)** | **0.43 ( 0.16; 0.71)** | 0.40 (-0.45; 1.24) | **0.40 ( 0.11; 0.70)** | **0.37 ( 0.02; 0.73)** | **0.35 ( 0.00; 0.69)** | 0.24 (-0.23; 0.71) | 0.23 (-0.19; 0.66) | 0.22 (-0.08; 0.52) | 0.17 (-0.13; 0.46) | **CPP** | 0.05 (-0.80; 0.89) | NA |
| **1.55 ( 0.72; 2.37)** | **0.91 ( 0.55; 1.26)** | **0.91 ( 0.28; 1.54)** | **0.83 ( 0.23; 1.44)** | **0.71 ( 0.37; 1.06)** | **0.70 ( 0.40; 1.00)** | **0.68 ( 0.42; 0.94)** | **0.64 ( 0.23; 1.04)** | **0.58 ( 0.10; 1.07)** | **0.56 ( 0.22; 0.89)** | **0.54 ( 0.32; 0.76)** | 0.55 (-0.10; 1.20) | **0.52 ( 0.23; 0.81)** | **0.50 ( 0.31; 0.70)** | 0.46 (-0.35; 1.28) | **0.47 ( 0.22; 0.72)** | **0.44 ( 0.14; 0.74)** | **0.42 ( 0.13; 0.70)** | 0.31 (-0.10; 0.72) | 0.30 (-0.10; 0.71) | **0.29 ( 0.03; 0.54)** | **0.23 ( 0.01; 0.46)** | 0.07 (-0.18; 0.32) | **CON** | NA |
| **1.63 ( 0.78; 2.47)** | **0.99 ( 0.57; 1.41)** | **0.99 ( 0.32; 1.67)** | **0.92 ( 0.28; 1.55)** | **0.79 ( 0.37; 1.22)** | **0.78 ( 0.40; 1.15)** | **0.76 ( 0.42; 1.10)** | **0.72 ( 0.29; 1.14)** | **0.67 ( 0.14; 1.19)** | **0.64 ( 0.26; 1.03)** | **0.62 ( 0.33; 0.90)** | 0.63 (-0.06; 1.32) | **0.61 ( 0.31; 0.90)** | **0.59 ( 0.28; 0.89)** | 0.55 (-0.30; 1.39) | **0.55 ( 0.25; 0.85)** | **0.53 ( 0.20; 0.85)** | **0.50 ( 0.17; 0.83)** | 0.39 (-0.08; 0.86) | 0.38 (-0.09; 0.86) | **0.37 ( 0.04; 0.69)** | **0.32 ( 0.02; 0.62)** | 0.15 (-0.18; 0.48) | 0.08 (-0.18; 0.35) | **STR** |

All results are presented in the form of SMD (95% CrI). Physical activity types are ranked according to the surface under the curve cumulative for balance starting with the best from left to right. The results of the network meta-analysis are showed in the lower left part, and results from pairwise comparisons in the upper right half (if available). Cells shown in bold indicate significant results. *NA* not available, *SMD* standardized Mean Difference, *CrI* Credible Interval, *AE* Aerobic Exercise, *AQE* Aquatic Exercise, *BGT* Balance and Gait Training, *BWS* Body Weight Support Treadmill Training, *CON* Control group, *CPP* Classic Physiotherapy Program, *DAN* Dance, *DT* Dual Task Balance and Gait Training, *ECA* Balance and Gait Training with External Cue or Attention, *ICA* Balance and Gait Training with Internal Cue or Attention, *MC* Multicomponent Exercise Program, *MD* Multidisciplinary Exercise Program, *PIL* Pilates, *NW* Nordic Walking, *PT* Power Training, *QIG* Qigong*, RS* Robotic Assisted Gait Training, *RT* Resistance Training, *STR* Stretch, *TAN* Tango, *TC* Tai Chi, *TT* Treadmill Training, *VR* Virtual Reality, *WBV* Whole Body Vibration, *YOG* Yoga.

## 10.2 Secondary outcomes: Gait Velocity

Figure 10.2: Network plot of gait (waking test). The size of the nodes corresponds to the number of participants randomized to each physical activity type. Physical activity type with direct comparisons are linked with a line; its thickness corresponds to the number of trials evaluating the comparison. *AE* Aerobic Exercise, *AQE* Aquatic Exercise, *BGT* Balance and Gait Training, *BGT_ECA* Balance and Gait Training with External Cue or Attention, *BGT_ICA* Balance and Gait Training with Internal Cue or Attention, *BWS_TT* Body Weight Support Treadmill Training, *CON* Control group, *CPP* Classic Physiotherapy Program, *DT_BGT* Dual Task Balance and Gait Training, *Mul_C* Multicomponent Exercise Program, *Mul_D* Multidisciplinary Exercise Program, *NW* Nordic Walking, *PT* Power Training, *RA_GT* Robotic Assisted Gait Training, *RT* Resistance Training, *TC* Tai Chi, *TT* Treadmill Training, *VR* Virtual Reality, *WBV* Whole Body Vibration.

**Table 10.2: League Table of Gait Velocity**

| **BWS** | NA | NA | 0.21 (-0.73; 1.15) | NA | NA | NA | NA | NA | NA | 0.81 ( 0.30; 1.32) | NA | NA | 0.21 (-0.73; 1.15) | NA | NA | NA | NA | NA | NA | NA | 0.37 (-0.58; 1.32) | **1.15 ( 0.64; 1.67)** | NA |
| --- | --- | --- | --- | --- | --- | --- | --- | --- | --- | --- | --- | --- | --- | --- | --- | --- | --- | --- | --- | --- | --- | --- | --- |
| 0.41 (-0.07; 0.90) | **RA** | NA | -0.11 (-0.52; 0.30) | NA | NA | 0.19 (-0.74; 1.13) | NA | NA | NA | NA | NA | NA | NA | 0.70 (-0.01; 1.41) | NA | NA | NA | NA | NA | NA | 1.59 ( 0.83; 2.35) | NA | 0.29 (-0.48; 1.07) |
| **0.44 ( 0.01; 0.87)** | 0.03 (-0.35; 0.40) | **VR** | **0.38 ( 0.01; 0.75)** | NA | NA | 0.53 (-0.24; 1.30) | NA | NA | NA | NA | NA | NA | 0.77 (-0.11; 1.66) | 0.31 (-0.39; 1.01) | -0.13 (-0.80; 0.54) | NA | NA | NA | NA | NA | 0.16 (-0.23; 0.55) | 0.09 (-0.54; 0.72) | NA |
| **0.50 ( 0.11; 0.90)** | 0.09 (-0.22; 0.40) | 0.06 (-0.19; 0.31) | **TT** | NA | NA | 0.12 (-0.38; 0.63) | NA | NA | -0.20 (-0.91; 0.51) | 0.26 (-0.12; 0.64) | 0.04 (-0.46; 0.53) | -0.64 (-1.60; 0.32) | 0.31 (-0.33; 0.95) | 0.26 (-0.54; 1.06) | 0.20 (-0.28; 0.67) | NA | NA | NA | NA | NA | 0.57 (-0.29; 1.43) | **0.42 ( 0.02; 0.83)** | NA |
| 0.39 (-0.61; 1.39) | -0.02 (-1.00; 0.96) | -0.05 (-1.01; 0.91) | -0.11 (-1.06; 0.83) | **WBV** | NA | 0.14 (-0.78; 1.06) | NA | NA | NA | NA | NA | NA | NA | NA | NA | NA | NA | NA | NA | NA | NA | NA | NA |
| **0.50 ( 0.00; 1.00)** | 0.08 (-0.39; 0.56) | 0.06 (-0.36; 0.48) | -0.01 (-0.39; 0.38) | 0.11 (-0.88; 1.10) | **MD** | 0.00 (-1.03; 1.03) | NA | NA | NA | -0.10 (-0.99; 0.80) | NA | NA | 0.26 (-0.16; 0.67) | NA | NA | NA | NA | NA | NA | NA | NA | 0.00 (-0.93; 0.93) | NA |
| **0.53 ( 0.13; 0.93)** | 0.12 (-0.23; 0.46) | 0.09 (-0.18; 0.36) | 0.03 (-0.19; 0.25) | 0.14 (-0.78; 1.06) | 0.03 (-0.34; 0.41) | **DT** | NA | NA | NA | NA | NA | NA | 0.43 (-0.36; 1.23) | NA | NA | NA | NA | NA | NA | NA | **0.43 ( 0.09; 0.77)** | **0.39 ( 0.17; 0.60)** | NA |
| 0.52 (-0.18; 1.22) | 0.11 (-0.57; 0.78) | 0.08 (-0.55; 0.72) | 0.02 (-0.60; 0.64) | 0.13 (-0.97; 1.24) | 0.02 (-0.66; 0.71) | -0.01 (-0.62; 0.61) | **YOG** | NA | NA | NA | NA | NA | NA | NA | NA | 0.14 (-0.68; 0.97) | NA | NA | NA | -0.25 (-1.23; 0.73) | -0.01 (-0.99; 0.97) | **1.21 ( 0.28; 2.15)** | NA |
| 0.54 (-0.27; 1.35) | 0.12 (-0.67; 0.91) | 0.10 (-0.66; 0.86) | 0.04 (-0.70; 0.77) | 0.15 (-1.03; 1.33) | 0.04 (-0.74; 0.82) | 0.01 (-0.73; 0.75) | 0.02 (-0.92; 0.95) | **AQE** | NA | NA | NA | NA | 0.08 (-0.62; 0.78) | NA | NA | NA | NA | NA | NA | NA | NA | NA | NA |
| **0.58 ( 0.17; 0.99)** | 0.17 (-0.20; 0.53) | 0.14 (-0.15; 0.43) | 0.08 (-0.16; 0.31) | 0.19 (-0.76; 1.14) | 0.08 (-0.31; 0.47) | 0.05 (-0.19; 0.29) | 0.06 (-0.56; 0.68) | 0.04 (-0.70; 0.78) | **MC** | -0.41 (-1.23; 0.41) | NA | 0.47 (-0.24; 1.19) | 0.59 (-0.13; 1.30) | 0.04 (-0.74; 0.82) | NA | NA | NA | NA | NA | 0.01 (-0.69; 0.71) | NA | **0.32 ( 0.11; 0.52)** | NA |
| **0.60 ( 0.22; 0.98)** | 0.19 (-0.16; 0.54) | 0.16 (-0.12; 0.44) | 0.10 (-0.11; 0.31) | 0.21 (-0.73; 1.16) | 0.10 (-0.27; 0.48) | 0.07 (-0.16; 0.30) | 0.08 (-0.54; 0.69) | 0.06 (-0.68; 0.80) | 0.02 (-0.21; 0.25) | **ECA** | NA | NA | NA | 0.25 (-0.28; 0.78) | 0.08 (-0.33; 0.48) | NA | NA | NA | 0.15 (-0.42; 0.71) | NA | 0.18 (-0.29; 0.66) | **0.34 ( 0.12; 0.56)** | NA |
| **0.62 ( 0.11; 1.12)** | 0.20 (-0.26; 0.67) | 0.18 (-0.24; 0.59) | 0.11 (-0.25; 0.47) | 0.23 (-0.77; 1.22) | 0.12 (-0.38; 0.62) | 0.08 (-0.31; 0.47) | 0.09 (-0.60; 0.79) | 0.08 (-0.73; 0.88) | 0.04 (-0.36; 0.43) | 0.02 (-0.37; 0.40) | **DAN** | NA | NA | NA | NA | NA | 0.06 (-0.97; 1.09) | NA | NA | NA | NA | 0.24 (-0.30; 0.77) | NA |
| 0.62 (-0.02; 1.26) | 0.21 (-0.40; 0.82) | 0.18 (-0.39; 0.76) | 0.12 (-0.42; 0.66) | 0.23 (-0.84; 1.30) | 0.13 (-0.50; 0.76) | 0.09 (-0.46; 0.64) | 0.10 (-0.69; 0.89) | 0.08 (-0.81; 0.98) | 0.04 (-0.49; 0.58) | 0.02 (-0.52; 0.57) | 0.01 (-0.62; 0.64) | **NW** | NA | NA | NA | NA | NA | NA | NA | NA | NA | 0.13 (-0.59; 0.84) | NA |
| **0.62 ( 0.22; 1.02)** | 0.21 (-0.16; 0.57) | 0.18 (-0.11; 0.47) | 0.12 (-0.12; 0.36) | 0.23 (-0.72; 1.18) | 0.12 (-0.21; 0.46) | 0.09 (-0.15; 0.33) | 0.10 (-0.52; 0.72) | 0.08 (-0.62; 0.78) | 0.04 (-0.21; 0.29) | 0.02 (-0.22; 0.26) | 0.01 (-0.39; 0.40) | -0.00 (-0.56; 0.55) | **CPP** | NA | 0.16 (-0.15; 0.46) | NA | NA | 0.40 (-0.18; 0.97) | NA | NA | NA | **0.38 ( 0.09; 0.66)** | NA |
| **0.63 ( 0.20; 1.07)** | 0.22 (-0.16; 0.59) | 0.19 (-0.12; 0.51) | 0.13 (-0.15; 0.41) | 0.24 (-0.72; 1.20) | 0.14 (-0.29; 0.56) | 0.10 (-0.19; 0.39) | 0.11 (-0.53; 0.75) | 0.09 (-0.67; 0.86) | 0.05 (-0.24; 0.35) | 0.03 (-0.24; 0.31) | 0.02 (-0.41; 0.44) | 0.01 (-0.57; 0.59) | 0.01 (-0.29; 0.31) | **AE** | 0.09 (-0.56; 0.74) | NA | NA | NA | NA | NA | **0.50 ( 0.03; 0.96)** | **0.50 ( 0.07; 0.93)** | NA |
| **0.63 ( 0.24; 1.03)** | 0.22 (-0.13; 0.56) | 0.19 (-0.07; 0.46) | 0.13 (-0.08; 0.34) | 0.24 (-0.70; 1.19) | 0.13 (-0.24; 0.51) | 0.10 (-0.12; 0.33) | 0.11 (-0.50; 0.72) | 0.09 (-0.64; 0.82) | 0.05 (-0.18; 0.29) | 0.03 (-0.18; 0.24) | 0.02 (-0.37; 0.40) | 0.01 (-0.54; 0.56) | 0.01 (-0.20; 0.22) | -0.00 (-0.28; 0.28) | **RT** | NA | 0.00 (-0.68; 0.68) | NA | NA | -0.03 (-0.50; 0.45) | 0.27 (-0.35; 0.88) | **0.39 ( 0.15; 0.63)** | **0.61 ( 0.24; 0.97)** |
| **0.65 ( 0.00; 1.30)** | 0.24 (-0.39; 0.86) | 0.21 (-0.37; 0.80) | 0.15 (-0.41; 0.71) | 0.26 (-0.81; 1.34) | 0.15 (-0.48; 0.79) | 0.12 (-0.44; 0.68) | 0.13 (-0.53; 0.79) | 0.11 (-0.79; 1.01) | 0.07 (-0.49; 0.63) | 0.05 (-0.51; 0.61) | 0.04 (-0.60; 0.68) | 0.03 (-0.72; 0.78) | 0.03 (-0.53; 0.59) | 0.02 (-0.57; 0.60) | 0.02 (-0.54; 0.57) | **PT** | NA | NA | NA | NA | NA | 0.40 (-0.16; 0.95) | NA |
| **0.68 ( 0.09; 1.28)** | 0.27 (-0.30; 0.83) | 0.24 (-0.28; 0.76) | 0.18 (-0.31; 0.67) | 0.29 (-0.75; 1.34) | 0.18 (-0.40; 0.77) | 0.15 (-0.35; 0.65) | 0.16 (-0.60; 0.92) | 0.14 (-0.71; 1.00) | 0.10 (-0.40; 0.60) | 0.08 (-0.41; 0.58) | 0.07 (-0.48; 0.61) | 0.06 (-0.64; 0.76) | 0.06 (-0.44; 0.56) | 0.05 (-0.48; 0.57) | 0.05 (-0.42; 0.52) | 0.03 (-0.68; 0.74) | **TAN** | NA | NA | NA | NA | 0.19 (-0.59; 0.97) | NA |
| **0.72 ( 0.21; 1.22)** | 0.30 (-0.17; 0.78) | 0.28 (-0.14; 0.70) | 0.22 (-0.17; 0.60) | 0.33 (-0.67; 1.32) | 0.22 (-0.26; 0.70) | 0.19 (-0.20; 0.57) | 0.20 (-0.49; 0.88) | 0.18 (-0.61; 0.97) | 0.14 (-0.25; 0.52) | 0.12 (-0.26; 0.50) | 0.10 (-0.39; 0.60) | 0.10 (-0.53; 0.72) | 0.10 (-0.26; 0.46) | 0.09 (-0.34; 0.51) | 0.09 (-0.28; 0.46) | 0.07 (-0.57; 0.70) | 0.04 (-0.54; 0.62) | **QIG** | NA | 0.08 (-0.56; 0.72) | NA | 0.44 (-0.09; 0.97) | NA |
| **0.75 ( 0.18; 1.31)** | 0.33 (-0.21; 0.87) | 0.31 (-0.19; 0.80) | 0.24 (-0.22; 0.71) | 0.36 (-0.67; 1.39) | 0.25 (-0.31; 0.81) | 0.21 (-0.25; 0.68) | 0.22 (-0.52; 0.96) | 0.21 (-0.64; 1.05) | 0.17 (-0.31; 0.64) | 0.15 (-0.30; 0.59) | 0.13 (-0.44; 0.70) | 0.12 (-0.56; 0.81) | 0.12 (-0.35; 0.60) | 0.11 (-0.39; 0.61) | 0.11 (-0.35; 0.58) | 0.09 (-0.60; 0.78) | 0.06 (-0.58; 0.71) | 0.03 (-0.54; 0.59) | **ICA** | NA | -0.09 (-1.00; 0.83) | 0.30 (-0.30; 0.91) | NA |
| **0.75 ( 0.30; 1.21)** | 0.34 (-0.07; 0.75) | 0.32 (-0.04; 0.67) | 0.25 (-0.06; 0.56) | 0.37 (-0.60; 1.33) | 0.26 (-0.18; 0.69) | 0.22 (-0.09; 0.54) | 0.23 (-0.39; 0.86) | 0.22 (-0.55; 0.98) | 0.18 (-0.13; 0.48) | 0.16 (-0.16; 0.47) | 0.14 (-0.31; 0.58) | 0.13 (-0.45; 0.72) | 0.13 (-0.18; 0.45) | 0.12 (-0.24; 0.48) | 0.12 (-0.16; 0.41) | 0.10 (-0.48; 0.69) | 0.07 (-0.46; 0.61) | 0.04 (-0.35; 0.43) | 0.01 (-0.51; 0.53) | **TC** | 0.24 (-0.75; 1.22) | -0.22 (-0.70; 0.26) | **0.67 ( 0.19; 1.14)** |
| **0.84 ( 0.44; 1.24)** | **0.42 ( 0.07; 0.78)** | **0.40 ( 0.13; 0.66)** | **0.33 ( 0.10; 0.57)** | 0.45 (-0.50; 1.39) | 0.34 (-0.06; 0.73) | **0.31 ( 0.08; 0.53)** | 0.31 (-0.30; 0.93) | 0.30 (-0.45; 1.05) | 0.26 (-0.01; 0.52) | **0.24 ( 0.00; 0.47)** | 0.22 (-0.18; 0.62) | 0.21 (-0.35; 0.77) | 0.22 (-0.05; 0.48) | 0.20 (-0.08; 0.49) | 0.20 (-0.03; 0.44) | 0.18 (-0.38; 0.75) | 0.15 (-0.35; 0.66) | 0.12 (-0.28; 0.52) | 0.09 (-0.38; 0.56) | 0.08 (-0.24; 0.41) | **BGT** | 0.42 (-0.12; 0.97) | NA |
| **0.94 ( 0.57; 1.31)** | **0.53 ( 0.20; 0.86)** | **0.50 ( 0.26; 0.75)** | **0.44 ( 0.26; 0.62)** | 0.55 (-0.38; 1.48) | **0.44 ( 0.09; 0.80)** | **0.41 ( 0.24; 0.58)** | 0.42 (-0.18; 1.01) | 0.40 (-0.32; 1.13) | **0.36 ( 0.18; 0.54)** | **0.34 ( 0.17; 0.51)** | 0.32 (-0.03; 0.68) | 0.32 (-0.21; 0.84) | **0.32 ( 0.13; 0.51)** | **0.31 ( 0.06; 0.56)** | **0.31 ( 0.14; 0.47)** | 0.29 (-0.24; 0.82) | 0.26 (-0.21; 0.73) | 0.22 (-0.13; 0.57) | 0.20 (-0.25; 0.64) | 0.19 (-0.09; 0.46) | 0.10 (-0.10; 0.31) | **CON** | NA |
| **1.20 ( 0.71; 1.70)** | **0.79 ( 0.37; 1.21)** | **0.76 ( 0.36; 1.17)** | **0.70 ( 0.34; 1.06)** | 0.81 (-0.18; 1.80) | **0.70 ( 0.23; 1.18)** | **0.67 ( 0.30; 1.04)** | **0.68 ( 0.00; 1.36)** | 0.66 (-0.13; 1.46) | **0.62 ( 0.25; 1.00)** | **0.60 ( 0.23; 0.97)** | **0.59 ( 0.10; 1.07)** | 0.58 (-0.04; 1.20) | **0.58 ( 0.21; 0.95)** | **0.57 ( 0.16; 0.98)** | **0.57 ( 0.25; 0.89)** | 0.55 (-0.08; 1.18) | 0.52 (-0.04; 1.08) | **0.48 ( 0.02; 0.95)** | 0.46 (-0.10; 1.01) | **0.45 ( 0.08; 0.82)** | 0.37 (-0.01; 0.75) | 0.26 (-0.08; 0.61) | **STR** |

All results are presented in the form of SMD (95% CrI). Physical activity types are ranked according to the surface under the curve cumulative for gait starting with the best from left to right. The results of the network meta-analysis are showed in the lower left part, and results from pairwise comparisons in the upper right half (if available). Cells shown in bold indicate significant results. *NA* not available, *SMD* standardized Mean Difference, *CrI* Credible Interval, *AE* Aerobic Exercise, *AQE* Aquatic Exercise, *BGT* Balance and Gait Training, *BWS* Body Weight Support Treadmill Training, *CON* Control group, *CPP* Classic Physiotherapy Program, *DAN* Dance, *DT* Dual Task Balance and Gait Training, *ECA* Balance and Gait Training with External Cue or Attention, *ICA* Balance and Gait Training with Internal Cue or Attention, *MC* Multicomponent Exercise Program, *MD* Multidisciplinary Exercise Program, *NW* Nordic Walking, *PT* Power Training, *QIG* Qigong*, RS* Robotic Assisted Gait Training, *RT* Resistance Training, *STR* Stretch, *TAN* Tango, *TC* Tai Chi, *TT* Treadmill Training, *VR* Virtual Reality, *WBV* Whole Body Vibration, *YOG* Yoga

## 10.3 Secondary outcomes: Walking distance

Figure 10.3: Network plot of walking distance. The size of the nodes corresponds to the number of participants randomized to each physical activity type. Physical activity type with direct comparisons are linked with a line; its thickness corresponds to the number of trials evaluating the comparison. *AE* Aerobic Exercise, *AQE* Aquatic Exercise, *BGT* Balance and Gait Training, *BGT_ECA* Balance and Gait Training with External Cue or Attention, *BWS_TT* Body Weight Support Treadmill Training, *CON* Control group, *CPP* Classic Physiotherapy Program, *DT_BGT* Dual Task Balance and Gait Training, *Mul_C* Multicomponent Exercise Program, *Mul_D* Multidisciplinary Exercise Program, *NW* Nordic Walking, *PT* Power Training, *RA_GT* Robotic Assisted Gait Training, *RT* Resistance Training, *TC* Tai Chi, *TT* Treadmill Training, *VR* Virtual Reality, *WBV* Whole Body Vibration.

**Table 10.3: League Table of Walking distance**

| **BWS** | NA | NA | NA | **1.07 ( 0.16; 1.97)** | NA | NA | NA | NA | NA | NA | NA | NA | NA | **1.42 ( 0.66; 2.18)** | NA | NA | NA | NA | NA | NA | **1.96 ( 1.18; 2.73)** |
| --- | --- | --- | --- | --- | --- | --- | --- | --- | --- | --- | --- | --- | --- | --- | --- | --- | --- | --- | --- | --- | --- |
| 0.71 (-0.33; 1.75) | **NW** | NA | NA | 0.66 (-0.57; 1.89) | NA | NA | NA | NA | NA | NA | NA | NA | NA | NA | NA | NA | NA | NA | NA | NA | 0.93 (-0.32; 2.18) |
| **0.90 ( 0.08; 1.73)** | 0.19 (-0.88; 1.27) | **AQE** | -0.06 (-1.22; 1.11) | NA | NA | NA | NA | NA | NA | NA | NA | **0.83 ( 0.01; 1.66)** | 0.04 (-0.99; 1.08) | NA | NA | NA | 0.09 (-1.33; 1.51) | NA | NA | NA | NA |
| **1.00 ( 0.31; 1.68)** | 0.29 (-0.69; 1.26) | 0.09 (-0.57; 0.75) | **AE** | -0.22 (-1.33; 0.89) | NA | -0.07 (-1.11; 0.96) | 0.09 (-0.63; 0.80) | NA | 0.15 (-1.25; 1.54) | NA | NA | NA | NA | 0.33 (-0.64; 1.30) | NA | 0.45 (-0.40; 1.29) | NA | 0.53 (-0.16; 1.21) | NA | 0.70 (-0.45; 1.85) | **2.29 ( 1.22; 3.36)** |
| **1.04 ( 0.46; 1.63)** | 0.33 (-0.56; 1.22) | 0.14 (-0.52; 0.79) | 0.05 (-0.41; 0.50) | **TT** | NA | 0.40 (-0.52; 1.33) | -0.40 (-1.55; 0.75) | -0.06 (-1.16; 1.03) | 0.17 (-0.34; 0.69) | NA | NA | NA | NA | 0.76 (-0.29; 1.81) | 0.49 (-0.57; 1.54) | 0.23 (-0.44; 0.90) | 0.32 (-0.51; 1.15) | NA | NA | NA | **0.87 ( 0.23; 1.50)** |
| **1.00 ( 0.12; 1.88)** | 0.29 (-0.83; 1.41) | 0.10 (-0.81; 1.00) | 0.00 (-0.77; 0.78) | -0.04 (-0.77; 0.68) | **TC** | NA | NA | NA | NA | NA | NA | NA | NA | NA | NA | 0.60 (-0.15; 1.34) | NA | NA | NA | NA | 0.75 (-0.40; 1.91) |
| **1.04 ( 0.15; 1.93)** | 0.33 (-0.80; 1.45) | 0.13 (-0.77; 1.04) | 0.04 (-0.67; 0.75) | -0.00 (-0.71; 0.70) | 0.04 (-0.94; 1.02) | **RA** | NA | NA | NA | NA | NA | NA | NA | NA | NA | NA | NA | **1.29 ( 0.23; 2.36)** | NA | NA | NA |
| **1.07 ( 0.33; 1.81)** | 0.36 (-0.65; 1.37) | 0.17 (-0.56; 0.90) | 0.07 (-0.47; 0.61) | 0.03 (-0.50; 0.56) | 0.07 (-0.73; 0.88) | 0.03 (-0.79; 0.86) | **VR** | NA | 0.03 (-1.18; 1.24) | NA | NA | -1.05 (-2.27; 0.16) | NA | NA | NA | 0.43 (-0.35; 1.21) | NA | NA | 0.13 (-1.05; 1.31) | NA | **2.12 ( 1.05; 3.18)** |
| **1.12 ( 0.34; 1.90)** | 0.41 (-0.62; 1.44) | 0.21 (-0.60; 1.03) | 0.12 (-0.57; 0.81) | 0.08 (-0.51; 0.67) | 0.12 (-0.76; 1.00) | 0.08 (-0.81; 0.98) | 0.05 (-0.69; 0.78) | **DAN** | NA | -0.18 (-1.46; 1.11) | NA | NA | NA | NA | NA | NA | NA | NA | NA | NA | **0.89 ( 0.24; 1.53)** |
| **1.16 ( 0.53; 1.80)** | 0.45 (-0.48; 1.38) | 0.26 (-0.38; 0.90) | 0.17 (-0.32; 0.65) | 0.12 (-0.24; 0.48) | 0.16 (-0.58; 0.91) | 0.13 (-0.63; 0.88) | 0.09 (-0.44; 0.63) | 0.04 (-0.58; 0.67) | **RT** | NA | NA | NA | -0.02 (-0.64; 0.59) | NA | NA | 1.21 (-0.12; 2.53) | 0.01 (-0.60; 0.62) | NA | NA | NA | **1.33 ( 0.67; 1.99)** |
| **1.20 ( 0.16; 2.24)** | 0.49 (-0.75; 1.73) | 0.30 (-0.77; 1.37) | 0.20 (-0.77; 1.18) | 0.16 (-0.76; 1.08) | 0.20 (-0.91; 1.32) | 0.16 (-0.97; 1.30) | 0.13 (-0.88; 1.14) | 0.08 (-0.81; 0.98) | 0.04 (-0.89; 0.97) | **TAN** | NA | NA | NA | NA | NA | NA | NA | NA | NA | NA | 0.59 (-0.51; 1.70) |
| **1.23 ( 0.11; 2.35)** | 0.52 (-0.79; 1.83) | 0.33 (-0.73; 1.38) | 0.23 (-0.80; 1.27) | 0.19 (-0.81; 1.19) | 0.23 (-0.95; 1.42) | 0.19 (-1.00; 1.39) | 0.16 (-0.90; 1.22) | 0.11 (-1.00; 1.22) | 0.07 (-0.90; 1.04) | 0.03 (-1.28; 1.34) | **PT** | NA | 0.10 (-0.77; 0.96) | NA | NA | NA | NA | NA | NA | NA | NA |
| **1.24 ( 0.49; 2.00)** | 0.53 (-0.48; 1.55) | 0.34 (-0.25; 0.93) | 0.25 (-0.36; 0.85) | 0.20 (-0.36; 0.76) | 0.25 (-0.60; 1.09) | 0.21 (-0.65; 1.06) | 0.17 (-0.45; 0.80) | 0.13 (-0.61; 0.86) | 0.08 (-0.46; 0.62) | 0.04 (-0.97; 1.05) | 0.01 (-0.98; 1.01) | **MD** | 0.08 (-0.59; 0.75) | NA | NA | NA | 0.20 (-0.87; 1.27) | NA | 1.18 (-0.04; 2.40) | NA | 0.66 (-0.57; 1.89) |
| **1.33 ( 0.62; 2.04)** | 0.62 (-0.37; 1.60) | 0.43 (-0.18; 1.03) | 0.33 (-0.23; 0.90) | 0.29 (-0.21; 0.79) | 0.33 (-0.48; 1.14) | 0.29 (-0.53; 1.11) | 0.26 (-0.35; 0.87) | 0.21 (-0.48; 0.91) | 0.17 (-0.28; 0.61) | 0.13 (-0.85; 1.11) | 0.10 (-0.77; 0.96) | 0.09 (-0.40; 0.57) | **CPP** | NA | NA | NA | NA | NA | NA | 0.06 (-0.90; 1.02) | 0.08 (-0.93; 1.09) |
| **1.41 ( 0.80; 2.03)** | 0.70 (-0.28; 1.68) | 0.51 (-0.22; 1.24) | 0.42 (-0.12; 0.95) | 0.37 (-0.10; 0.85) | 0.41 (-0.37; 1.20) | 0.38 (-0.43; 1.18) | 0.34 (-0.28; 0.97) | 0.30 (-0.40; 0.99) | 0.25 (-0.27; 0.77) | 0.21 (-0.77; 1.19) | 0.18 (-0.87; 1.24) | 0.17 (-0.48; 0.82) | 0.08 (-0.52; 0.69) | **ECA** | NA | 0.24 (-0.81; 1.29) | 0.28 (-0.83; 1.40) | NA | NA | NA | 0.54 (-0.19; 1.28) |
| **1.53 ( 0.32; 2.73)** | 0.82 (-0.56; 2.20) | 0.62 (-0.61; 1.86) | 0.53 (-0.62; 1.68) | 0.49 (-0.57; 1.54) | 0.53 (-0.75; 1.81) | 0.49 (-0.77; 1.75) | 0.46 (-0.72; 1.64) | 0.41 (-0.80; 1.62) | 0.36 (-0.75; 1.48) | 0.33 (-1.07; 1.72) | 0.30 (-1.15; 1.75) | 0.28 (-0.91; 1.47) | 0.20 (-0.97; 1.36) | 0.11 (-1.04; 1.27) | **DT** | NA | NA | NA | NA | NA | NA |
| **1.50 ( 0.81; 2.19)** | 0.79 (-0.19; 1.77) | 0.60 (-0.12; 1.31) | 0.50 (-0.02; 1.02) | 0.46 ( 0.00; 0.92) | 0.50 (-0.14; 1.14) | 0.46 (-0.33; 1.26) | 0.43 (-0.13; 0.99) | 0.38 (-0.31; 1.07) | 0.34 (-0.16; 0.83) | 0.30 (-0.68; 1.28) | 0.27 (-0.78; 1.32) | 0.25 (-0.38; 0.89) | 0.17 (-0.42; 0.76) | 0.08 (-0.46; 0.63) | -0.03 (-1.18; 1.12) | **BGT** | 0.27 (-0.56; 1.11) | NA | NA | NA | NA |
| **1.50 ( 0.85; 2.14)** | 0.78 (-0.16; 1.73) | 0.59 (-0.05; 1.23) | 0.50 (-0.01; 1.01) | 0.45 ( 0.05; 0.86) | 0.50 (-0.25; 1.25) | 0.46 (-0.32; 1.24) | 0.43 (-0.14; 1.00) | 0.38 (-0.26; 1.01) | 0.33 (-0.06; 0.72) | 0.29 (-0.64; 1.23) | 0.26 (-0.74; 1.27) | 0.25 (-0.29; 0.79) | 0.17 (-0.34; 0.67) | 0.08 (-0.43; 0.59) | -0.03 (-1.16; 1.10) | -0.00 (-0.50; 0.50) | **MC** | NA | NA | NA | 0.21 (-0.34; 0.77) |
| **1.63 ( 0.70; 2.56)** | 0.92 (-0.24; 2.08) | 0.73 (-0.20; 1.65) | 0.64 (-0.03; 1.30) | 0.59 (-0.19; 1.37) | 0.63 (-0.37; 1.64) | 0.59 (-0.24; 1.43) | 0.56 (-0.28; 1.40) | 0.51 (-0.42; 1.45) | 0.47 (-0.33; 1.27) | 0.43 (-0.73; 1.60) | 0.40 (-0.81; 1.62) | 0.39 (-0.49; 1.27) | 0.30 (-0.55; 1.15) | 0.22 (-0.62; 1.05) | 0.10 (-1.20; 1.41) | 0.13 (-0.69; 0.96) | 0.14 (-0.68; 0.95) | **STR** | NA | NA | NA |
| **1.77 ( 0.54; 3.01)** | 1.06 (-0.35; 2.47) | 0.87 (-0.32; 2.06) | 0.77 (-0.36; 1.91) | 0.73 (-0.40; 1.85) | 0.77 (-0.51; 2.06) | 0.73 (-0.56; 2.03) | 0.70 (-0.37; 1.78) | 0.65 (-0.58; 1.88) | 0.61 (-0.51; 1.73) | 0.57 (-0.84; 1.98) | 0.54 (-0.89; 1.96) | 0.53 (-0.56; 1.61) | 0.44 (-0.69; 1.57) | 0.36 (-0.82; 1.53) | 0.24 (-1.30; 1.78) | 0.27 (-0.88; 1.42) | 0.28 (-0.86; 1.41) | 0.14 (-1.17; 1.45) | **WBV** | NA | NA |
| **1.67 ( 0.84; 2.50)** | 0.96 (-0.11; 2.03) | 0.77 (-0.04; 1.57) | **0.67 ( 0.00; 1.34)** | 0.63 (-0.03; 1.29) | 0.67 (-0.24; 1.59) | 0.63 (-0.28; 1.55) | 0.60 (-0.15; 1.36) | 0.55 (-0.26; 1.36) | 0.51 (-0.15; 1.17) | 0.47 (-0.59; 1.53) | 0.44 (-0.64; 1.52) | 0.43 (-0.31; 1.16) | 0.34 (-0.30; 0.98) | 0.26 (-0.48; 0.99) | 0.14 (-1.10; 1.39) | 0.17 (-0.56; 0.90) | 0.18 (-0.51; 0.86) | 0.04 (-0.89; 0.97) | -0.10 (-1.34; 1.13) | **QIG** | 0.07 (-0.86; 1.00) |
| **1.99 ( 1.40; 2.57)** | **1.27 ( 0.38; 2.17)** | **1.08 ( 0.45; 1.71)** | **0.99 ( 0.53; 1.45)** | **0.94 ( 0.60; 1.28)** | **0.99 ( 0.28; 1.70)** | **0.95 ( 0.20; 1.69)** | **0.92 ( 0.39; 1.44)** | **0.87 ( 0.33; 1.40)** | **0.82 ( 0.47; 1.18)** | 0.78 (-0.08; 1.65) | 0.76 (-0.23; 1.74) | **0.74 ( 0.22; 1.27)** | **0.66 ( 0.19; 1.12)** | **0.57 ( 0.11; 1.04)** | 0.46 (-0.65; 1.56) | **0.49 ( 0.01; 0.96)** | **0.49 ( 0.12; 0.86)** | 0.35 (-0.43; 1.14) | 0.22 (-0.90; 1.33) | 0.32 (-0.30; 0.93) | **CON** |

All results are presented in the form of SMD (95% CrI). Physical activity types are ranked according to the surface under the curve cumulative for walking distance starting with the best from left to right. The results of the network meta-analysis are showed in the lower left part, and results from pairwise comparisons in the upper right half (if available). Cells shown in bold indicate significant results. *NA* not available, *SMD* standardized Mean Difference, *CrI* Credible Interval, *AE* Aerobic Exercise, *AQE* Aquatic Exercise, *BGT* Balance and Gait Training, *BWS* Body Weight Support Treadmill Training, *CON* Control group, *CPP* Classic Physiotherapy Program, *DAN* Dance, *DT* Dual Task Balance and Gait Training, *ECA* Balance and Gait Training with External Cue or Attention, *MC* Multicomponent Exercise Program, *MD* Multidisciplinary Exercise Program, *NW* Nordic Walking, *PT* Power Training, *QIG* Qigong*, RS* Robotic Assisted Gait Training, *RT* Resistance Training, *STR* Stretch, *TAN* Tango, *TC* Tai Chi, *TT* Treadmill Training, *VR* Virtual Reality, *WBV* Whole Body Vibration.

## 10.4 Secondary outcomes: Freezing of gait

Figure 11.4: Network plot of freezing of gait. The size of the nodes corresponds to the number of participants randomized to each physical activity type. Physical activity type with direct comparisons are linked with a line; its thickness corresponds to the number of trials evaluating the comparison. *AQE* Aquatic Exercise, *BGT* Balance and Gait Training, *BGT_ECA* Balance and Gait Training with External Cue or Attention, *CON* Control group, *CPP* Classic Physiotherapy Program, *DT_BGT* Dual Task Balance and Gait Training, *Mul_C* Multicomponent Exercise Program, *Mul_D* Multidisciplinary Exercise Program, *PT* Power Training, *RA_GT* Robotic Assisted Gait Training, *RT* Resistance Training, *TT* Treadmill Training, *VR* Virtual Reality.

**Table 10.4: League Table of Freezing of gait**

| **RA** | -0.43 (-0.93; 0.07) | NA | NA | NA | NA | NA | NA | NA | NA | NA | NA | NA | NA | NA | NA | NA |
| --- | --- | --- | --- | --- | --- | --- | --- | --- | --- | --- | --- | --- | --- | --- | --- | --- |
| -0.43 (-0.93; 0.07) | **TT** | NA | -0.16 (-0.62; 0.30) | NA | -0.42 (-0.98; 0.13) | NA | NA | NA | NA | NA | NA | NA | NA | NA | NA | -0.70 (-1.58; 0.18) |
| -0.65 (-1.48; 0.17) | -0.22 (-0.88; 0.44) | **RT** | NA | NA | NA | NA | NA | NA | -0.33 (-1.28; 0.61) | NA | NA | NA | -0.23 (-0.90; 0.45) | NA | -0.73 (-1.51; 0.04) | -0.22 (-1.02; 0.58) |
| -0.59 (-1.27; 0.09) | -0.16 (-0.62; 0.30) | 0.06 (-0.74; 0.87) | **VR** | NA | NA | NA | NA | NA | NA | NA | NA | NA | NA | NA | NA | NA |
| -0.78 (-1.66; 0.10) | -0.35 (-1.07; 0.37) | -0.13 (-0.75; 0.50) | -0.19 (-1.05; 0.67) | **DAN** | NA | NA | NA | NA | NA | NA | NA | NA | 0.02 (-0.96; 0.99) | NA | -0.69 (-1.57; 0.19) | -0.18 (-0.91; 0.56) |
| **-0.87 (-1.56; -0.18)** | -0.44 (-0.91; 0.04) | -0.22 (-0.71; 0.28) | -0.28 (-0.94; 0.38) | -0.09 (-0.66; 0.48) | **ECA** | NA | NA | NA | -0.03 (-0.36; 0.31) | NA | NA | NA | NA | NA | NA | -0.14 (-0.41; 0.14) |
| **-0.89 (-1.77; -0.01)** | -0.46 (-1.18; 0.26) | -0.24 (-0.92; 0.44) | -0.30 (-1.16; 0.56) | -0.11 (-0.84; 0.62) | -0.02 (-0.60; 0.56) | **MC** | NA | NA | NA | NA | NA | NA | NA | NA | NA | -0.20 (-0.72; 0.32) |
| -0.89 (-1.87; 0.09) | -0.46 (-1.30; 0.38) | -0.24 (-0.99; 0.52) | -0.30 (-1.26; 0.66) | -0.11 (-0.91; 0.69) | -0.02 (-0.74; 0.70) | -0.00 (-0.85; 0.85) | **MD** | -0.11 (-0.73; 0.51) | NA | NA | NA | NA | NA | NA | -0.08 (-0.78; 0.62) | NA |
| -0.90 (-1.90; 0.11) | -0.47 (-1.34; 0.40) | -0.25 (-1.05; 0.56) | -0.31 (-1.30; 0.68) | -0.12 (-0.96; 0.73) | -0.03 (-0.78; 0.73) | -0.01 (-0.89; 0.87) | -0.01 (-0.56; 0.55) | **AQE** | NA | NA | NA | NA | NA | NA | NA | -0.46 (-1.44; 0.53) |
| **-0.92 (-1.65; -0.18)** | -0.49 (-1.03; 0.05) | -0.26 (-0.76; 0.23) | -0.33 (-1.04; 0.38) | -0.14 (-0.73; 0.45) | -0.05 (-0.34; 0.24) | -0.03 (-0.62; 0.57) | -0.03 (-0.76; 0.70) | -0.02 (-0.79; 0.75) | **BGT** | NA | NA | 0.14 (-0.68; 0.97) | NA | NA | NA | -0.33 (-0.72; 0.05) |
| -0.93 (-2.01; 0.15) | -0.50 (-1.46; 0.45) | -0.28 (-1.20; 0.65) | -0.34 (-1.41; 0.72) | -0.15 (-1.12; 0.81) | -0.06 (-0.91; 0.79) | -0.04 (-1.01; 0.92) | -0.04 (-1.10; 1.02) | -0.03 (-1.12; 1.05) | -0.01 (-0.88; 0.85) | **YOG** | NA | NA | NA | NA | NA | -0.16 (-0.97; 0.66) |
| **-0.96 (-1.78; -0.14)** | -0.53 (-1.18; 0.13) | -0.31 (-0.87; 0.26) | -0.37 (-1.17; 0.43) | -0.18 (-0.81; 0.45) | -0.09 (-0.58; 0.40) | -0.07 (-0.74; 0.60) | -0.07 (-0.79; 0.65) | -0.06 (-0.84; 0.71) | -0.04 (-0.55; 0.47) | -0.03 (-0.94; 0.89) | **QIG** | NA | NA | NA | -0.18 (-0.74; 0.38) | -0.10 (-0.60; 0.41) |
| **-0.97 (-1.86; -0.08)** | -0.54 (-1.27; 0.20) | -0.32 (-1.01; 0.38) | -0.38 (-1.25; 0.49) | -0.19 (-0.95; 0.57) | -0.10 (-0.68; 0.48) | -0.08 (-0.84; 0.68) | -0.08 (-0.95; 0.79) | -0.07 (-0.97; 0.83) | -0.05 (-0.62; 0.51) | -0.04 (-1.02; 0.95) | -0.01 (-0.70; 0.68) | **DT** | NA | NA | NA | 0.02 (-0.69; 0.73) |
| **-0.97 (-1.80; -0.15)** | -0.54 (-1.20; 0.11) | -0.32 (-0.80; 0.16) | -0.38 (-1.18; 0.42) | -0.19 (-0.78; 0.39) | -0.10 (-0.59; 0.38) | -0.08 (-0.75; 0.59) | -0.08 (-0.85; 0.69) | -0.07 (-0.89; 0.74) | -0.06 (-0.56; 0.45) | -0.04 (-0.96; 0.88) | -0.01 (-0.59; 0.56) | -0.00 (-0.70; 0.69) | **TAN** | NA | NA | 0.01 (-0.53; 0.54) |
| **-1.13 (-2.12; -0.15)** | -0.70 (-1.55; 0.15) | -0.48 (-1.29; 0.33) | -0.54 (-1.51; 0.42) | -0.35 (-1.21; 0.51) | -0.26 (-0.99; 0.46) | -0.24 (-1.10; 0.62) | -0.24 (-1.20; 0.72) | -0.23 (-1.22; 0.75) | -0.21 (-0.96; 0.53) | -0.20 (-1.26; 0.86) | -0.17 (-0.98; 0.63) | -0.16 (-1.04; 0.72) | -0.16 (-0.96; 0.64) | **PT** | NA | 0.04 (-0.64; 0.73) |
| **-1.10 (-1.92; -0.28)** | **-0.67 (-1.33; -0.02)** | -0.45 (-0.96; 0.06) | -0.51 (-1.31; 0.29) | -0.32 (-0.89; 0.25) | -0.23 (-0.72; 0.25) | -0.21 (-0.88; 0.46) | -0.21 (-0.82; 0.40) | -0.20 (-0.91; 0.50) | -0.18 (-0.69; 0.32) | -0.17 (-1.09; 0.75) | -0.14 (-0.58; 0.30) | -0.13 (-0.82; 0.56) | -0.13 (-0.68; 0.42) | 0.03 (-0.77; 0.83) | **CPP** | -0.60 (-1.47; 0.27) |
| **-1.09 (-1.80; -0.38)** | **-0.66 (-1.16; -0.16)** | **-0.44 (-0.88; 0.00)** | -0.50 (-1.18; 0.18) | -0.31 (-0.83; 0.21) | -0.22 (-0.47; 0.03) | -0.20 (-0.72; 0.32) | -0.20 (-0.87; 0.48) | -0.19 (-0.91; 0.52) | -0.17 (-0.46; 0.12) | -0.16 (-0.97; 0.66) | -0.13 (-0.55; 0.29) | -0.12 (-0.67; 0.43) | -0.12 (-0.54; 0.31) | 0.04 (-0.64; 0.73) | 0.01 (-0.41; 0.43) | **CON** |

All results are presented in the form of SMD (95% CrI). Physical activity types are ranked according to the surface under the curve cumulative for freezing of gait starting with the best from left to right. The results of the network meta-analysis are showed in the lower left part, and results from pairwise comparisons in the upper right half (if available). Cells shown in bold indicate significant results. *NA* not available, *SMD* standardized Mean Difference, *CrI* Credible Interval, *AQE* Aquatic Exercise, *BGT* Balance and Gait Training, *CON* Control group, *CPP* Classic Physiotherapy Program, *DAN* Dance, *DT* Dual Task Balance and Gait Training, *ECA* Balance and Gait Training with External Cue or Attention, *MC* Multicomponent Exercise Program, *MD* Multidisciplinary Exercise Program, *PT* Power Training, *QIG* Qigong*, RS* Robotic Assisted Gait Training, *RT* Resistance Training, *TAN* Tango, *TT* Treadmill Training, *VR* Virtual Reality, *YOG* Yoga

## 10.5 Secondary outcomes: depression

Figure 10.5: Network plot of Depression. The size of the nodes corresponds to the number of participants randomized to each physical activity type. Physical activity type with direct comparisons are linked with a line; its thickness corresponds to the number of trials evaluating the comparison. *AE* Aerobic Exercise, *AQE* Aquatic Exercise, *BGT* Balance and Gait Training, *CON* Control group, *CPP* Classic Physiotherapy Program, *DT_BGT* Dual Task Balance and Gait Training, *Mul_C* Multicomponent Exercise Program, *Mul_D* Multidisciplinary Exercise Program, *NW* Nordic Walking, *RA_GT* Robotic Assisted Gait Training, *RT* Resistance Training, *TC* Tai Chi, *TT* Treadmill Training, *VR* Virtual Reality.

**Table 11.5: League Table of Depression**

| **DAN** | NA | NA | NA | NA | NA | NA | NA | NA | NA | NA | NA | NA | NA | NA | NA | NA | NA | **-1.71 (-2.79; -0.63)** |
| --- | --- | --- | --- | --- | --- | --- | --- | --- | --- | --- | --- | --- | --- | --- | --- | --- | --- | --- |
| -0.68 (-1.99; 0.62) | **YOG** | NA | NA | -0.67 (-1.46; 0.12) | NA | NA | NA | NA | NA | NA | NA | NA | NA | NA | NA | NA | NA | -0.28 (-1.41; 0.86) |
| -0.88 (-2.13; 0.36) | -0.20 (-1.15; 0.75) | **VR** | NA | NA | NA | -0.35 (-1.34; 0.64) | NA | NA | NA | NA | NA | NA | NA | -0.44 (-1.10; 0.22) | NA | -0.44 (-1.39; 0.51) | NA | **-1.16 (-2.09; -0.23)** |
| -0.91 (-2.10; 0.29) | -0.22 (-1.02; 0.58) | -0.02 (-0.79; 0.75) | **TT** | -0.13 (-0.79; 0.53) | NA | NA | NA | NA | NA | NA | -0.61 (-1.38; 0.17) | NA | NA | NA | NA | -0.49 (-1.27; 0.28) | NA | -0.46 (-1.66; 0.75) |
| -0.99 (-2.18; 0.21) | -0.30 (-0.97; 0.37) | -0.10 (-0.88; 0.68) | -0.08 (-0.60; 0.44) | **RT** | NA | NA | NA | NA | NA | NA | NA | NA | NA | NA | NA | NA | NA | **-1.04 (-1.70; -0.37)** |
| -1.16 (-2.36; 0.05) | -0.47 (-1.37; 0.42) | -0.27 (-1.03; 0.48) | -0.25 (-0.94; 0.44) | -0.17 (-0.88; 0.54) | **TC** | NA | NA | NA | NA | NA | NA | NA | NA | NA | NA | -0.45 (-0.95; 0.05) | NA | -0.21 (-1.20; 0.77) |
| -1.15 (-2.54; 0.24) | -0.47 (-1.60; 0.67) | -0.27 (-1.04; 0.51) | -0.24 (-1.24; 0.75) | -0.16 (-1.16; 0.83) | 0.01 (-0.97; 0.99) | **DT** | NA | NA | NA | NA | NA | NA | NA | NA | -0.40 (-1.29; 0.49) | NA | NA | NA |
| **-1.21 (-2.38; -0.04)** | -0.53 (-1.38; 0.33) | -0.33 (-1.06; 0.40) | -0.30 (-0.96; 0.35) | -0.22 (-0.88; 0.44) | -0.05 (-0.69; 0.58) | -0.06 (-1.02; 0.90) | **CPP** | 0.12 (-0.84; 1.08) | NA | -0.02 (-1.05; 1.00) | NA | NA | NA | NA | NA | -0.23 (-0.81; 0.36) | NA | -0.42 (-0.96; 0.12) |
| -1.20 (-2.49; 0.08) | -0.52 (-1.52; 0.48) | -0.32 (-1.23; 0.58) | -0.30 (-1.14; 0.54) | -0.22 (-1.06; 0.62) | -0.05 (-0.89; 0.79) | -0.06 (-1.15; 1.04) | 0.00 (-0.69; 0.70) | **MD** | NA | NA | -0.31 (-1.70; 1.09) | NA | NA | NA | NA | NA | NA | -0.19 (-1.32; 0.94) |
| -1.19 (-2.77; 0.39) | -0.51 (-1.87; 0.85) | -0.31 (-1.61; 0.99) | -0.29 (-1.54; 0.97) | -0.20 (-1.46; 1.05) | -0.03 (-1.30; 1.23) | -0.04 (-1.48; 1.40) | 0.02 (-1.21; 1.25) | 0.01 (-1.32; 1.35) | **NW** | NA | NA | NA | NA | NA | NA | NA | NA | -0.52 (-1.67; 0.63) |
| -1.27 (-2.59; 0.06) | -0.58 (-1.64; 0.48) | -0.38 (-1.35; 0.59) | -0.36 (-1.26; 0.54) | -0.28 (-1.19; 0.63) | -0.11 (-1.02; 0.80) | -0.12 (-1.27; 1.04) | -0.06 (-0.82; 0.71) | -0.06 (-1.03; 0.91) | -0.07 (-1.46; 1.31) | **AQE** | 0.02 (-0.99; 1.04) | NA | NA | NA | NA | NA | NA | NA |
| **-1.27 (-2.39; -0.16)** | -0.59 (-1.36; 0.18) | -0.39 (-1.05; 0.27) | -0.37 (-0.90; 0.17) | -0.29 (-0.84; 0.27) | -0.12 (-0.69; 0.46) | -0.12 (-1.03; 0.78) | -0.06 (-0.56; 0.43) | -0.07 (-0.78; 0.64) | -0.08 (-1.26; 1.10) | -0.01 (-0.77; 0.75) | **MC** | NA | NA | NA | NA | 0.11 (-0.67; 0.89) | NA | **-0.51 (-0.82; -0.20)** |
| **-1.32 (-2.58; -0.07)** | -0.64 (-1.60; 0.33) | -0.44 (-1.27; 0.39) | -0.42 (-1.22; 0.39) | -0.34 (-1.14; 0.47) | -0.17 (-0.98; 0.64) | -0.17 (-1.20; 0.85) | -0.11 (-0.88; 0.65) | -0.12 (-1.05; 0.81) | -0.13 (-1.44; 1.18) | -0.06 (-1.05; 0.93) | -0.05 (-0.74; 0.64) | **QIG** | NA | -0.68 (-1.75; 0.39) | NA | NA | NA | -0.12 (-0.86; 0.63) |
| -1.37 (-2.86; 0.12) | -0.69 (-1.94; 0.56) | -0.49 (-1.64; 0.66) | -0.47 (-1.57; 0.64) | -0.38 (-1.51; 0.74) | -0.21 (-1.27; 0.84) | -0.22 (-1.53; 1.09) | -0.16 (-1.24; 0.91) | -0.17 (-1.38; 1.05) | -0.18 (-1.72; 1.36) | -0.11 (-1.36; 1.15) | -0.10 (-1.15; 0.95) | -0.05 (-1.24; 1.14) | **RA** | NA | NA | -0.15 (-1.11; 0.81) | NA | NA |
| **-1.44 (-2.62; -0.27)** | -0.76 (-1.62; 0.10) | -0.56 (-1.13; 0.01) | -0.54 (-1.20; 0.13) | -0.46 (-1.13; 0.21) | -0.29 (-0.94; 0.36) | -0.30 (-1.09; 0.50) | -0.24 (-0.85; 0.38) | -0.24 (-1.05; 0.57) | -0.25 (-1.49; 0.98) | -0.18 (-1.07; 0.71) | -0.17 (-0.70; 0.36) | -0.12 (-0.81; 0.56) | -0.07 (-1.16; 1.01) | **AE** | -0.00 (-0.62; 0.61) | 0.14 (-0.59; 0.87) | NA | **-0.62 (-1.20; -0.05)** |
| **-1.48 (-2.78; -0.18)** | -0.80 (-1.81; 0.22) | -0.60 (-1.33; 0.13) | -0.57 (-1.43; 0.28) | -0.49 (-1.36; 0.37) | -0.32 (-1.17; 0.52) | -0.33 (-1.07; 0.41) | -0.27 (-1.09; 0.55) | -0.28 (-1.25; 0.70) | -0.29 (-1.64; 1.06) | -0.22 (-1.25; 0.82) | -0.21 (-0.96; 0.55) | -0.16 (-1.04; 0.72) | -0.11 (-1.32; 1.10) | -0.04 (-0.60; 0.53) | **STR** | NA | NA | NA |
| **-1.52 (-2.66; -0.38)** | **-0.84 (-1.64; -0.03)** | **-0.64 (-1.27; -0.01)** | **-0.62 (-1.17; -0.06)** | -0.54 (-1.13; 0.06) | -0.36 (-0.81; 0.08) | -0.37 (-1.26; 0.52) | -0.31 (-0.80; 0.18) | -0.32 (-1.06; 0.43) | -0.33 (-1.53; 0.87) | -0.26 (-1.07; 0.56) | -0.25 (-0.67; 0.18) | -0.20 (-0.91; 0.51) | -0.15 (-1.11; 0.81) | -0.08 (-0.58; 0.43) | -0.04 (-0.78; 0.70) | **BGT** | NA | 0.14 (-0.39; 0.67) |
| **-1.71 (-3.00; -0.42)** | **-1.03 (-2.04; -0.01)** | -0.83 (-1.75; 0.10) | -0.80 (-1.67; 0.06) | -0.72 (-1.58; 0.14) | -0.55 (-1.43; 0.33) | -0.56 (-1.68; 0.56) | -0.50 (-1.33; 0.33) | -0.50 (-1.48; 0.48) | -0.52 (-1.86; 0.82) | -0.44 (-1.49; 0.60) | -0.44 (-1.19; 0.32) | -0.39 (-1.33; 0.56) | -0.34 (-1.58; 0.90) | -0.26 (-1.10; 0.57) | -0.23 (-1.23; 0.77) | -0.19 (-0.98; 0.60) | **TAN** | -0.00 (-0.70; 0.70) |
| **-1.71 (-2.79; -0.63)** | **-1.03 (-1.76; -0.30)** | **-0.83 (-1.44; -0.22)** | **-0.81 (-1.31; -0.30)** | **-0.73 (-1.23; -0.23)** | **-0.55 (-1.09; -0.02)** | -0.56 (-1.43; 0.31) | **-0.50 (-0.95; -0.05)** | -0.51 (-1.19; 0.18) | -0.52 (-1.67; 0.63) | -0.45 (-1.22; 0.32) | **-0.44 (-0.72; -0.16)** | -0.39 (-1.02; 0.24) | -0.34 (-1.37; 0.69) | -0.27 (-0.73; 0.19) | -0.23 (-0.94; 0.48) | -0.19 (-0.56; 0.18) | -0.00 (-0.70; 0.70) | **CON** |

All results are presented in the form of SMD (95% CrI). Physical activity types are ranked according to the surface under the curve cumulative for depression starting with the best from left to right. The results of the network meta-analysis are showed in the lower left part, and results from pairwise comparisons in the upper right half (if available). Cells shown in bold indicate significant results. *NA* not available, *SMD* standardized Mean Difference, *CrI* Credible Interval, *AE* Aerobic Exercise, *AQE* Aquatic Exercise, *BGT* Balance and Gait Training, *CON* Control group, *CPP* Classic Physiotherapy Program, *DAN* Dance, *DT* Dual Task Balance and Gait Training, *MC* Multicomponent Exercise Program, *MD* Multidisciplinary Exercise Program, *NW* Nordic Walking, *PT* Power Training, *QIG* Qigong*, RS* Robotic Assisted Gait Training, *RT* Resistance Training, *STR* Stretch, *TAN* Tango, *TC* Tai Chi, *TT* Treadmill Training, *VR* Virtual Reality, *WBV* Whole Body Vibration, *YOG* Yoga*.*

## 10.6 Secondary outcomes: anxiety

Figure 10.6: Network plot of Anxiety. The size of the nodes corresponds to the number of participants randomized to each physical activity type. Physical activity type with direct comparisons are linked with a line; its thickness corresponds to the number of trials evaluating the comparison. *AE* Aerobic Exercise, *BGT* Balance and Gait Training, *CON* Control group, *CPP* Classic Physiotherapy Program, *Mul_C* Multicomponent Exercise Program, *Mul_D* Multidisciplinary Exercise Program, *RT* Resistance Training, *VR* Virtual Reality.

**Table 10.6: League Table of Anxiety**

| **YOG** | NA | NA | NA | **-0.51 (-0.85; -0.17)** | NA | 0.06 (-0.71; 0.83) | NA | NA | NA | NA |
| --- | --- | --- | --- | --- | --- | --- | --- | --- | --- | --- |
| -0.13 (-0.95; 0.68) | **MD** | 0.00 (-1.19; 1.19) | NA | NA | NA | NA | NA | -0.43 (-1.07; 0.21) | NA | NA |
| -0.13 (-1.57; 1.31) | 0.00 (-1.19; 1.19) | **MC** | NA | NA | NA | NA | NA | NA | NA | NA |
| -0.34 (-1.20; 0.51) | -0.21 (-1.19; 0.77) | -0.21 (-1.75; 1.33) | **VR** | NA | NA | NA | NA | NA | -0.25 (-0.94; 0.43) | NA |
| **-0.39 (-0.71; -0.08)** | -0.26 (-1.04; 0.52) | -0.26 (-1.68; 1.16) | -0.05 (-0.87; 0.77) | **RT** | NA | -0.29 (-0.68; 0.10) | NA | NA | NA | NA |
| -0.47 (-1.21; 0.27) | -0.34 (-1.25; 0.57) | -0.34 (-1.84; 1.16) | -0.13 (-1.07; 0.81) | -0.08 (-0.78; 0.62) | **STR** | NA | NA | NA | NA | -0.20 (-0.54; 0.15) |
| **-0.53 (-0.96; -0.11)** | -0.40 (-1.10; 0.29) | -0.40 (-1.78; 0.98) | -0.19 (-0.93; 0.55) | -0.14 (-0.49; 0.21) | -0.06 (-0.66; 0.54) | **CON** | -0.07 (-1.03; 0.88) | -0.03 (-0.30; 0.23) | -0.11 (-0.40; 0.18) | -0.13 (-0.65; 0.39) |
| -0.61 (-1.65; 0.44) | -0.47 (-1.65; 0.71) | -0.47 (-2.15; 1.20) | -0.26 (-1.47; 0.94) | -0.21 (-1.23; 0.80) | -0.13 (-1.26; 0.99) | -0.07 (-1.03; 0.88) | **QIG** | NA | NA | NA |
| **-0.56 (-1.07; -0.06)** | -0.43 (-1.07; 0.21) | -0.43 (-1.78; 0.92) | -0.22 (-0.96; 0.52) | -0.17 (-0.61; 0.27) | -0.09 (-0.74; 0.56) | -0.03 (-0.29; 0.23) | 0.04 (-0.94; 1.03) | **CPP** | 0.00 (-0.29; 0.30) | NA |
| **-0.60 (-1.11; -0.08)** | -0.46 (-1.17; 0.24) | -0.46 (-1.84; 0.92) | -0.25 (-0.94; 0.43) | -0.20 (-0.65; 0.25) | -0.12 (-0.76; 0.52) | -0.06 (-0.34; 0.22) | 0.01 (-0.98; 1.01) | -0.03 (-0.31; 0.25) | **BGT** | -0.22 (-1.11; 0.66) |
| **-0.67 (-1.33; -0.02)** | -0.54 (-1.39; 0.31) | -0.54 (-2.00; 0.92) | -0.33 (-1.20; 0.54) | -0.28 (-0.89; 0.33) | -0.20 (-0.54; 0.15) | -0.14 (-0.64; 0.36) | -0.07 (-1.14; 1.01) | -0.11 (-0.66; 0.44) | -0.08 (-0.62; 0.46) | **AE** |

All results are presented in the form of SMD (95% CrI). Physical activity types are ranked according to the surface under the curve cumulative for anxiety starting with the best from left to right. The results of the network meta-analysis are showed in the lower left part, and results from pairwise comparisons in the upper right half (if available). Cells shown in bold indicate significant results. *NA* not available, *SMD* standardized Mean Difference, *CrI* Credible Interval, *AE* Aerobic Exercise, *BGT* Balance and Gait Training, *CON* Control group, *CPP* Classic Physiotherapy Program, *MC* Multicomponent Exercise Program, *MD* Multidisciplinary Exercise Program, *QIG* Qigong*, RT* Resistance Training, *STR* Stretch, *VR* Virtual Reality, *YOG* Yoga*.*

## 10.7 Secondary outcomes: sleep quality

Figure 10.7: Network plot of Sleep quality. The size of the nodes corresponds to the number of participants randomized to each physical activity type. Physical activity type with direct comparisons are linked with a line; its thickness corresponds to the number of trials evaluating the comparison. *AE* Aerobic Exercise, *BGT* Balance and Gait Training, *CON* Control group, *Mul_C* Multicomponent Exercise Program, *RT* Resistance Training, *TC* Tai Chi.

**Table 10.7: League Table of Sleep Quality**

| **RT** | NA | NA | NA | NA | NA | NA | **-1.42 (-2.60; -0.23)** |
| --- | --- | --- | --- | --- | --- | --- | --- |
| -0.48 (-2.44; 1.47) | **MC** | NA | NA | NA | NA | NA | -0.93 (-2.48; 0.62) |
| -0.60 (-2.14; 0.95) | -0.11 (-1.95; 1.73) | **QIG** | NA | NA | NA | NA | -0.82 (-1.81; 0.17) |
| -1.13 (-3.36; 1.10) | -0.65 (-3.09; 1.79) | -0.54 (-2.67; 1.59) | **STR** | -0.04 (-1.53; 1.45) | NA | NA | NA |
| -1.17 (-2.83; 0.49) | -0.69 (-2.62; 1.25) | -0.58 (-2.10; 0.95) | -0.04 (-1.53; 1.45) | **AE** | -0.45 (-2.15; 1.25) | NA | -0.24 (-1.40; 0.92) |
| -1.38 (-3.37; 0.60) | -0.90 (-3.12; 1.32) | -0.79 (-2.66; 1.09) | -0.25 (-2.42; 1.92) | -0.21 (-1.79; 1.37) | **BGT** | NA | -0.28 (-1.98; 1.43) |
| -1.45 (-3.52; 0.61) | -0.97 (-3.26; 1.33) | -0.86 (-2.82; 1.10) | -0.32 (-2.85; 2.21) | -0.28 (-2.33; 1.77) | -0.07 (-2.39; 2.25) | **YOG** | 0.04 (-1.65; 1.73) |
| **-1.42 (-2.60; -0.23)** | -0.93 (-2.48; 0.62) | -0.82 (-1.81; 0.17) | -0.28 (-2.17; 1.60) | -0.24 (-1.40; 0.92) | -0.03 (-1.62; 1.56) | 0.04 (-1.65; 1.73) | **CON** |

All results are presented in the form of SMD (95% CrI). Physical activity types are ranked according to the surface under the curve cumulative for sleep quality starting with the best from left to right. The results of the network meta-analysis are showed in the lower left part, and results from pairwise comparisons in the upper right half (if available). Cells shown in bold indicate significant results. *NA* not available, *SMD* standardized Mean Difference, *CrI* Credible Interval, *AE* Aerobic Exercise, *BGT* Balance and Gait Training, *CON* Control group, *MC* Multicomponent Exercise Program, *QIG* Qigong*, RT* Resistance Training, *STR* Stretch, *YOG* Yoga*.*

## 10.8 Secondary outcomes: cognition

Figure 10.8: Network plot of cognition. The size of the nodes corresponds to the number of participants randomized to each physical activity type. Physical activity type with direct comparisons are linked with a line; its thickness corresponds to the number of trials evaluating the comparison. *AE* Aerobic Exercise, *BGT* Balance and Gait Training, *BGT_ECA* Balance and Gait Training with External Cue or Attention, *BGT_ICA* Balance and Gait Training with Internal Cue or Attention, *CON* Control group, *CPP* Classic Physiotherapy Program, *DT_BGT* Dual Task Balance and Gait Training, *Mul_C* Multicomponent Exercise Program, *Mul_D* Multidisciplinary Exercise Program, *PT* Power Training, *RT* Resistance Training, *TC* Tai Chi, *TT* Treadmill Training, *VR* Virtual Reality.

**Table 10.8: League Table of Cognition**

| DT | NA | NA | NA | NA | NA | NA | NA | NA | NA | NA | NA | NA | NA | 0.50 (-0.41; 1.42) | 0.87 (-0.66; 2.40) | NA | NA | NA |
| --- | --- | --- | --- | --- | --- | --- | --- | --- | --- | --- | --- | --- | --- | --- | --- | --- | --- | --- |
| 0.13 (-0.80; 1.07) | RT | NA | NA | 0.12 (-0.69; 0.94) | NA | 0.23 (-1.00; 1.45) | 0.21 (-1.19; 1.60) | NA | NA | NA | NA | -0.61 (-1.96; 0.74) | NA | NA | **0.71 ( 0.14; 1.28)** | 0.35 (-0.87; 1.57) | NA | NA |
| 0.10 (-0.97; 1.17) | -0.04 (-0.81; 0.74) | TAN | 0.49 (-0.74; 1.72) | NA | NA | NA | NA | 0.39 (-0.33; 1.11) | NA | NA | NA | NA | NA | NA | 0.39 (-0.57; 1.35) | NA | NA | NA |
| 0.12 (-0.92; 1.17) | -0.01 (-0.75; 0.73) | 0.03 (-0.75; 0.80) | DAN | NA | NA | NA | NA | 0.52 (-0.72; 1.75) | NA | NA | NA | NA | NA | NA | 0.55 (-0.19; 1.29) | 0.96 (-0.28; 2.19) | NA | NA |
| 0.19 (-0.74; 1.13) | 0.06 (-0.47; 0.59) | 0.10 (-0.70; 0.90) | 0.07 (-0.69; 0.83) | MC | NA | NA | NA | NA | NA | NA | NA | NA | NA | 0.01 (-0.91; 0.93) | **0.70 ( 0.04; 1.35)** | NA | 0.42 (-0.50; 1.33) | NA |
| 0.29 (-1.34; 1.92) | 0.16 (-1.29; 1.61) | 0.19 (-1.34; 1.72) | 0.17 (-1.35; 1.68) | 0.10 (-1.37; 1.56) | QIG | NA | NA | NA | NA | NA | NA | NA | NA | NA | 0.36 (-1.03; 1.74) | NA | NA | NA |
| 0.42 (-0.61; 1.45) | 0.28 (-0.39; 0.96) | 0.32 (-0.58; 1.22) | 0.29 (-0.58; 1.16) | 0.22 (-0.52; 0.97) | 0.13 (-1.39; 1.65) | AE | 0.03 (-0.77; 0.82) | NA | NA | NA | NA | NA | NA | 0.19 (-1.14; 1.52) | 0.17 (-0.55; 0.89) | NA | NA | NA |
| 0.42 (-0.77; 1.60) | 0.29 (-0.57; 1.14) | 0.32 (-0.75; 1.39) | 0.29 (-0.75; 1.34) | 0.22 (-0.71; 1.16) | 0.13 (-1.50; 1.76) | 0.00 (-0.71; 0.71) | STR | NA | NA | NA | NA | NA | NA | NA | NA | NA | NA | NA |
| 0.43 (-0.61; 1.46) | 0.29 (-0.43; 1.02) | 0.33 (-0.29; 0.95) | 0.30 (-0.44; 1.04) | 0.23 (-0.52; 0.98) | 0.14 (-1.37; 1.64) | 0.01 (-0.85; 0.86) | 0.01 (-1.03; 1.04) | TC | NA | NA | NA | NA | NA | NA | 0.25 (-0.49; 1.00) | NA | NA | NA |
| 0.47 (-0.59; 1.52) | 0.33 (-0.47; 1.14) | 0.37 (-0.58; 1.32) | 0.34 (-0.58; 1.26) | 0.27 (-0.55; 1.09) | 0.18 (-1.37; 1.73) | 0.05 (-0.87; 0.97) | 0.05 (-1.04; 1.14) | 0.04 (-0.87; 0.95) | ECA | NA | -0.05 (-0.90; 0.80) | NA | NA | 0.68 (-0.52; 1.87) | -0.12 (-0.97; 0.72) | NA | NA | NA |
| 0.48 (-0.78; 1.74) | 0.35 (-0.68; 1.37) | 0.38 (-0.75; 1.52) | 0.35 (-0.76; 1.47) | 0.28 (-0.76; 1.32) | 0.19 (-1.48; 1.86) | 0.06 (-1.06; 1.18) | 0.06 (-1.20; 1.32) | 0.05 (-1.05; 1.15) | 0.01 (-1.15; 1.17) | YOG | NA | NA | NA | NA | 0.17 (-0.76; 1.10) | NA | NA | NA |
| 0.48 (-0.76; 1.72) | 0.35 (-0.68; 1.37) | 0.38 (-0.76; 1.53) | 0.35 (-0.77; 1.48) | 0.28 (-0.76; 1.32) | 0.19 (-1.49; 1.87) | 0.06 (-1.06; 1.18) | 0.06 (-1.21; 1.33) | 0.05 (-1.06; 1.16) | 0.01 (-0.80; 0.83) | -0.00 (-1.32; 1.32) | ICA | NA | NA | NA | 0.06 (-1.18; 1.30) | NA | NA | NA |
| 0.49 (-0.47; 1.45) | 0.36 (-0.27; 0.99) | 0.39 (-0.45; 1.24) | 0.37 (-0.44; 1.18) | 0.30 (-0.36; 0.95) | 0.20 (-1.29; 1.69) | 0.07 (-0.72; 0.87) | 0.07 (-0.91; 1.06) | 0.07 (-0.73; 0.86) | 0.02 (-0.83; 0.88) | 0.01 (-1.06; 1.09) | 0.01 (-1.06; 1.08) | VR | NA | 0.04 (-1.17; 1.26) | -0.05 (-0.87; 0.77) | NA | 0.23 (-0.56; 1.02) | NA |
| 0.61 (-0.93; 2.15) | 0.48 (-0.88; 1.83) | 0.51 (-0.93; 1.95) | 0.48 (-0.94; 1.91) | 0.41 (-0.95; 1.78) | 0.32 (-1.57; 2.21) | 0.19 (-1.24; 1.62) | 0.19 (-1.35; 1.73) | 0.18 (-1.23; 1.60) | 0.14 (-1.32; 1.60) | 0.13 (-1.46; 1.72) | 0.13 (-1.46; 1.73) | 0.12 (-1.28; 1.51) | PT | NA | 0.04 (-1.25; 1.32) | NA | NA | NA |
| 0.58 (-0.21; 1.37) | 0.45 (-0.13; 1.02) | 0.48 (-0.30; 1.27) | 0.46 (-0.29; 1.20) | 0.38 (-0.19; 0.95) | 0.29 (-1.17; 1.75) | 0.16 (-0.56; 0.88) | 0.16 (-0.77; 1.09) | 0.15 (-0.58; 0.89) | 0.11 (-0.63; 0.86) | 0.10 (-0.93; 1.13) | 0.10 (-0.90; 1.10) | 0.09 (-0.52; 0.70) | -0.03 (-1.39; 1.33) | BGT | -0.13 (-0.80; 0.54) | 0.31 (-0.46; 1.07) | 0.41 (-0.51; 1.32) | NA |
| 0.65 (-0.20; 1.50) | **0.51 ( 0.09; 0.94)** | 0.55 (-0.10; 1.20) | 0.52 (-0.09; 1.13) | 0.45 (-0.01; 0.92) | 0.36 (-1.03; 1.74) | 0.23 (-0.39; 0.85) | 0.23 (-0.63; 1.08) | 0.22 (-0.37; 0.81) | 0.18 (-0.51; 0.87) | 0.17 (-0.76; 1.10) | 0.17 (-0.77; 1.11) | 0.16 (-0.38; 0.69) | 0.04 (-1.25; 1.32) | 0.07 (-0.38; 0.51) | CON | 0.36 (-0.22; 0.94) | 0.09 (-0.90; 1.08) | 0.13 (-0.67; 0.94) |
| 0.74 (-0.18; 1.66) | **0.61 ( 0.04; 1.17)** | 0.64 (-0.13; 1.42) | 0.62 (-0.09; 1.33) | 0.55 (-0.06; 1.15) | 0.45 (-1.00; 1.91) | 0.32 (-0.42; 1.06) | 0.32 (-0.62; 1.26) | 0.32 (-0.41; 1.04) | 0.28 (-0.53; 1.08) | 0.26 (-0.77; 1.29) | 0.26 (-0.77; 1.29) | 0.25 (-0.41; 0.91) | 0.13 (-1.23; 1.49) | 0.16 (-0.38; 0.71) | 0.10 (-0.34; 0.53) | CPP | NA | 0.47 (-0.11; 1.05) |
| 0.85 (-0.10; 1.80) | **0.71 ( 0.08; 1.35)** | 0.75 (-0.08; 1.58) | 0.72 (-0.07; 1.52) | **0.65 ( 0.04; 1.27)** | 0.56 (-0.92; 2.04) | 0.43 (-0.36; 1.22) | 0.43 (-0.55; 1.41) | 0.42 (-0.36; 1.21) | 0.38 (-0.46; 1.23) | 0.37 (-0.70; 1.44) | 0.37 (-0.70; 1.43) | 0.36 (-0.22; 0.93) | 0.24 (-1.15; 1.63) | 0.27 (-0.32; 0.85) | 0.20 (-0.32; 0.73) | 0.11 (-0.53; 0.74) | TT | -0.57 (-1.90; 0.76) |
| **0.98 ( 0.00; 1.95)** | **0.84 ( 0.20; 1.49)** | **0.88 ( 0.05; 1.71)** | **0.85 ( 0.07; 1.63)** | **0.78 ( 0.11; 1.45)** | 0.69 (-0.79; 2.17) | 0.56 (-0.24; 1.36) | 0.56 (-0.43; 1.54) | 0.55 (-0.23; 1.33) | 0.51 (-0.34; 1.36) | 0.50 (-0.57; 1.56) | 0.50 (-0.57; 1.57) | 0.49 (-0.23; 1.20) | 0.37 (-1.02; 1.76) | 0.40 (-0.24; 1.03) | 0.33 (-0.19; 0.85) | 0.24 (-0.25; 0.72) | 0.13 (-0.54; 0.80) | MD |

All results are presented in the form of SMD (95% CrI). Physical activity types are ranked according to the surface under the curve cumulative for cognition starting with the best from left to right. The results of the network meta-analysis are showed in the lower left part, and results from pairwise comparisons in the upper right half (if available). Cells shown in bold indicate significant results. *NA* not available, *SMD* standardized Mean Difference, *CrI* Credible Interval, *AE* Aerobic Exercise, *AQE* Aquatic Exercise, *BGT* Balance and Gait Training, *BWS* Body Weight Support Treadmill Training, *CON* Control group, *CPP* Classic Physiotherapy Program, *DAN* Dance, *DT* Dual Task Balance and Gait Training, *ECA* Balance and Gait Training with External Cue or Attention, *ICA* Balance and Gait Training with Internal Cue or Attention, *MC* Multicomponent Exercise Program, *MD* Multidisciplinary Exercise Program, *NW* Nordic Walking, *PT* Power Training, *QIG* Qigong*, RS* Robotic Assisted Gait Training, *RT* Resistance Training, *STR* Stretch, *TAN* Tango, *TC* Tai Chi, *TT* Treadmill Training, *VR* Virtual Reality, *WBV* Whole Body Vibration, *YOG* Yoga*.*

## 10.9 Secondary outcomes: muscle strength

Figure 10.9: Network plot of muscle strength. The size of the nodes corresponds to the number of participants randomized to each physical activity type. Physical activity type with direct comparisons are linked with a line; its thickness corresponds to the number of trials evaluating the comparison. *AE* Aerobic Exercise, *AQE* Aquatic Exercise, *BGT* Balance and Gait Training, *BGT_ECA* Balance and Gait Training with External Cue or Attention, *CON* Control group, *CPP* Classic Physiotherapy Program, *DT_BGT* Dual Task Balance and Gait Training, *Mul_C* Multicomponent Exercise Program, *Mul_D* Multidisciplinary Exercise Program, *NW* Nordic Walking, *PT* Power Training, *RA_GT* Robotic Assisted Gait Training, *RT* Resistance Training, *TC* Tai Chi, *TT* Treadmill Training, *VR* Virtual Reality, *WBV* Whole Body Vibration.

**Table 10.9: League Table of Muscle Strength**

| **PT** | NA | NA | 0.52 (-0.25; 1.29) | NA | NA | NA | NA | NA | NA | NA | 0.77 (-0.17; 1.70) | NA | NA | **0.98 ( 0.53; 1.43)** |
| --- | --- | --- | --- | --- | --- | --- | --- | --- | --- | --- | --- | --- | --- | --- |
| 0.09 (-0.36; 0.54) | **RT** | 0.02 (-0.32; 0.36) | NA | NA | 0.20 (-0.51; 0.91) | NA | 0.39 (-0.07; 0.85) | **0.39 ( 0.04; 0.73)** | NA | **0.55 ( 0.32; 0.78)** | **0.51 ( 0.14; 0.88)** | 0.25 (-0.45; 0.95) | NA | **0.96 ( 0.71; 1.21)** |
| 0.12 (-0.43; 0.68) | 0.04 (-0.30; 0.37) | **TC** | NA | NA | NA | NA | NA | **0.37 ( 0.02; 0.71)** | NA | NA | NA | NA | NA | NA |
| 0.16 (-0.43; 0.75) | 0.07 (-0.52; 0.67) | 0.04 (-0.65; 0.72) | **YOG** | NA | NA | NA | NA | NA | NA | NA | NA | NA | NA | **1.08 ( 0.46; 1.70)** |
| 0.22 (-0.65; 1.09) | 0.13 (-0.66; 0.93) | 0.10 (-0.77; 0.96) | 0.06 (-0.91; 1.03) | **DT** | NA | NA | NA | NA | NA | NA | 0.46 (-0.27; 1.18) | NA | NA | NA |
| 0.30 (-0.34; 0.93) | 0.21 (-0.27; 0.69) | 0.17 (-0.37; 0.72) | 0.14 (-0.61; 0.88) | 0.08 (-0.85; 1.00) | **AE** | NA | NA | 0.33 (-0.34; 1.01) | NA | NA | NA | NA | NA | 0.52 (-0.19; 1.24) |
| 0.38 (-0.54; 1.29) | 0.29 (-0.54; 1.12) | 0.25 (-0.64; 1.15) | 0.22 (-0.78; 1.21) | 0.16 (-0.98; 1.29) | 0.08 (-0.87; 1.03) | **MD** | NA | NA | NA | 0.18 (-0.75; 1.10) | NA | NA | NA | 0.71 (-0.24; 1.66) |
| 0.48 (-0.16; 1.12) | 0.39 (-0.07; 0.85) | 0.36 (-0.22; 0.93) | 0.32 (-0.44; 1.07) | 0.26 (-0.66; 1.18) | 0.18 (-0.48; 0.85) | 0.10 (-0.84; 1.05) | **TT** | NA | 0.07 (-0.76; 0.91) | NA | NA | NA | NA | NA |
| 0.51 (-0.04; 1.05) | **0.42 ( 0.09; 0.74)** | **0.38 ( 0.04; 0.72)** | 0.35 (-0.33; 1.02) | 0.29 (-0.57; 1.14) | 0.21 (-0.28; 0.70) | 0.13 (-0.76; 1.02) | 0.03 (-0.54; 0.59) | **STR** | NA | NA | NA | NA | NA | NA |
| 0.55 (-0.50; 1.61) | 0.46 (-0.49; 1.42) | 0.43 (-0.59; 1.44) | 0.39 (-0.74; 1.52) | 0.33 (-0.91; 1.57) | 0.25 (-0.82; 1.32) | 0.17 (-1.09; 1.44) | 0.07 (-0.76; 0.91) | 0.04 (-0.96; 1.05) | **ECA** | NA | NA | NA | NA | NA |
| **0.60 ( 0.12; 1.07)** | **0.51 ( 0.30; 0.72)** | **0.47 ( 0.07; 0.87)** | 0.43 (-0.19; 1.05) | 0.37 (-0.45; 1.19) | 0.30 (-0.22; 0.82) | 0.22 (-0.60; 1.04) | 0.12 (-0.39; 0.62) | 0.09 (-0.30; 0.47) | 0.04 (-0.93; 1.02) | **MC** | NA | **1.26 ( 0.09; 2.42)** | NA | 0.44 (-0.05; 0.93) |
| **0.68 ( 0.20; 1.16)** | **0.59 ( 0.26; 0.92)** | **0.56 ( 0.09; 1.03)** | 0.52 (-0.12; 1.15) | 0.46 (-0.27; 1.18) | 0.38 (-0.19; 0.95) | 0.30 (-0.57; 1.18) | 0.20 (-0.37; 0.77) | 0.17 (-0.29; 0.63) | 0.13 (-0.88; 1.14) | 0.08 (-0.30; 0.46) | **CPP** | NA | 0.38 (-0.10; 0.86) | 0.29 (-0.12; 0.69) |
| 0.74 (-0.01; 1.49) | **0.65 ( 0.05; 1.25)** | 0.61 (-0.08; 1.30) | 0.58 (-0.27; 1.42) | 0.52 (-0.48; 1.51) | 0.44 (-0.33; 1.21) | 0.36 (-0.66; 1.38) | 0.26 (-0.50; 1.02) | 0.23 (-0.45; 0.91) | 0.19 (-0.94; 1.32) | 0.14 (-0.48; 0.76) | 0.06 (-0.62; 0.74) | **BGT** | NA | NA |
| **1.06 ( 0.38; 1.74)** | **0.97 ( 0.39; 1.55)** | **0.94 ( 0.26; 1.61)** | **0.90 ( 0.10; 1.69)** | 0.84 (-0.03; 1.71) | **0.76 ( 0.02; 1.50)** | 0.68 (-0.32; 1.68) | 0.58 (-0.16; 1.32) | 0.55 (-0.11; 1.22) | 0.51 (-0.61; 1.63) | 0.46 (-0.15; 1.07) | 0.38 (-0.10; 0.86) | 0.32 (-0.51; 1.16) | **QIG** | NA |
| **1.04 ( 0.64; 1.44)** | **0.95 ( 0.73; 1.17)** | **0.91 ( 0.51; 1.32)** | **0.88 ( 0.32; 1.43)** | **0.82 ( 0.02; 1.61)** | **0.74 ( 0.24; 1.24)** | 0.66 (-0.16; 1.48) | **0.56 ( 0.05; 1.07)** | **0.53 ( 0.15; 0.92)** | 0.49 (-0.49; 1.47) | **0.44 ( 0.17; 0.72)** | **0.36 ( 0.03; 0.69)** | 0.30 (-0.34; 0.94) | -0.02 (-0.60; 0.56) | **CON** |

All results are presented in the form of SMD (95% CrI). Physical activity types are ranked according to the surface under the curve cumulative for muscle strength starting with the best from left to right. The results of the network meta-analysis are showed in the lower left part, and results from pairwise comparisons in the upper right half (if available). Cells shown in bold indicate significant results. *NA* not available, *SMD* standardized Mean Difference, *CrI* Credible Interval, *AE* Aerobic Exercise, *AQE* Aquatic Exercise, *BGT* Balance and Gait Training, *CON* Control group, *CPP* Classic Physiotherapy Program, *DAN* Dance, *DT* Dual Task Balance and Gait Training, *ECA* Balance and Gait Training with External Cue or Attention, *MC* Multicomponent Exercise Program, *MD* Multidisciplinary Exercise Program, *NW* Nordic Walking, *PT* Power Training, *QIG* Qigong*, RS* Robotic Assisted Gait Training, *RT* Resistance Training, *STR* Stretch, *TAN* Tango, *TC* Tai Chi, *TT* Treadmill Training, *VR* Virtual Reality, *WBV* Whole Body Vibration.

## 10.10 Secondary outcomes: concern of falling

Figure 10.10: Network plot of concern of falling. The size of the nodes corresponds to the number of participants randomized to each physical activity type. Physical activity type with direct comparisons are linked with a line; its thickness corresponds to the number of trials evaluating the comparison. *AE* Aerobic Exercise, *AQE* Aquatic Exercise, *BGT* Balance and Gait Training, *BGT_ECA* Balance and Gait Training with External Cue or Attention, *BGT_ICA* Balance and Gait Training with Internal Cue or Attention, *BWS_TT* Body Weight Support Treadmill Training, *CON* Control group, *CPP* Classic Physiotherapy Program, *DT_BGT* Dual Task Balance and Gait Training, *Mul_C* Multicomponent Exercise Program, *Mul_D* Multidisciplinary Exercise Program, *RA_GT* Robotic Assisted Gait Training, *RT* Resistance Training, *TC* Tai Chi, *TT* Treadmill Training, *VR* Virtual Reality.

**Table 10.10: League Table of Concern of falling**

| **YOG** | NA | NA | NA | -0.16 (-0.96; 0.65) | NA | NA | NA | NA | NA | NA | NA | NA | NA | NA | NA | NA | NA | NA |
| --- | --- | --- | --- | --- | --- | --- | --- | --- | --- | --- | --- | --- | --- | --- | --- | --- | --- | --- |
| -0.06 (-1.06; 0.93) | **TC** | NA | NA | NA | NA | NA | NA | NA | NA | NA | NA | NA | NA | NA | NA | -0.46 (-1.00; 0.07) | NA | NA |
| -0.14 (-0.99; 0.70) | -0.08 (-0.67; 0.51) | **ECA** | NA | -0.17 (-0.47; 0.14) | NA | NA | NA | 0.38 (-0.58; 1.34) | NA | NA | NA | -0.15 (-0.72; 0.41) | NA | NA | NA | -0.20 (-0.53; 0.13) | NA | NA |
| -0.14 (-1.05; 0.77) | -0.08 (-0.77; 0.61) | 0.00 (-0.46; 0.47) | **AQE** | 0.08 (-0.49; 0.66) | 0.04 (-0.65; 0.74) | NA | NA | NA | NA | NA | -0.22 (-0.84; 0.39) | NA | NA | NA | NA | NA | NA | NA |
| -0.16 (-0.96; 0.65) | -0.09 (-0.68; 0.49) | -0.01 (-0.26; 0.24) | -0.01 (-0.44; 0.41) | **BGT** | NA | NA | -0.12 (-0.56; 0.31) | 0.08 (-0.88; 1.04) | NA | 0.11 (-0.74; 0.97) | -0.16 (-0.73; 0.41) | -0.53 (-1.19; 0.13) | NA | NA | NA | -0.39 (-0.80; 0.01) | NA | NA |
| -0.13 (-1.18; 0.93) | -0.06 (-0.93; 0.80) | 0.02 (-0.68; 0.72) | 0.02 (-0.59; 0.62) | 0.03 (-0.65; 0.71) | **CPP** | NA | NA | NA | 0.00 (-0.97; 0.97) | NA | NA | NA | NA | -0.34 (-0.98; 0.30) | NA | NA | NA | NA |
| -0.15 (-1.06; 0.75) | -0.09 (-0.74; 0.56) | -0.01 (-0.43; 0.41) | -0.01 (-0.55; 0.54) | 0.01 (-0.40; 0.41) | -0.02 (-0.77; 0.72) | **RT** | NA | NA | NA | NA | NA | 0.07 (-0.44; 0.58) | NA | NA | NA | -0.53 (-1.07; 0.01) | NA | -0.73 (-1.48; 0.01) |
| -0.16 (-1.02; 0.71) | -0.09 (-0.71; 0.53) | -0.01 (-0.37; 0.34) | -0.01 (-0.51; 0.48) | -0.00 (-0.31; 0.31) | -0.03 (-0.75; 0.69) | -0.01 (-0.45; 0.44) | **VR** | NA | NA | 0.18 (-0.62; 0.99) | **-1.82 (-2.83; -0.80)** | -0.14 (-0.61; 0.33) | NA | NA | NA | -0.42 (-1.11; 0.27) | NA | NA |
| -0.13 (-1.16; 0.90) | -0.07 (-0.90; 0.76) | 0.01 (-0.63; 0.66) | 0.01 (-0.74; 0.76) | 0.02 (-0.62; 0.67) | -0.00 (-0.92; 0.91) | 0.02 (-0.71; 0.74) | 0.03 (-0.66; 0.71) | **ICA** | NA | NA | NA | NA | NA | NA | NA | -0.11 (-1.07; 0.85) | NA | NA |
| -0.18 (-1.17; 0.80) | -0.12 (-0.88; 0.65) | -0.04 (-0.62; 0.54) | -0.04 (-0.67; 0.59) | -0.03 (-0.59; 0.54) | -0.06 (-0.76; 0.65) | -0.03 (-0.65; 0.58) | -0.03 (-0.62; 0.57) | -0.05 (-0.88; 0.78) | **RA** | 0.00 (-0.64; 0.64) | NA | NA | NA | NA | NA | NA | NA | -0.60 (-1.44; 0.24) |
| -0.22 (-1.10; 0.66) | -0.16 (-0.78; 0.47) | -0.08 (-0.45; 0.30) | -0.08 (-0.58; 0.42) | -0.06 (-0.41; 0.29) | -0.09 (-0.79; 0.60) | -0.07 (-0.50; 0.36) | -0.06 (-0.45; 0.32) | -0.09 (-0.79; 0.61) | -0.04 (-0.53; 0.46) | **DT** | NA | -0.55 (-1.32; 0.23) | NA | NA | NA | -0.11 (-0.58; 0.36) | NA | -0.40 (-0.88; 0.08) |
| -0.24 (-1.09; 0.62) | -0.17 (-0.75; 0.40) | -0.09 (-0.39; 0.21) | -0.09 (-0.52; 0.33) | -0.08 (-0.36; 0.20) | -0.11 (-0.79; 0.57) | -0.08 (-0.48; 0.32) | -0.08 (-0.42; 0.27) | -0.10 (-0.77; 0.56) | -0.05 (-0.61; 0.51) | -0.02 (-0.37; 0.34) | **MC** | 0.04 (-0.40; 0.48) | -0.42 (-1.26; 0.41) | NA | NA | **-0.37 (-0.62; -0.12)** | NA | NA |
| -0.28 (-1.14; 0.58) | -0.21 (-0.81; 0.39) | -0.13 (-0.44; 0.18) | -0.13 (-0.61; 0.34) | -0.12 (-0.42; 0.18) | -0.15 (-0.85; 0.55) | -0.13 (-0.50; 0.25) | -0.12 (-0.44; 0.21) | -0.14 (-0.82; 0.53) | -0.09 (-0.67; 0.48) | -0.06 (-0.42; 0.30) | -0.04 (-0.33; 0.25) | **TT** | NA | NA | NA | -0.64 (-1.52; 0.24) | NA | NA |
| -0.39 (-1.38; 0.60) | -0.33 (-1.10; 0.44) | -0.25 (-0.84; 0.35) | -0.25 (-0.92; 0.42) | -0.23 (-0.81; 0.34) | -0.26 (-1.09; 0.57) | -0.24 (-0.85; 0.37) | -0.23 (-0.84; 0.37) | -0.26 (-1.09; 0.58) | -0.21 (-0.89; 0.47) | -0.17 (-0.72; 0.38) | -0.15 (-0.71; 0.40) | -0.11 (-0.70; 0.47) | **AE** | NA | NA | NA | NA | -0.49 (-1.04; 0.06) |
| -0.47 (-1.70; 0.76) | -0.41 (-1.48; 0.67) | -0.33 (-1.27; 0.62) | -0.33 (-1.21; 0.55) | -0.31 (-1.25; 0.62) | -0.34 (-0.98; 0.30) | -0.32 (-1.30; 0.66) | -0.31 (-1.27; 0.65) | -0.34 (-1.46; 0.78) | -0.29 (-1.24; 0.66) | -0.25 (-1.19; 0.69) | -0.23 (-1.17; 0.70) | -0.19 (-1.14; 0.76) | -0.08 (-1.13; 0.97) | **QIG** | NA | NA | NA | NA |
| -0.52 (-1.64; 0.61) | -0.45 (-1.37; 0.47) | -0.37 (-1.16; 0.42) | -0.37 (-1.24; 0.49) | -0.36 (-1.14; 0.43) | -0.39 (-1.40; 0.62) | -0.36 (-1.20; 0.47) | -0.36 (-1.17; 0.45) | -0.38 (-1.37; 0.60) | -0.33 (-1.26; 0.59) | -0.30 (-1.11; 0.52) | -0.28 (-1.06; 0.50) | -0.24 (-1.04; 0.56) | -0.13 (-1.06; 0.81) | -0.05 (-1.24; 1.15) | **DAN** | -0.01 (-0.76; 0.74) | NA | NA |
| -0.53 (-1.37; 0.32) | -0.46 (-1.00; 0.07) | **-0.38 (-0.63; -0.13)** | -0.38 (-0.82; 0.05) | **-0.37 (-0.61; -0.13)** | -0.40 (-1.08; 0.28) | **-0.37 (-0.74; -0.01)** | **-0.37 (-0.69; -0.05)** | -0.39 (-1.03; 0.24) | -0.34 (-0.89; 0.21) | -0.31 (-0.62; 0.01) | **-0.29 (-0.50; -0.08)** | -0.25 (-0.53; 0.03) | -0.14 (-0.69; 0.42) | -0.06 (-0.99; 0.88) | -0.01 (-0.76; 0.74) | **CON** | -0.27 (-1.25; 0.70) | NA |
| -0.80 (-2.09; 0.49) | -0.74 (-1.85; 0.38) | -0.66 (-1.67; 0.35) | -0.66 (-1.73; 0.41) | -0.64 (-1.65; 0.36) | -0.67 (-1.86; 0.52) | -0.65 (-1.69; 0.40) | -0.64 (-1.67; 0.38) | -0.67 (-1.84; 0.50) | -0.62 (-1.74; 0.50) | -0.58 (-1.61; 0.45) | -0.56 (-1.57; 0.44) | -0.52 (-1.54; 0.49) | -0.41 (-1.54; 0.71) | -0.33 (-1.68; 1.02) | -0.29 (-1.52; 0.95) | -0.27 (-1.25; 0.70) | **MD** | NA |
| -0.77 (-1.69; 0.16) | **-0.70 (-1.38; -0.02)** | **-0.62 (-1.09; -0.15)** | **-0.62 (-1.19; -0.06)** | **-0.61 (-1.06; -0.16)** | -0.64 (-1.37; 0.10) | **-0.61 (-1.09; -0.14)** | **-0.61 (-1.09; -0.12)** | -0.63 (-1.39; 0.12) | **-0.58 (-1.12; -0.05)** | **-0.55 (-0.92; -0.17)** | **-0.53 (-0.97; -0.09)** | **-0.49 (-0.94; -0.04)** | -0.38 (-0.85; 0.10) | -0.30 (-1.27; 0.68) | -0.25 (-1.11; 0.61) | -0.24 (-0.66; 0.19) | 0.04 (-1.03; 1.10) | **STR** |

All results are presented in the form of SMD (95% CrI). Physical activity types are ranked according to the surface under the curve cumulative for concern of falling starting with the best from left to right. The results of the network meta-analysis are showed in the lower left part, and results from pairwise comparisons in the upper right half (if available). Cells shown in bold indicate significant results. *NA* not available, *SMD* standardized Mean Difference, *CrI* Credible Interval, *AE* Aerobic Exercise, *AQE* Aquatic Exercise, *BGT* Balance and Gait Training, *BWS* Body Weight Support Treadmill Training, *CON* Control group, *CPP* Classic Physiotherapy Program, *DAN* Dance, *DT* Dual Task Balance and Gait Training, *ECA* Balance and Gait Training with External Cue or Attention, *ICA* Balance and Gait Training with Internal Cue or Attention, *MC* Multicomponent Exercise Program, *MD* Multidisciplinary Exercise Program, *QIG* Qigong*, RS* Robotic Assisted Gait Training, *RT* Resistance Training, *STR* Stretch, *TC* Tai Chi, *TT* Treadmill Training, *VR* Virtual Reality, *YOG* Yoga.

# Appendix 11: Evaluation of heterogeneity and inconsistency

## 11.1 Quantifying heterogeneity

We use the tau square (τ^2^) test and p-value to qualitatively analyze the statistical heterogeneity between the studies. The larger the τ^2^ and the smaller the p-value, the greater the possibility of heterogeneity; on the contrary, the smaller the existence heterogeneity. In addition, I^2^ is a parameter for quantitative analysis of the heterogeneity between the results of each study. It’ s value is distributed from 0-100%. When I^2^ is less than 25%, it means that the heterogeneity is low; 25%-50% means that the heterogeneity is moderate; I^2^ > 75% means high heterogeneity. In summary, when I^2^ > 50%, it means that there is substantial heterogeneity.

| Primary outcomes | τ^2^ | Q | df | P | I^2^ | Heterogeneity assessment |
| --- | --- | --- | --- | --- | --- | --- |
| Motor symptoms | 0.1041 | 367.96 | 179 | <0.0001 | 51.4% | moderate to high |
| Balance | 0.0552 | 167.53 | 108 | 0.0002 | 35.5% | moderate |
| Gait velocity | 0.0273 | 149.37 | 118 | 0.0270 | 21.0% | low |
| Freezing of gait | 0.0219 | 26.12 | 22 | 0.2465 | 15.8% | low |
| Depression | 0.1336 | 85.31 | 35 | <0.0001 | 59% | moderate to high |
| Anxiety | 0 | 6.77 | 7 | 0.4527 | 0% | low |
| Sleep quality | 0.5446 | 15.33 | 4 | 0.004 | 73.9% | moderate to high |
| Cognition | 0.2597 | 168.82 | 49 | <0.0001 | 71% | moderate to high |
| Walking distance | 0.1792 | 141.24 | 56 | <0.0001 | 60.4% | moderate to high |
| Muscle strength | 0 | 21.86 | 25 | 0.6440 | 0% | low |
| Concern of falling | 0.0440 | 75.88 | 51 | 0.0135 | 32.8% | moderate |

## 11.2 Evaluation of inconsistency

**Table 11.2.1 Summary of the global inconsistency and SIDE splitting results**

| Outcomes | Number of studies | SIDE splitting | | the Design-by-Treatment test | | | |
| --- | --- | --- | --- | --- | --- | --- | --- |
|  |  | Number of inconsistent comparisons out of total | Percentage of inconsistent comparisons out of total | Q | df | τ^2^ | p-value |
| Motor symptoms | 141 | 3 | 2.1% | 79.69 | 73 | 0.1011 | 0.2769 |
| Balance | 113 | 2 | 1.8% | 59.50 | 54 | 0.0429 | 0.2824 |
| Gait velocity | 106 | 4 | 3.8% | 68.66 | 65 | 0.0230 | 0.3544 |
| Freezing of gait | 35 | 0 | 0 | 16.92 | 12 | 0 | 0.1526 |
| Depression | 33 | 1 | 3.0% | 57.34 | 16 | 0.1252 | <0.0001 |
| Anxiety | 13 | 0 | 0 | 5.33 | 4 | 0 | 0.2535 |
| Sleep quality | 10 | 0 | 0 | 0.5 | 1 | 0.6707 | 0.4784 |
| Cognition | 50 | 0 | 0 | 8.39 | 25 | 0.4603 | 0.9986 |
| Walking distance | 55 | 5 | 9.1% | 32.40 | 31 | 0.1698 | 0.3976 |
| Muscle strength | 25 | 2 | 8.0% | 10.10 | 10 | 0 | 0.4319 |
| Concern of falling | 48 | 1 | 2.1% | 25.58 | 23 | 0.0447 | 0.3213 |

**Table 11.2.2 Details of SIDE splitting results (Motor symptoms)**

| **Comparison** | **k** | **prop** | **NMA** | | **Direct** | | **Indir** | | **Diff** | | **z** | **p** |
| --- | --- | --- | --- | --- | --- | --- | --- | --- | --- | --- | --- | --- |
|  |  |  | **TE** | **seTE** | **TE** | **seTE** | **TE** | **seTE** | **TE** | **seTE** |  |  |
| AE vs AQE | 0 | 0 | 0.04324368 | 0.1893493 | NA | NA | 0.04324368 | 0.1893493 | NA | NA | NA | NA |
| AE vs BGT | 5 | 0.43042448 | -0.024492 | 0.13439526 | -0.2204778 | 0.20484976 | 0.12361314 | 0.17807718 | -0.344091 | 0.27143122 | -1.2676913 | 0.20490822 |
| AE vs BGT_ECA | 3 | 0.27450364 | 0.25993855 | 0.13813307 | 0.56983496 | 0.26364747 | 0.14268408 | 0.16217356 | 0.42715088 | 0.30953231 | 1.37998801 | 0.16759034 |
| AE vs BGT_ICA | 0 | 0 | 0.15009352 | 0.2409311 | NA | NA | 0.15009352 | 0.2409311 | NA | NA | NA | NA |
| AE vs BWS_TT | 0 | 0 | 0.61495494 | 0.22227428 | NA | NA | 0.61495494 | 0.22227428 | NA | NA | NA | NA |
| AE vs CON | 4 | 0.24938385 | -0.2990374 | 0.12026409 | -0.4348811 | 0.24082514 | -0.2539048 | 0.138812 | -0.1809763 | 0.27796676 | -0.6510717 | 0.51500019 |
| AE vs CPP | 0 | 0 | -0.1933066 | 0.14971099 | NA | NA | -0.1933066 | 0.14971099 | NA | NA | NA | NA |
| AE vs Dance | 0 | 0 | 0.27417242 | 0.23612551 | NA | NA | 0.27417242 | 0.23612551 | NA | NA | NA | NA |
| AE vs DT_BGT | 0 | 0 | 0.03040808 | 0.17318923 | NA | NA | 0.03040808 | 0.17318923 | NA | NA | NA | NA |
| AE vs Mul_C | 3 | 0.3005259 | 0.29339473 | 0.12872433 | 0.04484518 | 0.23481167 | 0.40018293 | 0.15391283 | -0.3553377 | 0.28075911 | -1.2656321 | 0.20564482 |
| AE vs Mul_D | 0 | 0 | 0.24953827 | 0.17620251 | NA | NA | 0.24953827 | 0.17620251 | NA | NA | NA | NA |
| AE vs NW | 0 | 0 | 0.20975997 | 0.30484414 | NA | NA | 0.20975997 | 0.30484414 | NA | NA | NA | NA |
| AE vs Pialtes | 0 | 0 | 0.62187386 | 0.55417854 | NA | NA | 0.62187386 | 0.55417854 | NA | NA | NA | NA |
| AE vs PT | 0 | 0 | 1.1589483 | 0.38453013 | NA | NA | 1.1589483 | 0.38453013 | NA | NA | NA | NA |
| AE vs Qigong | 1 | 0.15092128 | 0.27751727 | 0.19782295 | 0.24685129 | 0.50921529 | 0.28296806 | 0.21468549 | -0.0361168 | 0.5526211 | -0.0653554 | 0.94789103 |
| AE vs RA_GT | 1 | 0.16533619 | 0.2042376 | 0.18391617 | 0.2347916 | 0.45230974 | 0.19818524 | 0.20130943 | 0.03660636 | 0.49508544 | 0.07393948 | 0.94105854 |
| AE vs RT | 0 | 0 | 0.3081256 | 0.13772198 | NA | NA | 0.3081256 | 0.13772198 | NA | NA | NA | NA |
| AE vs Stretch | 5 | 0.5970853 | -0.3378214 | 0.14140246 | -0.2312632 | 0.18299481 | -0.4957316 | 0.22276676 | 0.26446838 | 0.28829174 | 0.91736369 | 0.35895209 |
| AE vs Tango | 0 | 0 | 0.29603715 | 0.20942459 | NA | NA | 0.29603715 | 0.20942459 | NA | NA | NA | NA |
| AE vs TC | 0 | 0 | 0.29319398 | 0.16714528 | NA | NA | 0.29319398 | 0.16714528 | NA | NA | NA | NA |
| AE vs TT | 1 | 0.08734651 | 0.08356887 | 0.14584602 | -0.0290456 | 0.49348255 | 0.09434676 | 0.15266575 | -0.1233924 | 0.5165577 | -0.2388743 | 0.81120304 |
| AE vs VR | 1 | 0.20130636 | 0.11062638 | 0.19292779 | 0.33550474 | 0.42999761 | 0.05394702 | 0.21587616 | 0.28155772 | 0.48114495 | 0.58518273 | 0.55842486 |
| AE vs WBV | 0 | 0 | -0.3683383 | 0.38245943 | NA | NA | -0.3683383 | 0.38245943 | NA | NA | NA | NA |
| AE vs Yoga | 0 | 0 | 0.81715342 | 0.25000823 | NA | NA | 0.81715342 | 0.25000823 | NA | NA | NA | NA |
| AQE vs BGT | 0 | 0 | -0.0677357 | 0.18137073 | NA | NA | -0.0677357 | 0.18137073 | NA | NA | NA | NA |
| AQE vs BGT_ECA | 0 | 0 | 0.21669487 | 0.18290274 | NA | NA | 0.21669487 | 0.18290274 | NA | NA | NA | NA |
| AQE vs BGT_ICA | 0 | 0 | 0.10684983 | 0.2652056 | NA | NA | 0.10684983 | 0.2652056 | NA | NA | NA | NA |
| AQE vs BWS_TT | 0 | 0 | 0.57171125 | 0.2493433 | NA | NA | 0.57171125 | 0.2493433 | NA | NA | NA | NA |
| AQE vs CON | 5 | 0.4470124 | -0.3422811 | 0.1543644 | -0.1122308 | 0.23088056 | -0.5282442 | 0.20758189 | 0.41601348 | 0.31047718 | 1.33991646 | 0.18027251 |
| AQE vs CPP | 3 | 0.3053248 | -0.2365503 | 0.16708628 | -0.3726936 | 0.30238466 | -0.1767123 | 0.20047024 | -0.1959813 | 0.36280132 | -0.5401891 | 0.58906664 |
| AQE vs Dance | 0 | 0 | 0.23092873 | 0.25558314 | NA | NA | 0.23092873 | 0.25558314 | NA | NA | NA | NA |
| AQE vs DT_BGT | 1 | 0.17718183 | -0.0128356 | 0.19820989 | 0.00859065 | 0.47088594 | -0.0174494 | 0.21851104 | 0.02604009 | 0.51911525 | 0.05016244 | 0.95999294 |
| AQE vs Mul_C | 2 | 0.15848264 | 0.25015104 | 0.16408483 | -0.02944 | 0.41217115 | 0.30280631 | 0.17886975 | -0.3322463 | 0.44930996 | -0.7394589 | 0.45962838 |
| AQE vs Mul_D | 1 | 0.19728757 | 0.20629459 | 0.18903569 | -0.0251147 | 0.42559248 | 0.26316947 | 0.21099094 | -0.2882841 | 0.47502225 | -0.6068855 | 0.54392687 |
| AQE vs NW | 0 | 0 | 0.16651629 | 0.32161027 | NA | NA | 0.16651629 | 0.32161027 | NA | NA | NA | NA |
| AQE vs Pialtes | 0 | 0 | 0.57863017 | 0.56344275 | NA | NA | 0.57863017 | 0.56344275 | NA | NA | NA | NA |
| AQE vs PT | 0 | 0 | 1.11570461 | 0.3968488 | NA | NA | 1.11570461 | 0.3968488 | NA | NA | NA | NA |
| AQE vs Qigong | 0 | 0 | 0.23427359 | 0.22628519 | NA | NA | 0.23427359 | 0.22628519 | NA | NA | NA | NA |
| AQE vs RA_GT | 0 | 0 | 0.16099391 | 0.2219669 | NA | NA | 0.16099391 | 0.2219669 | NA | NA | NA | NA |
| AQE vs RT | 0 | 0 | 0.26488192 | 0.1735408 | NA | NA | 0.26488192 | 0.1735408 | NA | NA | NA | NA |
| AQE vs Stretch | 0 | 0 | -0.3810651 | 0.20573627 | NA | NA | -0.3810651 | 0.20573627 | NA | NA | NA | NA |
| AQE vs Tango | 0 | 0 | 0.25279346 | 0.23148351 | NA | NA | 0.25279346 | 0.23148351 | NA | NA | NA | NA |
| AQE vs TC | 0 | 0 | 0.24995029 | 0.20669029 | NA | NA | 0.24995029 | 0.20669029 | NA | NA | NA | NA |
| AQE vs TT | 0 | 0 | 0.04032518 | 0.18528694 | NA | NA | 0.04032518 | 0.18528694 | NA | NA | NA | NA |
| AQE vs VR | 0 | 0 | 0.0673827 | 0.22477127 | NA | NA | 0.0673827 | 0.22477127 | NA | NA | NA | NA |
| AQE vs WBV | 0 | 0 | -0.4115819 | 0.39433212 | NA | NA | -0.4115819 | 0.39433212 | NA | NA | NA | NA |
| AQE vs Yoga | 0 | 0 | 0.77390973 | 0.26928918 | NA | NA | 0.77390973 | 0.26928918 | NA | NA | NA | NA |
| BGT vs BGT_ECA | 5 | 0.38210635 | 0.2844306 | 0.12616037 | 0.07220315 | 0.20409429 | 0.41567236 | 0.1604967 | -0.3434692 | 0.25964142 | -1.3228598 | 0.185882 |
| BGT vs BGT_ICA | 0 | 0 | 0.17458556 | 0.23502879 | NA | NA | 0.17458556 | 0.23502879 | NA | NA | NA | NA |
| BGT vs BWS_TT | 1 | 0.14500696 | 0.63944698 | 0.21246176 | 0.39803672 | 0.55793853 | 0.6803902 | 0.22977326 | -0.2823535 | 0.60339966 | -0.4679378 | 0.6398291 |
| BGT vs CON | 4 | 0.28037781 | -0.2745453 | 0.11023292 | 0.00164422 | 0.20818024 | -0.3821538 | 0.12994484 | 0.38379799 | 0.24540716 | 1.56392336 | 0.11783557 |
| BGT vs CPP | 3 | 0.31880926 | -0.1688145 | 0.13486448 | 0.0133104 | 0.23885379 | -0.2540522 | 0.1634042 | 0.26736261 | 0.28939949 | 0.92385307 | 0.35556282 |
| BGT vs Dance | 0 | 0 | 0.29866446 | 0.23064642 | NA | NA | 0.29866446 | 0.23064642 | NA | NA | NA | NA |
| BGT vs DT_BGT | 2 | 0.1755541 | 0.05490013 | 0.16369986 | -0.1226782 | 0.39069947 | 0.09271293 | 0.18028817 | -0.2153912 | 0.43029049 | -0.5005715 | 0.61667271 |
| BGT vs Mul_C | 4 | 0.3079779 | 0.31788678 | 0.118991 | 0.13408434 | 0.21441459 | 0.39968631 | 0.14303889 | -0.265602 | 0.25774743 | -1.0304738 | 0.30278765 |
| BGT vs Mul_D | 0 | 0 | 0.27403032 | 0.16738598 | NA | NA | 0.27403032 | 0.16738598 | NA | NA | NA | NA |
| BGT vs NW | 0 | 0 | 0.23425202 | 0.30103225 | NA | NA | 0.23425202 | 0.30103225 | NA | NA | NA | NA |
| BGT vs Pialtes | 0 | 0 | 0.64636591 | 0.55199888 | NA | NA | 0.64636591 | 0.55199888 | NA | NA | NA | NA |
| BGT vs PT | 0 | 0 | 1.18344035 | 0.3815108 | NA | NA | 1.18344035 | 0.3815108 | NA | NA | NA | NA |
| BGT vs Qigong | 0 | 0 | 0.30200932 | 0.19784498 | NA | NA | 0.30200932 | 0.19784498 | NA | NA | NA | NA |
| BGT vs RA_GT | 1 | 0.15434688 | 0.22872964 | 0.18086703 | 0.41549503 | 0.46037367 | 0.19464161 | 0.19668139 | 0.22085342 | 0.50062709 | 0.44115356 | 0.65910183 |
| BGT vs RT | 1 | 0.07250128 | 0.33261765 | 0.12903444 | 0.06355557 | 0.47921763 | 0.35364985 | 0.13398278 | -0.2900943 | 0.49759514 | -0.5829926 | 0.55989828 |
| BGT vs Stretch | 0 | 0 | -0.3133293 | 0.16419398 | NA | NA | -0.3133293 | 0.16419398 | NA | NA | NA | NA |
| BGT vs Tango | 0 | 0 | 0.32052919 | 0.20372536 | NA | NA | 0.32052919 | 0.20372536 | NA | NA | NA | NA |
| BGT vs TC | 0 | 0 | 0.31768602 | 0.17138813 | NA | NA | 0.31768602 | 0.17138813 | NA | NA | NA | NA |
| BGT vs TT | 1 | 0.06810386 | 0.10806091 | 0.13811405 | 0.31596324 | 0.52923921 | 0.09286721 | 0.14307183 | 0.22309603 | 0.54823689 | 0.40693363 | 0.68405673 |
| BGT vs VR | 2 | 0.22942953 | 0.13511843 | 0.18367356 | -0.1291692 | 0.38346169 | 0.21380739 | 0.20923795 | -0.3429766 | 0.43683337 | -0.7851428 | 0.43236984 |
| BGT vs WBV | 0 | 0 | -0.3438462 | 0.37820318 | NA | NA | -0.3438462 | 0.37820318 | NA | NA | NA | NA |
| BGT vs Yoga | 0 | 0 | 0.84164547 | 0.24533657 | NA | NA | 0.84164547 | 0.24533657 | NA | NA | NA | NA |
| BGT_ECA vs BGT_ICA | 4 | 0.82182599 | -0.109845 | 0.2137546 | -0.2483462 | 0.23579008 | 0.5289901 | 0.50639952 | -0.7773363 | 0.55860312 | -1.3915716 | 0.16405215 |
| BGT_ECA vs BWS_TT | 2 | 0.40866055 | 0.35501639 | 0.20689059 | 0.62584617 | 0.3236379 | 0.16785241 | 0.26904338 | 0.45799376 | 0.42086319 | 1.08822479 | 0.27649588 |
| BGT_ECA vs CON | 10 | 0.5333287 | -0.5589759 | 0.10733651 | -0.4542114 | 0.14697721 | -0.6787046 | 0.15712379 | 0.22449318 | 0.21515154 | 1.04341889 | 0.29675433 |
| BGT_ECA vs CPP | 0 | 0 | -0.4532451 | 0.14252976 | NA | NA | -0.4532451 | 0.14252976 | NA | NA | NA | NA |
| BGT_ECA vs Dance | 0 | 0 | 0.01423387 | 0.23004468 | NA | NA | 0.01423387 | 0.23004468 | NA | NA | NA | NA |
| BGT_ECA vs DT_BGT | 0 | 0 | -0.2295305 | 0.17078302 | NA | NA | -0.2295305 | 0.17078302 | NA | NA | NA | NA |
| BGT_ECA vs Mul_C | 1 | 0.06036951 | 0.03345618 | 0.12238023 | -0.2308017 | 0.49808384 | 0.05043425 | 0.12625039 | -0.2812359 | 0.51383526 | -0.547327 | 0.58415411 |
| BGT_ECA vs Mul_D | 1 | 0.09604814 | -0.0104003 | 0.16658478 | -0.3228595 | 0.53751533 | 0.02279963 | 0.17521152 | -0.3456591 | 0.56535105 | -0.6114061 | 0.54093076 |
| BGT_ECA vs NW | 0 | 0 | -0.0501786 | 0.30049905 | NA | NA | -0.0501786 | 0.30049905 | NA | NA | NA | NA |
| BGT_ECA vs Pialtes | 0 | 0 | 0.36193531 | 0.55273938 | NA | NA | 0.36193531 | 0.55273938 | NA | NA | NA | NA |
| BGT_ECA vs PT | 0 | 0 | 0.89900975 | 0.38074836 | NA | NA | 0.89900975 | 0.38074836 | NA | NA | NA | NA |
| BGT_ECA vs Qigong | 0 | 0 | 0.01757872 | 0.19918684 | NA | NA | 0.01757872 | 0.19918684 | NA | NA | NA | NA |
| BGT_ECA vs RA_GT | 0 | 0 | -0.055701 | 0.18636661 | NA | NA | -0.055701 | 0.18636661 | NA | NA | NA | NA |
| BGT_ECA vs RT | 2 | 0.18776449 | 0.04818705 | 0.12788709 | -0.1191705 | 0.2951345 | 0.08687509 | 0.14190109 | -0.2060456 | 0.32747563 | -0.6291935 | 0.52922236 |
| BGT_ECA vs Stretch | 0 | 0 | -0.5977599 | 0.16667262 | NA | NA | -0.5977599 | 0.16667262 | NA | NA | NA | NA |
| BGT_ECA vs Tango | 0 | 0 | 0.0360986 | 0.20247222 | NA | NA | 0.0360986 | 0.20247222 | NA | NA | NA | NA |
| BGT_ECA vs TC | 0 | 0 | 0.03325542 | 0.17194701 | NA | NA | 0.03325542 | 0.17194701 | NA | NA | NA | NA |
| BGT_ECA vs TT | 3 | 0.20848123 | -0.1763697 | 0.13487247 | -0.1674323 | 0.29538594 | -0.1787237 | 0.15159773 | 0.01129142 | 0.33201615 | 0.03400866 | 0.97287024 |
| BGT_ECA vs VR | 0 | 0 | -0.1493122 | 0.19201457 | NA | NA | -0.1493122 | 0.19201457 | NA | NA | NA | NA |
| BGT_ECA vs WBV | 0 | 0 | -0.6282768 | 0.38065988 | NA | NA | -0.6282768 | 0.38065988 | NA | NA | NA | NA |
| BGT_ECA vs Yoga | 0 | 0 | 0.55721487 | 0.24429819 | NA | NA | 0.55721487 | 0.24429819 | NA | NA | NA | NA |
| BGT_ICA vs BWS_TT | 0 | 0 | 0.46486142 | 0.28737678 | NA | NA | 0.46486142 | 0.28737678 | NA | NA | NA | NA |
| BGT_ICA vs CON | 4 | 0.71038409 | -0.4491309 | 0.21820113 | -0.6436485 | 0.25888708 | 0.02799147 | 0.40545792 | -0.67164 | 0.48105992 | -1.396167 | 0.16266421 |
| BGT_ICA vs CPP | 0 | 0 | -0.3434001 | 0.2400563 | NA | NA | -0.3434001 | 0.2400563 | NA | NA | NA | NA |
| BGT_ICA vs Dance | 0 | 0 | 0.1240789 | 0.29951839 | NA | NA | 0.1240789 | 0.29951839 | NA | NA | NA | NA |
| BGT_ICA vs DT_BGT | 0 | 0 | -0.1196854 | 0.25830583 | NA | NA | -0.1196854 | 0.25830583 | NA | NA | NA | NA |
| BGT_ICA vs Mul_C | 0 | 0 | 0.14330121 | 0.22915009 | NA | NA | 0.14330121 | 0.22915009 | NA | NA | NA | NA |
| BGT_ICA vs Mul_D | 0 | 0 | 0.09944476 | 0.2553701 | NA | NA | 0.09944476 | 0.2553701 | NA | NA | NA | NA |
| BGT_ICA vs NW | 0 | 0 | 0.05966646 | 0.35689036 | NA | NA | 0.05966646 | 0.35689036 | NA | NA | NA | NA |
| BGT_ICA vs Pialtes | 0 | 0 | 0.47178034 | 0.58570783 | NA | NA | 0.47178034 | 0.58570783 | NA | NA | NA | NA |
| BGT_ICA vs PT | 0 | 0 | 1.00885478 | 0.42579762 | NA | NA | 1.00885478 | 0.42579762 | NA | NA | NA | NA |
| BGT_ICA vs Qigong | 0 | 0 | 0.12742376 | 0.27686184 | NA | NA | 0.12742376 | 0.27686184 | NA | NA | NA | NA |
| BGT_ICA vs RA_GT | 0 | 0 | 0.05414408 | 0.27020603 | NA | NA | 0.05414408 | 0.27020603 | NA | NA | NA | NA |
| BGT_ICA vs RT | 0 | 0 | 0.15803208 | 0.23262389 | NA | NA | 0.15803208 | 0.23262389 | NA | NA | NA | NA |
| BGT_ICA vs Stretch | 0 | 0 | -0.4879149 | 0.25665859 | NA | NA | -0.4879149 | 0.25665859 | NA | NA | NA | NA |
| BGT_ICA vs Tango | 0 | 0 | 0.14594363 | 0.27840543 | NA | NA | 0.14594363 | 0.27840543 | NA | NA | NA | NA |
| BGT_ICA vs TC | 0 | 0 | 0.14310046 | 0.25838956 | NA | NA | 0.14310046 | 0.25838956 | NA | NA | NA | NA |
| BGT_ICA vs TT | 0 | 0 | -0.0665247 | 0.23839805 | NA | NA | -0.0665247 | 0.23839805 | NA | NA | NA | NA |
| BGT_ICA vs VR | 0 | 0 | -0.0394671 | 0.27397094 | NA | NA | -0.0394671 | 0.27397094 | NA | NA | NA | NA |
| BGT_ICA vs WBV | 0 | 0 | -0.5184318 | 0.42734571 | NA | NA | -0.5184318 | 0.42734571 | NA | NA | NA | NA |
| BGT_ICA vs Yoga | 0 | 0 | 0.6670599 | 0.31044765 | NA | NA | 0.6670599 | 0.31044765 | NA | NA | NA | NA |
| BWS_TT vs CON | 2 | 0.37297559 | -0.9139923 | 0.2003539 | -0.9010344 | 0.32806326 | -0.9217001 | 0.25302042 | 0.0206657 | 0.41430042 | 0.04988094 | 0.96021726 |
| BWS_TT vs CPP | 0 | 0 | -0.8082615 | 0.22173137 | NA | NA | -0.8082615 | 0.22173137 | NA | NA | NA | NA |
| BWS_TT vs Dance | 0 | 0 | -0.3407825 | 0.28339663 | NA | NA | -0.3407825 | 0.28339663 | NA | NA | NA | NA |
| BWS_TT vs DT_BGT | 0 | 0 | -0.5845469 | 0.23798479 | NA | NA | -0.5845469 | 0.23798479 | NA | NA | NA | NA |
| BWS_TT vs Mul_C | 0 | 0 | -0.3215602 | 0.20973126 | NA | NA | -0.3215602 | 0.20973126 | NA | NA | NA | NA |
| BWS_TT vs Mul_D | 0 | 0 | -0.3654167 | 0.23927257 | NA | NA | -0.3654167 | 0.23927257 | NA | NA | NA | NA |
| BWS_TT vs NW | 0 | 0 | -0.405195 | 0.34247589 | NA | NA | -0.405195 | 0.34247589 | NA | NA | NA | NA |
| BWS_TT vs Pialtes | 0 | 0 | 0.00691892 | 0.57838664 | NA | NA | 0.00691892 | 0.57838664 | NA | NA | NA | NA |
| BWS_TT vs PT | 0 | 0 | 0.54399336 | 0.41674542 | NA | NA | 0.54399336 | 0.41674542 | NA | NA | NA | NA |
| BWS_TT vs Qigong | 0 | 0 | -0.3374377 | 0.26192074 | NA | NA | -0.3374377 | 0.26192074 | NA | NA | NA | NA |
| BWS_TT vs RA_GT | 0 | 0 | -0.4107173 | 0.24607412 | NA | NA | -0.4107173 | 0.24607412 | NA | NA | NA | NA |
| BWS_TT vs RT | 0 | 0 | -0.3068293 | 0.21347214 | NA | NA | -0.3068293 | 0.21347214 | NA | NA | NA | NA |
| BWS_TT vs Stretch | 0 | 0 | -0.9527763 | 0.23844605 | NA | NA | -0.9527763 | 0.23844605 | NA | NA | NA | NA |
| BWS_TT vs Tango | 0 | 0 | -0.3189178 | 0.26366035 | NA | NA | -0.3189178 | 0.26366035 | NA | NA | NA | NA |
| BWS_TT vs TC | 0 | 0 | -0.321761 | 0.24181236 | NA | NA | -0.321761 | 0.24181236 | NA | NA | NA | NA |
| BWS_TT vs TT | 4 | 0.48739025 | -0.5313861 | 0.19897096 | -0.4671737 | 0.28500421 | -0.5924393 | 0.27790494 | 0.1252656 | 0.39806853 | 0.31468349 | 0.75300198 |
| BWS_TT vs VR | 0 | 0 | -0.5043286 | 0.25180959 | NA | NA | -0.5043286 | 0.25180959 | NA | NA | NA | NA |
| BWS_TT vs WBV | 0 | 0 | -0.9832932 | 0.41551979 | NA | NA | -0.9832932 | 0.41551979 | NA | NA | NA | NA |
| BWS_TT vs Yoga | 0 | 0 | 0.20219848 | 0.2975198 | NA | NA | 0.20219848 | 0.2975198 | NA | NA | NA | NA |
| CON vs CPP | 6 | 0.33093726 | -0.1057308 | 0.1082858 | -0.3943773 | 0.18823426 | 0.0370419 | 0.13238479 | -0.4314192 | 0.23012577 | -1.8747106 | 0.06083255 |
| CON vs Dance | 3 | 0.48605142 | -0.5732098 | 0.20755667 | -0.4404201 | 0.29771148 | -0.6987917 | 0.28951886 | 0.25837163 | 0.41527496 | 0.62217001 | 0.53383009 |
| CON vs DT_BGT | 3 | 0.38703226 | -0.3294455 | 0.14546682 | -0.31875 | 0.23382469 | -0.3361986 | 0.18579975 | 0.01744862 | 0.29865621 | 0.05842375 | 0.9534111 |
| CON vs Mul_C | 14 | 0.42239257 | -0.5924321 | 0.0831706 | -1.0050842 | 0.12797104 | -0.290668 | 0.1094343 | -0.7144162 | 0.16838187 | -4.2428335 | 2.21E-05 |
| CON vs Mul_D | 3 | 0.37701276 | -0.5485757 | 0.13972917 | -0.2015288 | 0.22756688 | -0.7585977 | 0.17703026 | 0.55706888 | 0.28831649 | 1.93214365 | 0.05334178 |
| CON vs NW | 2 | 0.64409002 | -0.5087973 | 0.28483637 | -0.203361 | 0.3549132 | -1.0615452 | 0.4774472 | 0.85818415 | 0.59491109 | 1.44254185 | 0.14914957 |
| CON vs Pialtes | 0 | 0 | -0.9209112 | 0.54540008 | NA | NA | -0.9209112 | 0.54540008 | NA | NA | NA | NA |
| CON vs PT | 2 | 0.77112703 | -1.4579857 | 0.36610527 | -1.8752324 | 0.41691068 | -0.0521831 | 0.76525949 | -1.8230493 | 0.8714566 | -2.0919564 | 0.03644241 |
| CON vs Qigong | 3 | 0.43729822 | -0.5765547 | 0.17401704 | -0.2238304 | 0.26314972 | -0.8506709 | 0.23198111 | 0.62684055 | 0.35080338 | 1.78687146 | 0.07395827 |
| CON vs RA_GT | 1 | 0.07040235 | -0.503275 | 0.1687107 | -1.0637256 | 0.63584174 | -0.4608297 | 0.1749827 | -0.6028959 | 0.65947985 | -0.9141992 | 0.36061221 |
| CON vs RT | 8 | 0.3118411 | -0.607163 | 0.09400948 | -0.2680043 | 0.16834685 | -0.7608537 | 0.11332541 | 0.49284942 | 0.20293671 | 2.4285868 | 0.0151578 |
| CON vs Stretch | 0 | 0 | 0.038784 | 0.14514382 | NA | NA | 0.038784 | 0.14514382 | NA | NA | NA | NA |
| CON vs Tango | 5 | 0.68343072 | -0.5950745 | 0.17461809 | -0.5717331 | 0.21122338 | -0.6454655 | 0.31035222 | 0.07373237 | 0.37541153 | 0.19640411 | 0.84429388 |
| CON vs TC | 5 | 0.43434975 | -0.5922314 | 0.14389533 | -0.4886781 | 0.21833679 | -0.6717475 | 0.19132544 | 0.18306946 | 0.29030394 | 0.63061307 | 0.52829355 |
| CON vs TT | 4 | 0.25660788 | -0.3826062 | 0.11311786 | -0.2764395 | 0.22330384 | -0.4192534 | 0.13119648 | 0.14281394 | 0.25899251 | 0.55142112 | 0.58134502 |
| CON vs VR | 1 | 0.15578867 | -0.4096638 | 0.17432279 | -1.1355545 | 0.44165813 | -0.2757097 | 0.18972675 | -0.8598448 | 0.48068508 | -1.7887903 | 0.07364859 |
| CON vs WBV | 0 | 0 | 0.06930089 | 0.37053659 | NA | NA | 0.06930089 | 0.37053659 | NA | NA | NA | NA |
| CON vs Yoga | 4 | 0.61036424 | -1.1161908 | 0.22274162 | -1.2649043 | 0.28510631 | -0.8832311 | 0.35683872 | -0.3816732 | 0.45674882 | -0.8356304 | 0.40336285 |
| CPP vs Dance | 1 | 0.16699464 | 0.467479 | 0.22541567 | 1.1363759 | 0.5516109 | 0.33338359 | 0.2469791 | 0.80299231 | 0.60437841 | 1.32862506 | 0.1839717 |
| CPP vs DT_BGT | 0 | 0 | 0.22371466 | 0.172325 | NA | NA | 0.22371466 | 0.172325 | NA | NA | NA | NA |
| CPP vs Mul_C | 5 | 0.30819808 | 0.48670131 | 0.11576326 | 0.53289651 | 0.20852388 | 0.46612133 | 0.13918098 | 0.06677518 | 0.25070611 | 0.26634842 | 0.78997089 |
| CPP vs Mul_D | 3 | 0.35590222 | 0.44284486 | 0.15103095 | 0.66395259 | 0.25316322 | 0.32066972 | 0.18818719 | 0.34328286 | 0.31544577 | 1.08824684 | 0.27648615 |
| CPP vs NW | 0 | 0 | 0.40306656 | 0.30127549 | NA | NA | 0.40306656 | 0.30127549 | NA | NA | NA | NA |
| CPP vs Pialtes | 0 | 0 | 0.81518044 | 0.5513121 | NA | NA | 0.81518044 | 0.5513121 | NA | NA | NA | NA |
| CPP vs PT | 0 | 0 | 1.35225488 | 0.38103871 | NA | NA | 1.35225488 | 0.38103871 | NA | NA | NA | NA |
| CPP vs Qigong | 2 | 0.36731563 | 0.47082386 | 0.18433813 | 0.68686824 | 0.30415536 | 0.34539563 | 0.23175101 | 0.34147261 | 0.38238595 | 0.89300513 | 0.37185442 |
| CPP vs RA_GT | 0 | 0 | 0.39754418 | 0.1919689 | NA | NA | 0.39754418 | 0.1919689 | NA | NA | NA | NA |
| CPP vs RT | 1 | 0.06766458 | 0.50143218 | 0.12912284 | 0.95777415 | 0.49638927 | 0.468313 | 0.13372635 | 0.48946115 | 0.51408661 | 0.95209861 | 0.34104698 |
| CPP vs Stretch | 0 | 0 | -0.1445148 | 0.17134814 | NA | NA | -0.1445148 | 0.17134814 | NA | NA | NA | NA |
| CPP vs Tango | 0 | 0 | 0.48934373 | 0.20206185 | NA | NA | 0.48934373 | 0.20206185 | NA | NA | NA | NA |
| CPP vs TC | 0 | 0 | 0.48650056 | 0.17173467 | NA | NA | 0.48650056 | 0.17173467 | NA | NA | NA | NA |
| CPP vs TT | 0 | 0 | 0.27687545 | 0.14626067 | NA | NA | 0.27687545 | 0.14626067 | NA | NA | NA | NA |
| CPP vs VR | 1 | 0.146469 | 0.30393297 | 0.19019514 | 0.03899414 | 0.49696587 | 0.34939743 | 0.20586844 | -0.3104033 | 0.53791904 | -0.5770446 | 0.56390931 |
| CPP vs WBV | 0 | 0 | -0.1750317 | 0.37885399 | NA | NA | -0.1750317 | 0.37885399 | NA | NA | NA | NA |
| CPP vs Yoga | 0 | 0 | 1.01046 | 0.24480049 | NA | NA | 1.01046 | 0.24480049 | NA | NA | NA | NA |
| Dance vs DT_BGT | 0 | 0 | -0.2437643 | 0.24982304 | NA | NA | -0.2437643 | 0.24982304 | NA | NA | NA | NA |
| Dance vs Mul_C | 0 | 0 | 0.01922231 | 0.21967102 | NA | NA | 0.01922231 | 0.21967102 | NA | NA | NA | NA |
| Dance vs Mul_D | 0 | 0 | -0.0246341 | 0.24650985 | NA | NA | -0.0246341 | 0.24650985 | NA | NA | NA | NA |
| Dance vs NW | 0 | 0 | -0.0644124 | 0.35002923 | NA | NA | -0.0644124 | 0.35002923 | NA | NA | NA | NA |
| Dance vs Pialtes | 0 | 0 | 0.34770144 | 0.58206465 | NA | NA | 0.34770144 | 0.58206465 | NA | NA | NA | NA |
| Dance vs PT | 0 | 0 | 0.88477588 | 0.4204746 | NA | NA | 0.88477588 | 0.4204746 | NA | NA | NA | NA |
| Dance vs Qigong | 0 | 0 | 0.00334486 | 0.26785624 | NA | NA | 0.00334486 | 0.26785624 | NA | NA | NA | NA |
| Dance vs RA_GT | 0 | 0 | -0.0699348 | 0.26170913 | NA | NA | -0.0699348 | 0.26170913 | NA | NA | NA | NA |
| Dance vs RT | 0 | 0 | 0.03395318 | 0.22308831 | NA | NA | 0.03395318 | 0.22308831 | NA | NA | NA | NA |
| Dance vs Stretch | 0 | 0 | -0.6119938 | 0.24977576 | NA | NA | -0.6119938 | 0.24977576 | NA | NA | NA | NA |
| Dance vs Tango | 2 | 0.34314761 | 0.02186473 | 0.24207226 | -0.009184 | 0.41324207 | 0.03808494 | 0.29868343 | -0.0472689 | 0.50988312 | -0.0927053 | 0.92613765 |
| Dance vs TC | 0 | 0 | 0.01902156 | 0.25024703 | NA | NA | 0.01902156 | 0.25024703 | NA | NA | NA | NA |
| Dance vs TT | 1 | 0.21344259 | -0.1906036 | 0.22511492 | 0.01879102 | 0.48726334 | -0.2474255 | 0.25382773 | 0.26621651 | 0.54941249 | 0.48454762 | 0.62799728 |
| Dance vs VR | 0 | 0 | -0.163546 | 0.26546189 | NA | NA | -0.163546 | 0.26546189 | NA | NA | NA | NA |
| Dance vs WBV | 0 | 0 | -0.6425107 | 0.42212667 | NA | NA | -0.6425107 | 0.42212667 | NA | NA | NA | NA |
| Dance vs Yoga | 0 | 0 | 0.542981 | 0.30317235 | NA | NA | 0.542981 | 0.30317235 | NA | NA | NA | NA |
| DT_BGT vs Mul_C | 0 | 0 | 0.26298665 | 0.15917894 | NA | NA | 0.26298665 | 0.15917894 | NA | NA | NA | NA |
| DT_BGT vs Mul_D | 0 | 0 | 0.21913019 | 0.19336429 | NA | NA | 0.21913019 | 0.19336429 | NA | NA | NA | NA |
| DT_BGT vs NW | 0 | 0 | 0.17935189 | 0.31606673 | NA | NA | 0.17935189 | 0.31606673 | NA | NA | NA | NA |
| DT_BGT vs Pialtes | 0 | 0 | 0.59146578 | 0.56203366 | NA | NA | 0.59146578 | 0.56203366 | NA | NA | NA | NA |
| DT_BGT vs PT | 0 | 0 | 1.12854022 | 0.39342887 | NA | NA | 1.12854022 | 0.39342887 | NA | NA | NA | NA |
| DT_BGT vs Qigong | 0 | 0 | 0.24710919 | 0.22166629 | NA | NA | 0.24710919 | 0.22166629 | NA | NA | NA | NA |
| DT_BGT vs RA_GT | 2 | 0.33113053 | 0.17382952 | 0.18829816 | 0.0088657 | 0.327225 | 0.2554965 | 0.23023715 | -0.2466308 | 0.40010666 | -0.6164126 | 0.53762222 |
| DT_BGT vs RT | 0 | 0 | 0.27771752 | 0.16495333 | NA | NA | 0.27771752 | 0.16495333 | NA | NA | NA | NA |
| DT_BGT vs Stretch | 1 | 0.19005886 | -0.3682295 | 0.18358075 | -0.2212126 | 0.4210979 | -0.4027281 | 0.20398602 | 0.1815155 | 0.46790355 | 0.38793357 | 0.69806519 |
| DT_BGT vs Tango | 0 | 0 | 0.26562907 | 0.22545925 | NA | NA | 0.26562907 | 0.22545925 | NA | NA | NA | NA |
| DT_BGT vs TC | 0 | 0 | 0.2627859 | 0.19478854 | NA | NA | 0.2627859 | 0.19478854 | NA | NA | NA | NA |
| DT_BGT vs TT | 1 | 0.1271372 | 0.05316079 | 0.16397765 | -0.3510257 | 0.45988406 | 0.11203274 | 0.17551397 | -0.4630585 | 0.49223825 | -0.9407202 | 0.34684827 |
| DT_BGT vs VR | 1 | 0.17855613 | 0.08021831 | 0.20274052 | 0.52039274 | 0.47979219 | -0.0154618 | 0.2236926 | 0.53585455 | 0.52937597 | 1.01223814 | 0.31142421 |
| DT_BGT vs WBV | 1 | 0.44846199 | -0.3987463 | 0.36976508 | -0.5669843 | 0.55215763 | -0.2619501 | 0.49789547 | -0.3050342 | 0.74349038 | -0.4102733 | 0.68160549 |
| DT_BGT vs Yoga | 0 | 0 | 0.78674534 | 0.2641252 | NA | NA | 0.78674534 | 0.2641252 | NA | NA | NA | NA |
| Mul_C vs Mul_D | 2 | 0.14499119 | -0.0438565 | 0.14912477 | 0.38162174 | 0.39163272 | -0.1160085 | 0.16127405 | 0.49763019 | 0.42353926 | 1.17493285 | 0.24002158 |
| Mul_C vs NW | 1 | 0.40173214 | -0.0836348 | 0.28909652 | -0.3720199 | 0.45611523 | 0.11001357 | 0.37376189 | -0.4820335 | 0.58969403 | -0.8174298 | 0.41368288 |
| Mul_C vs Pialtes | 1 | 1 | 0.32847913 | 0.53902125 | 0.32847913 | 0.53902125 | NA | NA | NA | NA | NA | NA |
| Mul_C vs PT | 0 | 0 | 0.86555357 | 0.37406166 | NA | NA | 0.86555357 | 0.37406166 | NA | NA | NA | NA |
| Mul_C vs Qigong | 0 | 0 | -0.0158775 | 0.18549518 | NA | NA | -0.0158775 | 0.18549518 | NA | NA | NA | NA |
| Mul_C vs RA_GT | 0 | 0 | -0.0891571 | 0.17800253 | NA | NA | -0.0891571 | 0.17800253 | NA | NA | NA | NA |
| Mul_C vs RT | 12 | 0.54744432 | 0.01473087 | 0.0933593 | 0.25802961 | 0.12617916 | -0.279581 | 0.13877831 | 0.53761063 | 0.18756492 | 2.86626429 | 0.00415347 |
| Mul_C vs Stretch | 0 | 0 | -0.6312161 | 0.15350938 | NA | NA | -0.6312161 | 0.15350938 | NA | NA | NA | NA |
| Mul_C vs Tango | 0 | 0 | 0.00264242 | 0.18927471 | NA | NA | 0.00264242 | 0.18927471 | NA | NA | NA | NA |
| Mul_C vs TC | 1 | 0.11720403 | -0.0002008 | 0.15473562 | -0.118905 | 0.45197982 | 0.01555897 | 0.16468731 | -0.134464 | 0.48104851 | -0.2795227 | 0.77984369 |
| Mul_C vs TT | 2 | 0.13286815 | -0.2098259 | 0.12576664 | 0.08246876 | 0.34502852 | -0.2546133 | 0.1350588 | 0.3370821 | 0.37052066 | 0.9097525 | 0.36295305 |
| Mul_C vs VR | 0 | 0 | -0.1827683 | 0.18296359 | NA | NA | -0.1827683 | 0.18296359 | NA | NA | NA | NA |
| Mul_C vs WBV | 0 | 0 | -0.661733 | 0.37514609 | NA | NA | -0.661733 | 0.37514609 | NA | NA | NA | NA |
| Mul_C vs Yoga | 0 | 0 | 0.52375869 | 0.23232739 | NA | NA | 0.52375869 | 0.23232739 | NA | NA | NA | NA |
| Mul_D vs NW | 0 | 0 | -0.0397783 | 0.31449306 | NA | NA | -0.0397783 | 0.31449306 | NA | NA | NA | NA |
| Mul_D vs Pialtes | 0 | 0 | 0.37233559 | 0.55926926 | NA | NA | 0.37233559 | 0.55926926 | NA | NA | NA | NA |
| Mul_D vs PT | 0 | 0 | 0.90941003 | 0.39119696 | NA | NA | 0.90941003 | 0.39119696 | NA | NA | NA | NA |
| Mul_D vs Qigong | 0 | 0 | 0.027979 | 0.21578641 | NA | NA | 0.027979 | 0.21578641 | NA | NA | NA | NA |
| Mul_D vs RA_GT | 0 | 0 | -0.0453007 | 0.21246438 | NA | NA | -0.0453007 | 0.21246438 | NA | NA | NA | NA |
| Mul_D vs RT | 1 | 0.08096724 | 0.05858733 | 0.15743883 | -0.2502064 | 0.55329558 | 0.0857922 | 0.16422768 | -0.3359986 | 0.57715399 | -0.5821646 | 0.56045585 |
| Mul_D vs Stretch | 0 | 0 | -0.5873597 | 0.19455565 | NA | NA | -0.5873597 | 0.19455565 | NA | NA | NA | NA |
| Mul_D vs Tango | 0 | 0 | 0.04649887 | 0.22147269 | NA | NA | 0.04649887 | 0.22147269 | NA | NA | NA | NA |
| Mul_D vs TC | 0 | 0 | 0.0436557 | 0.19544564 | NA | NA | 0.0436557 | 0.19544564 | NA | NA | NA | NA |
| Mul_D vs TT | 0 | 0 | -0.1659694 | 0.17176448 | NA | NA | -0.1659694 | 0.17176448 | NA | NA | NA | NA |
| Mul_D vs VR | 1 | 0.14763518 | -0.1389119 | 0.20921409 | -0.7592523 | 0.54449763 | -0.0314649 | 0.22660953 | -0.7277874 | 0.58977076 | -1.2340174 | 0.21719642 |
| Mul_D vs WBV | 1 | 0.47833724 | -0.6178765 | 0.37771391 | -0.8206749 | 0.54612981 | -0.4319211 | 0.52295951 | -0.3887538 | 0.75613782 | -0.5141309 | 0.6071605 |
| Mul_D vs Yoga | 0 | 0 | 0.56761515 | 0.26046182 | NA | NA | 0.56761515 | 0.26046182 | NA | NA | NA | NA |
| NW vs Pialtes | 0 | 0 | 0.41211389 | 0.61165407 | NA | NA | 0.41211389 | 0.61165407 | NA | NA | NA | NA |
| NW vs PT | 0 | 0 | 0.94918833 | 0.46340402 | NA | NA | 0.94918833 | 0.46340402 | NA | NA | NA | NA |
| NW vs Qigong | 0 | 0 | 0.0677573 | 0.33193309 | NA | NA | 0.0677573 | 0.33193309 | NA | NA | NA | NA |
| NW vs RA_GT | 0 | 0 | -0.0055224 | 0.32467972 | NA | NA | -0.0055224 | 0.32467972 | NA | NA | NA | NA |
| NW vs RT | 0 | 0 | 0.09836563 | 0.29509975 | NA | NA | 0.09836563 | 0.29509975 | NA | NA | NA | NA |
| NW vs Stretch | 0 | 0 | -0.5475814 | 0.31573768 | NA | NA | -0.5475814 | 0.31573768 | NA | NA | NA | NA |
| NW vs Tango | 0 | 0 | 0.08627718 | 0.33287925 | NA | NA | 0.08627718 | 0.33287925 | NA | NA | NA | NA |
| NW vs TC | 0 | 0 | 0.083434 | 0.31647045 | NA | NA | 0.083434 | 0.31647045 | NA | NA | NA | NA |
| NW vs TT | 1 | 0.26090524 | -0.1261911 | 0.29461777 | -0.9344008 | 0.57678975 | 0.15911216 | 0.34269587 | -1.093513 | 0.67091496 | -1.6298831 | 0.1031262 |
| NW vs VR | 0 | 0 | -0.0991336 | 0.32839583 | NA | NA | -0.0991336 | 0.32839583 | NA | NA | NA | NA |
| NW vs WBV | 0 | 0 | -0.5780982 | 0.46457032 | NA | NA | -0.5780982 | 0.46457032 | NA | NA | NA | NA |
| NW vs Yoga | 0 | 0 | 0.60739345 | 0.36013624 | NA | NA | 0.60739345 | 0.36013624 | NA | NA | NA | NA |
| Pialtes vs PT | 0 | 0 | 0.53707444 | 0.6560991 | NA | NA | 0.53707444 | 0.6560991 | NA | NA | NA | NA |
| Pialtes vs Qigong | 0 | 0 | -0.3443566 | 0.57004593 | NA | NA | -0.3443566 | 0.57004593 | NA | NA | NA | NA |
| Pialtes vs RA_GT | 0 | 0 | -0.4176363 | 0.56765201 | NA | NA | -0.4176363 | 0.56765201 | NA | NA | NA | NA |
| Pialtes vs RT | 0 | 0 | -0.3137483 | 0.54704649 | NA | NA | -0.3137483 | 0.54704649 | NA | NA | NA | NA |
| Pialtes vs Stretch | 0 | 0 | -0.9596952 | 0.56045431 | NA | NA | -0.9596952 | 0.56045431 | NA | NA | NA | NA |
| Pialtes vs Tango | 0 | 0 | -0.3258367 | 0.57128698 | NA | NA | -0.3258367 | 0.57128698 | NA | NA | NA | NA |
| Pialtes vs TC | 0 | 0 | -0.3286799 | 0.56079142 | NA | NA | -0.3286799 | 0.56079142 | NA | NA | NA | NA |
| Pialtes vs TT | 0 | 0 | -0.538305 | 0.55349901 | NA | NA | -0.538305 | 0.55349901 | NA | NA | NA | NA |
| Pialtes vs VR | 0 | 0 | -0.5112475 | 0.56922718 | NA | NA | -0.5112475 | 0.56922718 | NA | NA | NA | NA |
| Pialtes vs WBV | 0 | 0 | -0.9902121 | 0.65671797 | NA | NA | -0.9902121 | 0.65671797 | NA | NA | NA | NA |
| Pialtes vs Yoga | 0 | 0 | 0.19527956 | 0.58695819 | NA | NA | 0.19527956 | 0.58695819 | NA | NA | NA | NA |
| PT vs Qigong | 0 | 0 | -0.881431 | 0.4049115 | NA | NA | -0.881431 | 0.4049115 | NA | NA | NA | NA |
| PT vs RA_GT | 0 | 0 | -0.9547107 | 0.40237201 | NA | NA | -0.9547107 | 0.40237201 | NA | NA | NA | NA |
| PT vs RT | 0 | 0 | -0.8508227 | 0.37455144 | NA | NA | -0.8508227 | 0.37455144 | NA | NA | NA | NA |
| PT vs Stretch | 0 | 0 | -1.4967697 | 0.39277419 | NA | NA | -1.4967697 | 0.39277419 | NA | NA | NA | NA |
| PT vs Tango | 0 | 0 | -0.8629112 | 0.40496732 | NA | NA | -0.8629112 | 0.40496732 | NA | NA | NA | NA |
| PT vs TC | 0 | 0 | -0.8657543 | 0.39254323 | NA | NA | -0.8657543 | 0.39254323 | NA | NA | NA | NA |
| PT vs TT | 0 | 0 | -1.0753794 | 0.38231811 | NA | NA | -1.0753794 | 0.38231811 | NA | NA | NA | NA |
| PT vs VR | 0 | 0 | -1.0483219 | 0.40482244 | NA | NA | -1.0483219 | 0.40482244 | NA | NA | NA | NA |
| PT vs WBV | 0 | 0 | -1.5272866 | 0.52043306 | NA | NA | -1.5272866 | 0.52043306 | NA | NA | NA | NA |
| PT vs Yoga | 1 | 0.56922736 | -0.3417949 | 0.37909635 | 0.04693243 | 0.50246605 | -0.8554631 | 0.57759782 | 0.90239555 | 0.76556605 | 1.17872984 | 0.23850577 |
| Qigong vs RA_GT | 0 | 0 | -0.0732797 | 0.2359539 | NA | NA | -0.0732797 | 0.2359539 | NA | NA | NA | NA |
| Qigong vs RT | 0 | 0 | 0.03060833 | 0.19142585 | NA | NA | 0.03060833 | 0.19142585 | NA | NA | NA | NA |
| Qigong vs Stretch | 0 | 0 | -0.6153387 | 0.21299634 | NA | NA | -0.6153387 | 0.21299634 | NA | NA | NA | NA |
| Qigong vs Tango | 0 | 0 | 0.01851987 | 0.24504437 | NA | NA | 0.01851987 | 0.24504437 | NA | NA | NA | NA |
| Qigong vs TC | 2 | 0.28080742 | 0.0156767 | 0.20375488 | -0.4096105 | 0.38450653 | 0.1817293 | 0.24026218 | -0.5913398 | 0.45339959 | -1.3042353 | 0.19215335 |
| Qigong vs TT | 0 | 0 | -0.1939484 | 0.20225297 | NA | NA | -0.1939484 | 0.20225297 | NA | NA | NA | NA |
| Qigong vs VR | 0 | 0 | -0.1668909 | 0.23965544 | NA | NA | -0.1668909 | 0.23965544 | NA | NA | NA | NA |
| Qigong vs WBV | 0 | 0 | -0.6458555 | 0.40586817 | NA | NA | -0.6458555 | 0.40586817 | NA | NA | NA | NA |
| Qigong vs Yoga | 0 | 0 | 0.53963615 | 0.28106413 | NA | NA | 0.53963615 | 0.28106413 | NA | NA | NA | NA |
| RA_GT vs RT | 0 | 0 | 0.103888 | 0.18232052 | NA | NA | 0.103888 | 0.18232052 | NA | NA | NA | NA |
| RA_GT vs Stretch | 2 | 0.3251213 | -0.542059 | 0.19192123 | -0.7901467 | 0.33658929 | -0.4225433 | 0.23362007 | -0.3676034 | 0.40972026 | -0.8972059 | 0.36960907 |
| RA_GT vs Tango | 0 | 0 | 0.09179955 | 0.24019749 | NA | NA | 0.09179955 | 0.24019749 | NA | NA | NA | NA |
| RA_GT vs TC | 0 | 0 | 0.08895638 | 0.2083393 | NA | NA | 0.08895638 | 0.2083393 | NA | NA | NA | NA |
| RA_GT vs TT | 3 | 0.48136806 | -0.1206687 | 0.16663041 | 0.03634057 | 0.24016826 | -0.2663969 | 0.23137936 | 0.30273743 | 0.33349244 | 0.90777899 | 0.363995 |
| RA_GT vs VR | 0 | 0 | -0.0936112 | 0.22303345 | NA | NA | -0.0936112 | 0.22303345 | NA | NA | NA | NA |
| RA_GT vs WBV | 0 | 0 | -0.5725759 | 0.39534066 | NA | NA | -0.5725759 | 0.39534066 | NA | NA | NA | NA |
| RA_GT vs Yoga | 0 | 0 | 0.61291582 | 0.27677295 | NA | NA | 0.61291582 | 0.27677295 | NA | NA | NA | NA |
| RT vs Stretch | 2 | 0.2482158 | -0.645947 | 0.15567547 | -0.4381931 | 0.31246795 | -0.7145409 | 0.17954511 | 0.27634772 | 0.3603785 | 0.76682632 | 0.44318478 |
| RT vs Tango | 2 | 0.22645815 | -0.0120885 | 0.18901958 | 0.09913765 | 0.39720326 | -0.0446504 | 0.21491408 | 0.14378809 | 0.45161764 | 0.31838457 | 0.75019324 |
| RT vs TC | 1 | 0.18595669 | -0.0149316 | 0.1584381 | 0.22800745 | 0.36741233 | -0.0704276 | 0.17560459 | 0.29843507 | 0.40722082 | 0.73285809 | 0.46364499 |
| RT vs TT | 2 | 0.17575652 | -0.2245567 | 0.1307845 | -0.2034558 | 0.31196117 | -0.2290562 | 0.14405505 | 0.02560037 | 0.34361553 | 0.07450295 | 0.94061019 |
| RT vs VR | 0 | 0 | -0.1974992 | 0.18848774 | NA | NA | -0.1974992 | 0.18848774 | NA | NA | NA | NA |
| RT vs WBV | 0 | 0 | -0.6764639 | 0.3779554 | NA | NA | -0.6764639 | 0.3779554 | NA | NA | NA | NA |
| RT vs Yoga | 1 | 0.38986057 | 0.50902782 | 0.22811296 | 0.31697638 | 0.36533838 | 0.63174286 | 0.29203534 | -0.3147665 | 0.46771441 | -0.6729886 | 0.50095453 |
| Stretch vs Tango | 0 | 0 | 0.63385853 | 0.22410326 | NA | NA | 0.63385853 | 0.22410326 | NA | NA | NA | NA |
| Stretch vs TC | 4 | 0.61525247 | 0.63101536 | 0.15619061 | 0.82759822 | 0.19912612 | 0.31665831 | 0.25180653 | 0.51093991 | 0.32102607 | 1.59158383 | 0.11147824 |
| Stretch vs TT | 0 | 0 | 0.42139025 | 0.16766471 | NA | NA | 0.42139025 | 0.16766471 | NA | NA | NA | NA |
| Stretch vs VR | 0 | 0 | 0.44844777 | 0.21272667 | NA | NA | 0.44844777 | 0.21272667 | NA | NA | NA | NA |
| Stretch vs WBV | 0 | 0 | -0.0305169 | 0.39007892 | NA | NA | -0.0305169 | 0.39007892 | NA | NA | NA | NA |
| Stretch vs Yoga | 0 | 0 | 1.1549748 | 0.261959 | NA | NA | 1.1549748 | 0.261959 | NA | NA | NA | NA |
| Tango vs TC | 0 | 0 | -0.0028432 | 0.22402536 | NA | NA | -0.0028432 | 0.22402536 | NA | NA | NA | NA |
| Tango vs TT | 0 | 0 | -0.2124683 | 0.20390694 | NA | NA | -0.2124683 | 0.20390694 | NA | NA | NA | NA |
| Tango vs VR | 0 | 0 | -0.1854108 | 0.24432605 | NA | NA | -0.1854108 | 0.24432605 | NA | NA | NA | NA |
| Tango vs WBV | 0 | 0 | -0.6643754 | 0.40843275 | NA | NA | -0.6643754 | 0.40843275 | NA | NA | NA | NA |
| Tango vs Yoga | 0 | 0 | 0.52111627 | 0.28071021 | NA | NA | 0.52111627 | 0.28071021 | NA | NA | NA | NA |
| TC vs TT | 0 | 0 | -0.2096251 | 0.17436045 | NA | NA | -0.2096251 | 0.17436045 | NA | NA | NA | NA |
| TC vs VR | 0 | 0 | -0.1825676 | 0.21858306 | NA | NA | -0.1825676 | 0.21858306 | NA | NA | NA | NA |
| TC vs WBV | 0 | 0 | -0.6615322 | 0.39343756 | NA | NA | -0.6615322 | 0.39343756 | NA | NA | NA | NA |
| TC vs Yoga | 0 | 0 | 0.52395944 | 0.26212143 | NA | NA | 0.52395944 | 0.26212143 | NA | NA | NA | NA |
| TT vs VR | 2 | 0.38590424 | 0.02705752 | 0.17868576 | 0.03448812 | 0.28764059 | 0.02238805 | 0.22801945 | 0.01210007 | 0.36705583 | 0.03296522 | 0.97370233 |
| TT vs WBV | 0 | 0 | -0.4519071 | 0.37807643 | NA | NA | -0.4519071 | 0.37807643 | NA | NA | NA | NA |
| TT vs Yoga | 0 | 0 | 0.73358455 | 0.24650668 | NA | NA | 0.73358455 | 0.24650668 | NA | NA | NA | NA |
| VR vs WBV | 1 | 0.50059997 | -0.4789646 | 0.37836211 | -0.0614227 | 0.53476409 | -0.8975099 | 0.53540616 | 0.83608726 | 0.75672477 | 1.10487629 | 0.2692132 |
| VR vs Yoga | 0 | 0 | 0.70652704 | 0.28046338 | NA | NA | 0.70652704 | 0.28046338 | NA | NA | NA | NA |
| WBV vs Yoga | 0 | 0 | 1.18549168 | 0.43095106 | NA | NA | 1.18549168 | 0.43095106 | NA | NA | NA | NA |

*NA* not available, *k* Number of studies providing direct evidence, *prop* Direct evidence proportion, *nma* Estimated treatment effect (SMD) in network meta-analysis, *direct* Estimated treatment effect (SMD) derived from direct evidence, *indir.* Estimated treatment effect (SMD) derived from indirect evidence, *Diff* Difference between direct and indirect treatment estimates, *z* z-value of test for disagreement (direct versus indirect), *p* p-value of test for disagreement (direct versus indirect), red is <0.05, yellow is 0.05-0.1.

# Appendix 12: Publication bias

As shown in the figure below, the funnel plot had good symmetry, and the linear fitting line (green) is not perpendicular to the 0 quadrant. Therefore, no small study effect was found for the primary outcome.

Fig 12.1 The funnel plot of change of motor symptoms of all physical activity types compared to control group.

# Appendix 13: Network Meta-Regression

***Changes in heterogeneity***

Below we present the results from the changes in heterogeneity in each meta-regression model.

| Covariate | Shared beta  (median and 95% CrI) | | Heterogeneity standard deviation (median and 95% CrI) | | % of variance explained | |
| --- | --- | --- | --- | --- | --- | --- |
| Motor symptoms | | | | | |
| None | - | 0.344 (0.28; 0.42) | | - | |
| Publish Year | -0.01 (-0.25; 0.23) | 0.344 (0.28; 0.42) | | 0% | |
| Mean Age | -0.03 (-0.24; 0.17) | 0.346 (0.28; 0.42) | | 0% | |
| Years of Diagnosis | 0.14 (-0.05; 0.32) | 0.337 (0.27; 0.41) | | -4.0% | |
| Hoehn and Yahr stage | -0.09 (-0.31; 1.46) | 0.345 (0.28; 0.42) | | 0% | |
| Percentage Male | 0.06 (-0.18; 0.28) | 0.342 (0.28; 0.41) | | -1.0% | |
| Sample Size | 0.04 (-0.09; 0.17) | 0.343 (0.28; 0.42) | | 0% | |
| Exercise Period | -0.32 (-0.52; -0.14) ^⋇^ | 0.330 (0.27; 0.40) | | -8.0% | |
| Exercise Frequency | -0.58 (-0.81; -0.36) ^⋇^ | 0.306 (0.24; 0.37) | | -20.9% | |
| Time of single session | -0.08 (-0.33; 0.17) | 0.345 (0.28; 0.42) | | 0% | |
| ON/OFF | -0.08 (-0.49; 0.33) | 0.343 (0.28; 0.41) | | 0% | |

CrI: Credible Interval; ⋇: Significant influence factors, 95% CrI does not contain zero.

## 13.1 Publication year

When the model was adjusted for centering value of publish year 2015, compared with the control group, the SMD value of physical activity types did not change significantly, and the hierarchy from the unadjusted model retained.

**Figure 13.1:** Forest plot overall change in motor symptoms adjusted for publish year 2015. Physical activity type are ranked according to SMD compared to CON. Treatments crossing the y-axis are not significantly different from CON. *SMD* standardized Mean Difference, *CrI* Credible Interval, *AE* Aerobic Exercise, *AQE* Aquatic Exercise, *BGT* Balance and Gait Training, *BGT_ECA* Balance and Gait Training with External Cue or Attention, *BGT_ICA* Balance and Gait Training with Internal Cue or Attention, *BWS_TT* Body Weight Support Treadmill Training, *CON* Control group, *CPP* Classic Physiotherapy Program, *DT_BGT* Dual Task Balance and Gait Training, *Mul_C* Multicomponent Exercise Program, *Mul_D* Multidisciplinary Exercise Program, *NW* Nordic Walking, *PT* Power Training, *RA_GT* Robotic Assisted Gait Training, *RT* Resistance Training, *TC* Tai Chi, *TT* Treadmill Training, *VR* Virtual Reality, *WBV* Whole Body Vibration.

## 13.2 Mean Age

When the model was adjusted for centering value of mean age 67, the SMD value of some physical activity types increased. However, the hierarchy from the unadjusted model retained.

**Figure 13.2:** Forest plot overall change in motor symptoms adjusted for mean age 67. Physical activity type are ranked according to SMD compared to CON. Treatments crossing the y-axis are not significantly different from CON. *SMD* standardized Mean Difference, *CrI* Credible Interval, *AE* Aerobic Exercise, *AQE* Aquatic Exercise, *BGT* Balance and Gait Training, *BGT_ECA* Balance and Gait Training with External Cue or Attention, *BGT_ICA* Balance and Gait Training with Internal Cue or Attention, *BWS_TT* Body Weight Support Treadmill Training, *CON* Control group, *CPP* Classic Physiotherapy Program, *DT_BGT* Dual Task Balance and Gait Training, *Mul_C* Multicomponent Exercise Program, *Mul_D* Multidisciplinary Exercise Program, *NW* Nordic Walking, *PT* Power Training, *RA_GT* Robotic Assisted Gait Training, *RT* Resistance Training, *TC* Tai Chi, *TT* Treadmill Training, *VR* Virtual Reality, *WBV* Whole Body Vibration.

## 13.3 Years of Diagnosis

When the model was adjusted for centering value of year of diagnosis 6.7, compared with the control group, the SMD value of physical activity types did not change significantly, and the hierarchy from the unadjusted model retained.

**Figure 13.3:** Forest plot overall change in motor symptoms adjusted for year of diagnosis 6.7. Physical activity type are ranked according to SMD compared to CON. Treatments crossing the y-axis are not significantly different from CON. *SMD* standardized Mean Difference, *CrI* Credible Interval, *AE* Aerobic Exercise, *AQE* Aquatic Exercise, *BGT* Balance and Gait Training, *BGT_ECA* Balance and Gait Training with External Cue or Attention, *BGT_ICA* Balance and Gait Training with Internal Cue or Attention, *BWS_TT* Body Weight Support Treadmill Training, *CON* Control group, *CPP* Classic Physiotherapy Program, *DT_BGT* Dual Task Balance and Gait Training, *Mul_C* Multicomponent Exercise Program, *Mul_D* Multidisciplinary Exercise Program, *NW* Nordic Walking, *PT* Power Training, *RA_GT* Robotic Assisted Gait Training, *RT* Resistance Training, *TC* Tai Chi, *TT* Treadmill Training, *VR* Virtual Reality, *WBV* Whole Body Vibration.

## 13.4 Hoehn and Yahr stage

When the model was adjusted for centering value of Hoehn and Yahr stage 2.4, compared with the control group, the SMD value of some physical activity types did not change significantly, and the hierarchy from the unadjusted model retained.

**Figure 13.4:** Forest plot overall change in motor symptoms adjusted for Hoehn and Yahr stage 2.4. Physical activity type are ranked according to SMD compared to CON. Treatments crossing the y-axis are not significantly different from CON. *SMD* standardized Mean Difference, *CrI* Credible Interval, *AE* Aerobic Exercise, *AQE* Aquatic Exercise, *BGT* Balance and Gait Training, *BGT_ECA* Balance and Gait Training with External Cue or Attention, *BGT_ICA* Balance and Gait Training with Internal Cue or Attention, *BWS_TT* Body Weight Support Treadmill Training, *CON* Control group, *CPP* Classic Physiotherapy Program, *DT_BGT* Dual Task Balance and Gait Training, *Mul_C* Multicomponent Exercise Program, *Mul_D* Multidisciplinary Exercise Program, *NW* Nordic Walking, *PT* Power Training, *RA_GT* Robotic Assisted Gait Training, *RT* Resistance Training, *TC* Tai Chi, *TT* Treadmill Training, *VR* Virtual Reality, *WBV* Whole Body Vibration.

## 13.5 Percentage Male

When the model was adjusted for centering value of percentage male 60%, compared with the control group, the SMD value of physical activity types did not change significantly, and the hierarchy from the unadjusted model retained.

**Figure 13.5:** Forest plot overall change in motor symptoms adjusted for percentage male 60%. Physical activity type are ranked according to SMD compared to CON. Treatments crossing the y-axis are not significantly different from CON. *SMD* standardized Mean Difference, *CrI* Credible Interval, *AE* Aerobic Exercise, *AQE* Aquatic Exercise, *BGT* Balance and Gait Training, *BGT_ECA* Balance and Gait Training with External Cue or Attention, *BGT_ICA* Balance and Gait Training with Internal Cue or Attention, *BWS_TT* Body Weight Support Treadmill Training, *CON* Control group, *CPP* Classic Physiotherapy Program, *DT_BGT* Dual Task Balance and Gait Training, *Mul_C* Multicomponent Exercise Program, *Mul_D* Multidisciplinary Exercise Program, *NW* Nordic Walking, *PT* Power Training, *RA_GT* Robotic Assisted Gait Training, *RT* Resistance Training, *TC* Tai Chi, *TT* Treadmill Training, *VR* Virtual Reality, *WBV* Whole Body Vibration.

## 13.6 Sample Size

When the model was adjusted for centering value of sample size 25, compared with the control group, the SMD value of physical activity types did not change significantly, and the hierarchy from the unadjusted model retained.

**Figure 13.6:** Forest plot overall change in motor symptoms adjusted for sample size 25. Physical activity type are ranked according to SMD compared to CON. Treatments crossing the y-axis are not significantly different from CON. *SMD* standardized Mean Difference, *CrI* Credible Interval, *AE* Aerobic Exercise, *AQE* Aquatic Exercise, *BGT* Balance and Gait Training, *BGT_ECA* Balance and Gait Training with External Cue or Attention, *BGT_ICA* Balance and Gait Training with Internal Cue or Attention, *BWS_TT* Body Weight Support Treadmill Training, *CON* Control group, *CPP* Classic Physiotherapy Program, *DT_BGT* Dual Task Balance and Gait Training, *Mul_C* Multicomponent Exercise Program, *Mul_D* Multidisciplinary Exercise Program, *NW* Nordic Walking, *PT* Power Training, *RA_GT* Robotic Assisted Gait Training, *RT* Resistance Training, *TC* Tai Chi, *TT* Treadmill Training, *VR* Virtual Reality, *WBV* Whole Body Vibration.

## 13.7 Exercise Period

When the model was adjusted for long-term exercise (exercise period ≥ 24 weeks), the SMD value of physical activity types increased. However, the hierarchy from the unadjusted model retained. In addition, the pooled regression coefficient showed that compare to short-term exercise, long-term exercise in motor symptoms there is -0.32 (-0.52; -0.14) difference in SMD.

**Figure 13.7:** Forest plot overall change in motor symptoms adjusted for long-term exercise. Physical activity type are ranked according to SMD compared to CON. Treatments crossing the y-axis are not significantly different from CON. *SMD* standardized Mean Difference, *CrI* Credible Interval, *AE* Aerobic Exercise, *AQE* Aquatic Exercise, *BGT* Balance and Gait Training, *BGT_ECA* Balance and Gait Training with External Cue or Attention, *BGT_ICA* Balance and Gait Training with Internal Cue or Attention, *BWS_TT* Body Weight Support Treadmill Training, *CON* Control group, *CPP* Classic Physiotherapy Program, *DT_BGT* Dual Task Balance and Gait Training, *Mul_C* Multicomponent Exercise Program, *Mul_D* Multidisciplinary Exercise Program, *NW* Nordic Walking, *PT* Power Training, *RA_GT* Robotic Assisted Gait Training, *RT* Resistance Training, *TC* Tai Chi, *TT* Treadmill Training, *VR* Virtual Reality, *WBV* Whole Body Vibration.

## 13.8 Exercise Frequency

When the model was adjusted for exercise frequency ≥ 5, the SMD value of physical activity types increased. However, the hierarchy from the unadjusted model retained. In addition, the pooled regression coefficient suggests that for one time of exercise frequency increase in motor symptoms there is -0.58 (-0.81; -0.36) difference in SMD.

**Figure 13.8:** Forest plot overall change in motor symptoms adjusted for exercise frequency 5. Physical activity type are ranked according to SMD compared to CON. Treatments crossing the y-axis are not significantly different from CON. *SMD* standardized Mean Difference, *CrI* Credible Interval, *AE* Aerobic Exercise, *AQE* Aquatic Exercise, *BGT* Balance and Gait Training, *BGT_ECA* Balance and Gait Training with External Cue or Attention, *BGT_ICA* Balance and Gait Training with Internal Cue or Attention, *BWS_TT* Body Weight Support Treadmill Training, *CON* Control group, *CPP* Classic Physiotherapy Program, *DT_BGT* Dual Task Balance and Gait Training, *Mul_C* Multicomponent Exercise Program, *Mul_D* Multidisciplinary Exercise Program, *NW* Nordic Walking, *PT* Power Training, *RA_GT* Robotic Assisted Gait Training, *RT* Resistance Training, *TC* Tai Chi, *TT* Treadmill Training, *VR* Virtual Reality, *WBV* Whole Body Vibration. *CPP* Classic Physiotherapy Program, *NW* Nordic Walking, *CON* Control group.

## 13.9 Time of single session

When the model was adjusted for centering value of time of single session 60 minutes, compared with the control group, the SMD value of physical activity types did not change significantly, and the hierarchy from the unadjusted model retained.

**Figure 13.9:** Forest plot overall change in motor symptoms adjusted for time of single session 60 minutes. Physical activity type are ranked according to SMD compared to CON. Treatments crossing the y-axis are not significantly different from CON. *SMD* standardized Mean Difference, *CrI* Credible Interval, *AE* Aerobic Exercise, *AQE* Aquatic Exercise, *BGT* Balance and Gait Training, *BGT_ECA* Balance and Gait Training with External Cue or Attention, *BGT_ICA* Balance and Gait Training with Internal Cue or Attention, *BWS_TT* Body Weight Support Treadmill Training, *CON* Control group, *CPP* Classic Physiotherapy Program, *DT_BGT* Dual Task Balance and Gait Training, *Mul_C* Multicomponent Exercise Program, *Mul_D* Multidisciplinary Exercise Program, *NW* Nordic Walking, *PT* Power Training, *RA_GT* Robotic Assisted Gait Training, *RT* Resistance Training, *TC* Tai Chi, *TT* Treadmill Training, *VR* Virtual Reality, *WBV* Whole Body Vibration.

## 13.10 ON/OFF

The outcome test situation are OFF (>12 hour withdrawal from medication) or ON (1-2 hour after taking their normal medication). When the model was adjusted for ON, the SMD value of physical activity types increased. However, the hierarchy from the unadjusted model retained.

**Figure 13.10:** Forest plot overall change in motor symptoms adjusted for ON. Physical activity type are ranked according to SMD compared to CON. Treatments crossing the y-axis are not significantly different from CON. *SMD* standardized Mean Difference, *CrI* Credible Interval, *PT* Power Training, *TC* Tai Chi, *RA_GT* Robotic Assisted Gait Training, *BWS_TT* Body Weight Support Treadmill Training, *Mul_D* Multidisciplinary Exercise Program *AE* Aerobic Exercise, *BGT_ECA* Balance and Gait Training with External Cue or Attention, *DT_BGT* Dual Task Balance and Gait Training, *BGT_ICA* Balance and Gait Training with Internal Cue or Attention, *Mul_C* Multicomponent Exercise Program, RT Resistance Training, *AQE* Aquatic Exercise, *VR* Virtual Reality, *TT* Treadmill Training, *BGT* Balance and Gait Training, *WBV* Whole Body Vibration, *CPP* Classic Physiotherapy Program, *NW* Nordic Walking, *CON* Control group

# Appendix 14: Sensitivity analyses

We assessed the sensitivity of our findings by repeating each network meta-analysis after excluding studies at TESTEX scale <11 points, sample size less than 20, exercise duration less than 4 weeks and more than 24 weeks, exercise frequency less than 2 and more than 4, OFF state during the test, Chinese studies, unpublished studies, and data were extracted using GetData and estimated standard deviations value.

***Changes in heterogeneity***

Below we present the results from the changes in heterogeneity in each sensitivity analysis.

| Including only studies with | Number of studies included | τ | % of variance explained |
| --- | --- | --- | --- |
| None | - | 0.344 | - |
| studies at overall low risk of bias | 52 | 0.253 | -45.9% |
| Sample size ≥20 | 59 | 0.345 | 0% |
| Exercise period 4-24 weeks | 118 | 0.313 | -17.2% |
| Exercise frequency 2-4 | 99 | 0.219 | -59.5% |
| ON state during the test | 122 | 0.348 | 0% |
| Non-Chinese literature | 135 | 0.311 | -18.3% |
| Original data and standard deviations | 120 | 0.370 | 15.7% |

## 14.1 Exclude studies at TESTEX scale <11 points

After excluding trials with TESTEX scale <11 points, the hierarchy did not change significantly.

Figure 14.1: Forest plot of studies at low RoB. Physical activity type are ranked according to SMD compared to CON. Treatments crossing the y-axis are not significantly different from CON. *SMD* standardized Mean Difference, *CrI* Credible Interval, *AE* Aerobic Exercise, *AQE* Aquatic Exercise, *BGT* Balance and Gait Training, *BGT_ECA* Balance and Gait Training with External Cue or Attention, *BWS_TT* Body Weight Support Treadmill Training, *CON* Control group, *CPP* Classic Physiotherapy Program, *DT_BGT* Dual Task Balance and Gait Training, *Mul_C* Multicomponent Exercise Program, *Mul_D* Multidisciplinary Exercise Program, *RA_GT* Robotic Assisted Gait Training, *RT* Resistance Training, *TC* Tai Chi, *TT* Treadmill Training, *VR* Virtual Reality, *WBV* Whole Body Vibration.

## 14.2 Exclude studies with sample size of less than 20

Pilates, WBV, and BGT_ICA lack trails with large sample size. The hierarchy did not change significantly.

Figure 14.2: Forest plot of sample size ≥20. Treatments are ranked according to SMD compared to CON. Treatments crossing the y-axis are not significantly different from CON. *SMD* standardized Mean Difference, *CrI* Credible Interval, *AE* Aerobic Exercise, *AQE* Aquatic Exercise, *BGT* Balance and Gait Training, *BGT_ECA* Balance and Gait Training with External Cue or Attention, *BWS_TT* Body Weight Support Treadmill Training, *CON* Control group, *CPP* Classic Physiotherapy Program, *DT_BGT* Dual Task Balance and Gait Training, *Mul_C* Multicomponent Exercise Program, *Mul_D* Multidisciplinary Exercise Program, *RA_GT* Robotic Assisted Gait Training, *RT* Resistance Training, *TC* Tai Chi, *TT* Treadmill Training, *VR* Virtual Reality, *WBV* Whole Body Vibration.

## 14.3 Exclude studies with exercise period less than 4 and more than 24

After excluding studies with exercise frequency less than 4 and more than 24, the hierarchy did not change significantly.

Figure 14.3: Forest plot of exercise period 4-24 weeks. Physical activity type are ranked according to SMD compared to CON. Treatments crossing the y-axis are not significantly different from CON. *SMD* standardized Mean Difference, *CrI* Credible Interval, *AE* Aerobic Exercise, *AQE* Aquatic Exercise, *BGT* Balance and Gait Training, *BGT_ECA* Balance and Gait Training with External Cue or Attention, *BWS_TT* Body Weight Support Treadmill Training, *CON* Control group, *CPP* Classic Physiotherapy Program, *DT_BGT* Dual Task Balance and Gait Training, *Mul_C* Multicomponent Exercise Program, *Mul_D* Multidisciplinary Exercise Program, *RA_GT* Robotic Assisted Gait Training, *RT* Resistance Training, *TC* Tai Chi, *TT* Treadmill Training, *VR* Virtual Reality, *WBV* Whole Body Vibration.

## 14.4 Exclude studies with exercise frequency less than 2 and more than 4

After excluding studies with exercise frequency less than 2 and more than 4, the hierarchy did not change significantly.

Figure 14.4: Forest plot of exercise frequency 2-4. Treatments are ranked according to SMD compared to CON. Treatments crossing the y-axis are not significantly different from CON. *SMD* standardized Mean Difference, *CrI* Credible Interval, *AE* Aerobic Exercise, *AQE* Aquatic Exercise, *BGT* Balance and Gait Training, *BGT_ECA* Balance and Gait Training with External Cue or Attention, *BWS_TT* Body Weight Support Treadmill Training, *CON* Control group, *CPP* Classic Physiotherapy Program, *DT_BGT* Dual Task Balance and Gait Training, *Mul_C* Multicomponent Exercise Program, *Mul_D* Multidisciplinary Exercise Program, *NW* Nordic Walking, *PT* Power Training, *RA_GT* Robotic Assisted Gait Training, *RT* Resistance Training, *TC* Tai Chi, *TT* Treadmill Training, *VR* Virtual Reality, *WBV* Whole Body Vibration.

## 14.5 Exclude studies that were OFF state during testing

After excluding studies that were OFF state during testing, the hierarchy did not change significantly.

Figure 14.5: Forest plot of studies that were ON state during the test. Treatments are ranked according to SMD compared to CON. Treatments crossing the y-axis are not significantly different from CON. *SMD* standardized Mean Difference, *CrI* Credible Interval, *AE* Aerobic Exercise, *AQE* Aquatic Exercise, *BGT* Balance and Gait Training, *BGT_ECA* Balance and Gait Training with External Cue or Attention, *BGT_ICA* Balance and Gait Training with Internal Cue or Attention, *BWS_TT* Body Weight Support Treadmill Training, *CON* Control group, *CPP* Classic Physiotherapy Program, *DT_BGT* Dual Task Balance and Gait Training, *Mul_C* Multicomponent Exercise Program, *Mul_D* Multidisciplinary Exercise Program, *NW* Nordic Walking, *PT* Power Training, *RA_GT* Robotic Assisted Gait Training, *RT* Resistance Training, *TC* Tai Chi, *TT* Treadmill Training, *VR* Virtual Reality, *WBV* Whole Body Vibration.

## 14.6 Exclude Chinese literature

After excluding Chinese literature, the hierarchy did not change significantly.

Figure 14.6: Forest plot of English literature. Treatments are ranked according to SMD compared to CON. Treatments crossing the y-axis are not significantly different from CON. *SMD* standardized Mean Difference, *CrI* Credible Interval, *AE* Aerobic Exercise, *AQE* Aquatic Exercise, *BGT* Balance and Gait Training, *BGT_ECA* Balance and Gait Training with External Cue or Attention, *BGT_ICA* Balance and Gait Training with Internal Cue or Attention, *BWS_TT* Body Weight Support Treadmill Training, *CON* Control group, *CPP* Classic Physiotherapy Program, *DT_BGT* Dual Task Balance and Gait Training, *Mul_C* Multicomponent Exercise Program, *Mul_D* Multidisciplinary Exercise Program, *NW* Nordic Walking, *PT* Power Training, *RA_GT* Robotic Assisted Gait Training, *RT* Resistance Training, *TC* Tai Chi, *TT* Treadmill Training, *VR* Virtual Reality, *WBV* Whole Body

## 14.7 Exclude studies that use GetData to extract data and estimated standard deviations value

After excluding studies that use GetData to extract data and estimated standard deviations value, the hierarchy did not change significantly.

Figure 14.7: Forest plot of original data and standard deviations. Treatments are ranked according to SMD compared to CON. Treatments crossing the y-axis are not significantly different from CON. *SMD* standardized Mean Difference, *CrI* Credible Interval, *AE* Aerobic Exercise, *AQE* Aquatic Exercise, *BGT* Balance and Gait Training, *BGT_ECA* Balance and Gait Training with External Cue or Attention, *BGT_ICA* Balance and Gait Training with Internal Cue or Attention, *BWS_TT* Body Weight Support Treadmill Training, *CON* Control group, *CPP* Classic Physiotherapy Program, *DT_BGT* Dual Task Balance and Gait Training, *Mul_C* Multicomponent Exercise Program, *Mul_D* Multidisciplinary Exercise Program, *NW* Nordic Walking, *PT* Power Training, *RA_GT* Robotic Assisted Gait Training, *RT* Resistance Training, *TC* Tai Chi, *TT* Treadmill Training, *VR* Virtual Reality, *WBV* Whole Body Vibration.

# Appendix 15: Characteristics of the sample

| Physical activity type | N(s) | n(s) | Male(s) | Male(%) | Age(m) | Duration ill in years(m) | Hoehn and Yahr stage(m) | Exercise duration(m) | Exercise frequency(m) | Course time(m) |
| --- | --- | --- | --- | --- | --- | --- | --- | --- | --- | --- |
| All included studies | | | | | | | | | | |
| Total | 250 | 13011 | 7340 | 56.41 | 67.12 | 6.84 | 2.35 | 12.34 | 3.10 | 54.16 |
| AE | 24 | 569 | 301 | 52.90 | 65.62 | 5.41 | 2.23 | 11.67 | 3.29 | 49.44 |
| AQE | 17 | 235 | 133 | 56.74 | 67.15 | 5.91 | 2.47 | 7.76 | 3.33 | 54.06 |
| BGT | 42 | 779 | 443 | 56.83 | 69.00 | 6.75 | 2.44 | 9.10 | 2.84 | 48.42 |
| BGT_ECA | 27 | 595 | 326 | 54.74 | 68.25 | 7.74 | 2.54 | 5.82 | 3.43 | 45.14 |
| BGT_ICA | 3 | 46 | 36 | 78.26 | 72.10 | 6.72 | 2.75 | 7.50 | 3.00 | 52.50 |
| BWS_TT | 5 | 61 | 21 | 34.43 | 64.48 | 5.68 | 2.58 | 6.00 | 4.00 | 37.50 |
| CON | 117 | 2964 | 1664 | 56.14 | 67.79 | 6.96 | 2.31 | 12.36 | 3.02 | 55.43 |
| CPP | 45 | 1180 | 615 | 52.14 | 66.61 | 6.53 | 2.45 | 16.81 | 3.67 | 61.92 |
| Dance | 10 | 153 | 61 | 39.87 | 68.15 | 7.38 | 2.25 | 11.40 | 1.80 | 62.22 |
| DT_BGT | 27 | 632 | 383 | 60.67 | 67.97 | 7.06 | 2.44 | 6.88 | 3.08 | 54.04 |
| Mul_C | 48 | 1471 | 850 | 57.76 | 66.65 | 6.56 | 2.31 | 14.59 | 3.33 | 60.51 |
| Mul_D | 20 | 1024 | 633 | 61.86 | 66.67 | 6.59 | 2.31 | 11.79 | 3.13 | 62.69 |
| NW | 4 | 53 | 20 | 37.74 | 64.24 | 5.43 | 2.23 | 7.50 | 3.50 | 60.00 |
| Pilates | 3 | 43 | 13 | 30.24 | 58.71 | 6.78 | 2.43 | 9.33 | 3.67 | 55.00 |
| PT | 4 | 63 | 37 | 58.73 | 70.83 | 7.45 | 2.18 | 10.00 | 2.50 | 56.25 |
| Qigong | 8 | 163 | 65 | 39.88 | 65.64 | 7.43 | 2.28 | 12.88 | 3.63 | 56.25 |
| RA_GT | 8 | 171 | 92 | 54.04 | 69.71 | 8.17 | 2.78 | 4.00 | 4.00 | 43.33 |
| RT | 42 | 906 | 513 | 56.62 | 65.83 | 7.36 | 2.35 | 24.48 | 2.32 | 59.96 |
| Stretch | 13 | 325 | 189 | 58.10 | 66.98 | 6.15 | 2.35 | 18.08 | 2.64 | 56.69 |
| Tai Chi | 13 | 276 | 168 | 60.76 | 68.51 | 6.83 | 2.31 | 14.62 | 2.31 | 56.54 |
| Tango | 10 | 161 | 93 | 57.77 | 66.43 | 6.43 | 2.23 | 15.10 | 2.00 | 56.67 |
| TT | 34 | 727 | 414 | 57.00 | 66.40 | 6.73 | 2.28 | 8.20 | 3.44 | 41.82 |
| VR | 23 | 470 | 281 | 59.89 | 67.34 | 7.53 | 2.25 | 7.30 | 3.41 | 44.43 |
| WBV | 3 | 61 | 35 | 57.38 | 64.19 | 6.21 | 2.89 | 6.33 | 5.33 | 73.33 |
| Yoga | 8 | 161 | 92 | 57.13 | 67.25 | 6.16 | 2.23 | 11.50 | 3.13 | 48.57 |
| Motor symptoms | | | | | | | | | | |
| Total | 141 | 7983 | 4264 | 53.41 | 67.29 | 6.81 | 2.36 | 11.63 | 3.26 | 55.41 |
| AE | 17 | 485 | 255 | 52.58 | 65.18 | 5.74 | 2.26 | 12.53 | 3.31 | 45.83 |
| AQE | 12 | 173 | 104 | 60.31 | 68.41 | 6.25 | 2.50 | 7.42 | 3.90 | 53.75 |
| BGT | 23 | 478 | 260 | 54.49 | 68.56 | 6.58 | 2.50 | 10.95 | 3.00 | 51.32 |
| BGT_ECA | 21 | 444 | 228 | 51.29 | 68.29 | 7.89 | 2.60 | 6.47 | 3.53 | 48.00 |
| BGT_ICA | 2 | 35 | 28 | 80.00 | 73.05 | 6.72 | NA | 11.00 | 3.00 | 60.00 |
| BWS_TT | 3 | 41 | 13 | 31.71 | 65.22 | 2.65 | 2.40 | 6.00 | 3.00 | 45.00 |
| CON | 71 | 1817 | 1038 | 57.11 | 67.76 | 6.78 | 2.21 | 12.92 | 3.10 | 57.68 |
| CPP | 29 | 624 | 302 | 48.39 | 67.80 | 6.83 | 2.43 | 12.45 | 3.39 | 58.38 |
| Dance | 8 | 100 | 31 | 31.00 | 67.92 | 7.48 | 2.29 | 11.38 | 1.75 | 62.86 |
| DT_BGT | 12 | 269 | 157 | 58.49 | 69.11 | 7.67 | 2.58 | 6.64 | 3.27 | 60.00 |
| Mul_C | 33 | 798 | 453 | 56.72 | 66.86 | 6.43 | 2.30 | 10.77 | 3.57 | 59.46 |
| Mul_D | 12 | 620 | 391 | 63.06 | 67.23 | 6.10 | 2.32 | 7.08 | 3.09 | 64.30 |
| NW | 3 | 39 | 20 | 51.28 | 63.97 | 5.43 | 2.44 | 7.00 | 3.33 | 60.00 |
| Pilates | 1 | 13 | 5 | 38.50 | 62.85 | 5.77 | 2.08 | 12.00 | 5.00 | 45.00 |
| PT | 2 | 28 | 17 | 60.72 | 71.60 | 6.60 | 2.20 | 12.00 | 2.00 | 60.00 |
| Qigong | 7 | 158 | 65 | 41.14 | 65.62 | 7.33 | 2.21 | 13.86 | 3.86 | 55.71 |
| RA_GT | 7 | 171 | 92 | 54.04 | 69.71 | 8.17 | 2.78 | 4.00 | 4.00 | 43.33 |
| RT | 24 | 565 | 321 | 56.90 | 65.72 | 7.48 | 2.36 | 20.50 | 2.32 | 61.14 |
| Stretch | 8 | 233 | 134 | 57.43 | 66.14 | 6.13 | 2.56 | 23.88 | 2.63 | 56.25 |
| Tai Chi | 10 | 239 | 148 | 61.80 | 68.55 | 6.81 | 2.29 | 15.40 | 2.50 | 55.50 |
| Tango | 7 | 115 | 61 | 53.05 | 65.96 | 6.26 | 2.29 | 16.57 | 1.86 | 58.33 |
| TT | 22 | 525 | 285 | 54.20 | 66.30 | 6.59 | 2.28 | 8.74 | 3.70 | 44.13 |
| VR | 9 | 195 | 106 | 54.61 | 68.30 | 7.90 | 2.51 | 6.11 | 4.11 | 46.67 |
| WBV | 3 | 61 | 35 | 57.38 | 64.19 | 6.21 | 2.89 | 6.33 | 5.33 | 73.33 |
| Yoga | 6 | 137 | 76 | 55.46 | 65.71 | 6.15 | 2.22 | 12.00 | 3.00 | 52.00 |
| Balance | | | | | | | | | | |
| Total | 113 | 5201 | 2931 | 56.35 | 67.94 | 6.98 | 2.43 | 9.31 | 3.00 | 55.54 |
| Gait velocity | | | | | | | | | | |
| Total | 106 | 4780 | 2723 | 56.97 | 67.59 | 6.60 | 2.39 | 10.10 | 2.94 | 55.69 |
| Freezing of gait | | | | | | | | | | |
| Total | 35 | 1146 | 620 | 54.12 | 69.02 | 7.16 | 2.26 | 10.89 | 2.78 | 52.11 |
| Depression | | | | | | | | | | |
| Total | 34 | 2007 | 1010 | 50.32 | 66.41 | 6.36 | 2.24 | 12.73 | 3.08 | 53.86 |
| Anxiety | | | | | | | | | | |
| Total | 13 | 757 | 357 | 47.15 | 66.04 | 5.86 | 2.13 | 16.08 | 2.77 | 48.08 |
| Sleep quality | | | | | | | | | | |
| Total | 10 | 452 | 221 | 48.89 | 65.36 | 6.64 | 2.14 | 15.60 | 3.45 | 42.78 |
| Cognition | | | | | | | | | | |
| Total | 50 | 3044 | 1714 | 56.32 | 66.90 | 7.31 | 2.26 | 14.22 | 2.72 | 58.62 |
| Walking distance | | | | | | | | | | |
| Total | 55 | 2955 | 1617 | 54.72 | 65.98 | 6.20 | 2.21 | 12.51 | 3.30 | 51.05 |
| Muscle strength | | | | | | | | | | |
| Total | 25 | 856 | 453 | 52.92 | 66.45 | 6.71 | 2.26 | 17.20 | 2.49 | 57.70 |
| Concern of falling | | | | | | | | | | |
| Total | 49 | 2858 | 1581 | 55.32 | 67.82 | 7.16 | 2.40 | 9.95 | 3.01 | 52.18 |

*N* number of studies, *n* number of sample size, *s* sum, *m* mean, *AE* Aerobic Exercise, *AQE* Aquatic Exercise, *BGT* Balance and Gait Training, *BGT_ECA* Balance and Gait Training with External Cue or Attention, *BWS_TT* Body Weight Support Treadmill Training, *CON* Control group, *CPP* Classic Physiotherapy Program, *DT_BGT* Dual Task Balance and Gait Training, *Mul_C* Multicomponent Exercise Program, *Mul_D* Multidisciplinary Exercise Program, *RA_GT* Robotic Assisted Gait Training, *RT* Resistance Training, *TC* Tai Chi, *TT* Treadmill Training, *VR* Virtual Reality, *WBV* Whole Body Vibration.

Appendix 16: Grading the evidence for primary outcome (motor symptoms) of the network meta-analysis using CINeMA

## 16.1 Summary of study limitations of the included studies

The figure below showed that RT, BWS_TT, Mul_D, Dance, TT, BGT_ECA, and DT_BG had high risk of bias (RoB) studies. In addition, there was no comparison of high RoB for each type of physical activity.


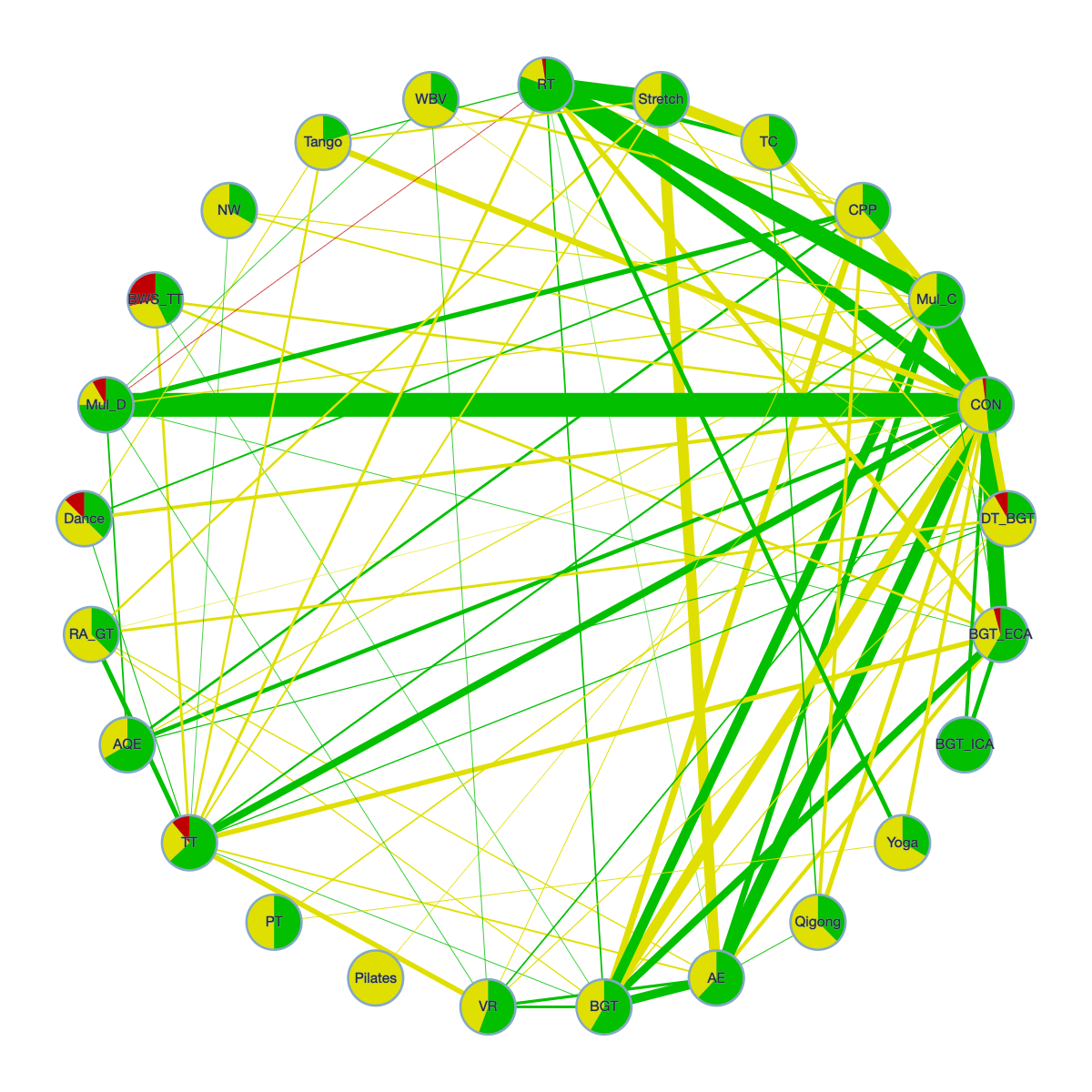


Figure 17.1 Network plot of study limitations of the included studies. Node size by equal size, node color by RoB. The colors in the circles indicate the percentage of low RoB studies (green), moderate RoB studies (yellow), high RoB studies (red) about each physical activity type. Edge width by sample size. Edge color by average RoB. The colors of the lines indicate the summative RoB assessment of each comparison. Low RoB is green, moderate RoB is yellow, high RoB is red. *AE* Aerobic Exercise, *AQE* Aquatic Exercise, *BGT* Balance and Gait Training, *BGT_ECA* Balance and Gait Training with External Cue or Attention, *BGT_ICA* Balance and Gait Training with Internal Cue or Attention, *BWS_TT* Body Weight Support Treadmill Training, *CON* Control group, *CPP* Classic Physiotherapy Program, *DT_BGT* Dual Task Balance and Gait Training, *Mul_C* Multicomponent Exercise Program, *Mul_D* Multidisciplinary Exercise Program, *NW* Nordic Walking, *PT* Power Training, *RA_GT* Robotic Assisted Gait Training, *RT* Resistance Training, *TC* Tai Chi, *TT* Treadmill Training, *VR* Virtual Reality, *WBV* Whole Body Vibration.

## 16.2 Contribution percentage of low, moderate, and high RoB comparisons to each network estimate

Low RoB is green, moderate RoB is yellow, high RoB is red.


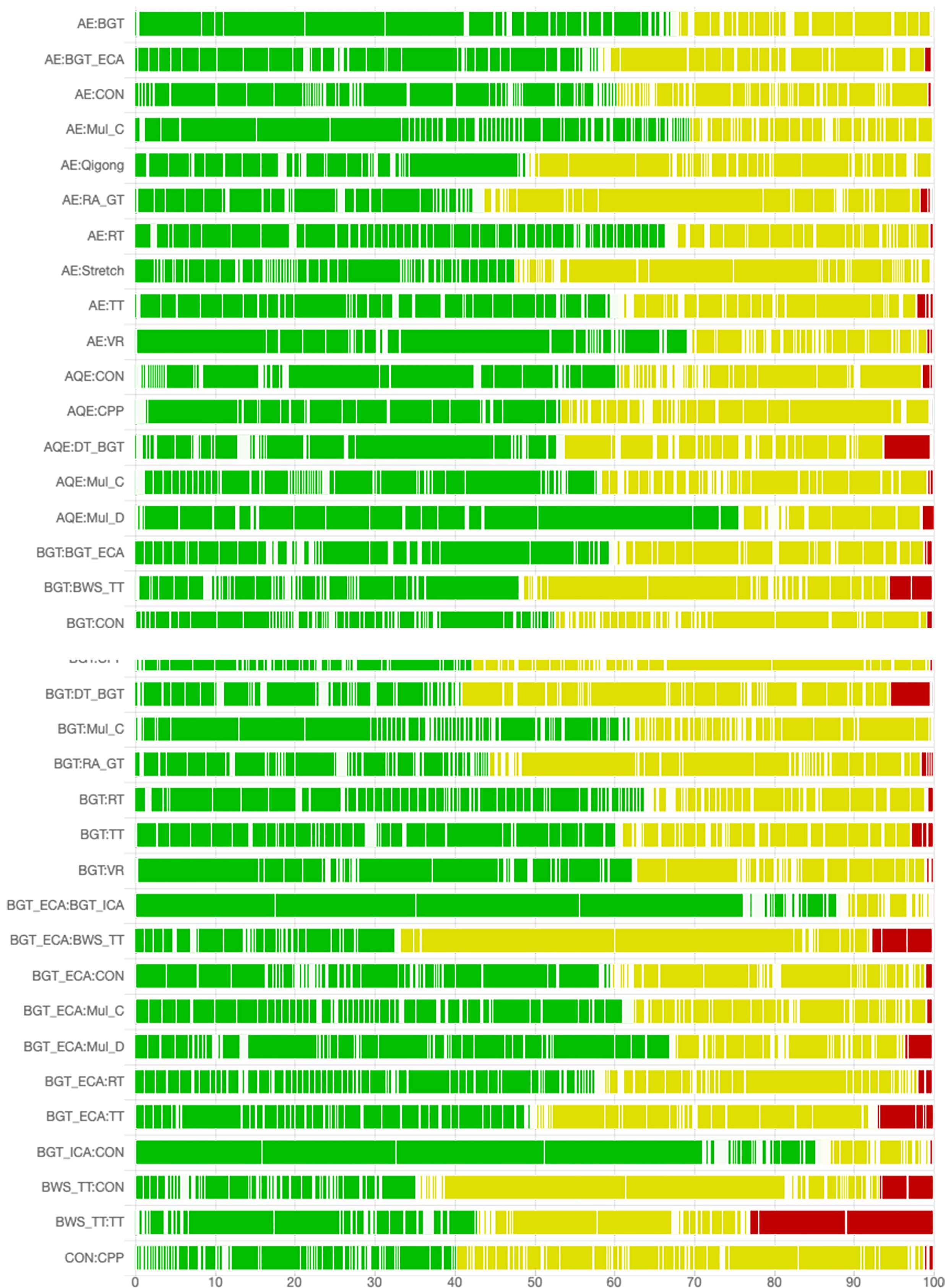


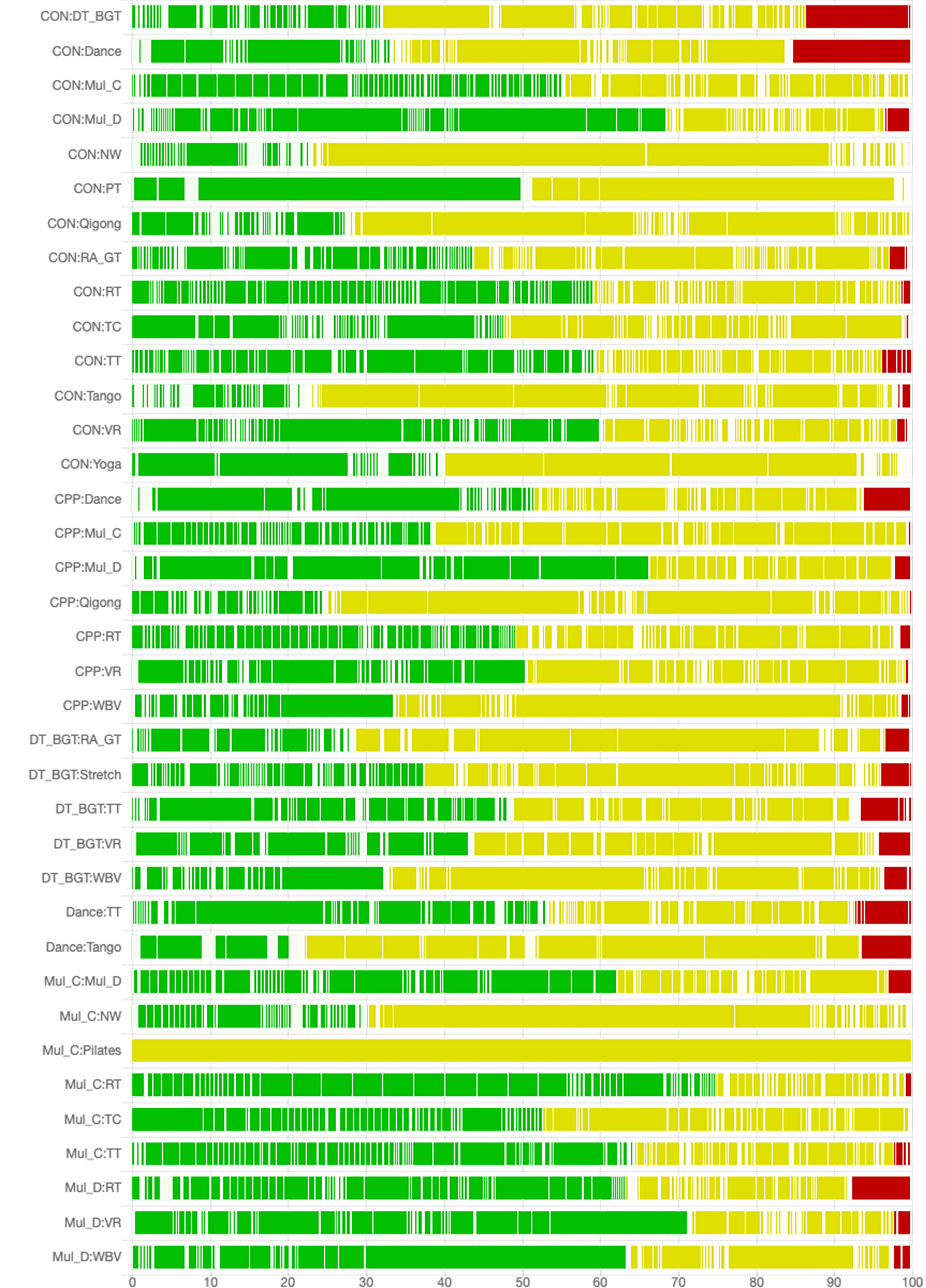


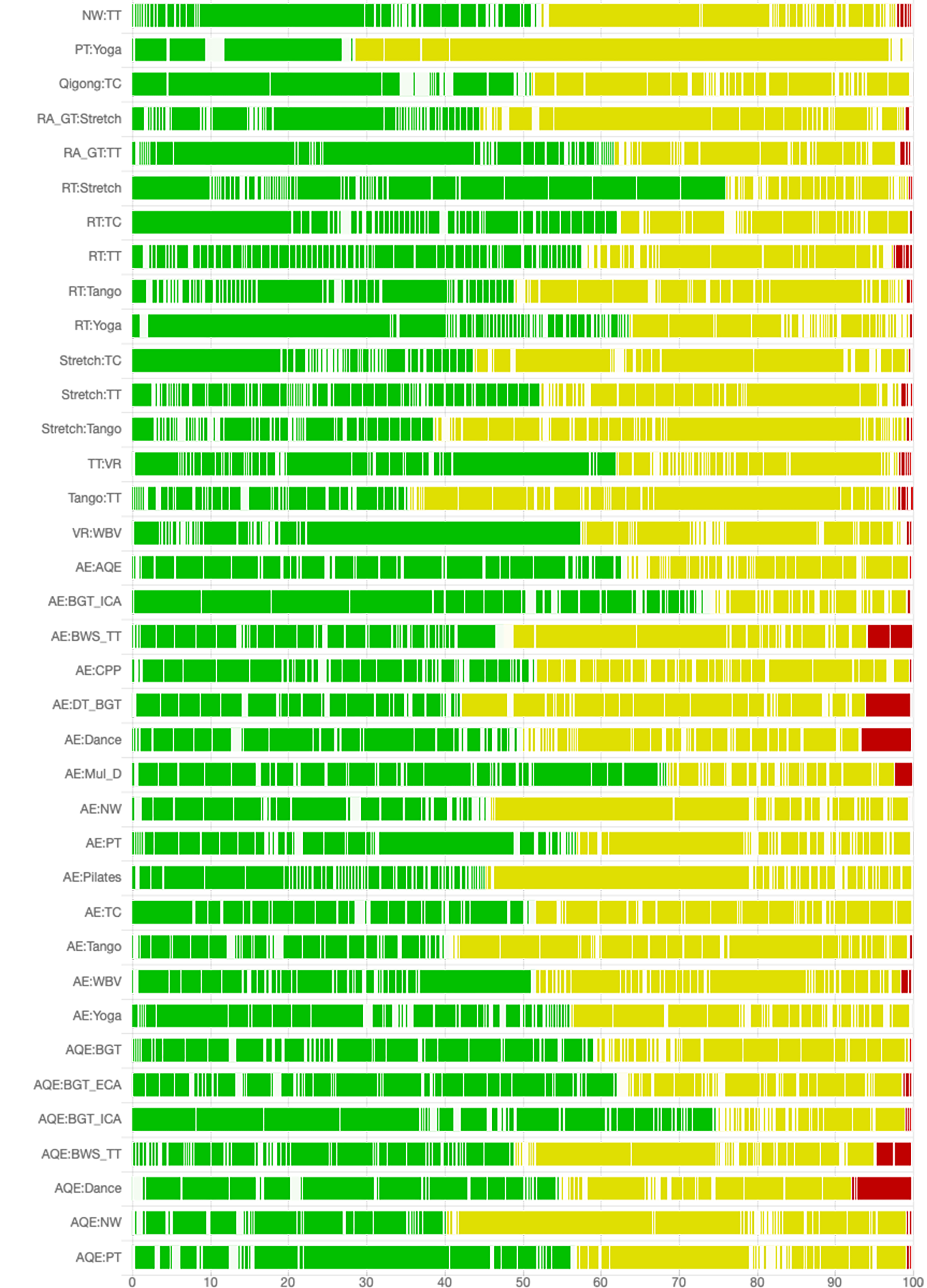


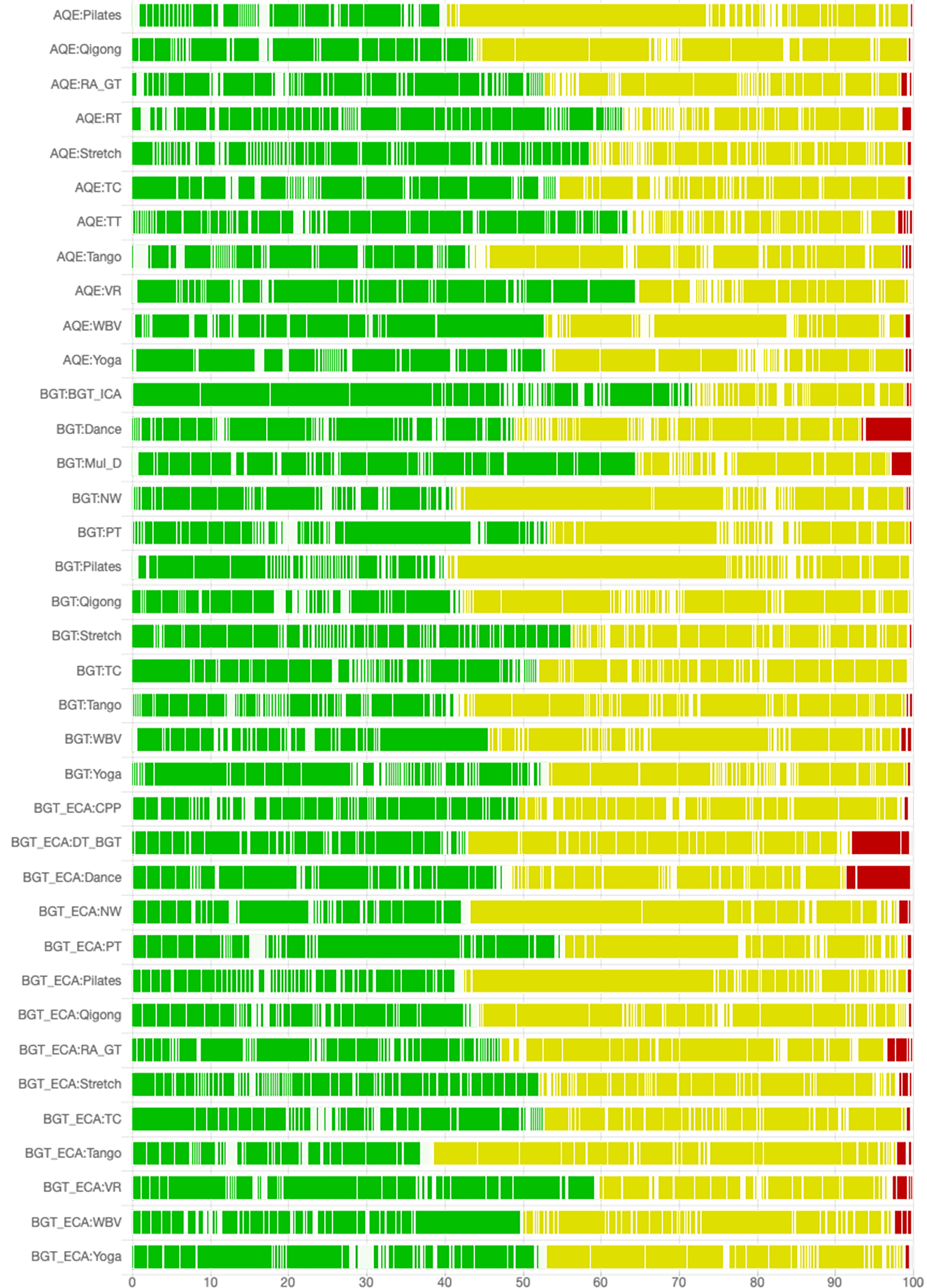


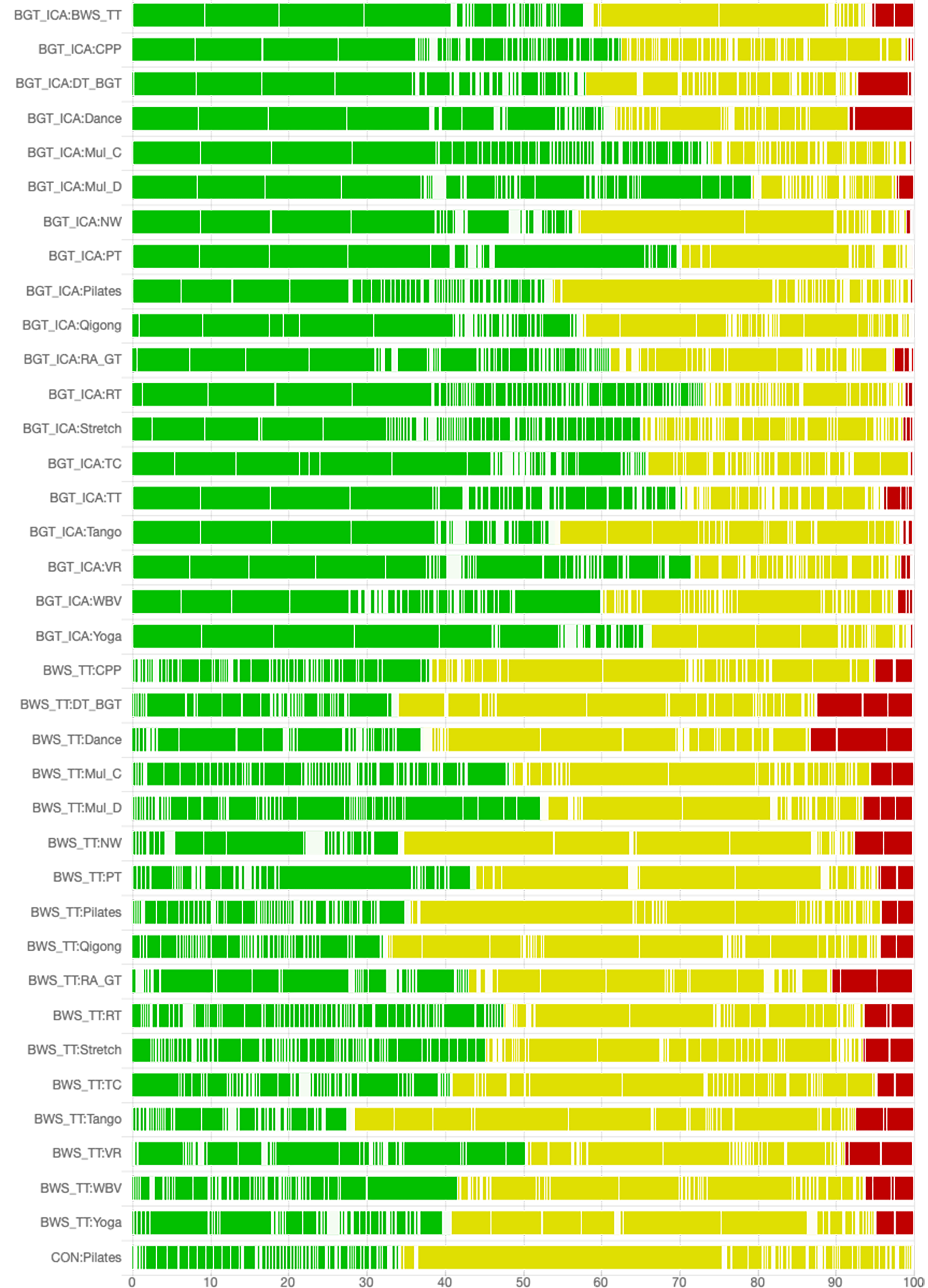


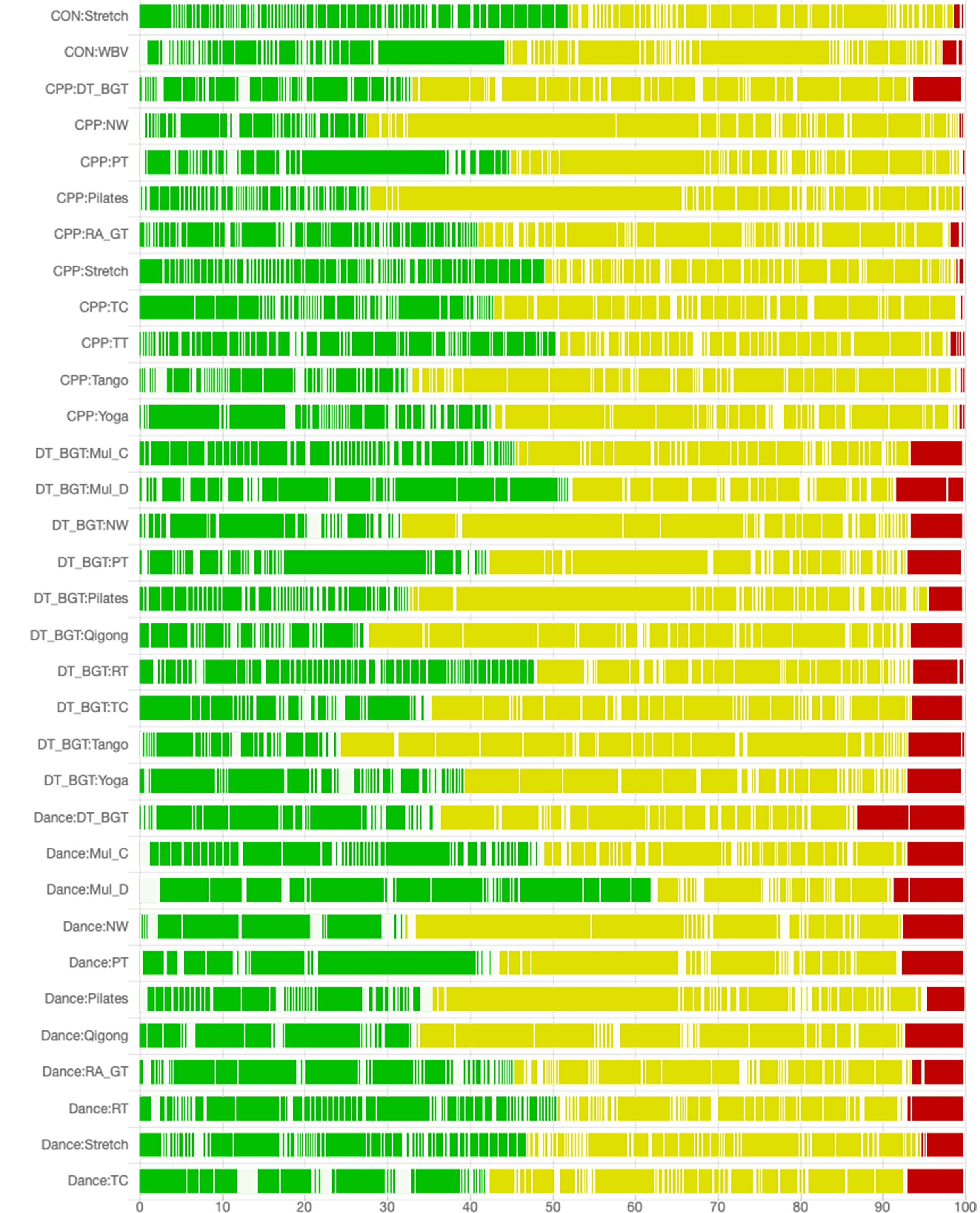


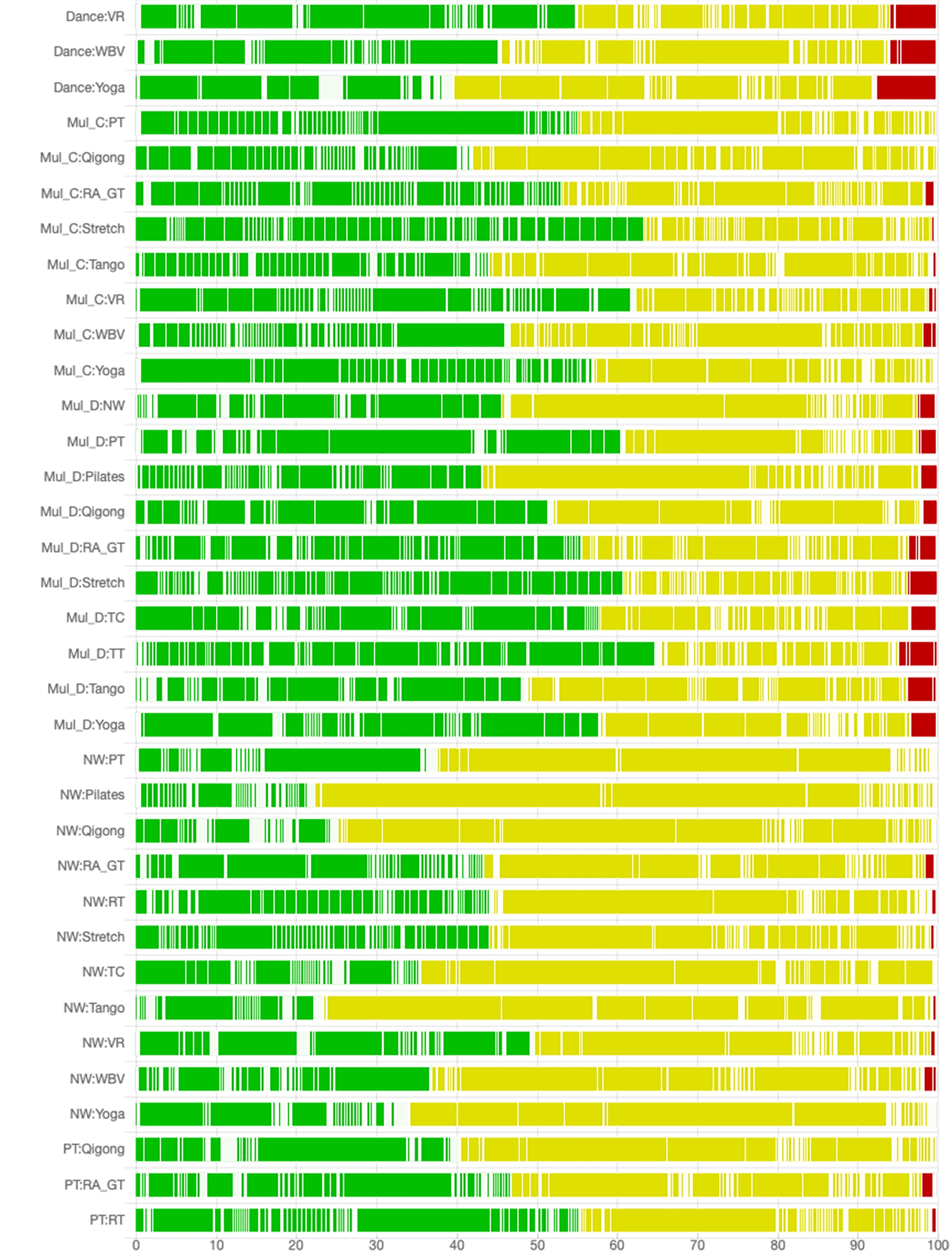


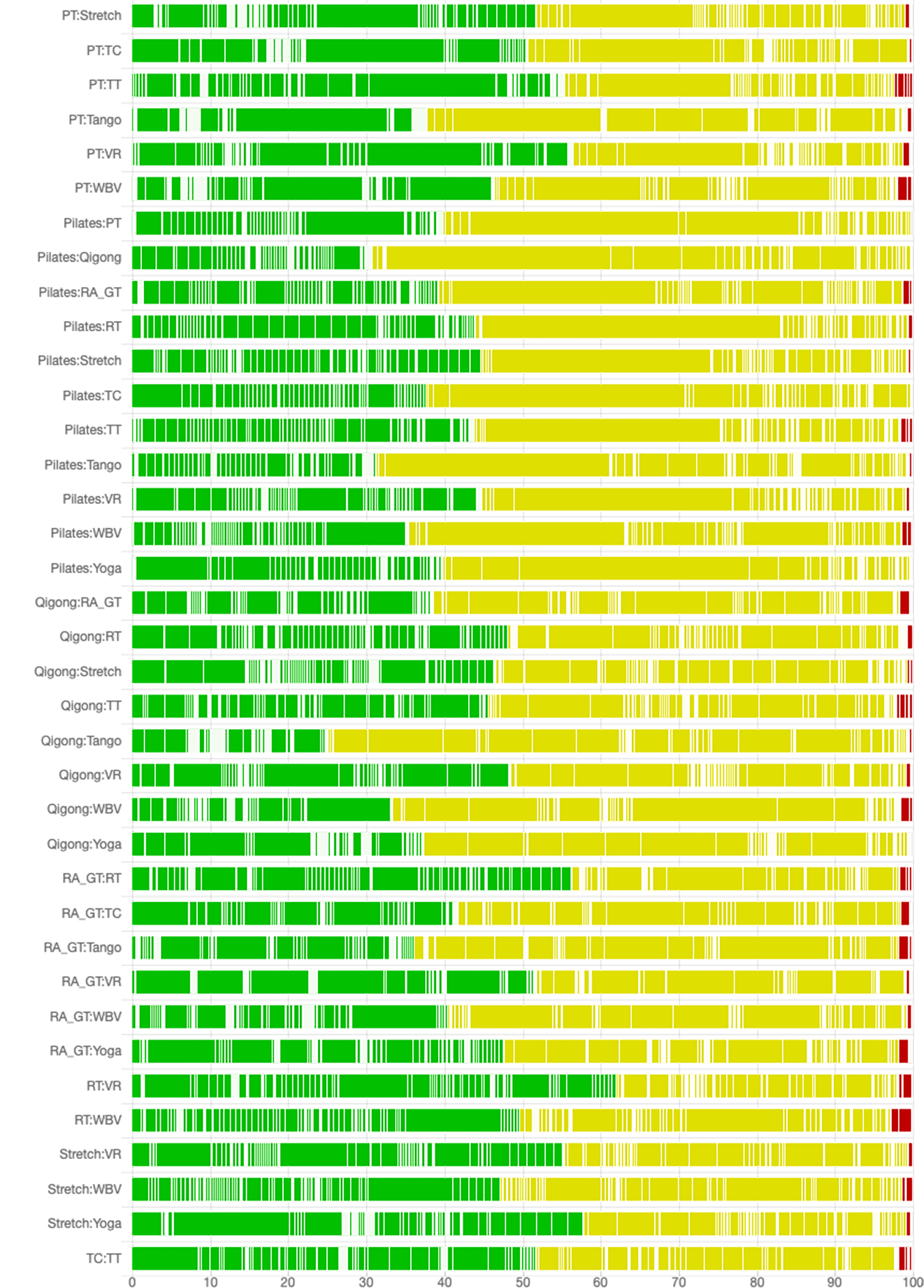


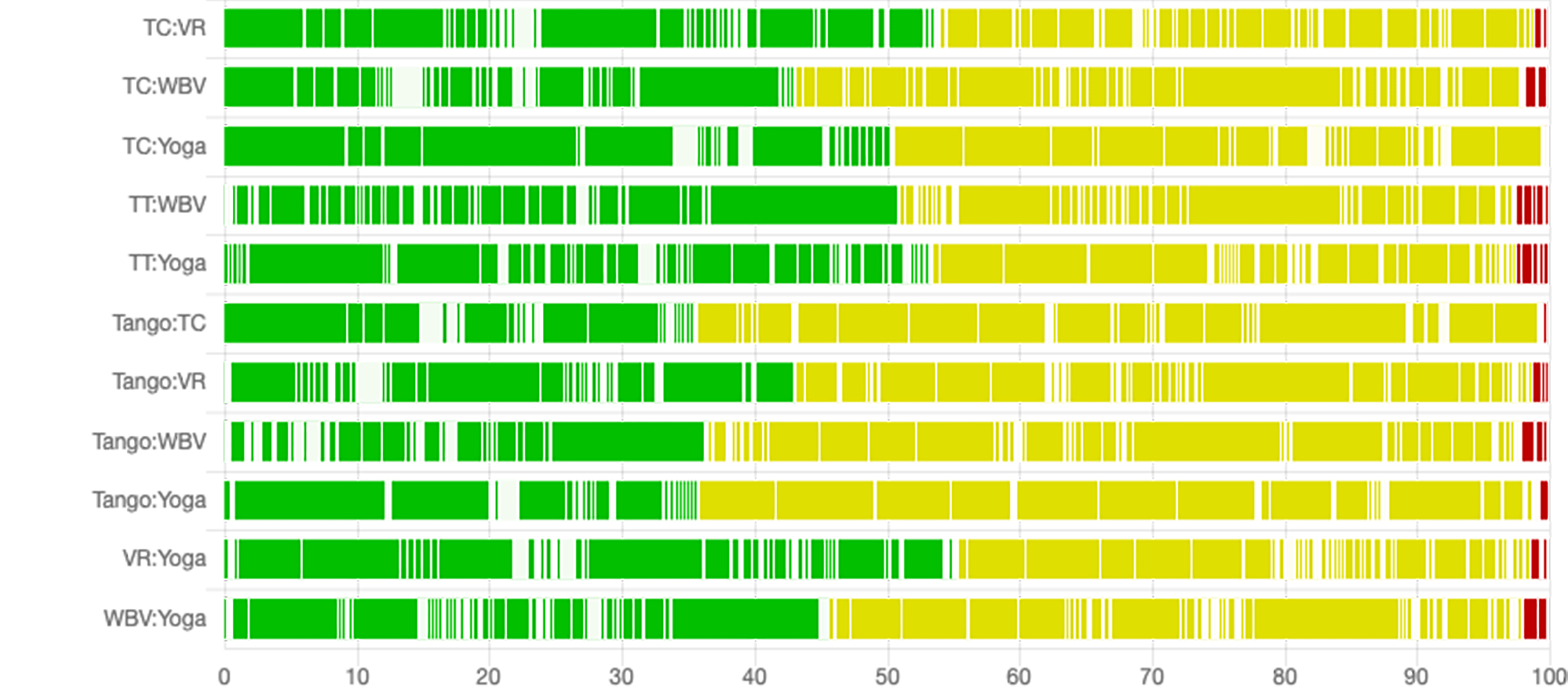


## 16.3 Reasons for downgrading

Based on the recommendations of the CINeMA online document (https://cinema.ispm.unibe.ch/), we only graded the results of the primary outcome (motor symptoms), and judged whether each module needs to be downgraded according to the following criteria.

***With-study bias***

We classified the quality evaluation results (appendix 8) of each included study into low-risk (TESTEX scale ≥11 points), moderate-risk (TESTEX scale 6-10 points), and high-risk (TESTEX scale ≤5 points). We selected the rule is average RoB. No need to downgrade when the result was “no concerns”, downgrade one level when “some concerns” and downgrade two level “major concerns”.

***Across-study bias (publication bias)***

Our search was relatively comprehensive, including published and unpublished studies. Due to the language is not limited, Chinese studies were also included in the scope of our inclusion. Even if it is possible that we missed other small unpublished experiments, then it does not seem to affect our results. In appendix 13, we evaluated the primary outcome of publication bias, and comparison-adjusted funnel plots for physical activity types vs. CON show no evidence of asymmetry. In appendix 14, we performed meta regression on the primary outcome with sample size as a covariate, and the results showed that the sample size did not significantly affect our results. Therefore, the primary outcome was deemed to have no publication bias. In addition, we independently evaluated publication bias for more than 10 studies comparing the same two types of exercise, and downgraded those with publication bias, otherwise it would not be downgraded.

***Indirectness***

Meta-regression was performed on the primary outcome with the baseline information (published years, sample size, average age, disease grade, diagnosis years, and the ratio of male to female subjects) of the included studies as covariates, and the results showed that the baseline information did not significantly affect the results . Therefore no indirectness was assumed and no comparison was downgraded for this reason.

***Imprecision***

The primary outcome (motor symptoms) of this network meta-analysis is a continuous variable, and the effect size measure for continuous outcomes chooses the standardized mean difference (SMD) of the change score (end-point minus baseline score) because the studies use different rating scales or units (appendix 6). Therefore, for CON comparisons the clinically meaningful threshold was set at a standardized mean difference of higher or lower than 0, and for the comparisons of the two types of physical activity, the threshold was set at SMD -0.1 and 0.1. If the confidence interval crossed one threshold, it will be downgraded by one level, and two thresholds will be downgraded by two levels.

***Heterogeneity***

For heterogeneity, we used the same threshold as the above clinically significant threshold and follow the recommendations automatically provided by CINeMA (https://cinema.ispm.unibe.ch/). No need to downgrade when the result was “no concerns”, downgrade one level when “some concerns” and downgrade two level when “major concerns”.

***Incoherence***

For incoherence, we will use global and local methods to test the inconsistency of the research results. For global inconsistency, we evaluated inconsistency statistically using the design-by-treatment test. In addition, we will assessment of local inconsistency by separating indirect from direct evidence (SIDE test) using the R netmeta package (appendix 12). No need to downgrade when p >0.1, downgrade one level when p was 0.05-0.1 and downgrade two level when p <0.05 .

***Summarising judgments across the 6 domains***

Τhe final output of CINeMA is a table with the level of concern for each of the 6 domains. we choose to summarise judgments across domains using the 4 levels of confidence of the GRADE approach: very low, low, moderate, or high.^1^ Due to factors that may reduce the confidence in a treatment effect may affect more than 1 domain. Indirectness includes consideration of intransitivity, which is manifested as statistical incoherence in the data. Heterogeneity will increase the imprecision of treatment effect, and may be related to the variability of bias within the study or the existence of reporting bias. In addition, in the presence of heterogeneity, the ability to detect important discontinuities will be reduced.^2^ Therefore, the 6 CINeMA domains should be considered jointly rather than in isolation to avoid downgrading the overall level of confidence more than once for related concerns. For example, the comparison between Mul_C and CON showed ‘major confers’ in both heterogeneity and inconsistency (appendix 17.4 pp 204), we only downgraded two levels, because imprecision, heterogeneity, and incoherence are interconnected.^3^

1. Puhan MA, Schünemann HJ, Murad MH, et al. A GRADE Working Group approach for rating the quality of treatment effect estimates from network meta-analysis. *BMJ* 2014; **349**.

2. Veroniki AA, Mavridis D, Higgins JPT, Salanti G. Characteristics of a loop of evidence that affect detection and estimation of inconsistency: a simulation study. *BMC Med Res Methodol* 2014; **14**: 106.

3. Nikolakopoulou A, Higgins JPT, Papakonstantinou T, et al. CINeMA: An approach for assessing confidence in the results of a network meta-analysis. *PLoS Med* 2020; **17**(4): e1003082.

## 16.4 CINeMA for the primary outcome “clinical symptoms”

| **Comparison** | **Number of studies** | **Within-study bias** | **Reporting bias** | **Indirectness** | **Imprecision** | **Heterogeneity** | **Incoherence** | **Confidence rating** |
| --- | --- | --- | --- | --- | --- | --- | --- | --- |
| AE:AQE | 0 | No concerns | Low risk | No concerns | Major concerns | No concerns | No concerns | Low |
| AE:BGT | 6 | No concerns | Low risk | No concerns | Major concerns | No concerns | No concerns | Low |
| AE:BGT_ECA | 3 | No concerns | Low risk | No concerns | Major concerns | No concerns | No concerns | Low |
| AE:BGT_ICA | 0 | No concerns | Low risk | No concerns | Major concerns | No concerns | No concerns | Low |
| AE:BWS_TT | 0 | Some concerns | Low risk | No concerns | Major concerns | No concerns | No concerns | Very low |
| AE:CON | 8 | No concerns | Low risk | No concerns | No concerns | Major concerns | No concerns | Low |
| AE:CPP | 0 | No concerns | Low risk | No concerns | No concerns | Major concerns | No concerns | Low |
| AE:Dance | 0 | Some concerns | Low risk | No concerns | Major concerns | No concerns | No concerns | Very low |
| AE:DT_BGT | 0 | Some concerns | Low risk | No concerns | Major concerns | No concerns | No concerns | Very low |
| AE:Mul_C | 3 | No concerns | Low risk | No concerns | Major concerns | No concerns | No concerns | Low |
| AE:Mul_D | 0 | No concerns | Low risk | No concerns | Major concerns | No concerns | No concerns | Low |
| AE:NW | 0 | Some concerns | Low risk | No concerns | Major concerns | No concerns | No concerns | Very low |
| AE:Pilates | 0 | Some concerns | Low risk | No concerns | Major concerns | No concerns | No concerns | Very low |
| AE:PT | 0 | No concerns | Low risk | No concerns | No concerns | Major concerns | No concerns | Low |
| AE:Qigong | 1 | Some concerns | Low risk | No concerns | Major concerns | No concerns | No concerns | Very low |
| AE:RA_GT | 1 | Some concerns | Low risk | No concerns | Major concerns | No concerns | No concerns | Very low |
| AE:RT | 1 | No concerns | Low risk | No concerns | Major concerns | No concerns | No concerns | Low |
| AE:Stretch | 4 | Some concerns | Low risk | No concerns | Major concerns | No concerns | No concerns | Very low |
| AE:Tango | 0 | Some concerns | Low risk | No concerns | Major concerns | No concerns | No concerns | Very low |
| AE:TC | 0 | No concerns | Low risk | No concerns | Major concerns | No concerns | No concerns | Low |
| AE:TT | 2 | No concerns | Low risk | No concerns | Major concerns | No concerns | No concerns | Low |
| AE:VR | 2 | No concerns | Low risk | No concerns | Major concerns | No concerns | No concerns | Low |
| AE:WBV | 0 | No concerns | Low risk | No concerns | Major concerns | No concerns | No concerns | Low |
| AE:Yoga | 0 | No concerns | Low risk | No concerns | No concerns | Major concerns | No concerns | Low |
| AQE:BGT | 0 | No concerns | Low risk | No concerns | Major concerns | No concerns | No concerns | Low |
| AQE:BGT_ECA | 0 | No concerns | Low risk | No concerns | Major concerns | No concerns | No concerns | Low |
| AQE:BGT_ICA | 0 | No concerns | Low risk | No concerns | Major concerns | No concerns | No concerns | Low |
| AQE:BWS_TT | 0 | Some concerns | Low risk | No concerns | Major concerns | No concerns | No concerns | Very low |
| AQE:CON | 5 | No concerns | Low risk | No concerns | No concerns | Major concerns | No concerns | Low |
| AQE:CPP | 3 | No concerns | Low risk | No concerns | Major concerns | No concerns | No concerns | Low |
| AQE:Dance | 0 | Some concerns | Low risk | No concerns | Major concerns | No concerns | No concerns | Very low |
| AQE:DT_BGT | 1 | Some concerns | Low risk | No concerns | Major concerns | No concerns | No concerns | Very low |
| AQE:Mul_C | 2 | No concerns | Low risk | No concerns | Major concerns | No concerns | No concerns | Low |
| AQE:Mul_D | 1 | No concerns | Low risk | No concerns | Major concerns | No concerns | No concerns | Low |
| AQE:NW | 0 | Some concerns | Low risk | No concerns | Major concerns | No concerns | No concerns | Very low |
| AQE:Pilates | 0 | Some concerns | Low risk | No concerns | Major concerns | No concerns | No concerns | Very low |
| AQE:PT | 0 | No concerns | Low risk | No concerns | No concerns | Major concerns | No concerns | Low |
| AQE:Qigong | 0 | Some concerns | Low risk | No concerns | Major concerns | No concerns | No concerns | Very low |
| AQE:RA_GT | 0 | No concerns | Low risk | No concerns | Major concerns | No concerns | No concerns | Low |
| AQE:RT | 0 | No concerns | Low risk | No concerns | Major concerns | No concerns | No concerns | Low |
| AQE:Stretch | 0 | No concerns | Low risk | No concerns | Major concerns | No concerns | No concerns | Low |
| AQE:Tango | 0 | Some concerns | Low risk | No concerns | Major concerns | No concerns | No concerns | Very low |
| AQE:TC | 0 | No concerns | Low risk | No concerns | Major concerns | No concerns | No concerns | Low |
| AQE:TT | 0 | No concerns | Low risk | No concerns | Major concerns | No concerns | No concerns | Low |
| AQE:VR | 0 | No concerns | Low risk | No concerns | Major concerns | No concerns | No concerns | Low |
| AQE:WBV | 0 | No concerns | Low risk | No concerns | Major concerns | No concerns | No concerns | Low |
| AQE:Yoga | 0 | No concerns | Low risk | No concerns | No concerns | Major concerns | No concerns | Low |
| BGT:BGT_ECA | 5 | No concerns | Low risk | No concerns | Major concerns | No concerns | No concerns | Low |
| BGT:BGT_ICA | 0 | No concerns | Low risk | No concerns | Major concerns | No concerns | No concerns | Low |
| BGT:BWS_TT | 1 | Some concerns | Low risk | No concerns | No concerns | Major concerns | No concerns | Very low |
| BGT:CON | 5 | No concerns | Low risk | No concerns | No concerns | Major concerns | No concerns | Low |
| BGT:CPP | 3 | Some concerns | Low risk | No concerns | Major concerns | No concerns | No concerns | Very low |
| BGT:Dance | 0 | Some concerns | Low risk | No concerns | Major concerns | No concerns | No concerns | Very low |
[truncated: 28,568 more chars]
